# Ion-pairing-modulated magnetic properties of thiaporphyrin Cu^II^ complex cations

**DOI:** 10.1039/d6sc04368b

**Published:** 2026-08-24

**Authors:** Masaki Fujita, Hiroki Horita, Ko Furukawa, Kunihisa Sugimoto, Kazuki Okazawa, Yasuteru Shigeta, Yukihide Ishibashi, Hiromitsu Maeda

**Affiliations:** a Department of Applied Chemistry, College of Life Sciences, Ritsumeikan University Kusatsu 525-8577 Japan maedahir@ph.ritsumei.ac.jp; b Center for Instrumental Analysis, Institute for Research Administration, Niigata University Niigata 950-2181 Japan; c Department of Chemistry, Faculty of Science and Engineering, Kindai University Higashiosaka 577-8502 Japan; d Institute of Pure and Applied Sciences, The University of Tsukuba Tsukuba 305-8577 Japan; e Center for Computational Sciences, The University of Tsukuba Tsukuba 305-8577 Japan; f Department of Applied Chemistry, Graduate School of Science and Engineering, Ehime University Matsuyama 790-8577 Japan

## Abstract

The metal complexation of π-electronic systems with partial charge compensation for metal ions produces positively charged π-electronic systems that can act as building blocks for ion-pairing assemblies. In this study, the Cu^II^ complexation of thiaporphyrins was investigated to afford paramagnetic π-electronic cations that modulate ion-pairing assembly modes in combination with coexisting anions. Counteranion-dependent stacking modes regulated spin–spin interactions between the Cu^II^ complex cations, resulting in antiferromagnetic properties in the solid state.

## Abstract 2

Solid-state magnetic properties of thiaporphyrin Cu^II^ ion pairs can be controlled by the assembly mode: dimer-based packing induces antiferromagnetic behaviour, whereas charge-by-charge assembly does not.

## Graphical abstract

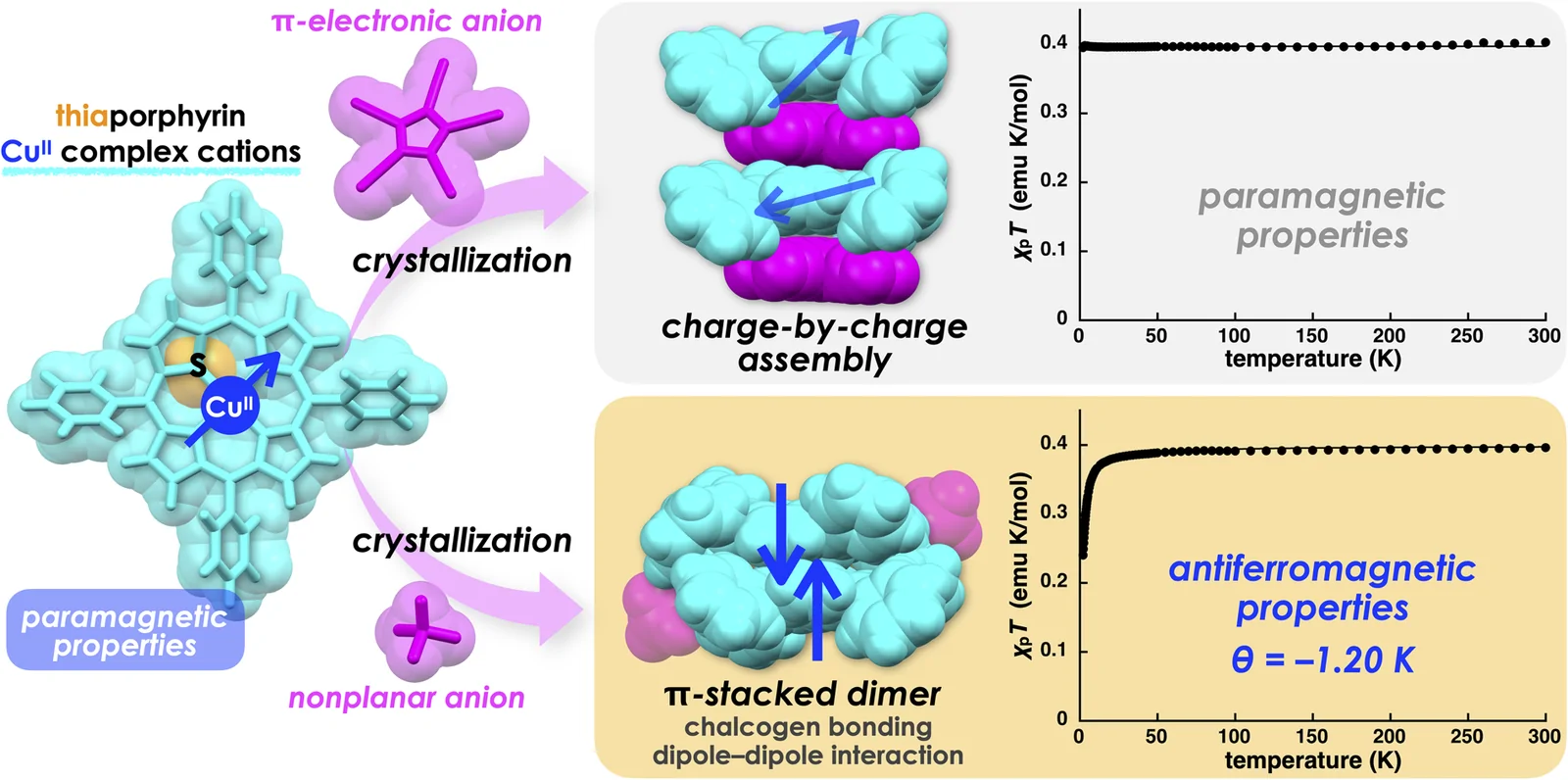

## Introduction

**Fig. 1:**
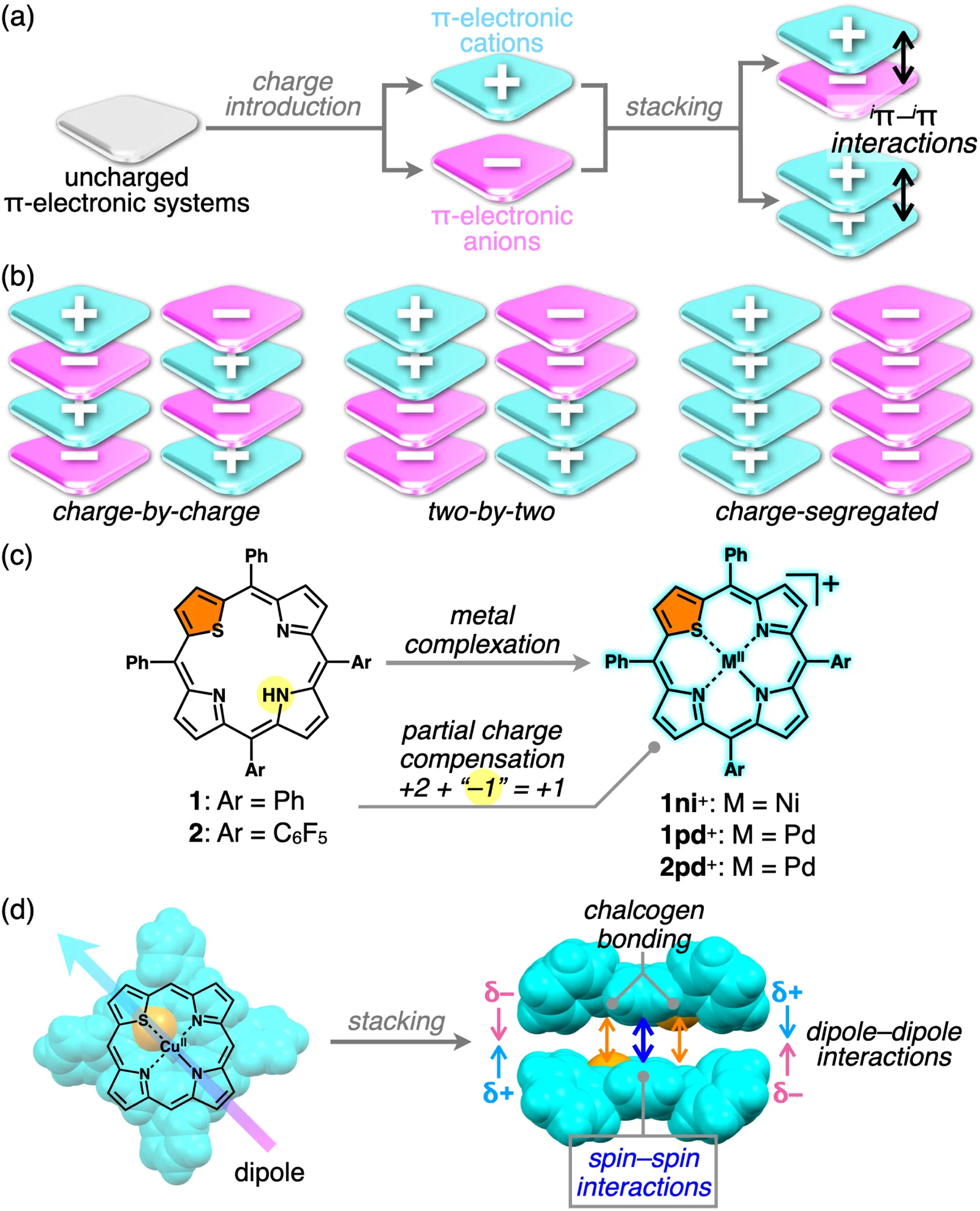
(a) Charged π-electronic systems that form *π-sips* and stacked dimers, (b) ion-pairing assemblies driven by ^*i*^π–^*i*^π interactions, (c) thiaporphyrin M^II^ complexes as π-electronic cations formed by partial charge compensation and (d) spin-induced π-electronic cations that form π-stacked dimers with magnetic properties.

The electronic and photophysical properties of π-electronic molecular assemblies are determined by their combination of molecular design and ordered arrangements.^1^ Magnetic properties, such as molecular magnetism and qubits, derived from electron spin are observed in stacked π-electronic systems.^2^ The stacking of π-electronic systems is achieved mainly by dispersion forces as fundamental intermolecular forces.^3^ Meanwhile, increased contributions of other fundamental intermolecular forces modulate the assembly modes of π-electronic systems and their resulting properties. More effective electrostatic forces, working for charged π-electronic systems, control their assembly modes in collaboration with dispersion forces. Synergetic electrostatic and dispersion forces for charged π-electronic systems, termed ^*i*^π–^*i*^π interactions, can arrange both oppositely and identically charged species (Fig. 1a),^4^ in contrast to conventional π–π interactions for uncharged π-electronic systems. Oppositely charged π-electronic systems form π-stacked ion pairs (*π-sips*) *via* attractive electrostatic forces and resulting charge-by-charge assembly (Fig. 1b). On the other hand, identically charged π-electronic systems can form their stacked dimers *via* intermolecular forces, thereby overcoming electrostatic repulsion. Such identically charged stacked dimers can be included in several assembly modes such as two-by-two and charge-segregated assemblies.

The introduction of (i) charge and (ii) electron spin can be achieved through metal complexation using π-electronic systems. For (i) charge introduction, partial charge compensation of metal ions with π-electronic ligands provides charged π-electronic systems,^4^ as seen in Au^III^ complexes of porphyrins as dianionic ligands.^5,6^ On the other hand, thiaporphyrins, which include a thiophene unit in the porphyrin macrocycle, serve as monoanionic ligands for producing π-electronic cations by complexation with dicationic metals (Fig. 1c).^7,8^ The Ni^II^ complex 1ni^+^ of 5,10,15,20-tetraphenylthiaporphyrin 1 and Pd^II^ complexes 1pd^+^ and 2pd^+^, prepared from 1 and 10,15-bis(pentafluorophenyl)-5,20-diphenylthiaporphyrin 2, respectively, afforded various ion-pairing assemblies.^8^ These thiaporphyrin cations, with slightly distorted geometries, formed stacked dimers in the solid state *via* S⋯N chalcogen-bonding^9^ and dipole–dipole interactions.^10^ Furthermore, for (ii) electron spin introduction, magnetic properties of porphyrins and related macrocycles are induced by Cu^II^ complexation,^11^ as demonstrated by antiferromagnetic interaction of dimeric octaethylporphyrin Cu^II^ complexes in frozen solutions,^11*a*^ the switching spin states of rotaxane-based porphyrin and phthalocyanine Cu^II^ complexes in solution^11*b*,*d*^ and quantum information retention in thin films comprising phthalocyanine free base and Cu^II^ complex.^11*c*^ Nevertheless, solid-state intermolecular spin–spin interactions in Cu^II^ complexes of π-electronic macrocycles have been reported in a few cases,^12^ as exemplified by Cu^II^ porphyrins,^12*e*^ Cu^II^ porphyrazines,^12*a*^ Cu^II^ phthalocyanines^12*b*,*c*,*f*^ and a Cu^II^ benzocorrole cation.^12*d*^ These limited precedents prompted us to investigate the potential of Cu^II^ complexes as building blocks of supramolecular spintronic materials.^13^ Therefore, charged Cu^II^ complexes have been focused on to induce magnetic properties in ion-pairing assemblies. Thiaporphyrin Cu^II^ complexes, as paramagnetic π-electronic cations, would exhibit spin–spin interactions *via* counteranion-dependent stacked dimerization (Fig. 1d). In this study, ion pairs of thiaporphyrin Cu^II^ complexes were synthesized to investigate the assembly modes and resulting magnetic behaviours controlled by coexisting anions.

## Results and discussion

### Synthesis and characterization of thiaporphyrin Cu^II^ complexes

**Fig. 2:**
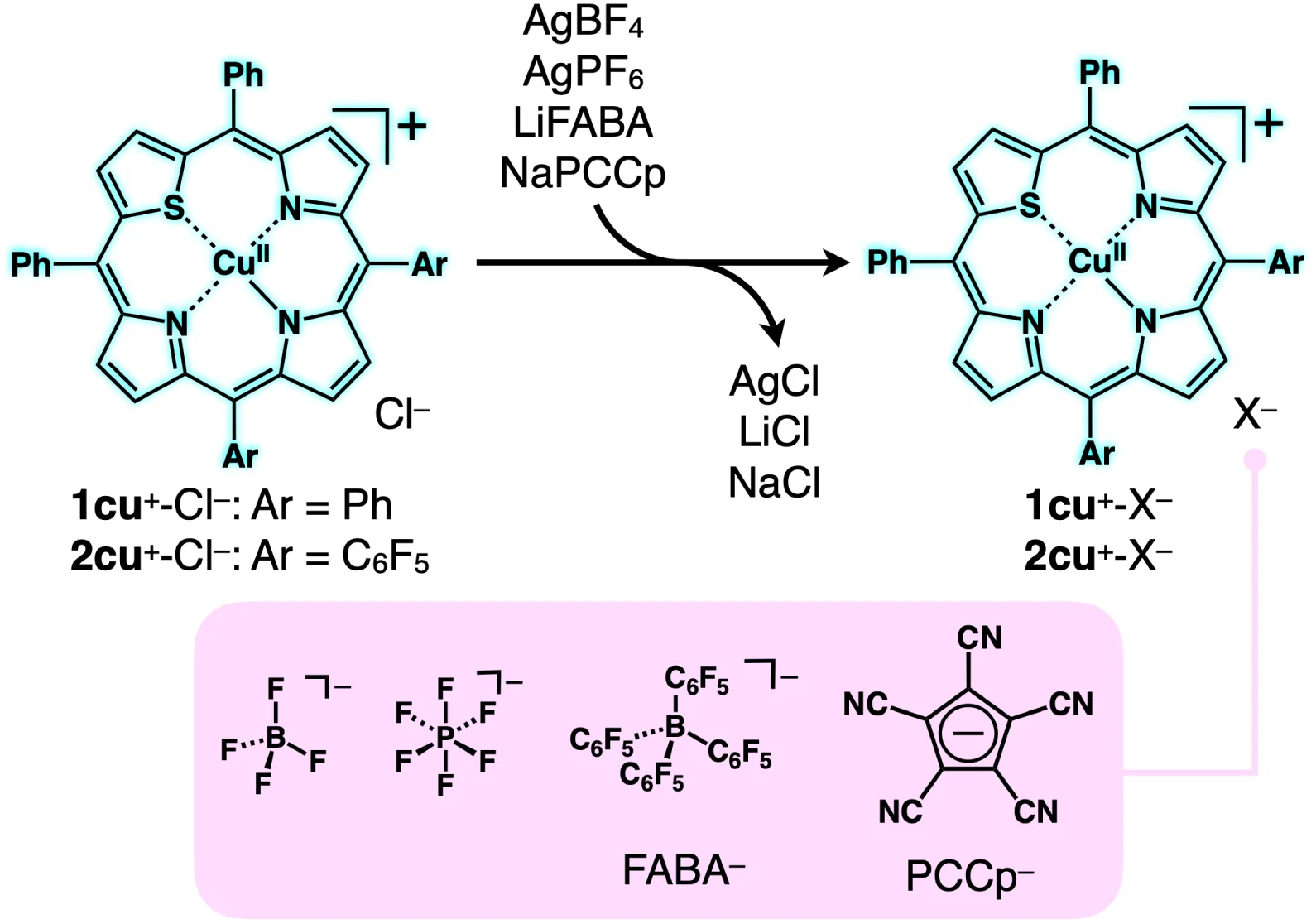
Ion-pair metathesis of Cu^II^ complexes of 1cu^+^ and 2cu^+^ from the Cl^−^ ion pairs.

Treatment of thiaporphyrins 1 and 2 with CuCl_2_ under reflux for 1 h in CH_3_CN afforded the corresponding Cu^II^ complexes 1cu^+^^7*b*^ and 2cu^+^ as Cl^−^ ion pairs (Fig. 2) in 90% and 86% yields, respectively. The Cl^−^ in 1cu^+^-Cl^−^ and 2cu^+^-Cl^−^ was exchanged with other anions, such as BF_4_^−^, PF_6_^−^, B(C_6_F_5_)_4_^−^ (FABA^−^) and pentacyanocyclopentadienide (PCCp^−^),^14^ by ion-pair metathesis using their metal salts, providing the corresponding ion pairs in 44–88% yields *via* silica gel column chromatography and recrystallization (Fig. 2).^15^ The broad ^1^H NMR signals in the obtained ion pairs revealed the paramagnetic Cu^II^ centres of 1cu^+^ and 2cu^+^ (Fig. S1–S8). Optimized structures of 1cu^+^ and 2cu^+^ at UB3LYP/6-31+G(d,p) with LanL2DZ for Cu^16^ showed distortion of the thiophene rings, with dihedral angles of 32.1° and 32.3°, respectively. The distortion induced mean-plane deviations of 0.27 and 0.26 Å for the core planes (25 atoms) and tetracoordinated geometry index (*τ*_4_)^17^ of 0.20 and 0.19, respectively (Fig. S48). 1cu^+^ and 2cu^+^, both of which have a sulfur atom, exhibit dipole moments of 2.26 and 12.67 D, respectively, due to the presence of a sulfur atom, the latter of which is larger owing to C_6_F_5_ substituents (Fig. S48). Furthermore, the theoretically estimated natural bond orbital (NBO) charges^18,19^ suggested a slightly higher Lewis acidity of the Cu^II^ centre in 2cu^+^ (1.18) than in 1cu^+^ (1.16) (Fig. S56).

**Fig. 3:**
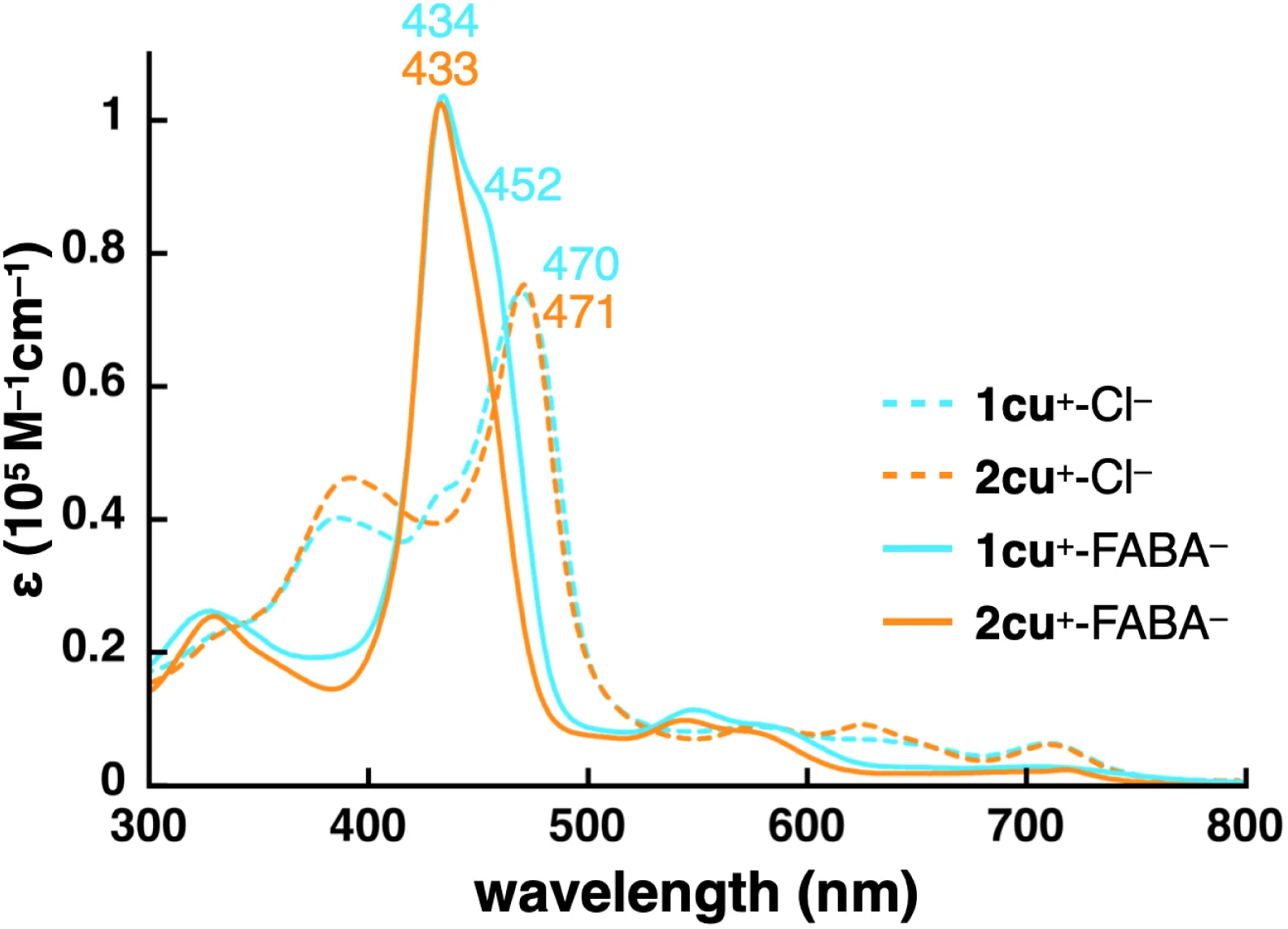
UV/vis absorption spectra of 1cu^+^-Cl^−^ (blue broken line), 2cu^+^-Cl^−^ (orange broken line), 1cu^+^-FABA^−^ (blue solid line) and 2cu^+^-FABA^−^ (orange solid line) in CH_2_Cl_2_ (0.01 mM).

The UV/vis absorption spectra of the ion pairs in CH_2_Cl_2_ (0.01 mM) also exhibited different features depending on the counteranions and peripheral substituents (Fig. 3 and S95). Similar to 1ni^+^,^8*a*^1cu^+^-Cl^−^ and 2cu^+^-Cl^−^ displayed Soret bands with maxima at 470 and 471 nm, respectively, whereas the other ion pairs of 1cu^+^ and 2cu^+^, except for 2cu^+^-PF_6_^−^,^20^ exhibited similar absorption spectra with peaks at ∼434/452 and 433 nm, respectively, suggesting that 1cu^+^ and 2cu^+^ formed no axially coordinated structures in the absence of Cl^−^. Differences in the Q bands were also observed in the presence and absence of axial coordination. Optimized structures of 1cu^+^-Cl^−^ and 2cu^+^-Cl^−^ at UB3LYP/6-31+G(d,p) with LanL2DZ for Cu^16^ showed thiophene–porphyrin dihedral angles of 29.7° and 29.5° and Cu⋯Cl distances of 2.32 and 2.30 Å, respectively (Fig. S49). Mean-plane deviations of the theoretically estimated core parts (25 atoms), *τ*_4_ and pentacoordinated geometry index (*τ*_5_)^21^ were 0.25/0.19 Å, 0.25/0.53 and 0.33/0.24 for 1cu^+^-Cl^−^ and 2cu^+^-Cl^−^, respectively.^22^ The observed spectra were correlated with the time-dependent (TD)-DFT-based theoretical spectra at UB3LYP/6-31+G(d,p) with LanL2DZ for Cu (Fig. S54 and S55). Theoretically estimated Soret bands of 1cu^+^-Cl^−^/2cu^+^-Cl^−^ and 1cu^+^/2cu^+^ were found at 460/460 and 438/438 nm, respectively. Observed absorption bands are assigned to various electronic transitions between molecular orbitals. In 1cu^+^-Cl^−^, the theoretically estimated 178β → 184β transition responsible for the absorption at 470 nm has charge-transfer character from the pyrrole ring and phenyl units to the core macrocycle (Fig. S52 and S55a), whereas in 2cu^+^-Cl^−^, the 222α → 224α and 221β → 224β transitions for the absorption at 471 nm correspond to the changes in the spin distribution over the core macrocycle (Fig. S53 and S55b). In 1cu^+^ in the absence of axial coordination, the 165β → 174β transition for the absorption at 434 nm exhibits the charge transfer from phenyl units close to a thiophene unit to the core macrocycle (Fig. S50 and S54a), whereas in 2cu^+^, the 212β → 215β transition for the absorption at 433 nm involves mainly redistribution of spin density within the macrocyclic core (Fig. S51 and S54b).

### Crystal-state ion-pairing assemblies with π-electronic anions

**Fig. 4:**
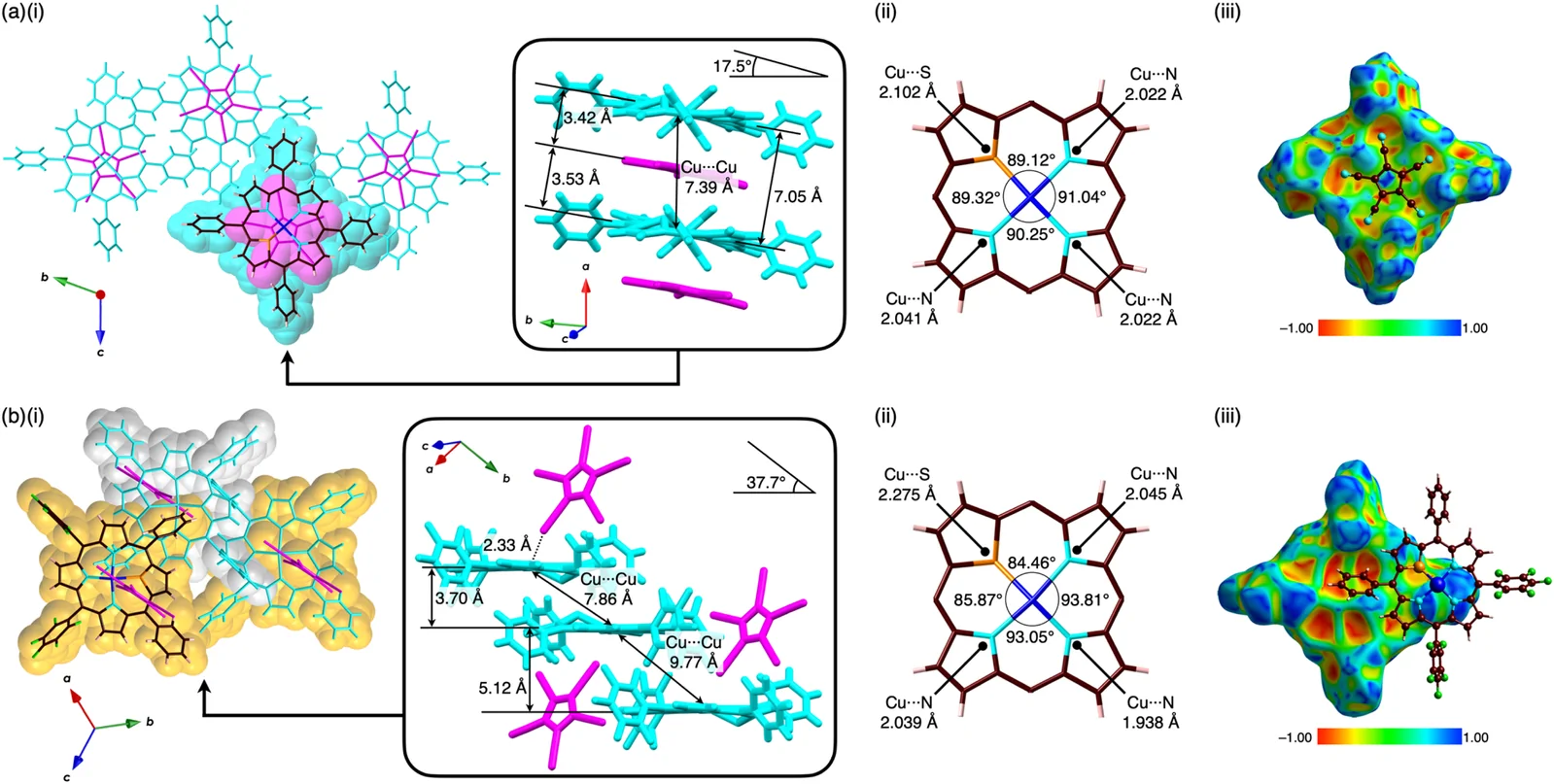
Single-crystal X-ray structures of (a) 1cu^+^-PCCp^−^ as a major disordered structure and (b) 2cu^+^-PCCp^−^: (i) packing diagrams and side views of the highlighted space-filling modeling indicated by the arrow, (ii) top view of monomeric cations with the aryl moieties omitted and (iii) Hirshfeld surfaces of the cations mapped over shape-index property with the ball-and-stick model of the neighbouring PCCp^−^ (a) and 2cu^+^ (b). Colour code in (i) (selected regions), (ii) and (iii): brown, cyan, yellow green, orange and blue refer to carbon, nitrogen, fluorine, sulfur and copper, respectively. Colour code in (i): cyan and magenta refer to cation and anion parts, respectively. In (b)(i), cations are stacked in the order of yellow, white and yellow space-filling models.

π-Electronic anions, such as PCCp^−^, promote *π-sip* formation with π-electronic cations and thus favour charge-by-charge assemblies. The crystal-state assemblies with PCCp^−^ were examined to clarify the influence of the anion geometries. Single crystals of 1cu^+^-PCCp^−^ and 2cu^+^-PCCp^−^ suitable for X-ray analysis were formed using CH_2_Cl_2_-1-butanol/*n*-hexane and CH_2_Cl_2_-1-propanol/*n*-hexane, respectively (Fig. 4, S12, S13 and S20–S23). 1cu^+^-PCCp^−^ formed a charge-by-charge assembly based on the *π-sips* with stacking distances of 3.42 and 3.53 Å, whereas the 1cu^+^ plane and Cu⋯Cu distances were 7.05 and 7.39 Å, respectively (Fig. 4a(i), S20 and S21). On the other hand, in 2cu^+^-PCCp^−^, the PCCp^−^-N demonstrated axial coordination with Cu^II^ of 2cu^+^ (N⋯Cu: 2.33 Å), despite the low coordination ability of PCCp^−^, suggesting high Lewis acidity of 2cu^+^ (Fig. 4b(i), S22 and S23). The interplanar distances of 2cu^+^ in 2cu^+^-PCCp^−^ were 3.70 and 5.12 Å, whereas the Cu⋯Cu distances were 7.86 and 9.77 Å, respectively. 1cu^+^ showed comparable Cu–N distances (2.04/2.02/2.02 Å) and a shorter Cu–S distance (2.10 Å) relative to 2cu^+^ (2.05/2.04/1.94 and 2.28 Å, respectively) (Fig. 4(ii) and S24). The observed PCCp^−^-N–Cu–N and PCCp^−^-N–Cu–S angles in the axially coordinated ion pair were 115.0°/94.7°/94.3° and 89.3°, respectively (Fig. S22).

In the crystal state, the thiophene–porphyrin dihedral angles of 1cu^+^-PCCp^−^ and 2cu^+^-PCCp^−^ (24.5° and 22.5°, respectively) (Fig. S20 and S22) were smaller than those of the optimized 1cu^+^ and 2cu^+^ (32.1° and 32.3°, respectively) (Fig. S48), suggesting that ion pairing, along with self-stacking for the latter, suppressed the out-of-plane distortion of the thiophene unit. The reduced distorted structures were also reflected in the coordination geometries around the Cu^II^ centre. In 1cu^+^-PCCp^−^, the *τ*_4_ value of 1cu^+^ (0.13) was smaller than that of the optimized 1cu^+^ (0.20), indicating that ion pairing in the crystal state drove the Cu^II^ centre toward a more square-planar geometry. In contrast, 2cu^+^ in 2cu^+^-PCCp^−^ exhibited a larger *τ*_4_ value (0.28) than 1cu^+^-PCCp^−^ (0.13), reflecting enhanced distortion around the Cu^II^ centre. The distorted structure was also observed in 2cu^+^-PCCp^−^, particularly demonstrated by the *τ*_5_ value of 0.32, which was larger than that for optimized 2cu^+^-Cl^−^ (0.24) (Fig. S39).^23^ Hirshfeld surface analysis^24,25^ of the *π-sip* in 1cu^+^-PCCp^−^ showed a bow-tie shape, indicating ^*i*^π–^*i*^π interactions (Fig. 4a(iii) and S29). In contrast, the shape-index surface of 2cu^+^-PCCp^−^ exhibited a distinct red region (Fig. 4b(iii) and S30), suggesting axial coordination between 2cu^+^-Cu and PCCp^−^-N. In noncovalent interaction (NCI) analysis^26–29^ for the *π-sip* of 1cu^+^-PCCp^−^, green isosurfaces were observed between the π-surfaces (Fig. S37 and S38), and quantum theory of atoms in molecules (QTAIM) topology analysis^30^ revealed multiple bond critical points (BCPs) between 1cu^+^ and PCCp^−^ (Fig. S44). These results suggested the presence of spatially widespread dispersion-dominated attractive interactions, consistent with ^*i*^π–^*i*^π interactions.

**Fig. 5:**
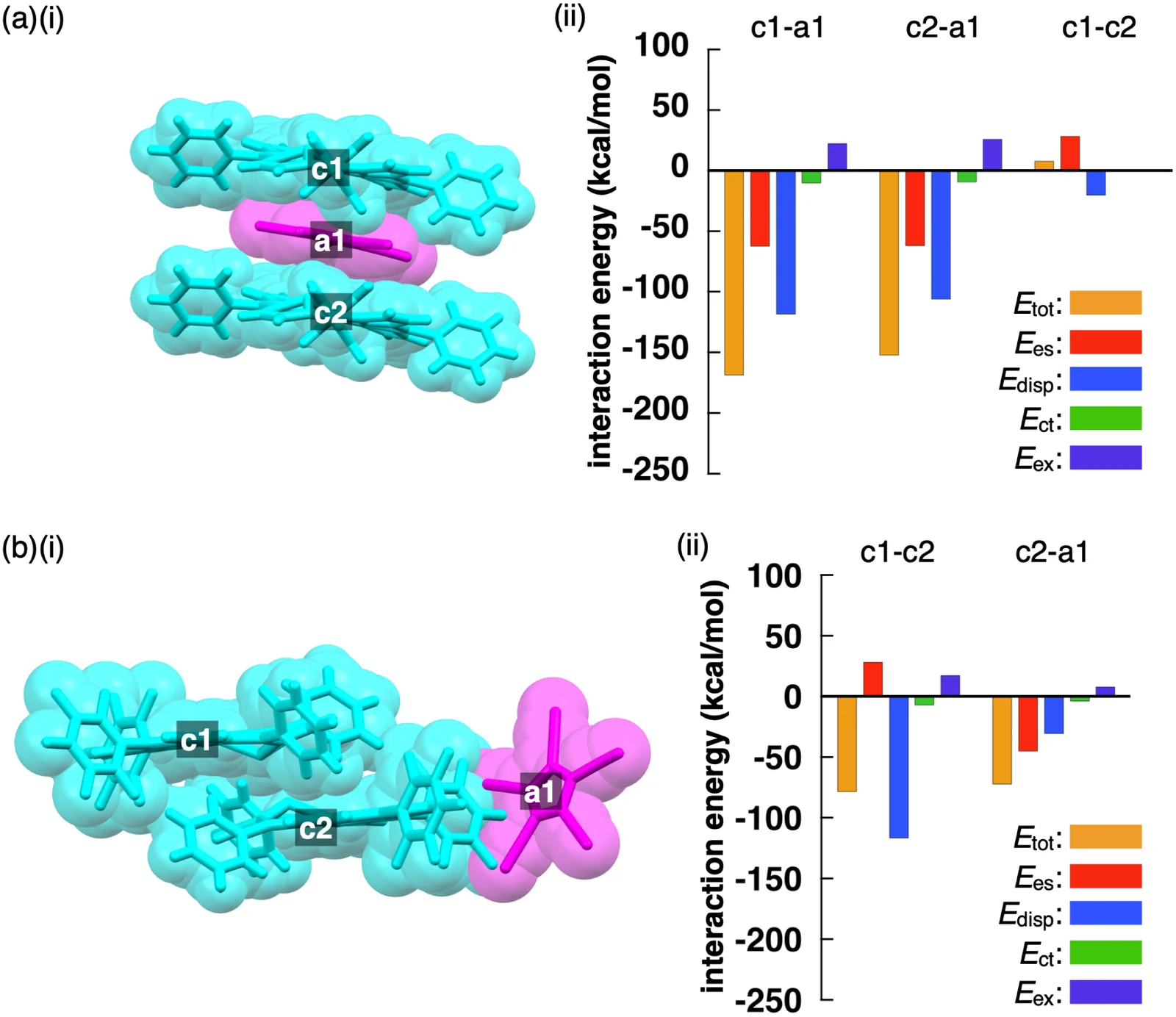
PIEDA of (a) 1cu^+^-PCCp^−^ and (b) 2cu^+^-PCCp^−^: (i) single-crystal X-ray structures and (ii) estimated interaction energies based on the FMO2-MP2 using mixed basis sets including NOSeC-V-DZP with MCP with TZP for Cu. Colour code in (i): cyan and magenta refer to cation and anion parts, respectively.

The interactions in the packing structures were theoretically elucidated using pair interaction energy decomposition analysis (PIEDA)^31^ in the framework of the fragment molecular orbital (FMO) method at the FMO2-MP2 using mixed basis sets including NOSeC-V-DZP with TZP for Cu with MCP (Fig. 5, S92, S93, Table S39 and S40).^32–34^ PIEDA calculations determine electrostatic (*E*_es_), dispersion (*E*_disp_), charge transfer (*E*_ct_) and exchange-repulsion (*E*_ex_) interaction energies and the total interaction energy (*E*_tot_). PIEDA in 1cu^+^-PCCp^−^ (c1–a1: *E*_tot_ = −165.55 kcal mol^−1^) suggested attractive ^*i*^π–^*i*^π interactions in the *π-sip* with synergetic electrostatic and dispersion forces (*E*_es_ = −59.70 and *E*_disp_ = −117.99 kcal mol^−1^) (Fig. 5a). In 2cu^+^-PCCp^−^, the interaction between neighbouring 2cu^+^ units (c1–c2) had a *E*_tot_ value of −78.65 kcal mol^−1^, which was dominated by a large contribution of *E*_disp_ (−116.71 kcal mol^−1^), whereas the electrostatic term was repulsive (*E*_es_ = 28.16 kcal mol^−1^) (Fig. 5b). The dipole moment of 2cu^+^ may contribute to the antiparallel orientation in the stacked cations. The cation–anion interaction (c2–a1) exhibited *E*_tot_ = −72.21 kcal mol^−1^, with the stabilization mainly arising from electrostatic attraction (*E*_es_ = −45.27 kcal mol^−1^) together with dispersion force (*E*_disp_ = −30.67 kcal mol^−1^). In addition, the short contact (2.33 Å) between 2cu^+^-Cu and N of another PCCp^−^ is correlated with the stabilization by electrostatic, polarization and charge transfer interactions for the axial coordination.^35^

### Crystal-state two-by-two assemblies of π-stacked cations with nonplanar counteranions

**Fig. 6:**
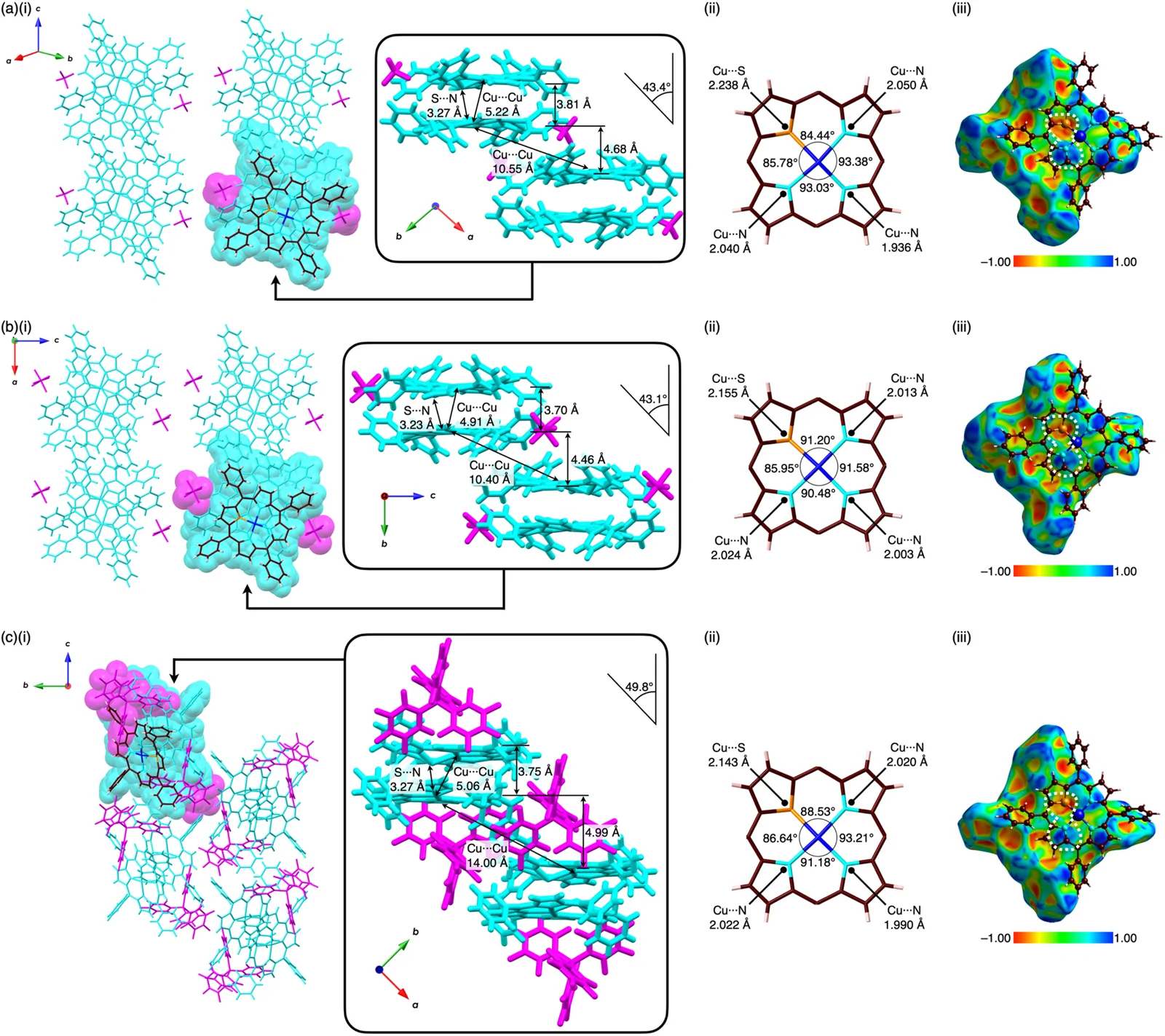
Single-crystal X-ray structures of (a) 1cu^+^-BF_4_^−^, (b) 1cu^+^-PF_6_^−^ and (c) 1cu^+^-FABA^−^: (i) packing diagrams and side views of the highlighted space-filling modeling indicated by the arrow, (ii) top view of monomeric cations with the aryl moieties omitted and (iii) Hirshfeld surfaces of cations mapped over shape-index property with the ball-and-stick model of the neighbouring 1cu^+^. In (b) and (c), major structures are indicated as representatives. Colour code in (i) (selected region), (ii) and (iii): brown, yellow, cyan, orange and blue refer to carbon, boron, nitrogen, sulfur and copper, respectively. Colour code in (i): cyan and magenta refer to cation and anion parts, respectively. In (iii), characteristic mapping patterns are indicated by dashed lines.

Nonplanar counteranions can interfere with the charge-by-charge mode and instead allow stacking of π-electronic cations by themselves. In fact, thiaporphyrin-based cations have been shown to form π-stacked dimers as fundamental motifs for assemblies.^8^ The ion pairs 1cu^+^-BF_4_^−^, 1cu^+^-PF_6_^−^ and 1cu^+^-FABA^−^ provided single crystals suitable for X-ray analysis *via* vapour diffusion of CH_2_Cl_2_-1-butanol/*n*-hexane or *n*-octane for 1cu^+^-PF_6_^−^ (Fig. 6, S9–S11 and S14–S19). These ion pairs formed stacked cation dimers, resulting in two-by-two assemblies (Fig. 6(i)). 1cu^+^-BF_4_^−^ exhibited no crystallographic disorder, whereas in 1cu^+^-PF_6_^−^ and 1cu^+^-FABA^−^, the stacked dimers exhibited S/N disorder in their crystal structures (Fig. S10, S11, S25, Table S4 and S5). The stacking distances in the dimers (as the major structures for 1cu^+^-PF_6_^−^ and 1cu^+^-FABA^−^ here and following parts) were 3.81, 3.70 and 3.75 Å, respectively, ascribable to intercationic ^*i*^π–^*i*^π interactions (Fig. 6(i), S15, S17 and S19). The Cu⋯Cu distances in the dimers were 5.22, 4.91 and 5.06 Å, respectively, suggesting the close stacking between 1cu^+^ units. The arrangement of the stacked dimers differed significantly among these ion pairs. In 1cu^+^-BF_4_^−^ and 1cu^+^-PF_6_^−^, the dimers were slip-stacked with interplanar and Cu⋯Cu distances and angles along the stacking axis of 4.68/4.46 and 10.55/10.40 Å and 43.3°/43.1°, respectively (Fig. 6a(i) and b(i)). The close two-by-two mode was likely facilitated by the relatively small BF_4_^−^ and PF_6_^−^ with C(–H)⋯F distances of 3.17 and 3.25 Å, respectively (Fig. S14 and S16). On the other hand, in 1cu^+^-FABA^−^, the stacked dimers were laterally offset and spatially separated, with interplanar and Cu⋯Cu distances and an angle along the stacking axis of 4.99 and 14.00 Å and 49.8°, respectively (Fig. 6c(i)), with a C(–H)⋯F distance of 3.14 Å (Fig. S18). Bulky FABA^−^ anions modulate the arrangement, as observed in the spatially isolated stacked dimers.

In 1cu^+^-BF_4_^−^, 1cu^+^-PF_6_^−^ and 1cu^+^-FABA^−^, 1cu^+^ units formed stacked dimers with proximal convex sides, and the cations exhibited distorted thiophene rings with dihedral angles of 28.6°, 26.6° and 25.0°, respectively, for the major structures of the latter two ion pairs (Fig. S14, S16 and S18). The multiple S/N disorder patterns in 1cu^+^-PF_6_^−^ and 1cu^+^-FABA^−^ with different occupancies induced statistical populations of dimeric arrangements. In some combinations, 1cu^+^ formed dimers with antiparallel dipole orientations, reducing electrostatic repulsion as seen in dipole–dipole interactions.^8,10^ The interplanar S⋯N distances were 3.27, 3.23 and 3.27 Å in 1cu^+^-BF_4_^−^, 1cu^+^-PF_6_^−^ and 1cu^+^-FABA^−^, respectively, suggesting the formation of chalcogen bonding (Fig. 6i, S15, S17 and S19).^9^

1cu^+^ in 1cu^+^-BF_4_^−^, 1cu^+^-PF_6_^−^ and 1cu^+^-FABA^−^ exhibited Cu–N and Cu–S distances of 2.05/2.04/1.94 and 2.24 Å, 2.02/2.01/2.00 and 2.16 Å and 2.02/2.02/1.99 and 2.14 Å, respectively (Fig. 6(ii) and S24). The crystal-state thiophene–porphyrin dihedral angles were 28.6°, 26.6° and 25.0°, respectively (Fig. S14, S16 and S18), which were smaller than those of the optimized 1cu^+^ (32.1°) (Fig. S48). The *τ*_4_ values of 1cu^+^-BF_4_^−^ (0.26), 1cu^+^-PF_6_^−^ (0.15) and 1cu^+^-FABA^−^ (0.18) were comparable to that of optimized 1cu^+^ (0.20).^23^ These geometrical parameters were modulated by the crystal packing and coexisting counteranions.^36^

**Fig. 7:**
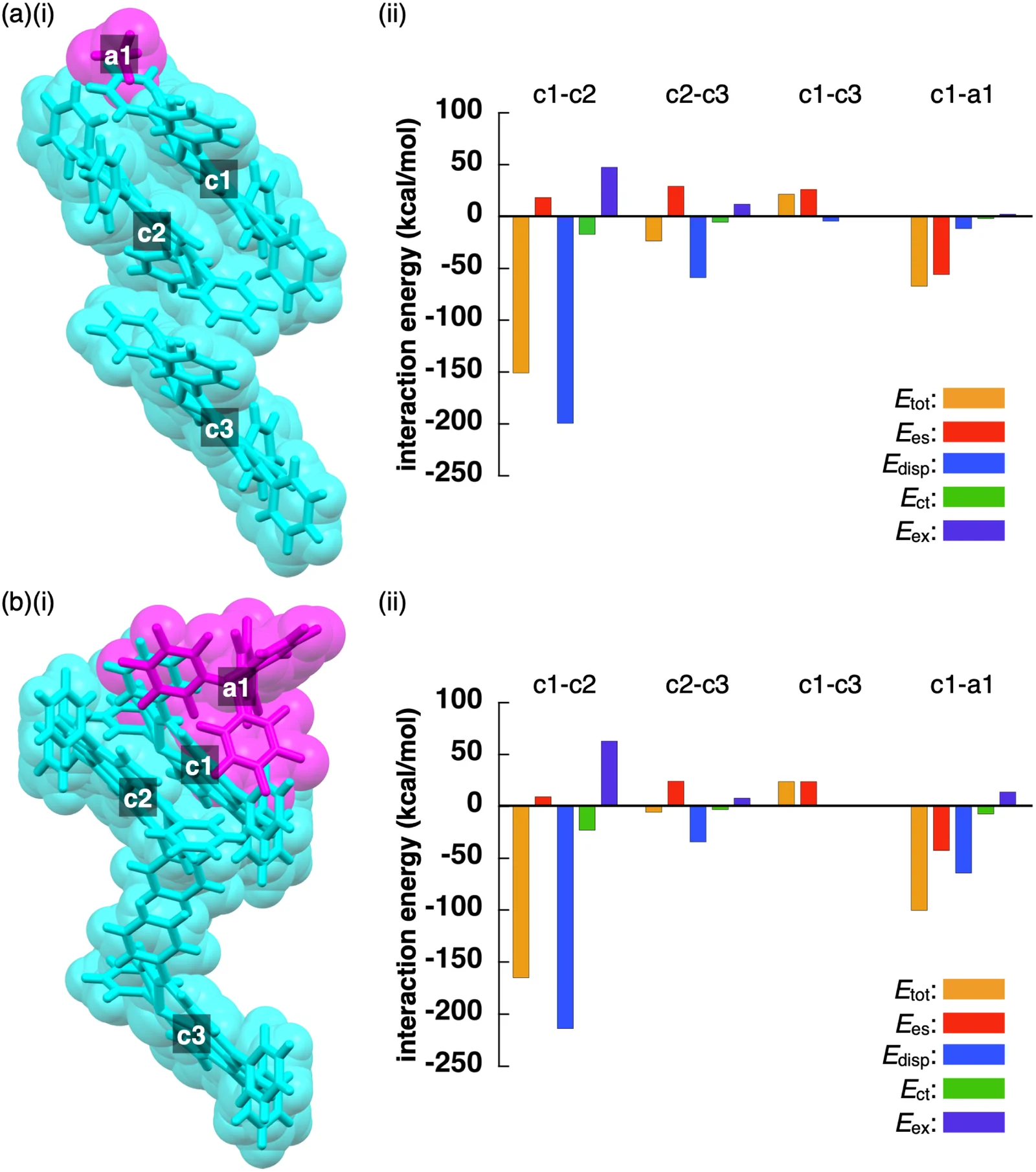
PIEDA of (a) 1cu^+^-BF_4_^−^ and (b) 1cu^+^-FABA^−^: (i) single-crystal X-ray structures and (ii) estimated interaction energies based on the FMO2-MP2 using mixed basis sets including NOSeC-V-DZP with MCP with TZP for Cu. Colour code in (i): cyan and magenta refer to cation and anion parts, respectively.

Hirshfeld surface analysis^24,25^ of 1cu^+^-BF_4_^−^, 1cu^+^-PF_6_^−^ and 1cu^+^-FABA^−^ showed large blue and red patterns localized on the sulfur and nitrogen atoms, respectively, which were ascribed to chalcogen bonding (Fig. 6(iii) and S26–S28). The flat regions on the curvedness surface suggest ^*i*^π–^*i*^π interactions in dimeric 1cu^+^. NCI analysis^26–29^ for these ion pairs displayed green isosurfaces between the 1cu^+^ units in the stacked dimers, consistent with intercationic ^*i*^π–^*i*^π interactions (Fig. S31–S36). In addition, green isosurfaces appeared between the sulfur and nitrogen atoms due to the chalcogen bonding.^9^ BCPs derived from S⋯π (pyrrole ring) interactions between the stacked dimers were also identified by QTAIM topology analysis (Fig. S41–S43).^30^ PIEDA revealed that the main contributions to the interionic interactions for dimeric 1cu^+^ (c1–c2: *E*_tot_ = −150.90 kcal mol^−1^) in 1cu^+^-BF_4_^−^ were *E*_disp_ and *E*_es_ with values of −199.34 and 18.24 kcal mol^−1^, respectively, suggesting ^*i*^π–^*i*^π interactions between 1cu^+^ units (Fig. 7a, S89 and Table S36). Considerable exchange-repulsion forces were observed in the tightly stacked 1cu^+^ dimer (47.49 kcal mol^−1^). A similar tendency was observed for the cation dimers, c1–c2, in 1cu^+^-PF_6_^−^ (c1–c2: *E*_tot_ = −164.75 kcal mol^−1^, *E*_disp_ = −216.90 kcal mol^−1^ and *E*_es_ = −5.86 kcal mol^−1^, Fig. S90 and Table S37) and 1cu^+^-FABA^−^ (c1–c2: *E*_tot_ = −165.10 kcal mol^−1^, *E*_disp_ = −213.92 kcal mol^−1^ and *E*_es_ = −9.05 kcal mol^−1^, Fig. 7b, S91 and Table S38). In addition, the *E*_es_ values of c1–c2 in 1cu^+^-BF_4_^−^, 1cu^+^-PF_6_^−^ and 1cu^+^-FABA^−^ were smaller than those of c2–c3 (29.19, 22.59 and 24.21 kcal mol^−1^, respectively), suggesting dipole–dipole interactions in the stacked dimers.

**Fig. 8:**
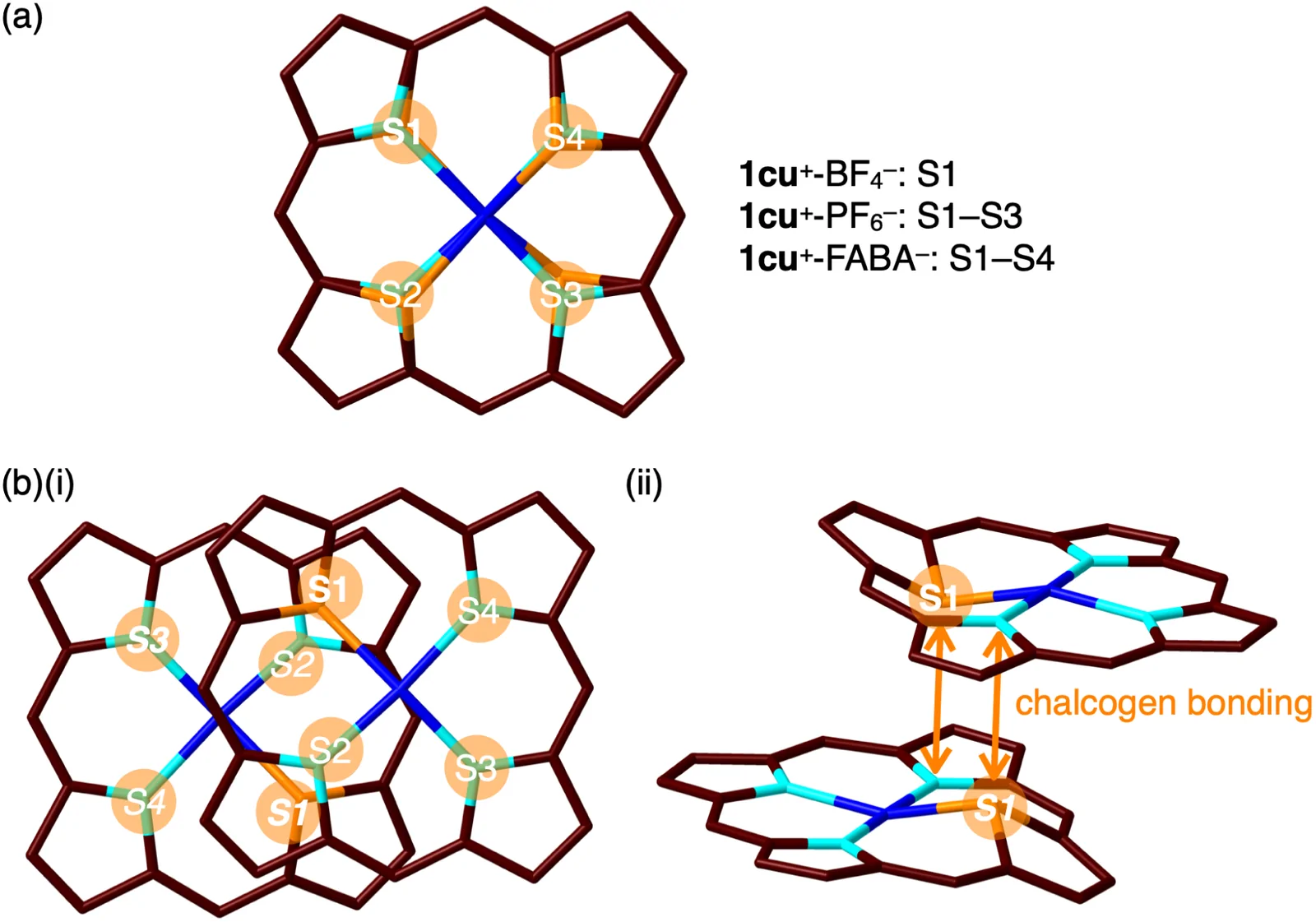
Disorder diagrams of 1cu^+^ (core 25 atoms) of 1cu^+^-FABA^−^ as a representative: (a) top view of the monomer and (b)(i) top and (ii) side views of the stacked dimer showing the N_3_S site disorder labelled as S1–S4. The bold S1 positions denote the major S-donor sites, and the roman and italic labels indicate the upper and lower cations, respectively. In (b), the S1–S1 combination is shown as representative. Atom colour code: brown, cyan, orange and blue refer to carbon, nitrogen, sulfur and copper, respectively.

Related to the statistical populations of dimeric arrangements, the number of chalcogen bonds in the cation dimers depended on relative monomer orientations (Fig. 8, S25, Table S4 and S5). For example, the combinations retaining appropriate inward-oriented sites for 1cu^+^-FABA^−^ allowed chalcogen bonding, as observed in the S1–S1 combination with two chalcogen bonds and S1–S3 and S1–S4 with a single chalcogen bond. In contrast, the S3–S3 combination formed no chalcogen bonding because the two S atoms were directed away from the dimer overlap region. Similarly, no chalcogen bonding was observed in the combinations of S1–S2, S2–S3, S2–S4, S3–S4, S2–S2 and S4–S4. Notably, the combinations including S2 induced no chalcogen bonding because of their locations on the opposite sides from the neighbouring cations. 1cu^+^-PF_6_^−^, which lacks the corresponding S4 site, also exhibited similar disorder combinations.

### Arrangement-dependent spin–spin interactions

**Fig. 9:**
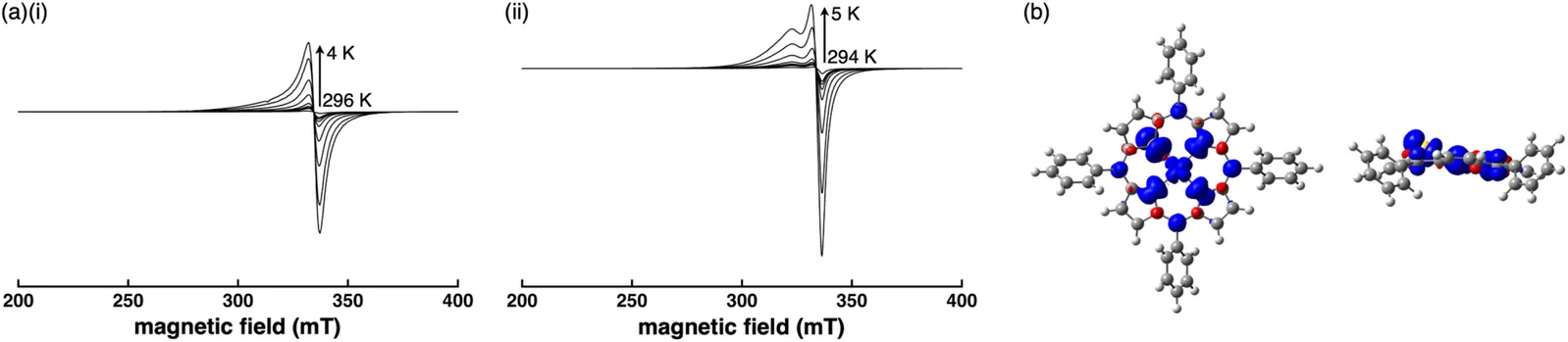
(a) Solid-state ESR spectra of (i) 1cu^+^-PCCp^−^ and (ii) 1cu^+^-BF_4_^−^ and (b) theoretically estimated spin density mapping (*δ* = 0.001) of the optimized structure of 1cu^+^ as top and side views calculated at UB3LYP/Def2TZVP. The unpaired spin is predominantly localized on the Cu centre with significant polarization on the coordinating N and S atoms. These plots define the intrinsic spin distribution of the cations for discussion of solid-state magnetic properties.

The magnetic properties of the obtained ion pairs were examined by comparing the charge-by-charge and two-by-two assembly modes. Magnetic properties are investigated using larger amounts of solid samples prepared under similar conditions to those used to prepare single crystals, often resulting in polymorphs production. Therefore, the diffraction patterns of the solid samples for the magnetic properties were compared with the simulated patterns of the corresponding single crystals (Fig. S46 and S47).^37^ In fact, the partial agreement for 1cu^+^-PCCp^−^, 1cu^+^-BF_4_^−^, 1cu^+^-PF_6_^−^ and 1cu^+^-FABA^−^ prompted us to focus on these ion pairs for further detailed examinations of the magnetic properties. Solid-state electron spin resonance (ESR) spectra of these ion pairs provided similar *g̃* values (2.082, 2.095, 2.124 and 2.070, respectively) (Fig. 9a and S97), suggesting that the electron spin was predominantly in the Cu^II^ centre and the spin distributions in the square-planar CuN_3_S core were not significantly dependent on the counteranions. 1cu^+^-PCCp^−^ and 1cu^+^-PF_6_^−^ exhibited rhombic *g* value patterns with three different values (*g*_*x*_ ≠ *g*_*y*_ ≠ *g*_*z*_), suggesting reduced local symmetry around the CuN_3_S core (Fig. 9a(i) and S97b). In contrast, 1cu^+^-BF_4_^−^ and 1cu^+^-FABA^−^ were approximately axis-symmetric, allowing the definition of *g*_⊥_ (*g*_*x*_ = *g*_*y*_) and *g*_∥_ (*g*_*z*_) (Fig. 9a(ii) and S97c).^38^ DFT-based spin density calculations at UB3LYP/Def2TZVP of the optimized structure of 1cu^+^ exhibited predominantly Cu-localized spin distributions (Fig. 9b, S57 and Table S8). The electron spin was primarily localized on the Cu d_*x*^2^–*y*^2^_ orbital (0.473) and coordinating N (0.119, 0.119 and 0.126) and S (0.113) atoms, resulting in a total spin population of 0.979 in the CuN_3_S unit.^38*a*^

**Fig. 10:**
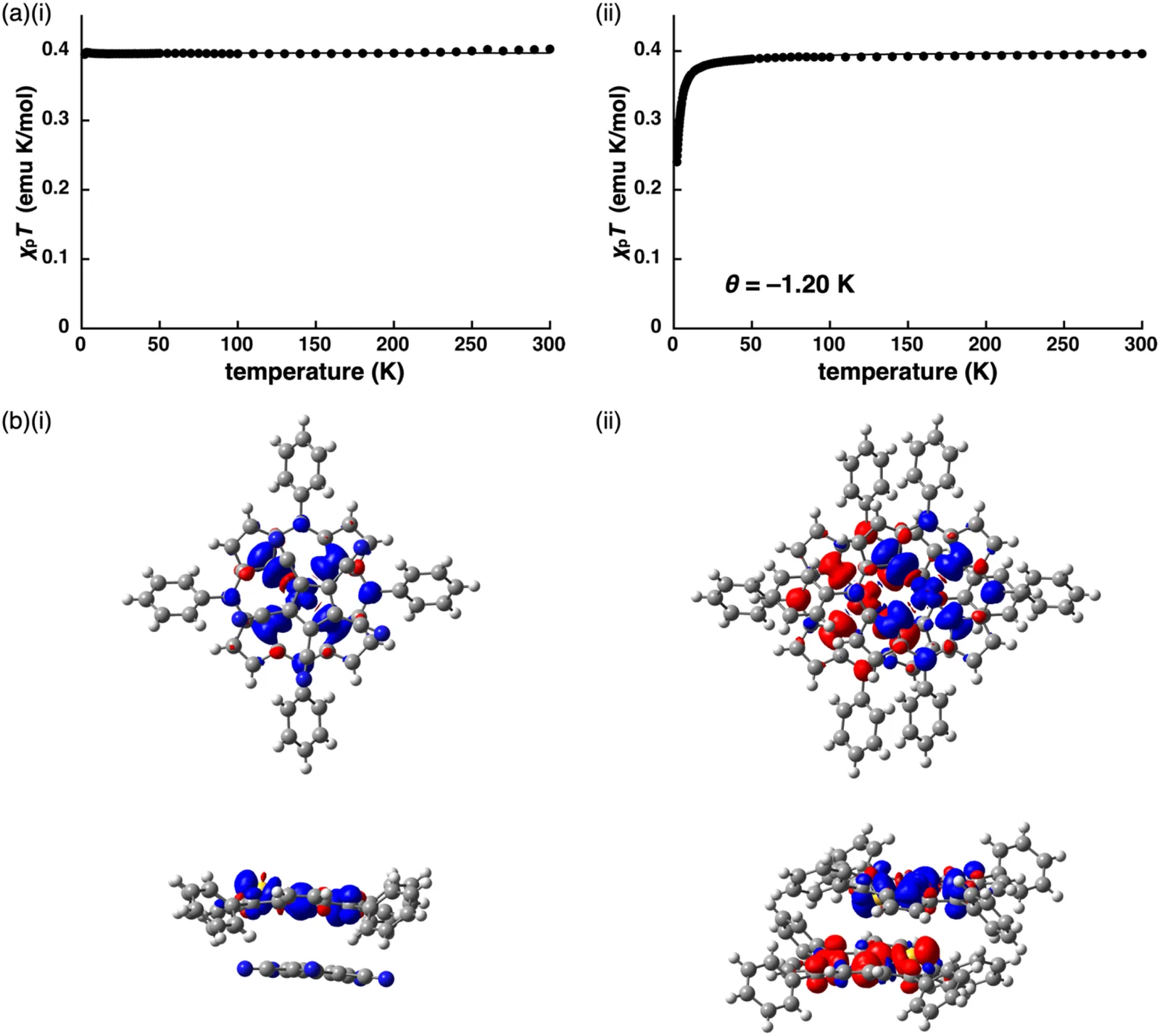
(a) Temperature dependence of the molar magnetic susceptibility (*χ*_p_*T*) with solid lines representing Curie–Weiss fits with Curie–Weiss temperatures *θ* (K) of (i) 1cu^+^-PCCp^−^ and (ii) 1cu^+^-BF_4_^−^ and (b) theoretically estimated spin density mappings (*δ* = 0.001) of single crystal structures of (i) 1cu^+^-PCCp^−^ and (ii) 1cu^+^ dimer of 1cu^+^-BF_4_^−^ as top and side views calculated at GD3BJ-UB3LYP/Def2TZVP.

Magnetic interactions depended on the assembly mode (Fig. 10a and S98). No detectable Curie–Weiss temperature (*θ*) was obtained for 1cu^+^-PCCp^−^, likely due to the electron spin in 1cu^+^ isolated by PCCp^−^ in charge-by-charge assembly. In contrast, the Curie–Weiss fitting yielded Curie–Weiss temperatures of −1.20 K for 1cu^+^-BF_4_^−^, −0.76 K for 1cu^+^-PF_6_^−^ and −0.20 K for 1cu^+^-FABA^−^, suggesting antiferromagnetic interactions *via* the close arrangement of the CuN_3_S units in the stacked cation dimers. Notably, only S⋯N contacts were shorter than the sum of the van der Waals radii around the CuN_3_S cores, suggesting that the S⋯N contact was the most likely superexchange pathway. Differences in the antiferromagnetic interactions between 1cu^+^-BF_4_^−^, 1cu^+^-PF_6_^−^ and 1cu^+^-FABA^−^ were correlated with the arrangement of the CuN_3_S units. In 1cu^+^-BF_4_^−^, the stacked dimer adopted the S1–S1 arrangement with a high proportion of short S⋯N contacts in the solid state (Fig. 8), which was correlated with the higher *θ* of −1.20 K. In contrast, 1cu^+^-PF_6_^−^ and 1cu^+^-FABA^−^ showed a smaller contribution from S⋯N contacts due to the S/N disorder, consistent with the weaker antiferromagnetic interaction than that of 1cu^+^-BF_4_^−^. In addition to the local S⋯N contacts, differences in the interdimer distances in the two-by-two assemblies (Fig. 6(i)) and chemical pressure^39^ may provide an additional structural factor affecting the observed magnetic behaviours.

The spin density calculations for the single-crystal structures of the ion pairs theoretically supported the experimental results (Fig. 10b, S57–S84 and Table S8–S35). In the *π-sip*-based charge-by-charge assembly of 1cu^+^-PCCp^−^, the spin density remained localized on the CuN_3_S core with negligible delocalization onto PCCp^−^, consistent with no spin–spin interactions (Fig. 10b(i), S84 and Table S35). In contrast, the crystal-state stacked cation dimers of 1cu^+^-BF_4_^−^ and 1cu^+^-FABA^−^ exhibited the localization of α and β spins at the respective stacked cations (Fig. 10b(ii), S57–S83 and Table S8–S34), suggesting antiferromagnetic interactions.^40^ Each CuN_3_S core retained a largely localized spin density (*ca.* 0.9). The experimental and theoretical results indicated that the magnetic properties of the ion-pairing assemblies were modulated by the anion-dependent arrangement of the paramagnetic thiaporphyrin Cu^II^ complex cations.^41^

## Conclusions

In this study, ion pairs of thiaporphyrin Cu^II^ complexes with various counteranions were prepared to modulate their magnetic properties. The PCCp^−^ ion pairs formed a charge-by-charge assembly based on *π-sips* and an assembly based on pentacoordinated complexes *via* axial Cu–N coordination with PCCp^−^. In contrast, ion pairs with nonplanar counteranions showed π-stacked dimers that formed two-by-two assemblies. ESR and magnetic susceptibility measurements of the solid materials, supported by theoretical calculations, suggest that the magnetic behaviour is dependent on the assembly modes of the paramagnetic cations. In contrast to the charge-by-charge assembly of the PCCp^−^ ion pair, the ion pairs with nonplanar anions exhibited antiferromagnetic behaviours owing to their dimer-based packing structures. Theoretical calculations suggested that the spin density was primarily localized within the CuN_3_S core of the cations, supporting the crucial factors in the distance and orientation of the CuN_3_S units in the assemblies for spin–spin interactions. This study demonstrates the modulation of assembly modes and the resulting magnetic properties *via* the dimerization of macrocycle Cu^II^ complexes using chalcogen-bonding and dipole–dipole interactions.^12,42^ Counteranions can control the dimerization, thereby modulating the building blocks for tuning the spin arrangements. The design of π-electronic systems with charge and spin would provide fascinating strategies for the construction of supramolecular spintronic materials.

## Author contributions

H. M. designed and conducted the project. M. F. carried out the synthesis, characterization, and physical property measurements. H. H. carried out the physical property measurements and contributed to the discussion and the preparation of the manuscript. K. F. measured the solid-state ESR and magnetic susceptibility. K. S. performed Le Bail fitting. K. O. and Y. S. conducted the NEDA and spin density calculations. Y. I. measured solid-state UV/vis absorption.

## Conflicts of interest

There are no conflicts of interest to declare.

## Supplementary Material

SC-017-D6SC04368B-s001

SC-017-D6SC04368B-s002

## Data Availability

CCDC 2537959, 2537960, 2537961, 2537962 and 2537963 contain the supplementary crystallographic data for this paper.^43*a*–*e*^ Data supporting the work in this publication are available *via* the supplementary information (SI) and associated crystallo-graphic data. Supplementary information: synthetic procedures, analytical data, computational details and CIF les for the single-crystal X-ray structural analysis. See DOI: https://doi.org/10.1039/d6sc04368b.
