# Supplementary material for: Ion-pairing-modulated magnetic properties of thiaporphyrin CuII complex cations
Source: Chem Sci. 2026 Aug 24;17(36):17269–78. doi: 10.1039/d6sc04368b (PMC13539314; doi:10.1039/d6sc04368b)
Supplement: Supplementary file 1 [file SC-017-D6SC04368B-s001.pdf]

Supplementary Information (SI) for Chemical Science.
This journal is © The Royal Society of Chemistry 2026

Electronic Supplementary Information

Ion-pairing-modulated magnetic properties of thiaporphyrin Cu^{II} complex cations

Masaki Fujita, Hiroki Horita, Ko Furukawa, Kunihisa Sugimoto, Kazuki Okazawa, Yasuteru Shigeta, Yukihide Ishibashi and Hiromitsu Maeda*

Department of Applied Chemistry, College of Life Sciences, Ritsumeikan University, Kusatsu 525–8577, Japan, Fax: +81 77 561 3729; Tel: +81 77 561 5969; E-mail: maedahir@ph.ritsumei.ac.jp, Center for Instrumental Analysis, Institute for Research Promotion, Niigata University, Niigata 950–2181, Japan, Department of Chemistry, Faculty of Science and Engineering, Kindai University, Higashiosaka 577–8502, Japan, Institute of Pure and Applied Sciences, the University of Tsukuba, Tsukuba 305–8577, Japan, Center for Computational Sciences, The University of Tsukuba, Tsukuba 305–8577, Japan, and Department of Applied Chemistry, Graduate School of Science and Engineering, Ehime University, Matsuyama 790–8577, Japan

Table of Contents

1. Synthetic procedures and spectroscopic data S2
Fig. S1–8 ^{1}H NMR and ^{19}F NMR. S5
2. X-ray crystallographic data S12
Fig. S9–13 Ortep drawings. S13
Fig. S14–25 Packing diagrams. S17
Fig. S26–30 Hirshfeld surfaces. S26
Fig. S31–40 Noncovalent interaction (NCI) analysis. S31
Fig. S41–45 Quantum theory of atoms in molecules (QTAIM) topology analysis S36
Fig. S46,47 XRD patterns of the crystalline powders and calculated patterns of single crystals. S38
3. Theoretical studies S41
Fig. S48,49 Optimized structures. S41
Fig. S50–55 Molecular orbitals and theoretical UV/vis absorption spectra. S42
Fig. S56 NBO analysis. S47
Fig. S57–84 Spin density. S49
Fig. S85,86 NICS values and ACID plots. S94
Fig. S87,88 ESP mapping. S95
Fig. S89–94 Single-crystal X-ray structures for EDA calculations. S97
Cartesian coordination of optimized structures S101
4. Solution-state and solid-state properties S105
Fig. S95 UV/vis absorption spectra. S105
Fig. S96 Cyclic voltammograms. S106
Fig. S97 ESR spectra. S107
Fig. S98,99 Magnetic measurements. S108
Fig. S100 Solid-state absorption spectrum. S109

1. Synthetic procedures and spectroscopic data

General procedures. Starting materials were purchased from FUJIFILM Wako Pure Chemical Industries Ltd., Nacalai Tesque Inc., Tokyo Chemical Industry Co., Ltd., and Sigma-Aldrich Co., and used without further purification unless otherwise stated. 5,10,15,20-Tetraphenyl-21-thiaporphyrin[S1] **1**, Cu^{II} complex **1cu**$^+$ as a Cl^- ion pair[S2] (**1cu**$^+$-Cl^-), and 10,15-bis(pentafluorophenyl)-5,20-diphenyl-21-thiaporphyrin[S3] **2** were prepared according to the literature procedures. NMR spectra used in the characterization of products were recorded on a JEOL ECA-600 600 MHz spectrometer. All NMR spectra were referenced to solvent. UV-visible spectra were recorded on a Hitachi U-3500 spectrometer. High-resolution (HR) electrospray ionization mass spectrometry (ESI-MS) was recorded on a BRUKER microTOF using ESI-TOF method. TLC analyses were carried out on aluminum sheets coated with silica gel 60 (Merck 5554). Column chromatography was performed on Sumitomo alumina KCG-1525 and Wakogel C-300.

Cu^{II} complex of 5,10,15,20-tetraphenyl-21-thiaporphyrin as a BF_4^- ion pair, 1cu$^+$-BF_4^-. To a MeOH solution (70 mL) of **1cu**$^+$-Cl^- (48.0 mg, 65.8 µmol) was added $AgBF_4$ (41.7 mg, 0.214 mmol), and the reaction mixture was stirred at r.t. for 40 min, followed by filtration and evaporation to dryness. The residue was purified by silica gel column chromatography (Wakogel C-300; eluent: 25% MeOH/$CHCl_3$) and recrystallized from CH_2Cl_2/*n*-hexane to afford **1cu**$^+$-BF_4^- (44.1 mg, 56.5 µmol, 86%) as a green solid. R_f = 0.15 (5% MeOH/CH_2Cl_2). The signals in ^{1}H NMR (600 MHz, $CDCl_3$, 20 °C), observed at 9.16, 8.33, 7.09, and 6.70 ppm, were too broad to discuss in detail due to the paramagnetic Cu^{II}, whereas the signals in $^{13}C\{^1H\}$ NMR (151 MHz, $CDCl_3$, 20 °C) and ^{19}F NMR (564 MHz, $CDCl_3$, 20 °C) were not detected. UV/vis (CH_2Cl_2, λ_{max}[nm] (ε, 10^5 $M^{-1}cm^{-1}$)): 328 (0.28), 434 (0.97), 548 (0.12), 708 (0.042). HRMS (ESI-TOF) *m/z*: [M – BF_4]$^+$ Calcd for $C_{44}H_{28}CuN_3S^+$ 693.1294; Found 693.1294. This compound was further characterized by single-crystal X-ray analysis.

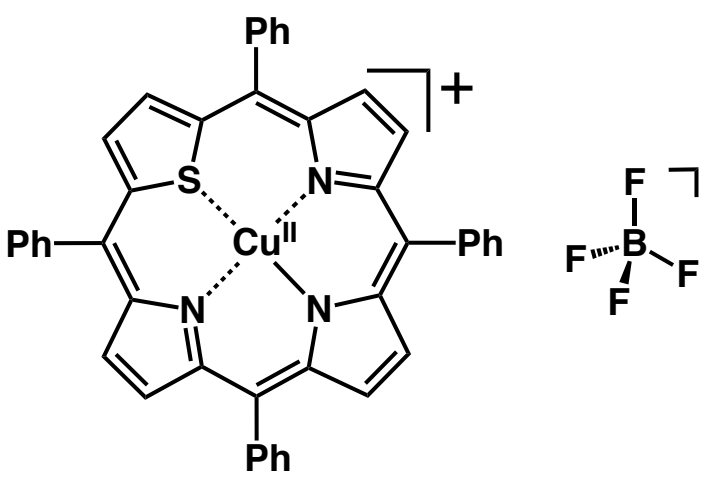

Cu^{II} complex of 5,10,15,20-tetraphenyl-21-thiaporphyrin as a PF_6^- ion pair, 1cu$^+$-PF_6^-. To a MeOH solution (70 mL) of **1cu**$^+$-Cl^- (47.6 mg, 65.2 µmol) was added $AgPF_6$ (52.0 mg, 0.206 mmol) and the reaction mixture was stirred at r.t. for 40 min, followed by filtration and evaporation to dryness. The residue was purified by silica gel column chromatography (Wakogel C-300; eluent: 25% MeOH/$CHCl_3$) and recrystallized from $CHCl_3$/*n*-hexane to afford **1cu**$^+$-PF_6^- (48.5 mg, 57.8 µmol, 71%) as a green solid. R_f = 0.24 (5% MeOH/CH_2Cl_2). The signals in ^{1}H NMR (600 MHz, $CDCl_3$, 20 °C), observed at 9.36, 8.52, 6.91, and 6.53 ppm, were too broad to discuss in detail due to the paramagnetic Cu^{II}, whereas the signals in $^{13}C\{^1H\}$ NMR (151 MHz, $CDCl_3$, 20 °C) and ^{19}F NMR (564 MHz, $CDCl_3$, 20 °C) were not detected. UV/vis (CH_2Cl_2, λ_{max}[nm] (ε, 10^5 $M^{-1}cm^{-1}$)): 327 (0.26), 434 (1.0), 549 (0.11), 704 (0.025). HRMS (ESI-TOF) *m/z*: [M – F_6P]$^+$ Calcd for $C_{44}H_{28}CuN_3S^+$ 693.1294; Found 693.1294. [M – $C_{44}H_{28}CuN_3S$]$^-$ Calcd for F_6P^- 144.9647; Found 144.9647.

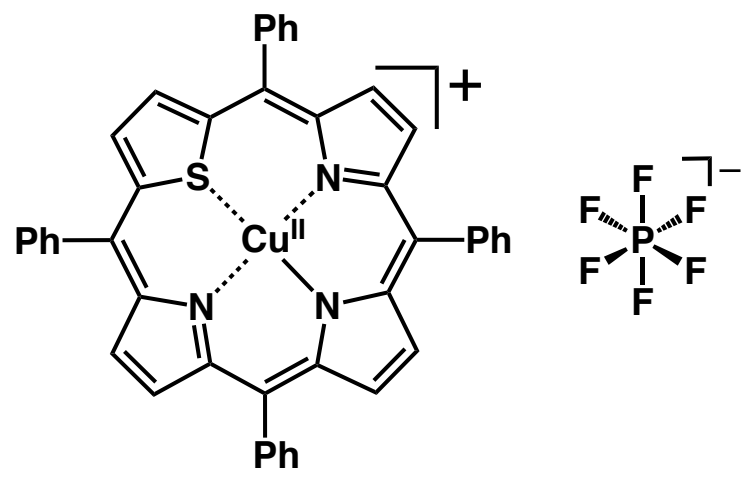

Cu^{II} complex of 5,10,15,20-tetraphenyl-21-thiaporphyrin as a $B(C_6F_5)_4^-$ ion pair, 1cu$^+$-$B(C_6F_5)_4^-$. To a MeOH solution (70 mL) of **1cu**$^+$-Cl^- (51.2 mg, 62.2 µmol) was added lithium tetrakis(pentafluorophenyl)borate ($LiB(C_6F_5)_4$) (51.2 mg, 74.6 µmol) and the reaction mixture was stirred at r.t. for 40 min, followed by filtration and evaporation to dryness. The residue was purified by silica gel column chromatography (Wakogel C-300; eluent: 25% MeOH/$CHCl_3$) and recrystallized from $CHCl_3$/*n*-hexane to afford **1cu**$^+$-$B(C_6F_5)_4^-$ (75.3 mg, 54.8 µmol, 88%) as a green solid. R_f = 0.51 (5% MeOH/CH_2Cl_2). The signals in ^{1}H NMR (600 MHz, $CDCl_3$, 20 °C), observed at 9.60, 8.72, 6.75, and 6.43 ppm, were too broad to discuss in detail due to the paramagnetic Cu^{II}, whereas the signals in $^{13}C\{^1H\}$ NMR (151 MHz, $CDCl_3$, 20 °C) were not detected. ^{19}F NMR (564 MHz, $CDCl_3$, 20 °C): δ (ppm) –135.26 (s, 8F, Ar-F), –168.17 (s, 8F, Ar-F), –171.54 (s, 8F, Ar-F). UV/vis (CH_2Cl_2, λ_{max}[nm] (ε, 10^5 $M^{-1}cm^{-1}$)): 328 (0.26), 434 (1.0), 548 (0.11), 702 (0.029). HRMS (ESI-TOF) *m/z*: [M – $C_{24}BF_{20}$]$^+$ Calcd for $C_{44}H_{28}CuN_3S^+$ 693.1294; Found 693.1294. [M – $C_{44}H_{28}CuN_3S$]$^-$ Calcd for $C_{24}BF_{20}^-$ 678.9779; Found 678.9779. This compound was further characterized by single-crystal X-ray analysis.

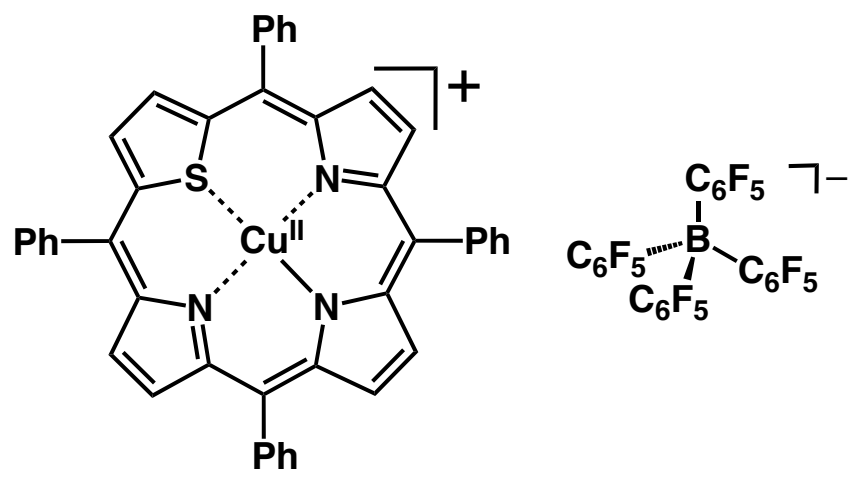

Cu^{II} complex of 5,10,15,20-tetraphenyl-21-thiaporphyrin as a PCCp$^{–}$ ion pair, 1cu^{+}-PCCp$^{–}$. To a MeOH solution (30 mL) of **1cu**$^{+}$-Cl$^{–}$ (15.3 mg, 21.0 μmol) was added sodium pentacyanocyclopentadienide (NaPCCp)[S4] (4.53 mg, 21.2 μmol) and the reaction mixture was stirred at r.t. for 1 h, followed by filtration and evaporation to dryness. The residue was purified by silica gel column chromatography (Wakogel C-300; eluent: 5% MeOH/CH_2Cl_2) and recrystallized from CH_2Cl_2/*n*-hexane to afford **1cu**$^{+}$-PCCp$^{–}$ (13.2 mg, 15.0 μmol, 63%) as a purple solid. R_f = 0.38 (5% MeOH/CH_2Cl_2). The signals in ^{1}H NMR (600 MHz, $CDCl_3$, 20 °C), observed at 9.36, 8.49, 6.86, and 6.48 ppm, were too broad to discuss in detail due to the paramagnetic Cu^{II}, whereas the signals in ^{13}C{^{1}H} NMR (151 MHz, $CDCl_3$, 20 °C) were not detected. UV/vis (CH_2Cl_2, λ_{max}[nm] (ε, 10^5 $M^{–1}cm^{–1}$)): 256 (0.35), 328 (0.26), 434 (1.0), 549 (0.11), 702 (0.025). HRMS (ESI-TOF) *m/z*: [M – $C_{10}N_5$]$^+$ Calcd for $C_{44}H_{28}CuN_3S^+$ 693.1294; Found 693.1294. [M – $C_{44}H_{28}CuN_3S$]$^–$ Calcd for $C_{10}N_5^–$ 190.0159; Found 190.0159. This compound was further characterized by single-crystal X-ray analysis.

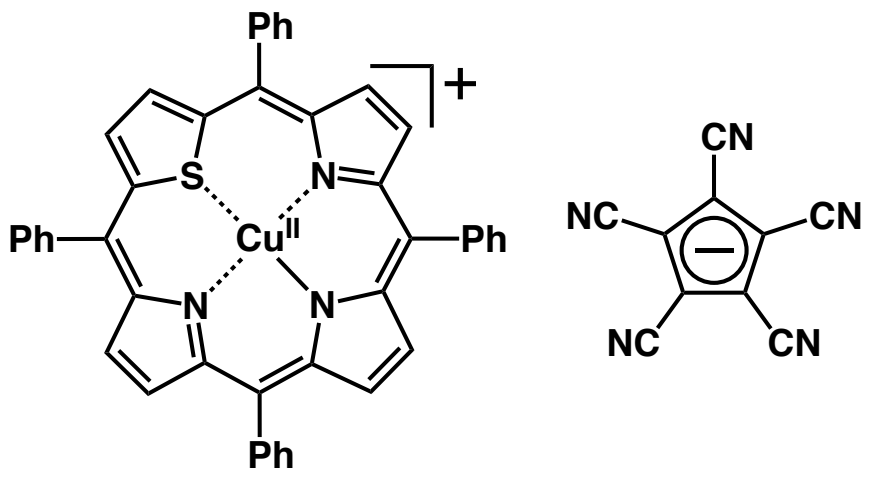

Cu^{II} complex of 10,15-bis(pentafluorophenyl)-5,20-diphenyl-21-thiaporphyrin as a Cl$^–$ ion pair, 2cu$^+$-Cl$^–$. To a $CHCl_3$/MeOH solution (40 mL/60 mL) of **2** (42.0 mg, 51.7 μmol) was added $CuCl_2$ (51.3 mg, 0.266 mmol). The reaction mixture was heated to reflux and stirred under air for 10 h, followed by filtration and evaporation to dryness. The residue was purified by silica gel column chromatography (Wakogel C-300; eluent: CH_2Cl_2 to 10% MeOH/CH_2Cl_2) and recrystallized from CH_2Cl_2/*n*-hexane to afford **2cu**$^+$-Cl$^–$ (80.0 mg, 87.9 μmol, 86%) as a green solid. R_f = 0.49 (10% MeOH/CH_2Cl_2). The signals in ^{1}H NMR (600 MHz, $CDCl_3$, 20 °C), observed at 7.85 ppm, were too broad to discuss in detail due to the paramagnetic Cu^{II}, whereas the signals in ^{13}C{^{1}H} NMR (151 MHz, $CDCl_3$, 20 °C) were not detected. ^{19}F NMR (564 MHz, $CDCl_3$, 20 °C): δ (ppm) –138.97 (m, 4F, Ar-F), –153.81 (s, 2F, Ar-F), –164.11 (s, 4F, Ar-F). UV/vis (CH_2Cl_2, λ_{max}[nm] (ε, 10^5 $M^{–1}cm^{–1}$)): 391 (0.46), 471 (0.75), 626 (0.092), 711 (0.062). HRMS (ESI-TOF) *m/z*: [M – Cl]$^+$ Calcd for $C_{44}H_{18}CuF_{10}N_3S^+$ 873.0352; Found 873.0352.

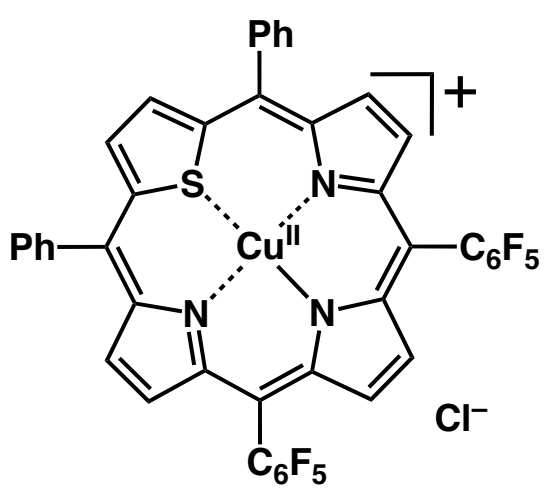

Cu^{II} complex of 10,15-bis(pentafluorophenyl)-5,20-diphenyl-21-thiaporphyrin as a $PF_6^–$ ion pair, 2cu$^+$-$PF_6^–$. To a $CHCl_3$/MeOH solution (40 mL/60 mL) of **2cu**$^+$-Cl$^–$ (28.9 mg, 40.0 μmol) was added $AgPF_6$ (20.2 mg, 79.9 μmol) and the reaction mixture was stirred at r.t. for 15 min, followed by filtration and evaporation to dryness. The residue was purified by silica gel column chromatography (Wakogel C-300; eluent: 5% MeOH/CH_2Cl_2) and recrystallized from CH_2Cl_2/*n*-hexane to afford **2cu**$^+$-$PF_6^–$ (22.3 mg, 26.7 μmol, 67%) as a green solid. R_f = 0.53 (10% MeOH/CH_2Cl_2). The signals in ^{1}H NMR (600 MHz, $CDCl_3$, 20 °C), observed at 8.85 and 7.70 ppm, were too broad to discuss in detail due to the paramagnetic Cu^{II}, whereas the signals in ^{13}C{^{1}H} NMR (151 MHz, $CDCl_3$, 20 °C) and ^{19}F NMR (564 MHz, $CDCl_3$, 20 °C) were not detected. UV/vis (CH_2Cl_2, λ_{max}[nm] (ε, 10^5 $M^{–1}cm^{–1}$)): 331 (0.34), 432 (1.0), 543 (0.12), 623 (0.074), 709 (0.054). HRMS (ESI-TOF) *m/z*: [M – F_6P]$^+$ Calcd for $C_{44}H_{18}CuF_{10}N_3S^+$ 873.0352; Found 873.0352. [M – $C_{44}H_{18}CuF_{10}N_3S$]$^–$ Calcd for $F_6P^–$ 144.9647; Found 144.9647.

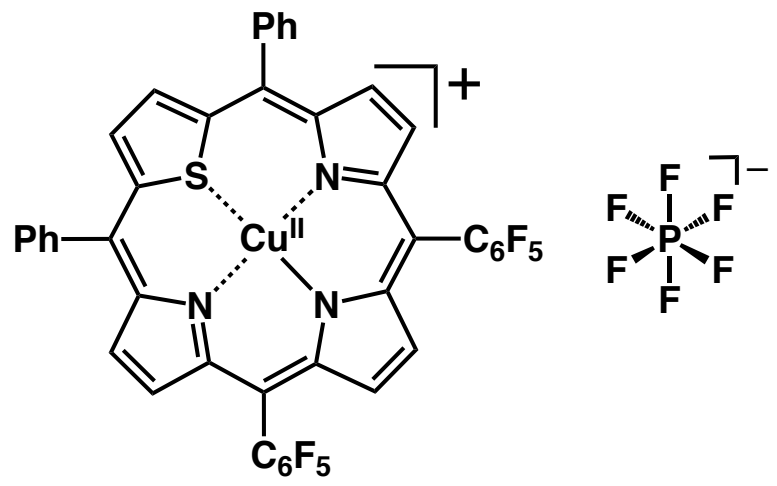

Cu^{II} complex of 10,15-bis(pentafluorophenyl)-5,20-diphenyl-21-thiaporphyrin as a $B(C_6F_5)_4^–$ ion pair, 2cu$^+$-$B(C_6F_5)_4^–$. To a MeOH/$CHCl_3$ solution (15 mL/15 mL) of **2cu**$^+$-Cl$^–$ (20.0 mg, 22.0 μmol) was added $LiB(C_6F_5)_4$ (18.1 mg, 26.4 μmol) and the reaction mixture was stirred at r.t. for 30 min, followed by filtration and evaporation to dryness. The residue was purified by silica gel column chromatography (Wakogel C-300; eluent: 25% MeOH/$CHCl_3$) and recrystallized from $CHCl_3$/*n*-hexane to afford **2cu**$^+$-$B(C_6F_5)_4^–$ (25.9 mg, 16.7 μmol, 76%) as a green solid. R_f = 0.74 (10% MeOH/CH_2Cl_2). The signals in ^{1}H NMR (600 MHz, $CDCl_3$, 20 °C), observed at 8.32, 7.37, and 6.67 ppm, were too broad to discuss in detail due to the paramagnetic Cu^{II}, whereas the signals in ^{13}C{^{1}H} NMR (151 MHz, $CDCl_3$, 20 °C) were not detected. The signals in ^{19}F NMR (564 MHz, $CDCl_3$, 20 °C), observed at –134.60, –149.94, –161.18, –167.66, and –171.70 ppm, were too broad to discuss in detail due to the paramagnetic Cu^{II}. HRMS (ESI-TOF) *m/z*: [M – $C_{24}BF_{20}$]$^+$ Calcd for $C_{44}H_{18}CuF_{10}N_3S^+$ 873.0352; Found 873.0352. [M – $C_{44}H_{18}CuF_{10}N_3S$]$^–$ Calcd for $C_{24}BF_{20}^–$ 678.9779; Found 678.9779.

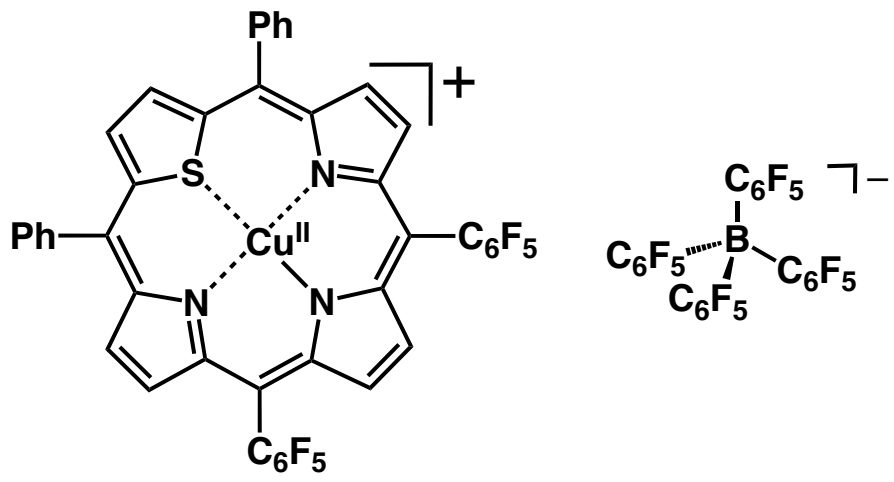

Cu^{II} complex of 10,15-bis(pentafluorophenyl)-5,20-diphenyl-21-thiaporphyrin as a PCCp$^-$ ion pair, 2cu$^+$-PCCp$^-$. To a $CHCl_3$/MeOH solution (40 mL/60 mL) of **2cu**$^+$-Cl^- (16.2 mg, 17.8 μmol) was added NaPCCp (4.00 mg, 18.7 μmol) and the reaction mixture was stirred at r.t. for 30 min, followed by filtration and evaporation to dryness. The residue was purified by silica gel column chromatography (Wakogel C-300; eluent: 5% MeOH/CH_2Cl_2) and recrystallized from CH_2Cl_2/*n*-hexane to afford **2cu**$^+$-PCCp$^-$ (12.1 mg, 11.3 μmol, 63%) as a green solid. R_f = 0.36 (5% MeOH/CH_2Cl_2). The signals in ^{1}H NMR (600 MHz, $CDCl_3$, 20 °C), observed at 8.05, 7.57, and 7.22 ppm, were too broad to discuss in detail due to the paramagnetic Cu^{II}, whereas the signals in ^{13}C{^{1}H} NMR (151 MHz, $CDCl_3$, 20 °C) were not detected. ^{19}F NMR (564 MHz, $CDCl_3$, 20 °C): δ (ppm) –135.26 (s, 4F, Ar-F), –168.17 (s, 2F, Ar-F), –171.54 (s, 4F, Ar-F). UV/vis (CH_2Cl_2, λ_{max}[nm] (ε, 10^5 $M^{-1}cm^{-1}$)): 256 (0.90), 330 (0.26), 433 (1.0), 546 (0.099), 696 (0.018). HRMS (ESI-TOF) *m/z*: [M – $C_{10}N_5$]$^+$ Calcd for $C_{44}H_{18}CuF_{10}N_3S^+$ 873.0352; Found 873.0352. [M – $C_{44}H_{18}CuF_{10}N_3S$]$^-$ Calcd for $C_{10}N_5^-$ 190.0159; Found 190.0159.

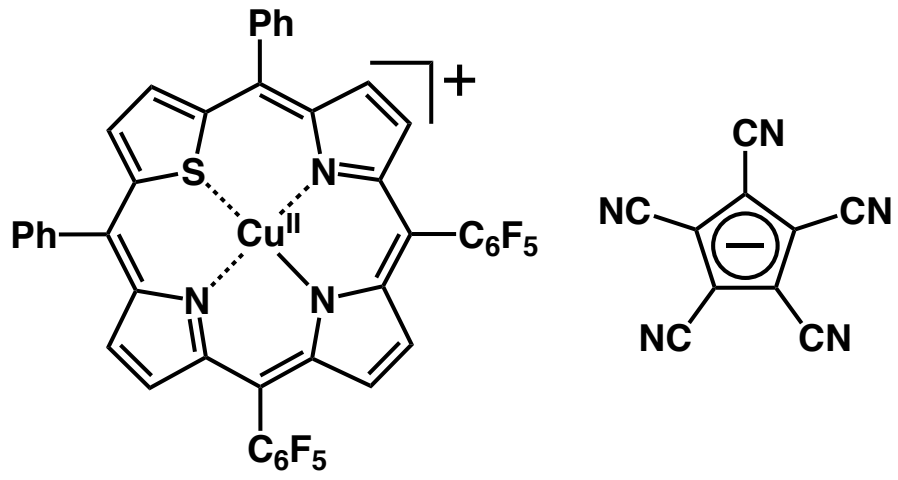

[S1] (a) C. E. Stilts, M. I. Nelen, D. G. Hilmey, S. R. Davies, S. O. Gollnick, A. R. Oseroff, S. L. Gibson, R. Hilf and M. R. Detty, *J. Med. Chem.*, 2000, **43**, 2403–2410; (b) D. G. Hilmey, M. Abe, M. I. Nelen, C. E. Stilts, G. A. Baker, S. N. Baker, F. V. Bright, S. R. Davies, S. O. Gollnick, A. R. Oseroff, S. L. Gibson, R. Hilf and M. R. Detty, *J. Med. Chem.*, 2002, **4**, 449–461.

[S2] L. Latos-Grażyński, J. Lisowski, M. M. Olmstead and A. L. Balch, *J. Am. Chem. Soc.*, 1987, **109**, 4428–4429.

[S3] H. Mori, J. M. Lim, D. Kim and A. Osuka, *Angew. Chem. Int. Ed.*, 2013, **52**, 12997–13001.

[S4] (a) O. W. Webster, *J. Am. Chem. Soc.*, 1965, **87**, 1820–1821; (b) T. Sakai, S. Seo, J. Matsuoka and Y. Mori, *J. Org. Chem.*, 2013, **78**, 10978–10985.

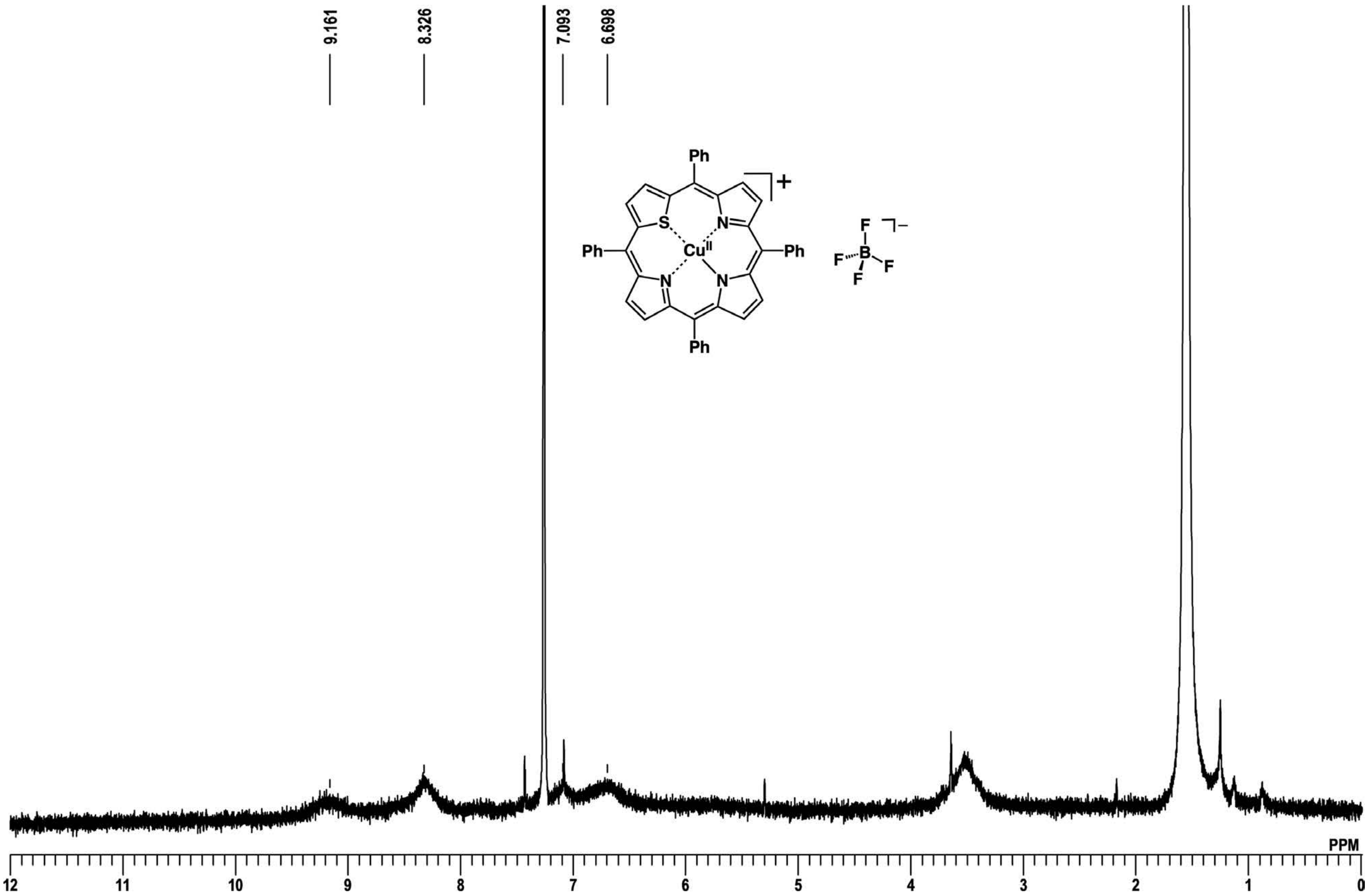

Fig. S1 ^{1}H NMR spectrum of **1cu**$^{+}$-BF_4^{-} in $CDCl_3$ at 20 °C.

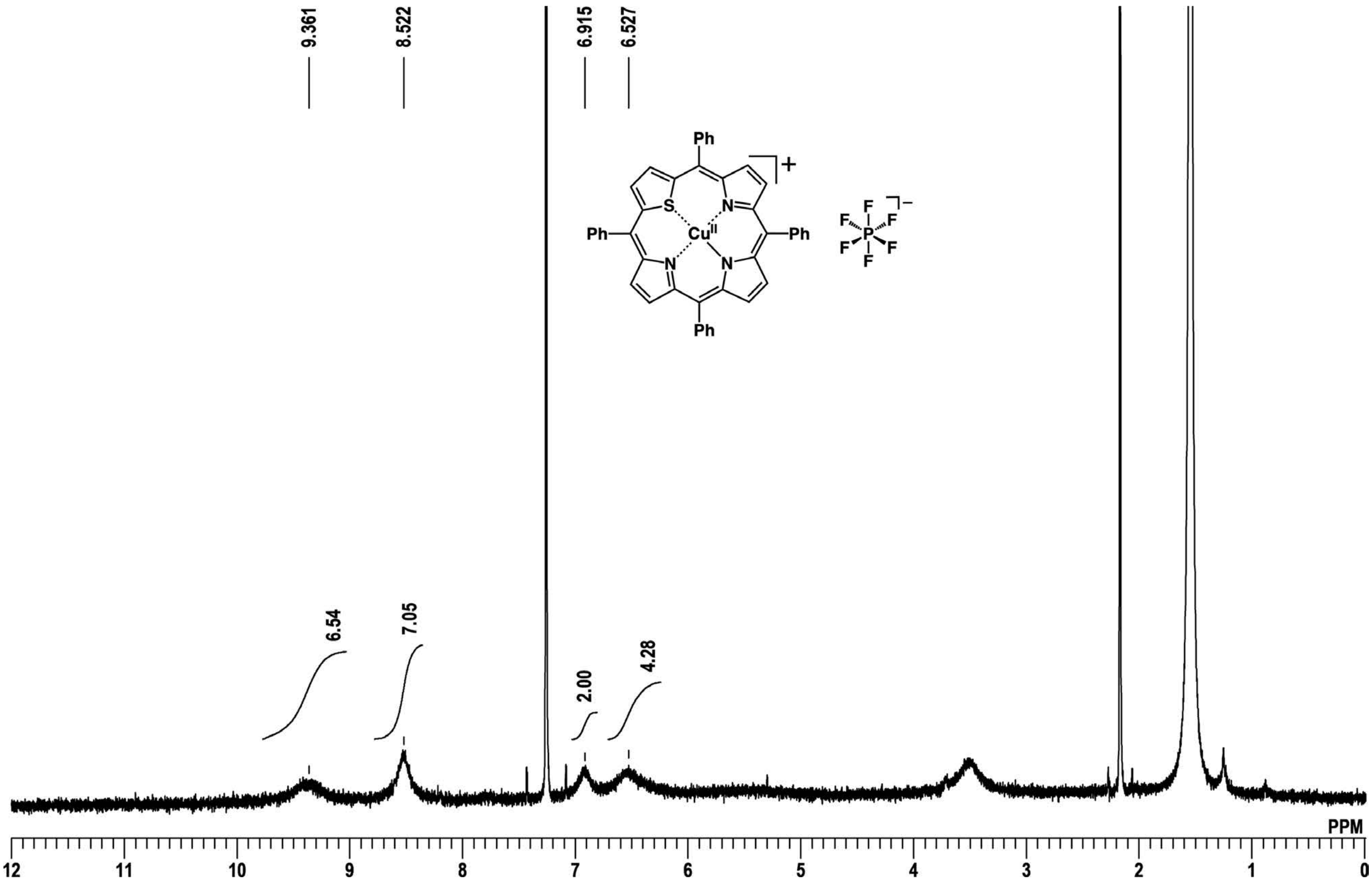

Fig. S2 ^{1}H NMR spectrum of **1cu**$^{+}$-PF_6^{-} in $CDCl_3$ at 20 °C.

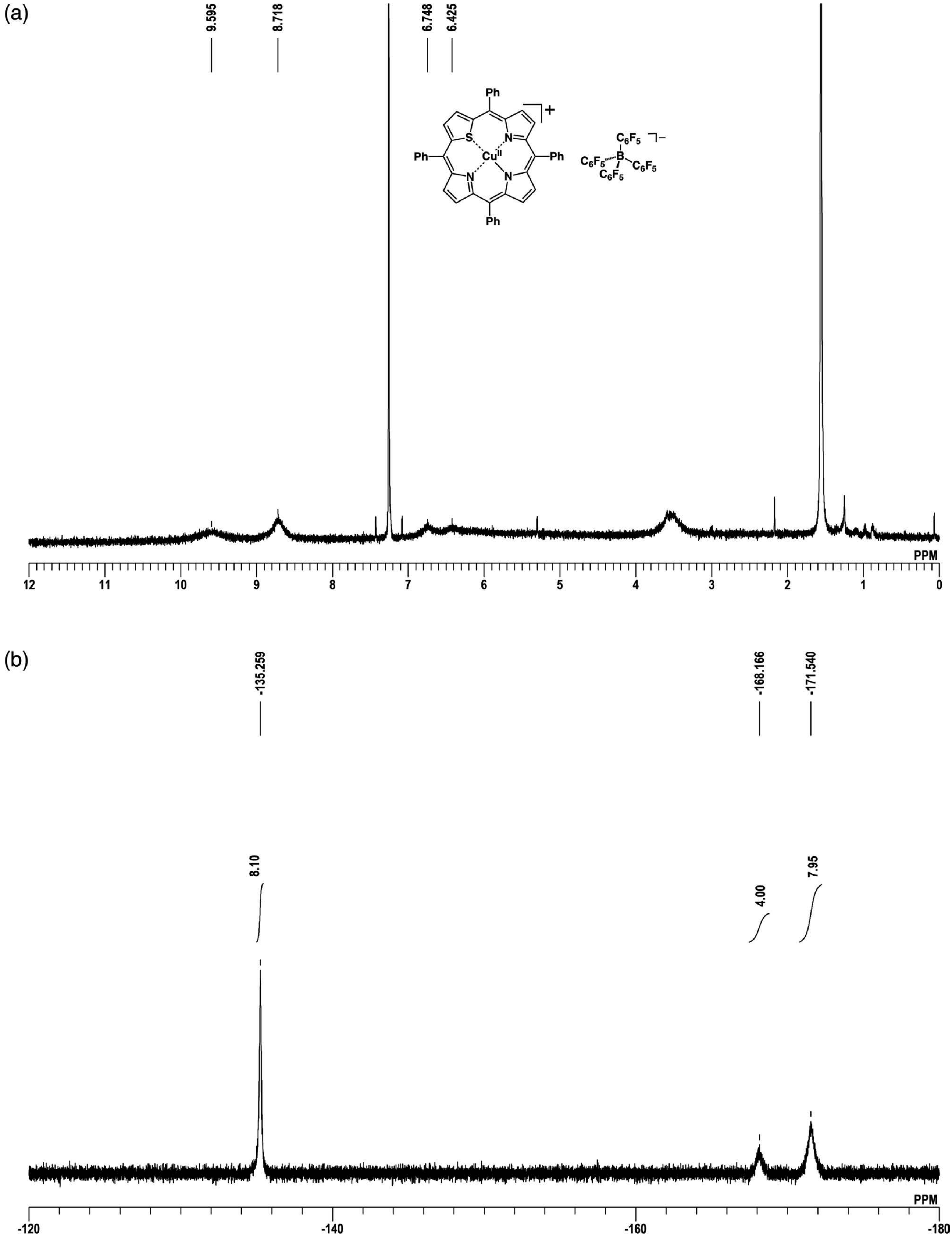

Fig. S3 (a) ^{1}H NMR and (b) ^{19}F NMR spectra of **1cu**$^{+}$-$B(C_6F_5)_4^{-}$ in $CDCl_3$ at 20 °C.

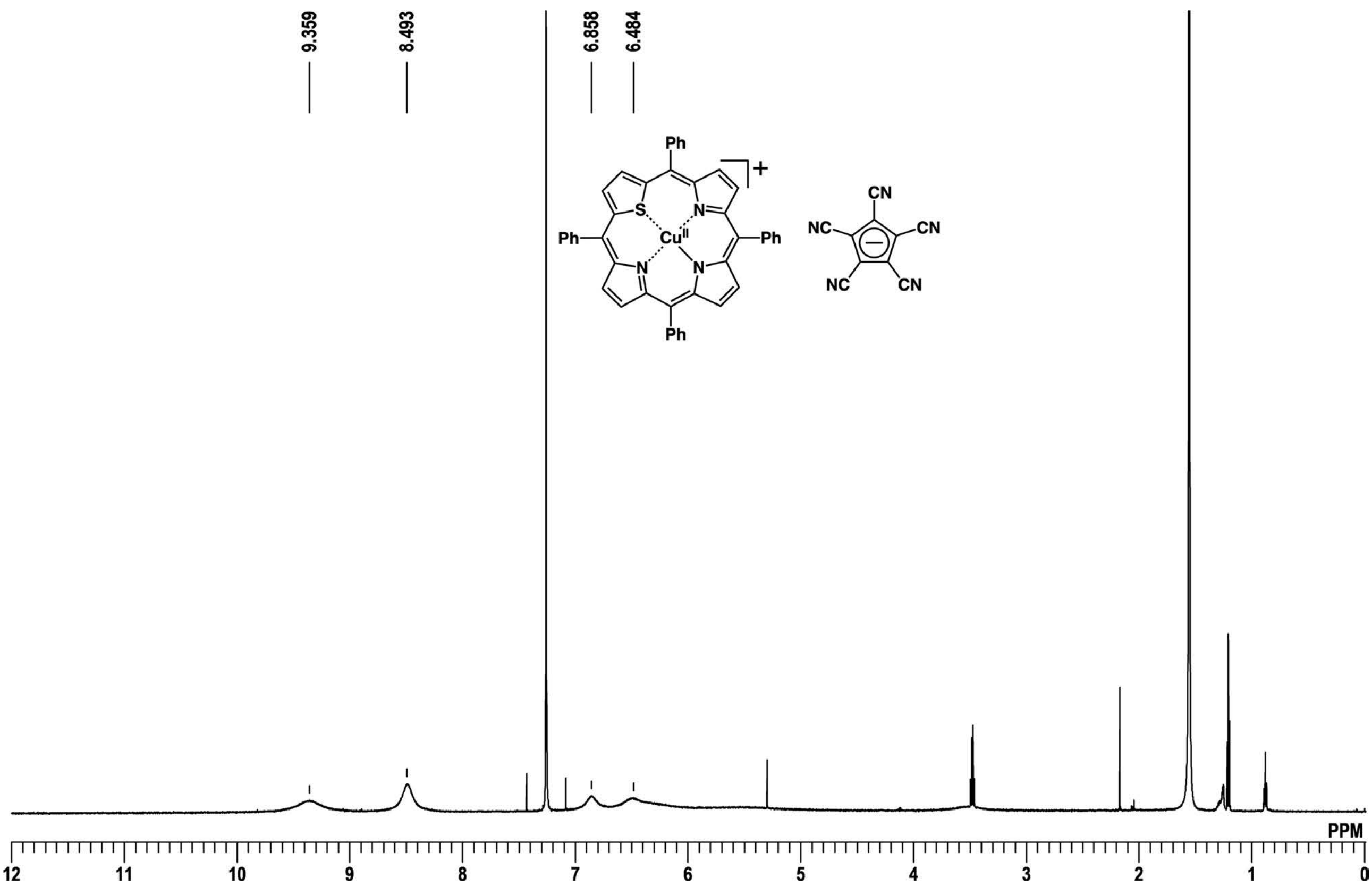

Fig. S4 ^{1}H NMR spectrum of **1cu**$^+$-PCCp$^-$ in $CDCl_3$ at 20 °C.

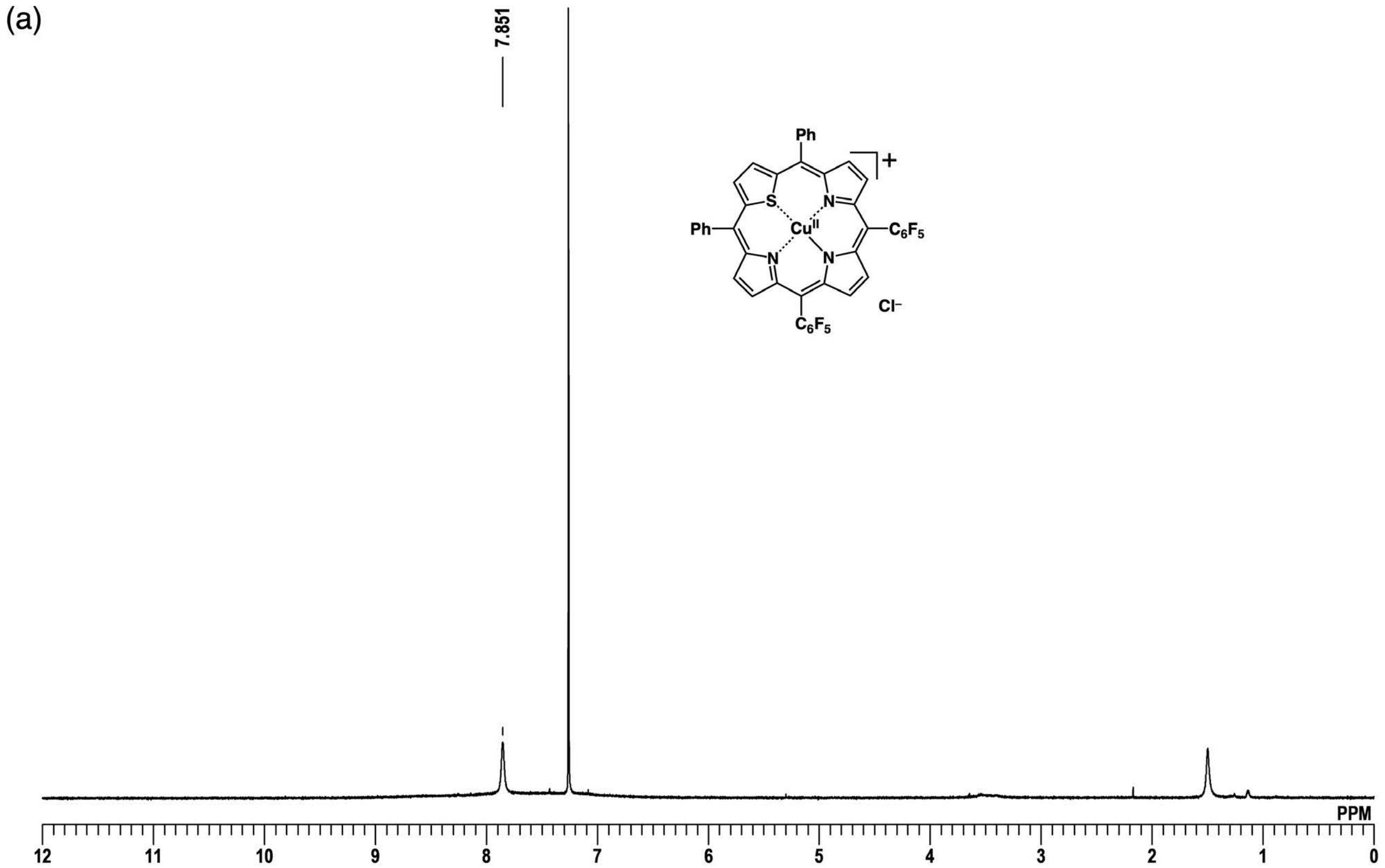

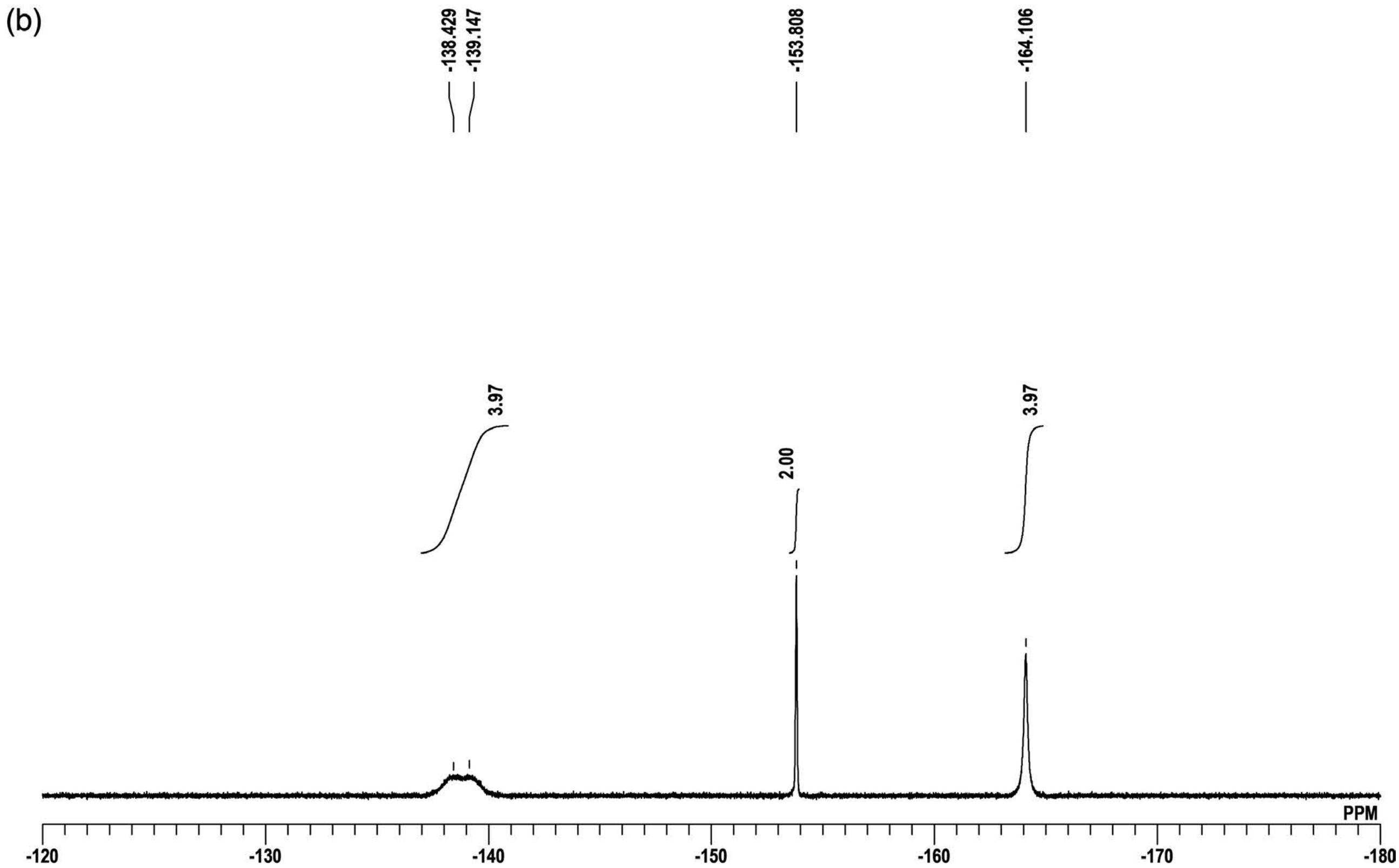

Fig. S5 (a) ^{1}H NMR and (b) ^{19}F NMR spectra of **2cu**$^{+}$-Cl^{-} in $CDCl_3$ at 20 °C.

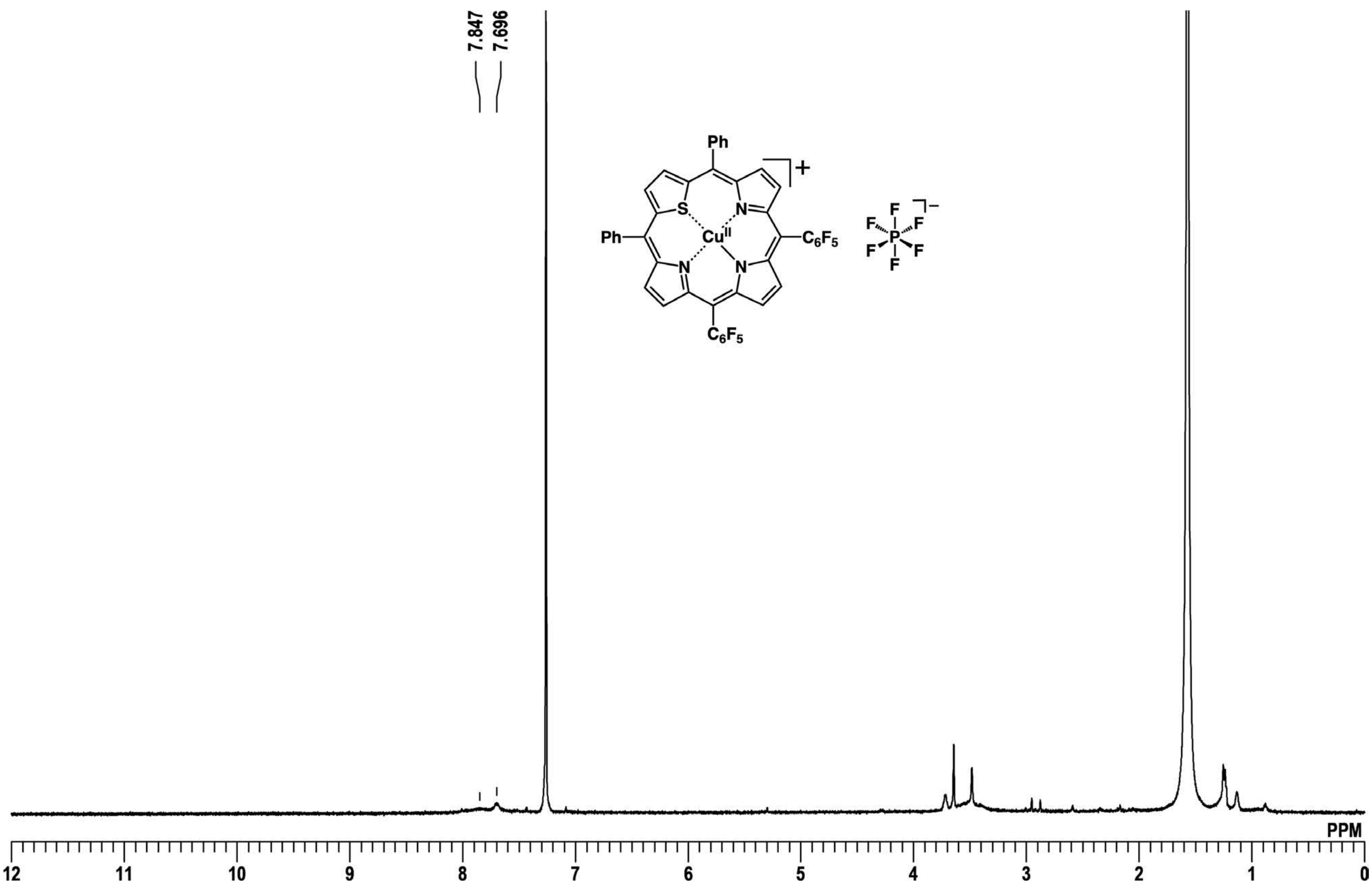

Fig. S6 ^{1}H NMR spectrum of **2cu**$^+$-PF_6^- in $CDCl_3$ at 20 °C.

(a)

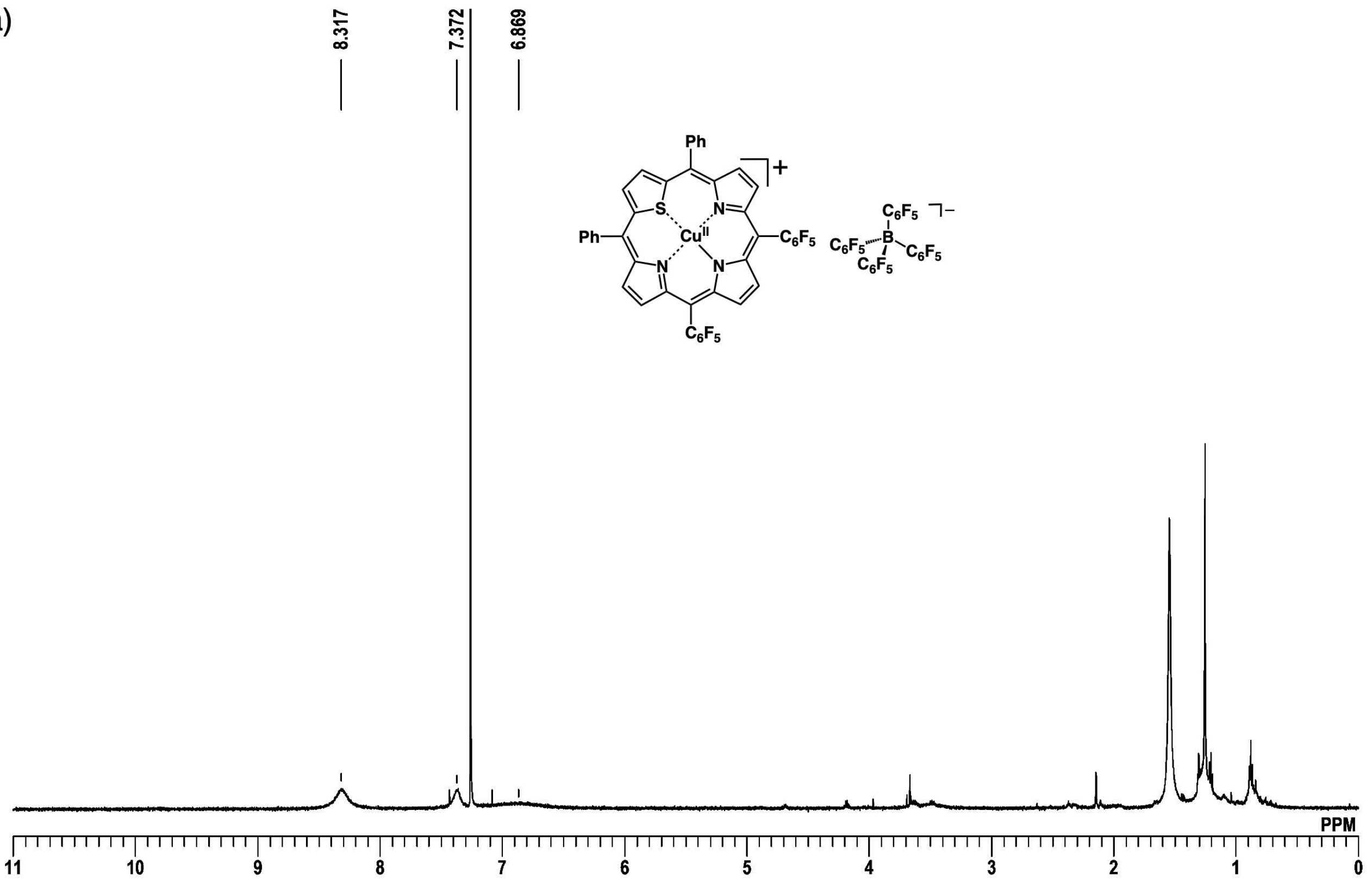

(b)

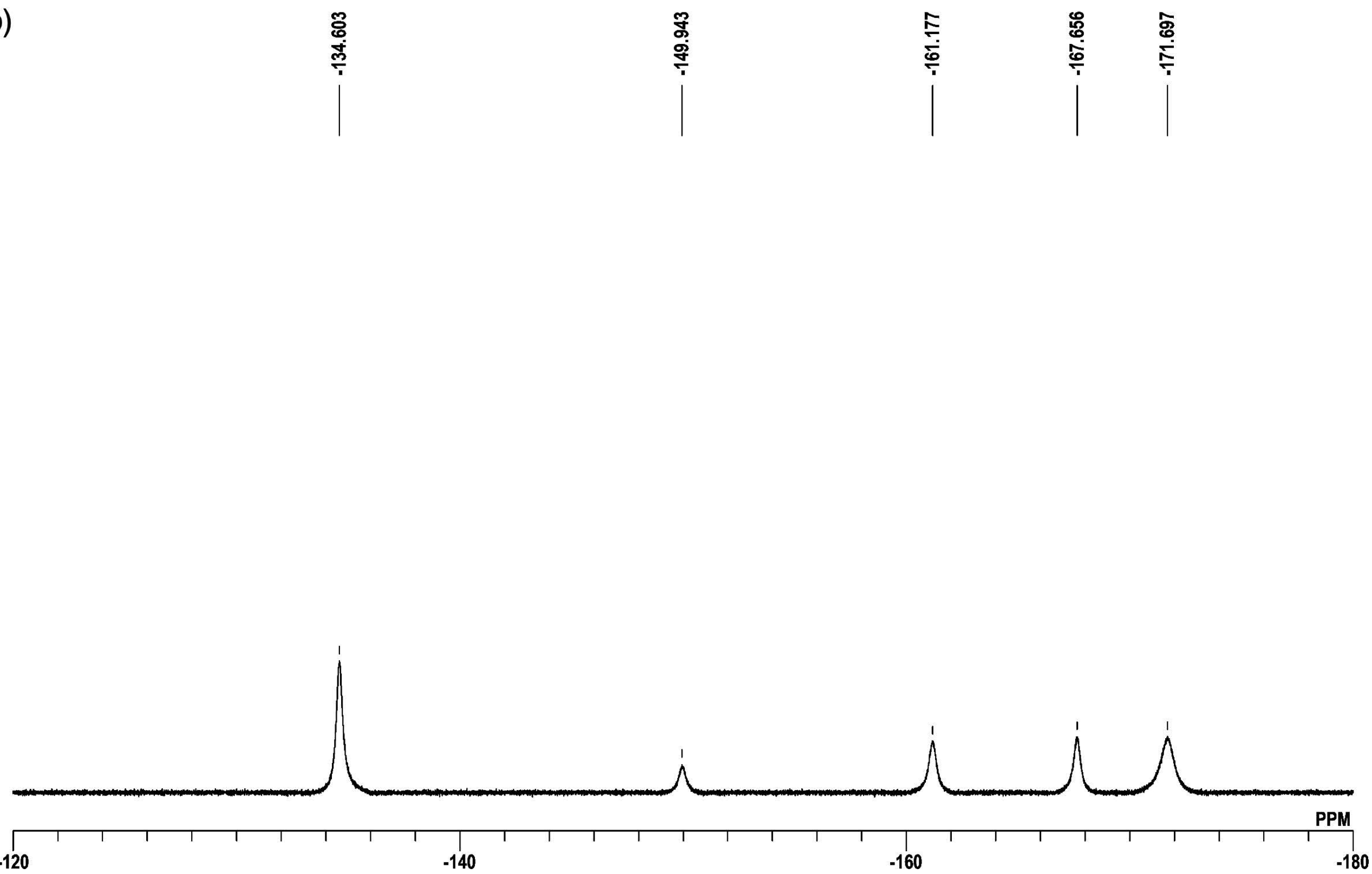

Fig. S7 (a) ^{1}H NMR and (b) ^{19}F NMR spectra of **2cu**$^{+}$-$B(C_6F_5)_4^{-}$ in $CDCl_3$ at 20 °C.

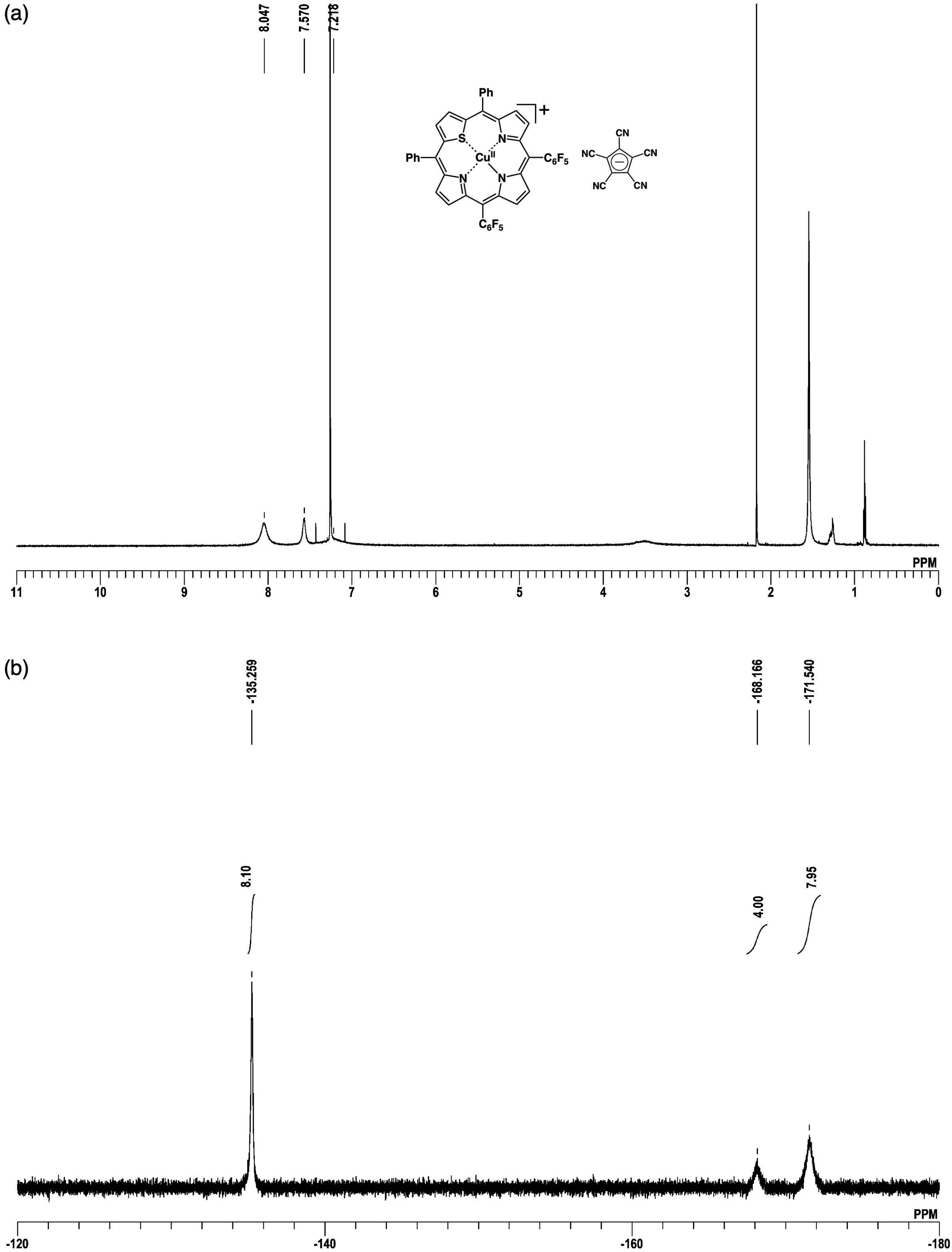

Fig. S8 (a) ^{1}H NMR and (b) ^{19}F NMR spectra of **2cu**$^+$-PCCp$^-$ in CDCl$_3$ at 20 °C.

2. X-ray crystallographic data

Method for single-crystal X-ray analysis. Crystallographic data are summarized in Table S1. A single crystal of **1cu**$^{+}$-BF_4^{-} was obtained by vapor diffusion of *n*-hexane into a CH_2Cl_2 solution with a small amount of 1-butanol. The data crystal was a purple prism of approximate dimensions 0.13 mm × 0.08 mm × 0.01 mm. A single crystal of **1cu**$^{+}$-PF_6^{-} was obtained by vapor diffusion of *n*-hexane into a CH_2Cl_2 solution with a small amount of *n*-octane. The data crystal was a purple prism of approximate dimensions 0.07 mm × 0.05 mm × 0.05 mm. A single crystal of **1cu**$^{+}$-$B(C_6F_5)_4^{-}$ was obtained by vapor diffusion of *n*-hexane into a CH_2Cl_2 solution with a small amount of 1-butanol. The data crystal was a green plate of approximate dimensions 0.42 mm × 0.17 mm × 0.04 mm. A single crystal of **1cu**$^{+}$-PCCp^{-} was obtained by vapor diffusion of *n*-hexane into a CH_2Cl_2 solution with a small amount of 1-butanol. The data crystal was a purple prism of approximate dimensions 0.12 mm × 0.11 mm × 0.06 mm. A single crystal of **2cu**$^{+}$-PCCp^{-} was obtained by vapor diffusion of *n*-hexane into a CH_2Cl_2 solution with a small amount of 1-propanol. The data crystal was a purple prism of approximate dimensions 0.20 mm × 0.15 mm × 0.10 mm. The data of **1cu**$^{+}$-BF_4^{-} and **1cu**$^{+}$-$B(C_6F_5)_4^{-}$ were collected at 100 K on a Dectris EIGER X 1M diffractometer with Si (111) monochromated synchrotron radiation (λ = 0.81022 and 0.81170 Å, respectively) at BL40XU (SPring-8).[S5] The data of **1cu**$^{+}$-PF_6^{-} was collected at 100 K on a DECTRIS EIGER2 XE 16 M diffractometer with Si (111) monochromated synchrotron radiation (λ = 0.700 Å) at BL41XU (SPring-8).[S6] The data of **1cu**$^{+}$-PCCp^{-} and **2cu**$^{+}$-PCCp^{-} were collected at 90 and 100 K, respectively, on a DECTRIS PILATUS3 CdTe 1M diffractometer with Si (311) monochromated synchrotron radiation (λ = 0.41440 and 0.41360 Å, respectively) at BL02B1 (SPring-8).[S7] All the structures were solved by dual-space method. The structures were refined by a full-matrix least-squares method by using SHELXL 2014[S8] (Yadokari-XG[S9]) for **1cu**$^{+}$-PCCp^{-} and **2cu**$^{+}$-PCCp^{-} and SHELXL 2019[S8] (ShelXle[S10]) for **1cu**$^{+}$-BF_4^{-}, **1cu**$^{+}$-PF_6^{-}, and **1cu**$^{+}$-$B(C_6F_5)_4^{-}$. In each structure, the non-hydrogen atoms were refined anisotropically. Disordered solvents, presumably *n*-hexane in **1cu**$^{+}$-BF_4^{-} and CH_2Cl_2 in **1cu**$^{+}$-PF_6^{-} and **1cu**$^{+}$-$B(C_6F_5)_4^{-}$ were removed using the SQUEEZE protocol included in PLATON.[S11] CIF files (CCDC-2537959–2537963) can be obtained free of charge from the Cambridge Crystallographic Data Centre via www.ccdc.cam.ac.uk/data_request/cif.

Table S1 Crystallographic details.

	**1cu**$^{+}$-BF_4^{-}	**1cu**$^{+}$-PF_6^{-}	**1cu**$^{+}$-$B(C_6F_5)_4^{-}$	**1cu**$^{+}$-PCCp^{-}	**2cu**$^{+}$-PCCp^{-}
formula	$C_{44}H_{28}CuN_3S\cdot BF_4\cdot CH_2Cl_2\cdot C_6H_{14}$	$C_{44}H_{28}CuN_3S\cdot F_6P\cdot CH_2Cl_2$	$C_{44}H_{28}CuN_3S\cdot C_{24}BF_{20}\cdot CH_2Cl_2$	$C_{44}H_{28}CuN_3S\cdot C_{10}N_5\cdot CHCl_3$	$C_{44}H_{18}CuF_{10}N_3S\cdot C_{10}N_5\cdot 1.5CH_2Cl_2$
fw	952.20	1009.11	1458.27	1003.81	1191.77
crystal size, mm	0.13 × 0.08 × 0.01	0.07 × 0.05 × 0.05	0.42 × 0.17 × 0.04	0.12 × 0.11 × 0.06	0.20 × 0.15 × 0.10
crystal system	triclinic	monoclinic	monoclinic	triclinic	triclinic
space group	$P\bar{1}$ (no. 2)	$P2_1/c$ (no. 14)	$P2_1/n$ (no. 14)	$P\bar{1}$ (no. 2)	$P\bar{1}$ (no. 2)
a, Å	12.3586(5)	13.7563(4)	13.4605(2)	7.394(7)	13.338(8)
b, Å	13.3252(9)	16.2597(4)	27.1550(6)	16.27(3)	14.435(8)
c, Å	13.7953(9)	18.8801(4)	16.7657(3)	20.57(2)	15.236(10)
α, °	101.013(6)	90	90	109.80(4)	108.451(16)
β, °	104.250(5)	90.067(2)	99.115(2)	95.927(16)	103.792(13)
γ, °	94.031(4)	90	90	90.33(4)	98.349(8)
V, Å^3	2144.9(2)	4222.97(18)	6050.8(2)	2313(6)	2624(3)
ρ_{calcd}, gcm^{-3}	1.474	1.587	1.601	1.441	1.508
Z	2	4	4	2	2
T, K	100(2)	100(2)	100(2)	90(2)	100(2)
μ, mm^{-1}	1.054 ^{a}	0.888 ^{a}	0.846 ^{a}	0.178 ^{a}	0.167 ^{a}
no. of reflns	26807	34406	74099	67061	20676
no. of unique reflns	9934	11810	14504	10592	11297
variables	543	708	834	672	717
λ, Å	0.81022 ^{a}	0.700 ^{a}	0.81170 ^{a}	0.41440 ^{a}	0.41360 ^{a}
R_1 ($I > 2\sigma(I)$)	0.0530	0.0993	0.0682	0.0775	0.1078
wR_2 ($I > 2\sigma(I)$)	0.1540	0.2537	0.1985	0.1799	0.3195
GOF	0.682	1.072	0.834	1.215	1.082

^{a} Synchrotron radiation.

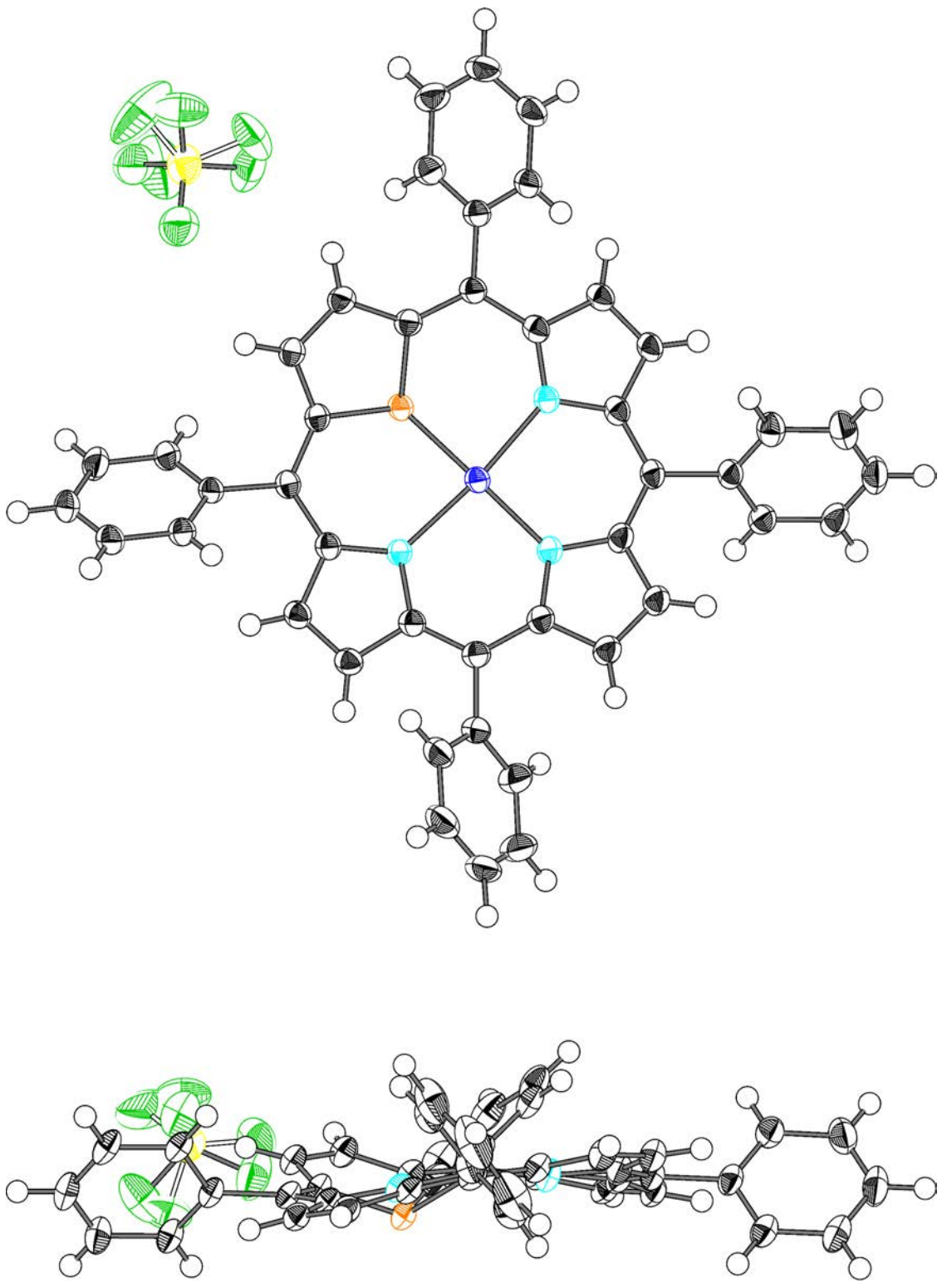

Fig. S9 Ortep drawing of single-crystal X-ray structure (top and side views) of **1cu**$^{+}$-BF_4^{-}. Disordered structures are represented by gray and white bonds for major and minor structures, respectively, in the ratio of 64 : 36 for the fluorine atoms in BF_4^{-}. Thermal ellipsoids are scaled to the 50% probability level. Solvent molecules are omitted for clarity. Atom color code: black, white (sphere), yellow, cyan, yellow green, orange, and blue refer to carbon, hydrogen, boron, nitrogen, fluorine, sulfur, and copper, respectively.

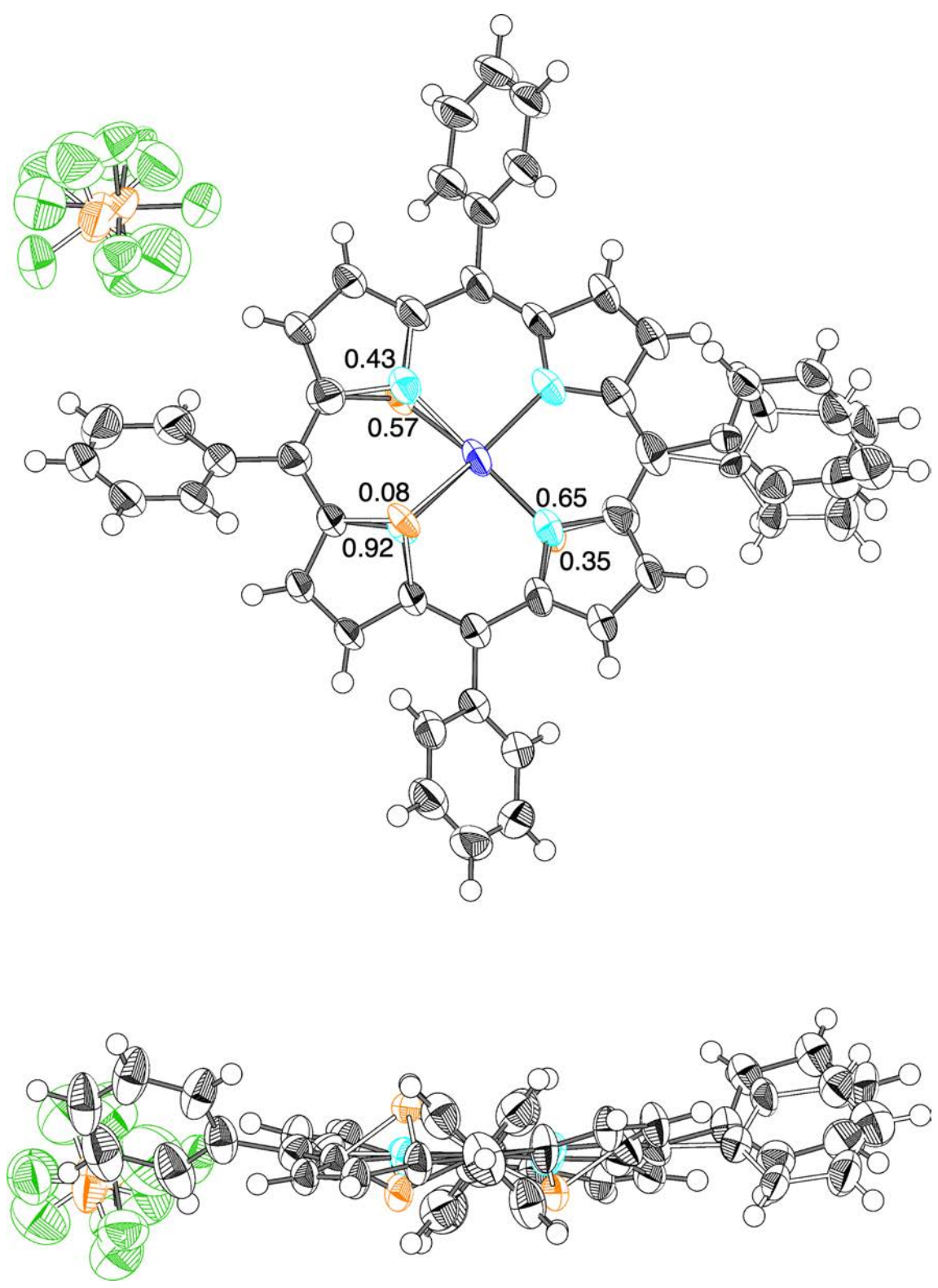

Fig. S10 Ortep drawing of single-crystal X-ray structure (top and side views) of **1cu**$^{+}$-PF_6^{-}. Disordered structures are represented by gray and white bonds for major and minor structures, respectively, in the ratio of 57 : 35 : 8, 73 : 27, and 66 : 34 for the porphyrin inner atoms (according to the existence of sulfur), a PF_6^{-} unit, and two phenyl groups, respectively. Thermal ellipsoids are scaled to the 50% probability level. Solvent molecules are omitted for clarity. Atom color code: black, white (sphere), blue, yellow green, light orange, orange, and bule refer to carbon, hydrogen, nitrogen, fluorine, phosphorus, sulfur, and copper, respectively.

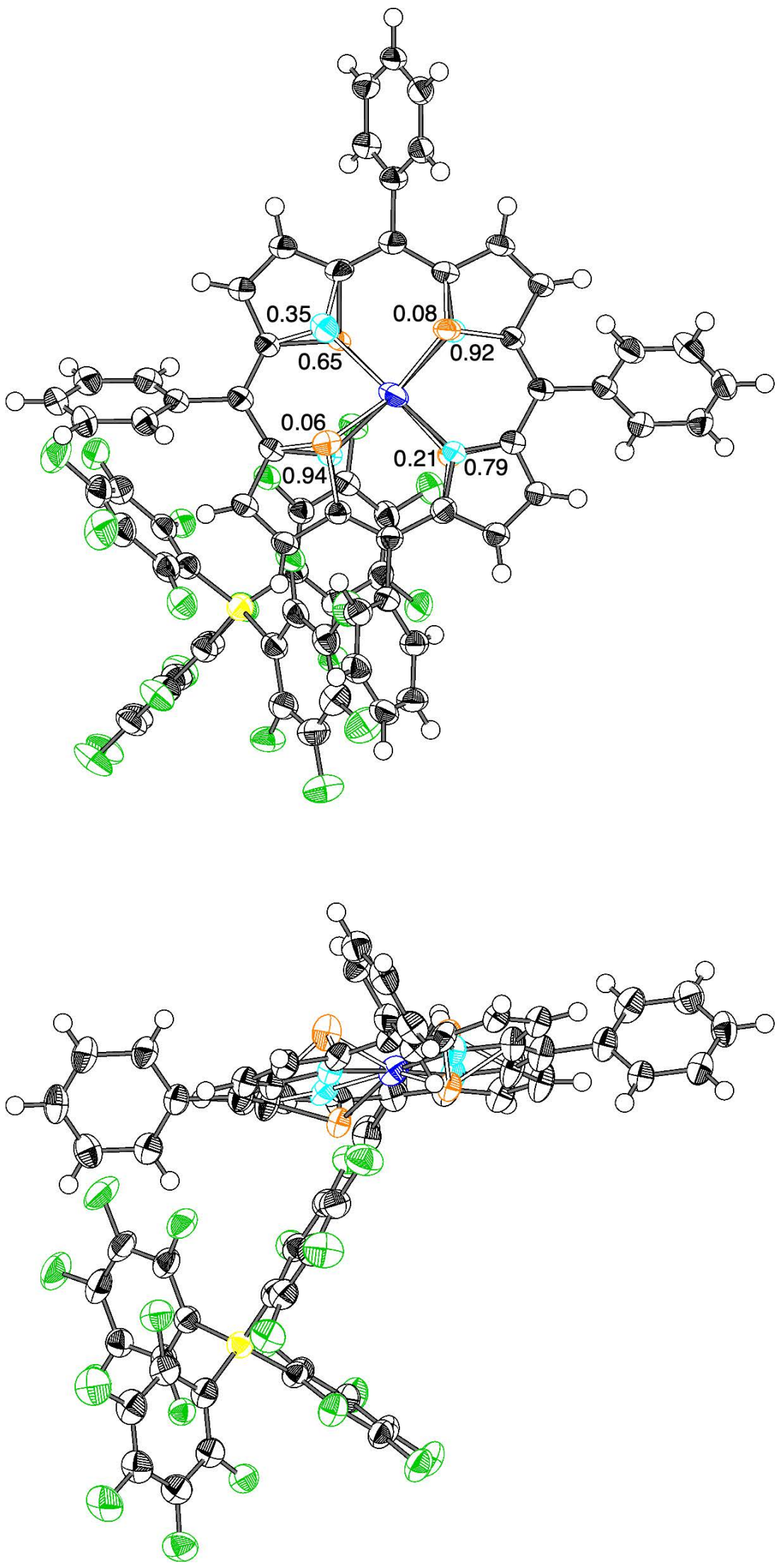

Fig. S11 Ortep drawing of single-crystal X-ray structure (top and side views) of **1cu**$^{+}$-B(C_6F_5)$_4^{-}$. Disordered structures are represented by gray and white bonds for major and minor structures, respectively, in the ratio of 65 : 21 : 8 : 6 for the porphyrin inner atoms (according to the existence of sulfur). Thermal ellipsoids are scaled to the 50% probability level. Solvent molecules are omitted for clarity. Atom color code: black, white (sphere), yellow, cyan, yellow green, orange, and blue refer to carbon, hydrogen, boron, nitrogen, fluorine, sulfur, and copper, respectively.

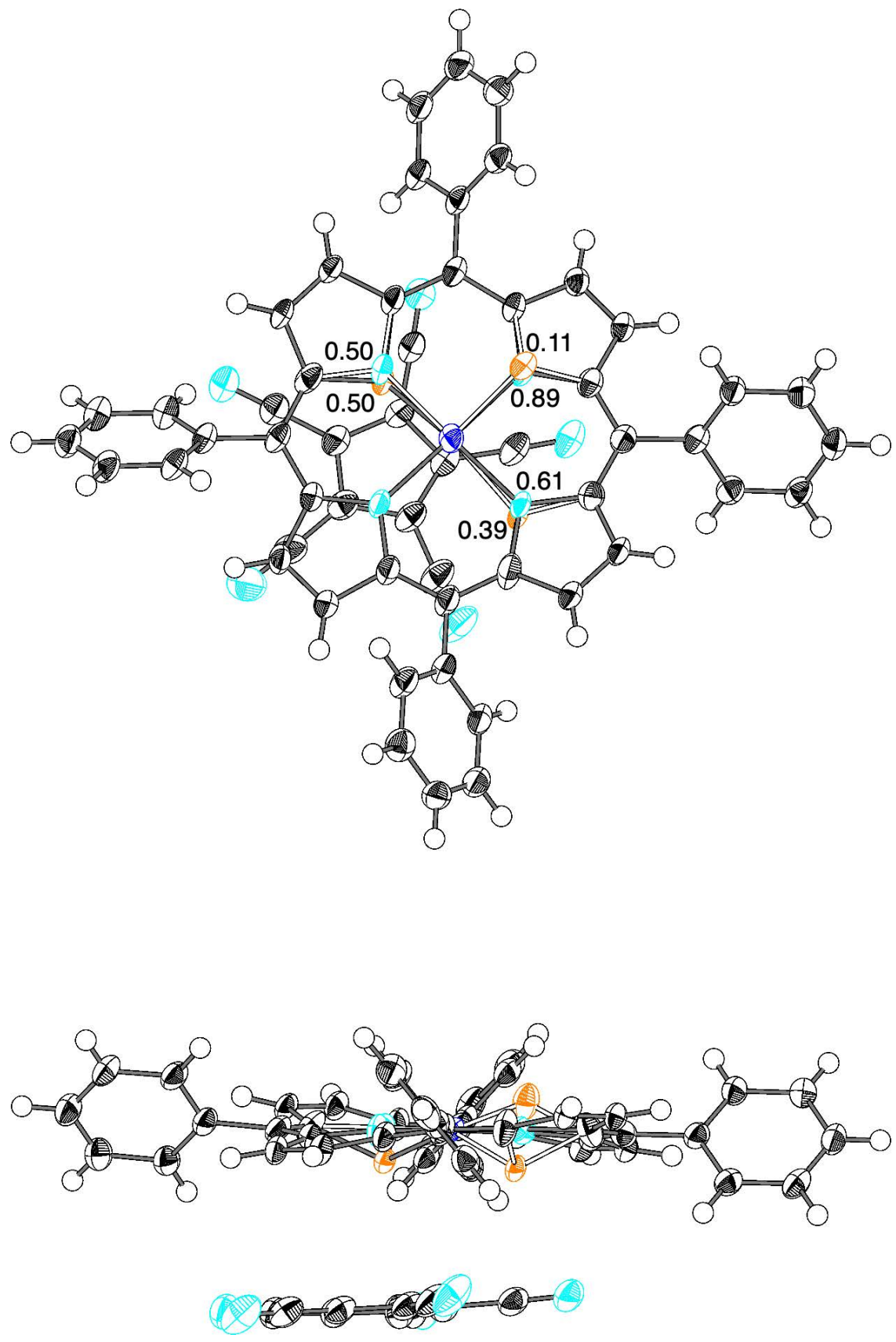

Fig. S12 Ortep drawing of single-crystal X-ray structure (top and side views) of **1cu**$^{+}$-PCCp^{-}. Disordered structures are represented by gray and white bonds for major and minor structures, respectively, in the ratio of 50 : 39 : 11 for the porphyrin inner atoms (according to the existence of sulfur). Thermal ellipsoids are scaled to the 50% probability level. Solvent molecules are omitted for clarity. Atom color code: black, white (sphere), cyan, orange, and blue refer to carbon, hydrogen, nitrogen, sulfur, and copper, respectively.

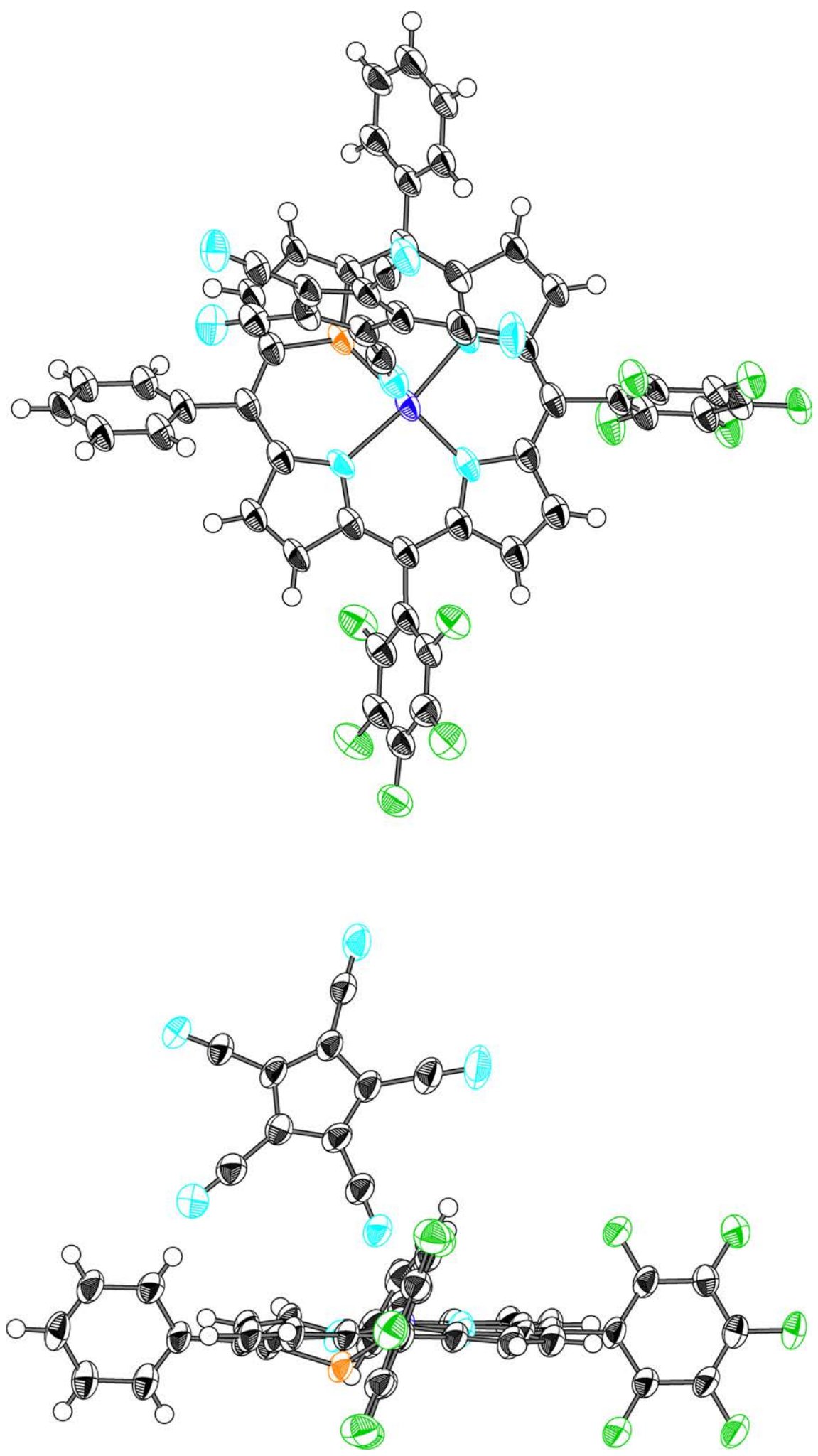

Fig. S13 Ortep drawing of single-crystal X-ray structure (top and side views) of **2cu**$^{+}$-PCCp^{-}. Thermal ellipsoids are scaled to the 50% probability level. Solvent molecules are omitted for clarity. Atom color code: black, white (sphere), cyan, yellow green, orange, and blue refer to carbon, hydrogen, nitrogen, fluorine, sulfur, and copper, respectively.

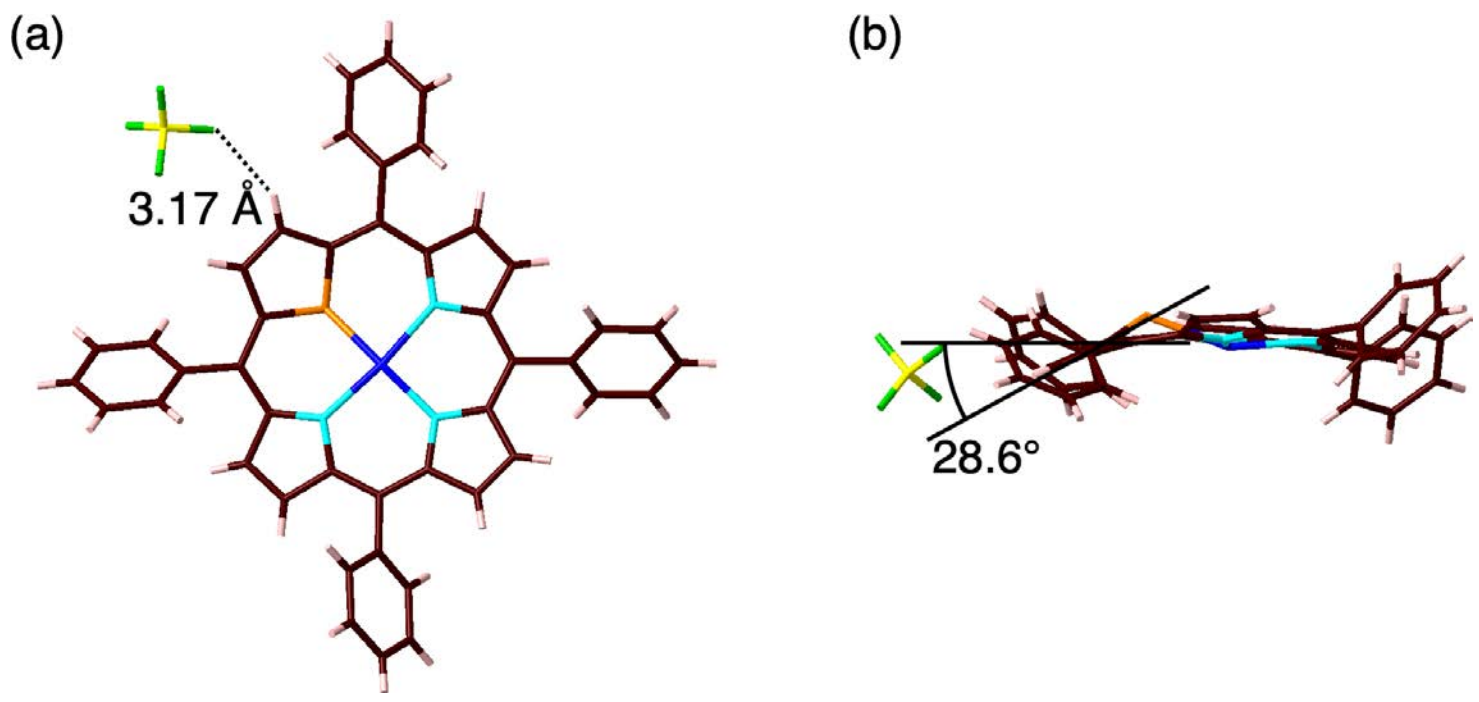

Fig. S14 Crystal structure of **1cu**$^{+}$-BF_4^{-} as (a) top and (b) side views. The dihedral angle between the thiophene plane and the core porphyrin plane (25 atoms) is 28.6°. Mean-plane deviation of the **1cu**$^{+}$ core part (25 atoms) and τ_4 value[S12] are 0.30 Å and 0.26, respectively. The C(–H)···F distance between **1cu**$^{+}$ and BF_4^{-} is 3.17 Å. Solvent molecules are omitted for clarity. Atom color code: brown, pink, yellow, cyan, yellow green, orange, and blue refer to carbon, hydrogen, boron, nitrogen, fluorine, sulfur, and copper, respectively.

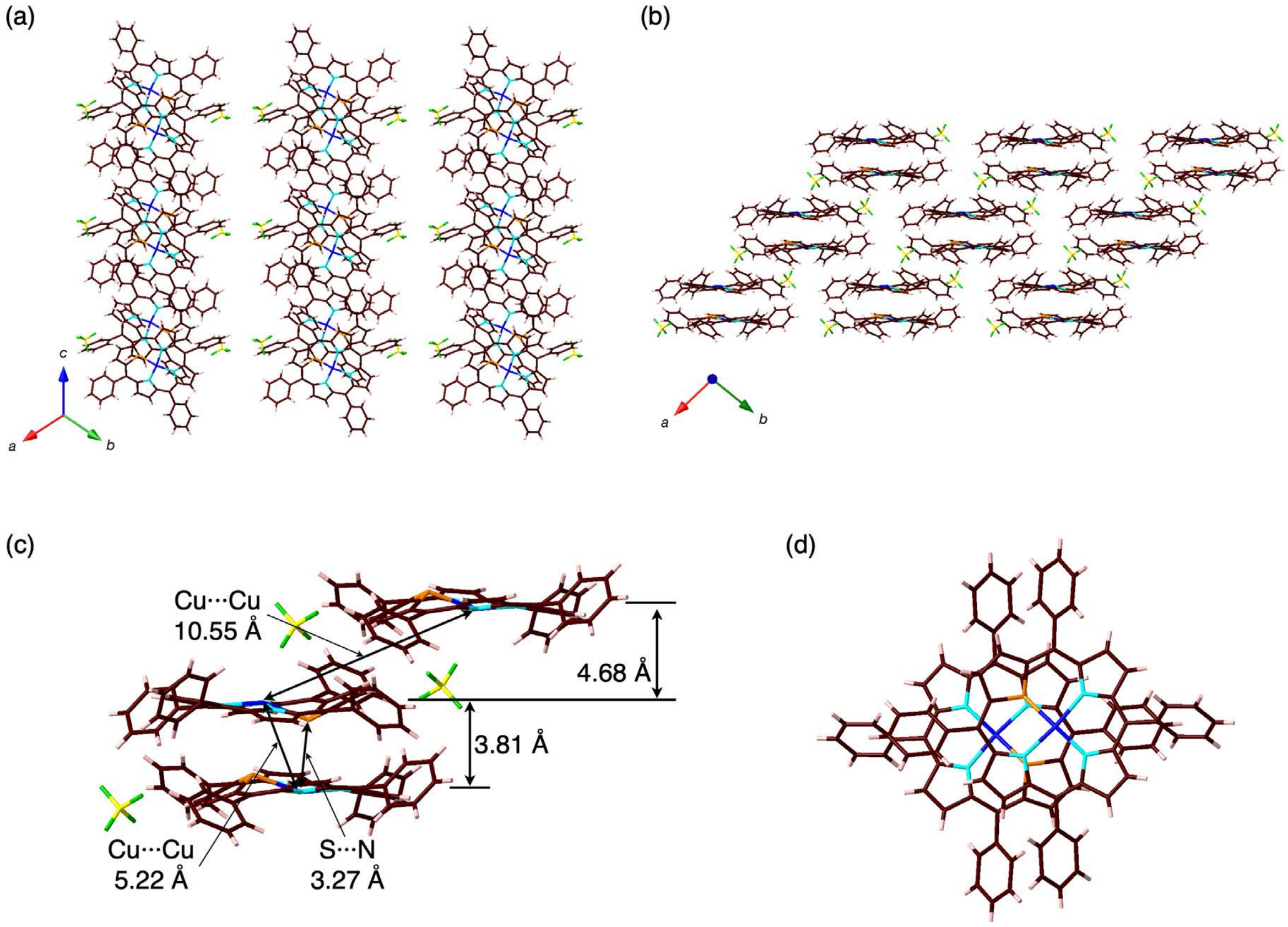

Fig. S15 Packing diagram (stacking assembly) of **1cu**$^+$-BF_4^- as (a) top and (b) side views and (c) enlarged side and (d) stacked dimer views. The stacking distances between two **1cu**$^+$ (core 25 atoms) and the Cu···Cu distances in the column are 3.81/4.68 and 5.22/10.55 Å, respectively. The S···N distance in two stacked **1cu**$^+$ units is 3.27 Å. Solvent molecules are omitted for clarity. Atom color code: brown, pink, yellow, cyan, yellow green, orange, and blue refer to carbon, hydrogen, boron, nitrogen, fluorine, sulfur, and copper, respectively.

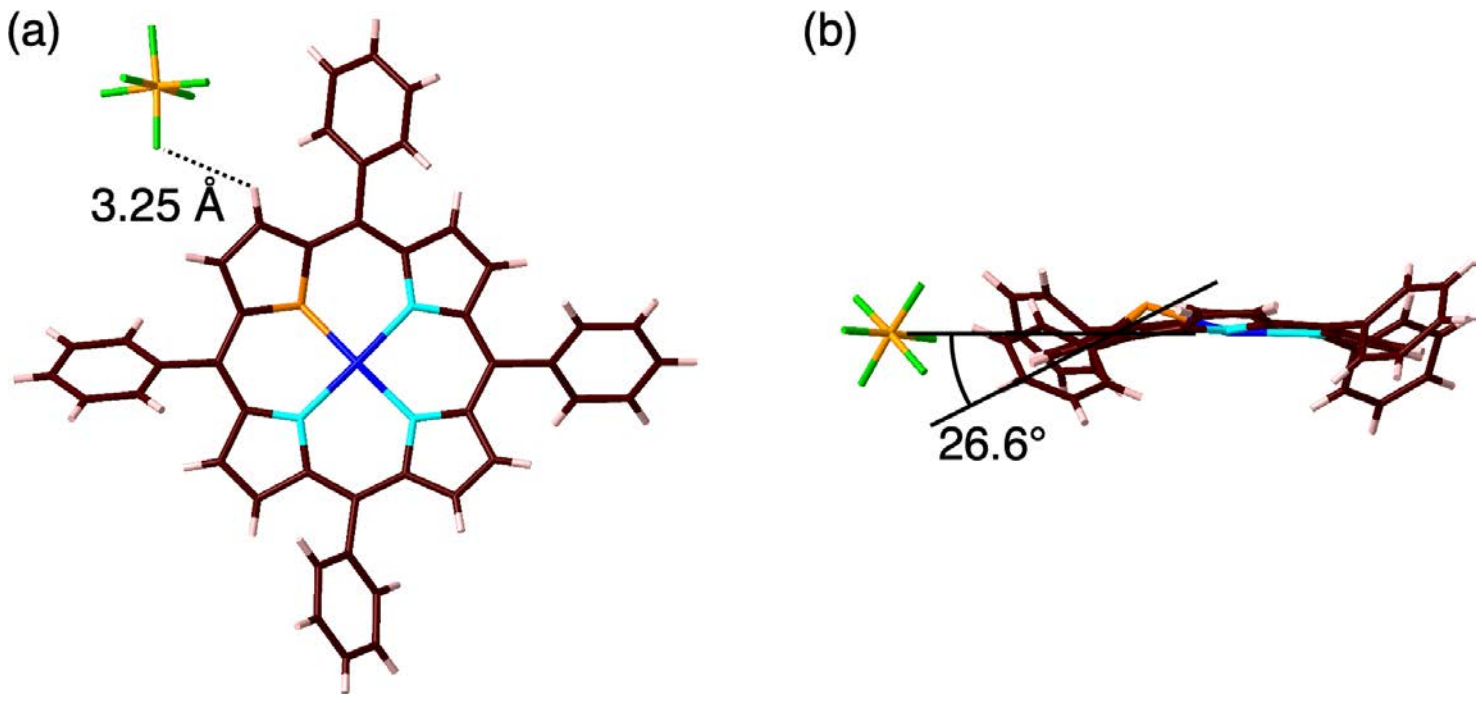

Fig. S16 Crystal structure of **1cu**$^+$-PF_6^- as (a) top and (b) side views. The dihedral angle between the thiophene plane and the core porphyrin plane (25 atoms) is 26.6°. Mean-plane deviation of the **1cu**$^+$ core part (25 atoms) and τ_4 value[S12] are 0.30 Å and 0.15, respectively. The C(–H)···F distance between **1cu**$^+$ and PF_6^- is 3.25 Å. Solvent molecules are omitted for clarity. Atom color code: brown, pink, blue, yellow green, light orange, orange, and blue refer to carbon, hydrogen, nitrogen, fluorine, phosphorus, sulfur, and copper, respectively.

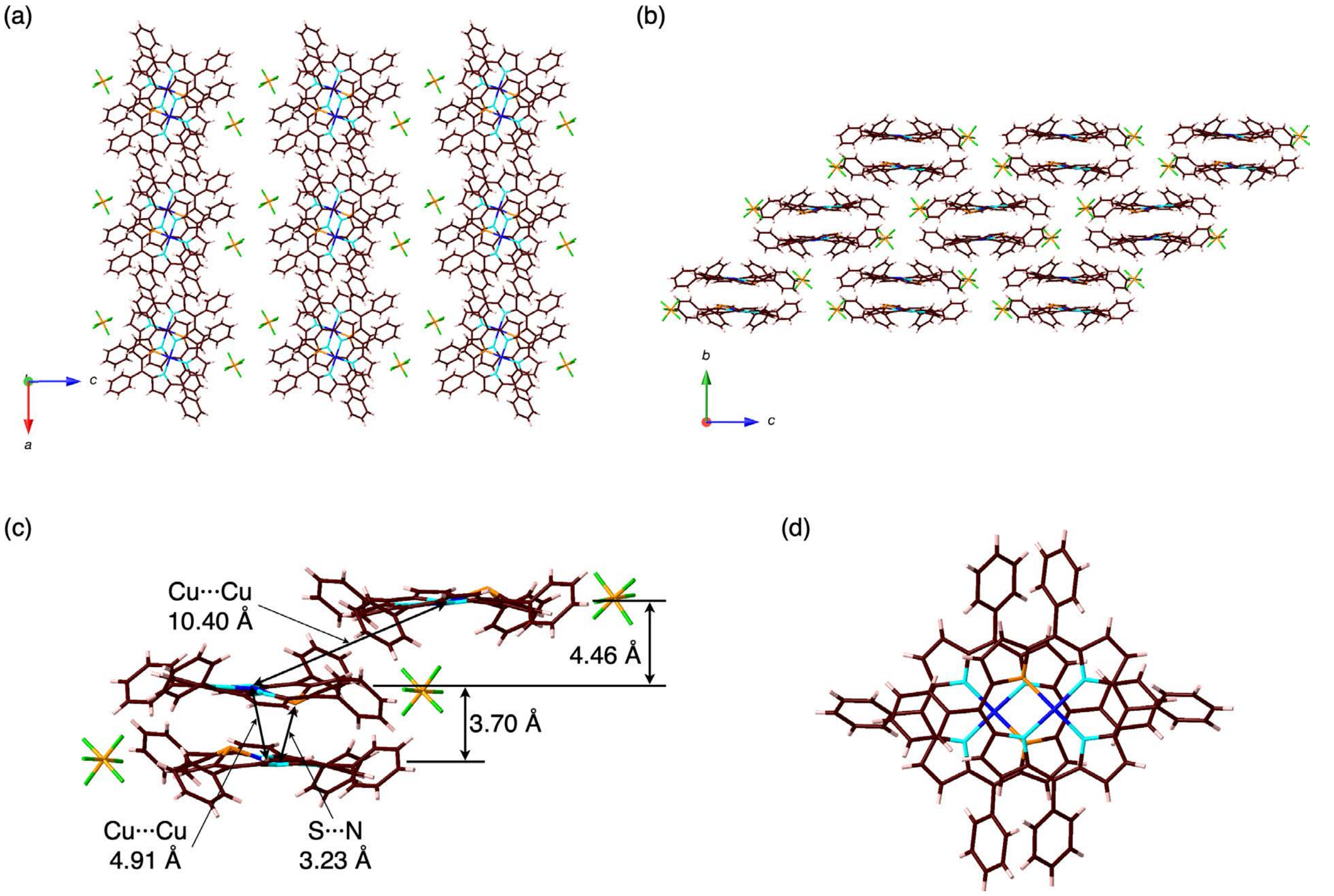

Fig. S17 Packing diagram (stacking assembly) of **1cu**$^+$-PF_6^- as (a) top and (b) side views and (c) enlarged side and (d) stacked dimer views. The stacking distances between two **1cu**$^+$ (core 25 atoms) and the Cu···Cu distances in the column are 3.70/4.46 and 4.91/10.40 Å, respectively. The S···N distance in two stacked **1cu**$^+$ units is 3.23 Å. Solvent molecules are omitted for clarity. Atom color code: brown, pink, blue, yellow green, light orange, orange, and blue refer to carbon, hydrogen, nitrogen, fluorine, phosphorus, sulfur, and copper, respectively.

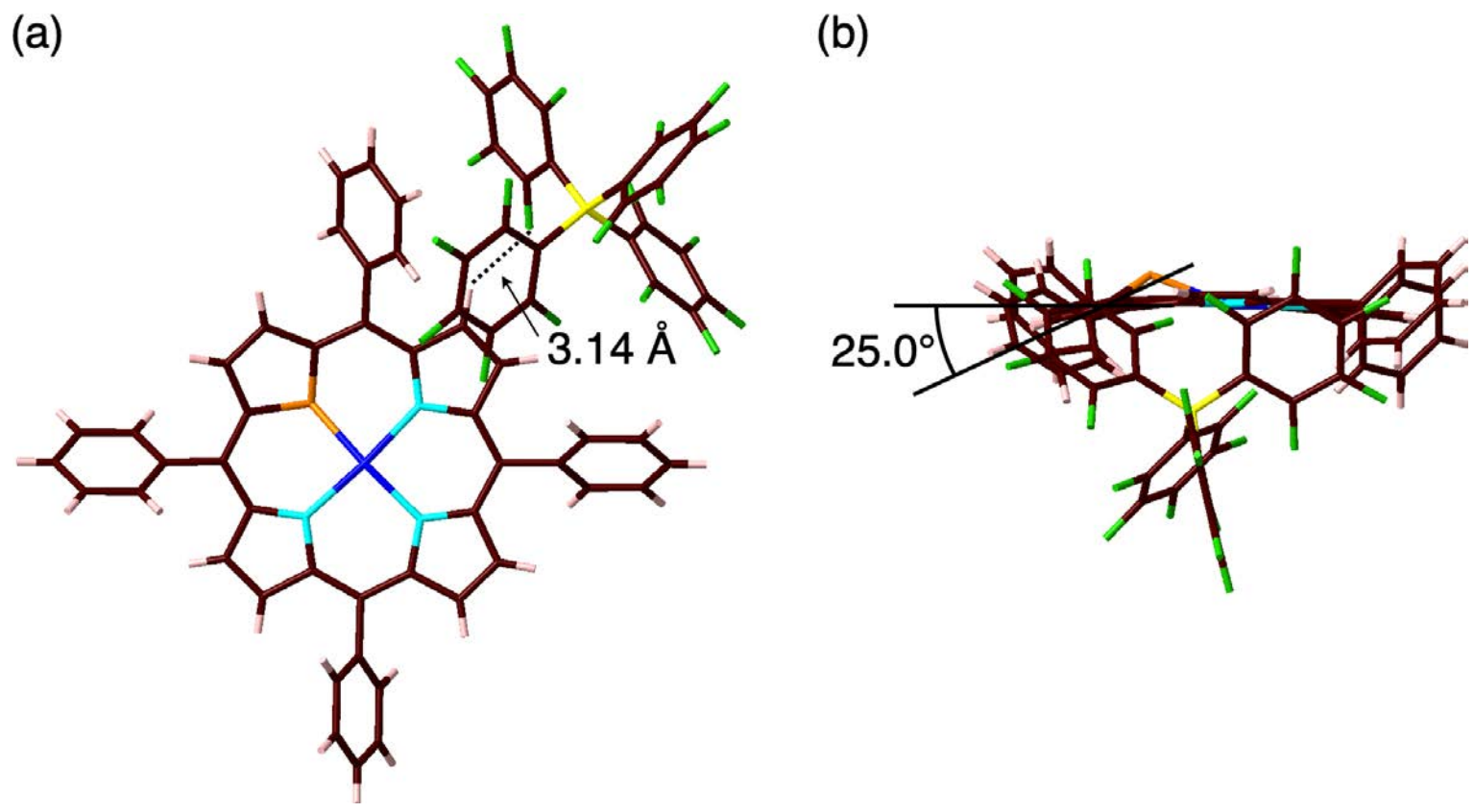

Fig. S18 Crystal structure of **1cu**$^+$-$B(C_6F_5)_4^-$ as (a) top and (b) side views. The dihedral angle between the thiophene plane and the core porphyrin plane (25 atoms) is 25.0°. Mean-plane deviation of the **1cu**$^+$ core part (25 atoms) and τ_4 value[S12] are 0.21 Å and 0.18, respectively. The C(–H)···F distance between **1cu**$^+$ and $B(C_6F_5)_4^-$ is 3.14 Å. Solvent molecules are omitted for clarity. Atom color code: brown, pink, yellow, cyan, yellow green, orange, and blue refer to carbon, hydrogen, boron, nitrogen, fluorine, sulfur, and copper, respectively.

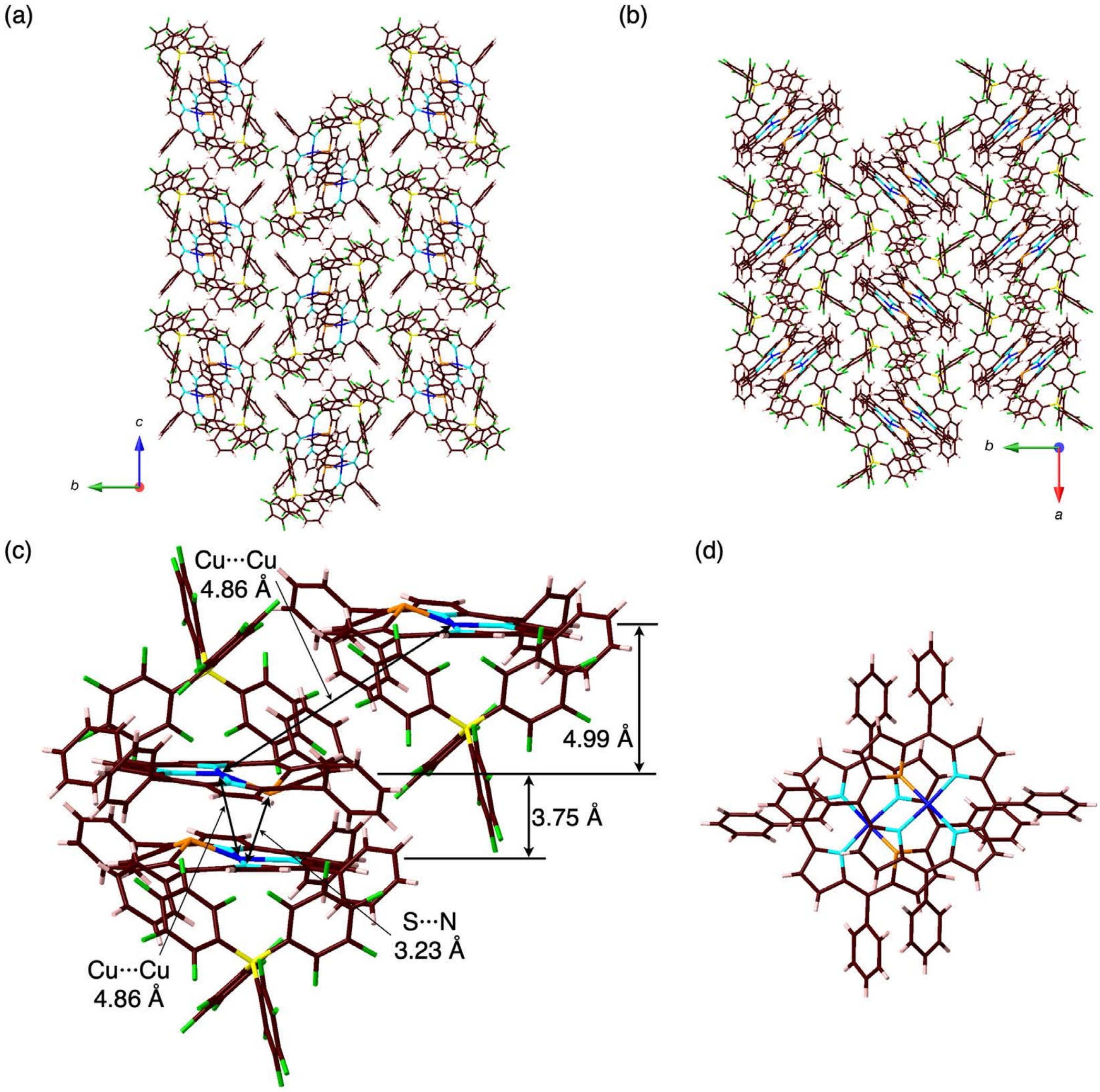

Fig. S19 Packing diagram (stacking assembly) of **1cu**$^+$- $B(C_6F_5)_4^-$ as (a) top and (b) side views and (c) an enlarged side and (d) stacked dimer views. The stacking distances between two **1cu**$^+$ (core 25 atoms) and the Cu···Cu distances in the column are 3.75/4.99 and 5.06/14.00 Å, respectively. The S···N distance in two stacked **1cu**$^+$ units is 3.27 Å. Solvent molecules are omitted for clarity. Atom color code: brown, pink, yellow, cyan, yellow green, orange, and blue refer to carbon, hydrogen, boron, nitrogen, fluorine, sulfur, and copper, respectively.

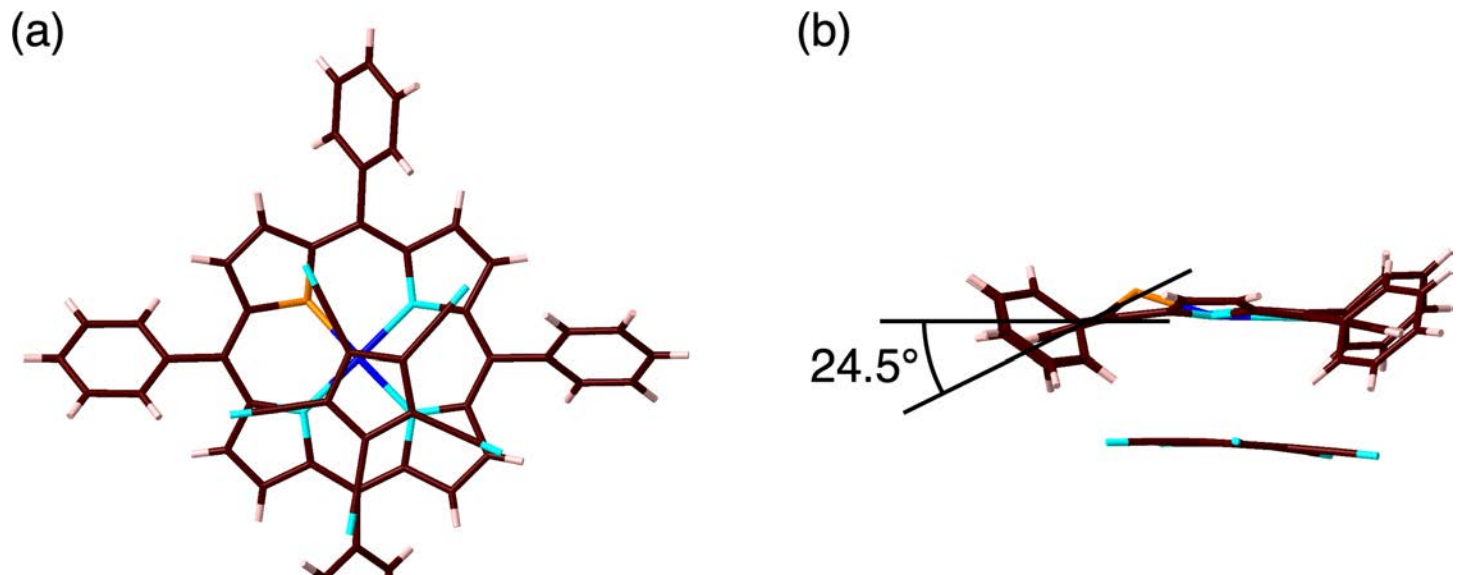

Fig. S20 Crystal structure of **1cu**$^+$-PCCp$^-$ as (a) top and (b) side views. The dihedral angle between the thiophene plane and the core porphyrin plane (25 atoms) is 24.5°. Mean-plane deviation of the **1cu**$^+$ core part (25 atoms) and τ_4 value[S12] are 0.26 Å and 0.13, respectively. Solvent molecules are omitted for clarity. Atom color code: brown, pink, cyan, yellow green, orange, and blue refer to carbon, hydrogen, nitrogen, fluorine, sulfur, and copper, respectively.

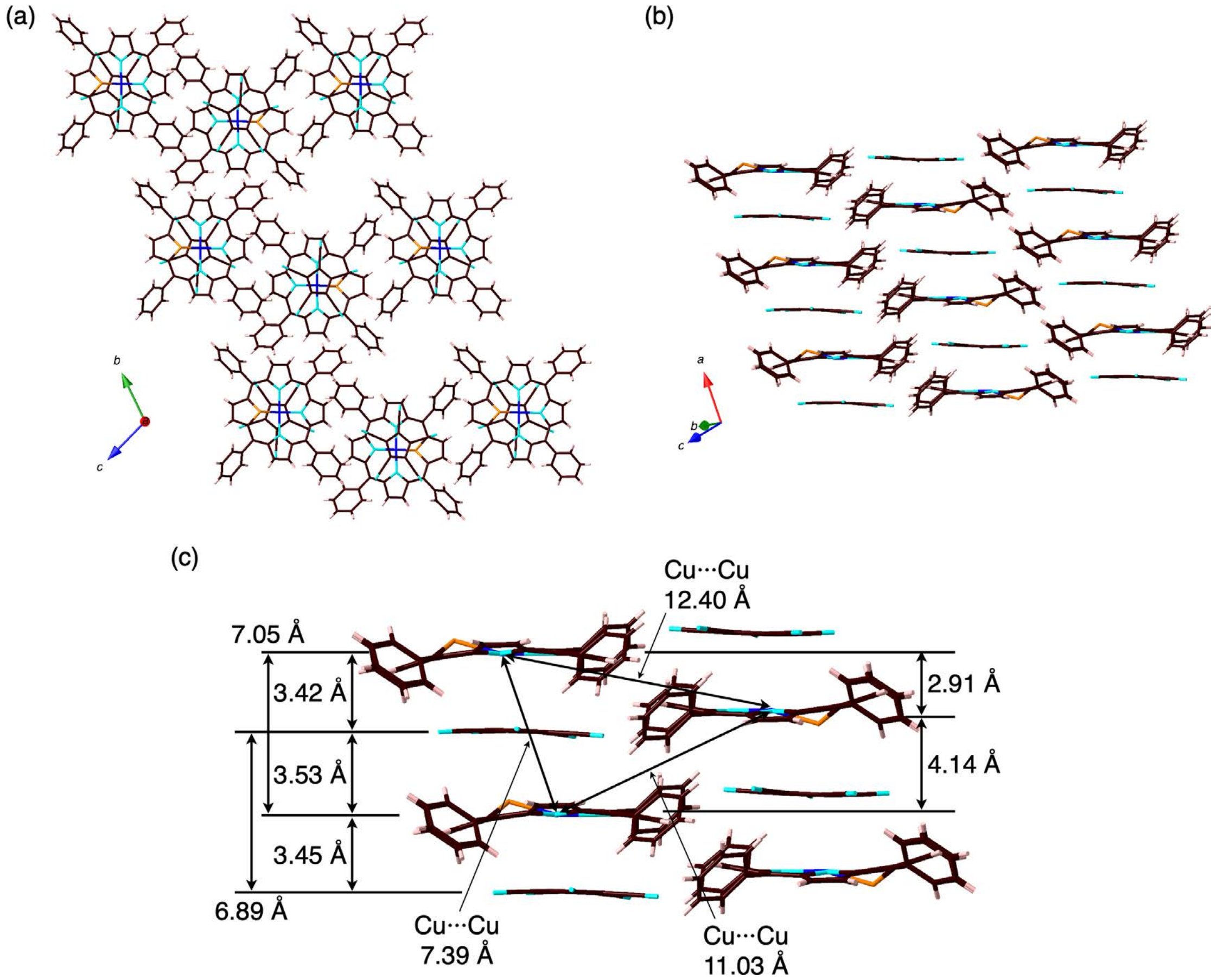

Fig. S21 Packing diagram (stacking assembly) of **1cu**$^+$-PCCp$^-$ as (a) top and (b) side views and (c) an enlarged side view. The stacking distances between **1cu**$^+$ (core 25 atoms) and PCCp$^-$ are 3.42 and 3.53 Å. The distances between two **1cu**$^+$, that between two PCCp$^-$, and the Cu···Cu distances in the column are 2.91/4.14/7.05, 6.89, and 7.39/11.03/12.40 Å, respectively. Solvent molecules are omitted for clarity. Atom color code: brown, pink, cyan, orange, and blue refer to carbon, hydrogen, nitrogen, sulfur, and copper, respectively.

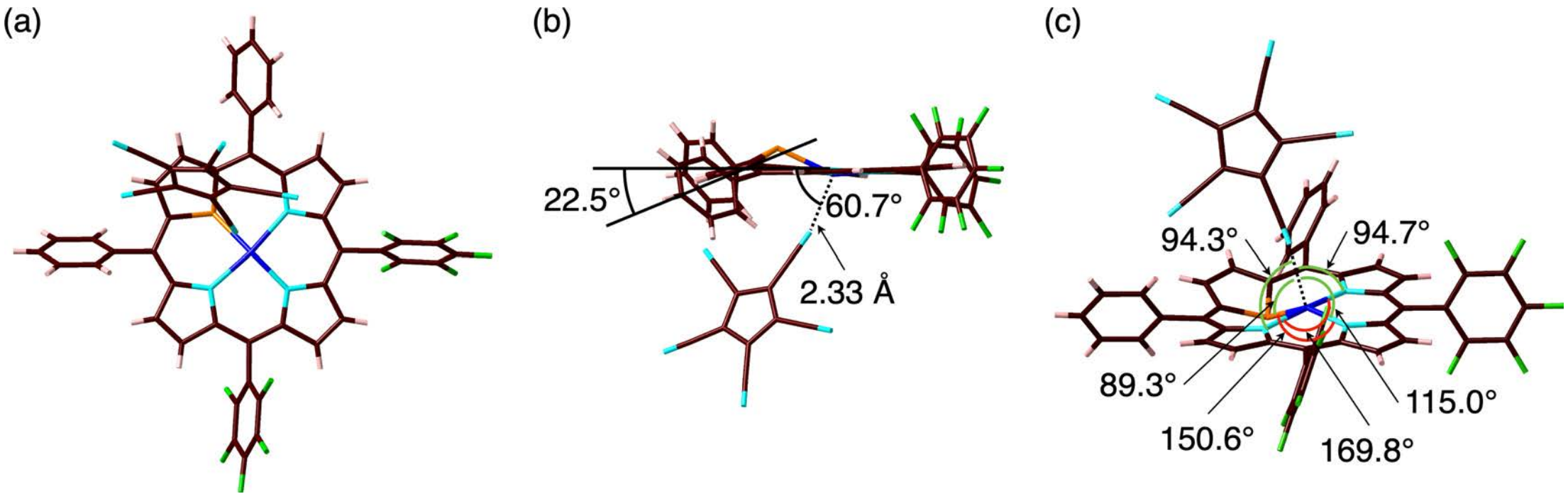

Fig. S22 Crystal structure of **2cu**$^+$-PCCp$^-$ as (a) top and (b) side views and (c) another view. The Cu–N distance is 2.33 Å and the angle of the line through Cu and PCCp$^-$ nitrogen to the core porphyrin plane (25 atoms) is 60.7°. The dihedral angle between the thiophene plane and the core porphyrin plane (25 atoms) is 22.5°. Mean-plane deviation of the **2cu**$^+$ core part (25 atoms), τ_4,[S12] and τ_5 values[S13] are 0.19 Å, 0.28, and 0.32, respectively. Solvent molecules are omitted for clarity. Atom color code: brown, pink, cyan, yellow green, orange, and blue refer to carbon, hydrogen, nitrogen, fluorine, sulfur, and copper, respectively. The τ_4 value was calculated for the basal tetracoordinated environment around Cu (excluding the axial Cl$^-$ ligand), whereas the τ_5 value was calculated for the pentacoordinated geometry including the axial ligand.

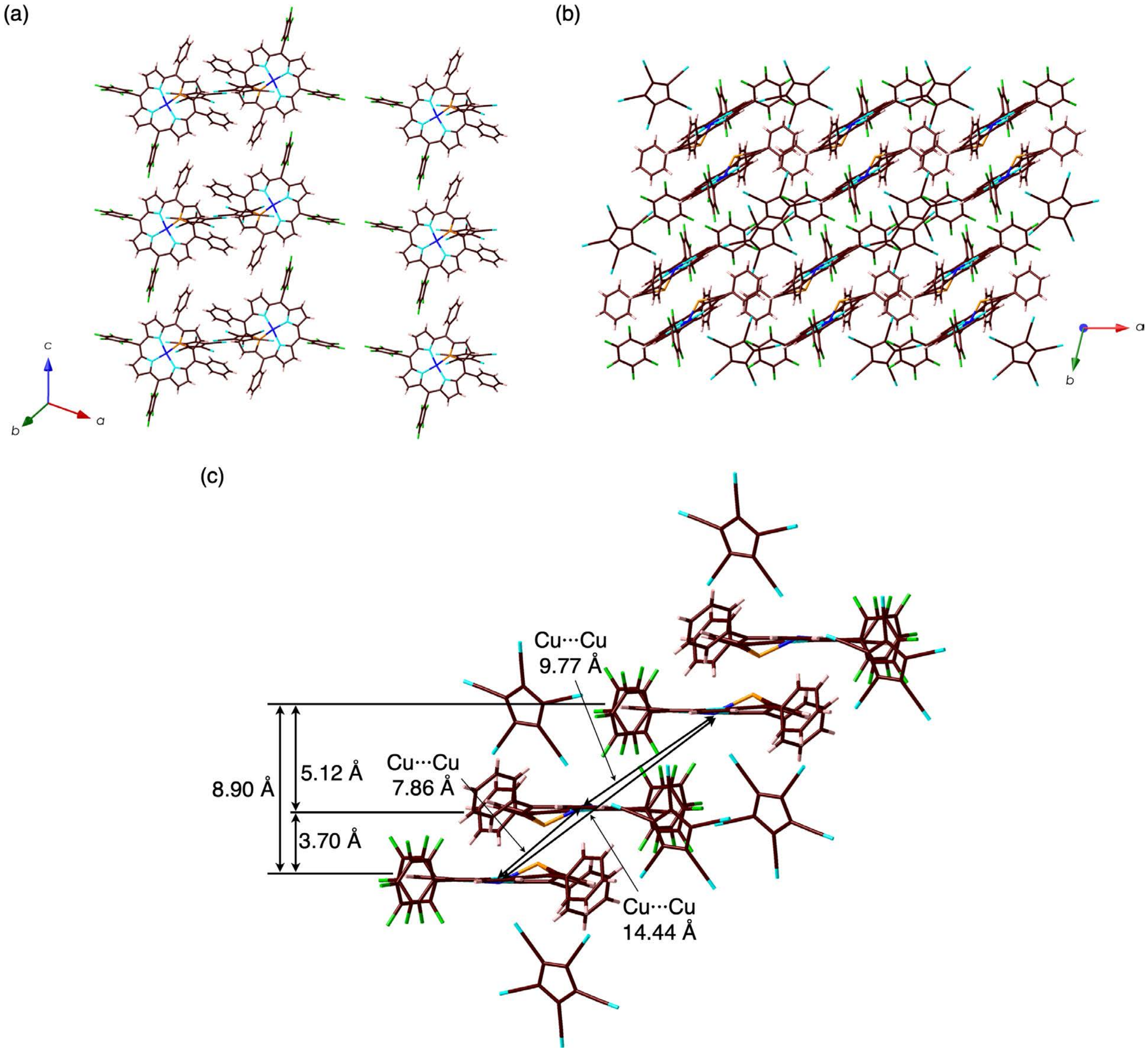

Fig. S23 Packing diagram (stacking assembly) of **2cu**$^{+}$-PCCp^{-} as (a) top and (b) side views and (c) an enlarged side view. The distances between two **2cu**$^{+}$ (core 25 atoms) and the Cu···Cu distances are 3.70/5.12/8.90 and 7.86/9.77/14.44 Å, respectively. Solvent molecules are omitted for clarity. Atom color code: brown, pink, cyan, yellow green, orange, and blue refer to carbon, hydrogen, nitrogen, fluorine, sulfur, and copper, respectively.

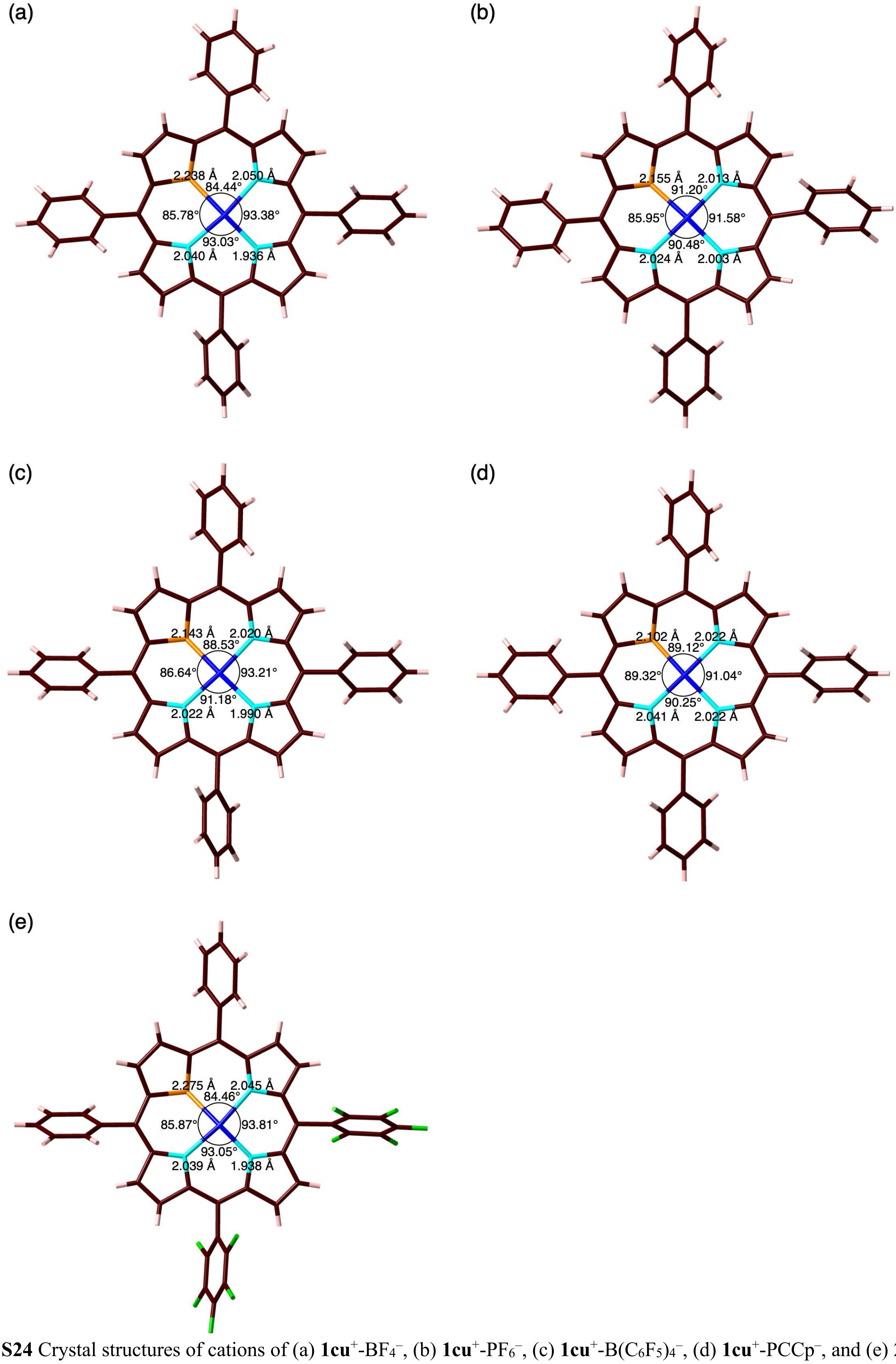

Fig. S24 Crystal structures of cations of (a) **1cu**$^+$-BF_4^-, (b) **1cu**$^+$-PF_6^-, (c) **1cu**$^+$-$B(C_6F_5)_4^-$, (d) **1cu**$^+$-PCCp$^-$, and (e) **2cu**$^+$-PCCp$^-$. Atom color code: brown, pink, cyan, yellow green, orange, and blue refer to carbon, hydrogen, nitrogen, fluorine, sulfur, and copper, respectively.

Table S2 The τ_4 and S(G) values obtained from the SHAPE program[S14] for the **1cu**$^+$ ion pairs, in which **1cu**$^+$ exhibits tetracoordinated geometries. SP-4, T-4, SS-4, and vTBPY-4 refer to square planar, tetrahedral, seesaw, and vacant trigonal bipyramidal geometries, respectively. As shown in the table, **1cu**$^+$ preferentially adopts SP-4 geometry in all ion pair. The S(G) value of **1cu**$^+$-BF_4^- (1.057) is the largest among these ion pairs, indicating that **1cu**$^+$ in **1cu**$^+$-BF_4^- adopts the most distorted geometry. These results are consistent with the τ_4 values.

	τ_4	S(G)			
		SP-4 (D_{4h})	T-4 (T_d)	SS-4 (C_{2v})	vTBPY-4 (C_{3v})
**1cu**$^+$-BF_4^-	0.26	1.057	26.943	12.983	27.660
**1cu**$^+$-PF_6^-	0.15	0.628	27.559	14.261	29.059
**1cu**$^+$-$B(C_6F_5)_4^-$	0.18	0.927	25.827	12.886	27.073
**1cu**$^+$-PCCp$^-$	0.13	0.619	26.98	13.789	28.509

Table S3 The τ_5 and S(G) values obtained from the SHAPE program[S14] for **2cu**$^+$-PCCp$^-$, which exhibits a pentacoordinated geometry. SPY-5, PP-5, vOC-5, and TBPY-5 refer to spherical square pyramidal, pentagonal, vacant octahedral, and trigonal bipyramidal geometries, respectively.

	τ_5	S(G)			
		SPY-5 (C_{4v})	PP-5 (D_{5h})	vOC-5 (C_{4v})	TBPY-5 (D_{3h})
**2cu**$^+$-PCCp$^-$	0.32	1.284	31.494	2.272	2.611

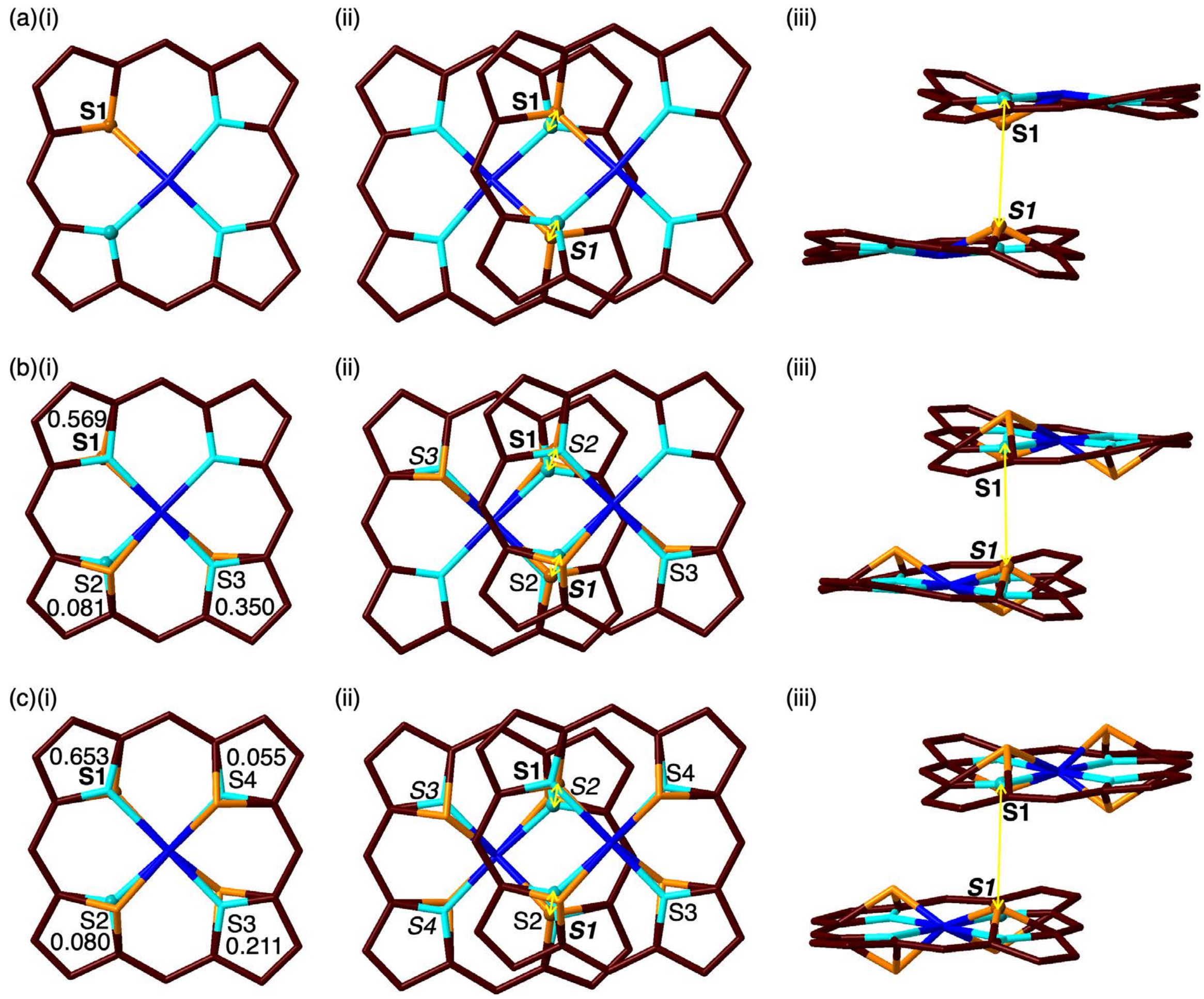

Fig. S25 Crystal structures of **1cu**$^+$ (core 25 atoms) of (a) **1cu**$^+$-BF_4^-, (b) **1cu**$^+$-PF_6^-, and (c) **1cu**$^+$-$B(C_6F_5)_4^-$: (i) monomer as top view and stacked dimer as (ii) top and (iii) side views showing the positional disorder of the thiophene unit. The bold S1 atoms denote the major sites, and the roman and italic labels indicate the upper and lower cations, respectively. Atom color code: brown, cyan, orange, and blue refer to carbon, nitrogen, sulfur, and copper, respectively.

Table S4 Estimated probabilities (%) for the combinations of disordered structures (S1–3) in the stacked dimers of **1cu**$^{+}$-PF_6^{-} (Fig. S25b). The possibilities were obtained from multiplicative combinations of crystallographic occupancies. The true populations may differ depending on the relative stability of each combination because the values do not include relative stability factors.

combinations of disordered structures	number of chalcogen bonds	possibility (%)
S1-S1	2	32.42
S1-S2, S2-S1	0	9.21
S1-S3, S3-S1	1	39.83
S2-S2	0	0.65
S2-S3, S3-S2	0	5.65
S3-S3	0	12.23

Table S5 Estimated probabilities (%) for the combinations of disordered structures (S1–4) in the stacked dimers of **1cu**$^{+}$-$B(C_6F_5)_4^{-}$ (Fig. S25c). The possibilities were obtained from multiplicative combinations of crystallographic occupancies. The true populations may differ depending on the relative stability of each combination because the values do not include relative stability factors.

combinations of disordered structures	number of chalcogen bonds	possibility (%)
S1-S1	2	42.64
S1-S2, S2-S1	0	10.45
S1-S3, S3-S1	1	27.56
S1-S4, S4-S1	1	7.18
S2-S2	0	0.64
S2-S3, S3-S2	0	3.38
S2-S4, S4-S2	0	0.88
S3-S3	0	4.45
S3-S4, S4-S3	0	2.32
S4-S4	0	0.30

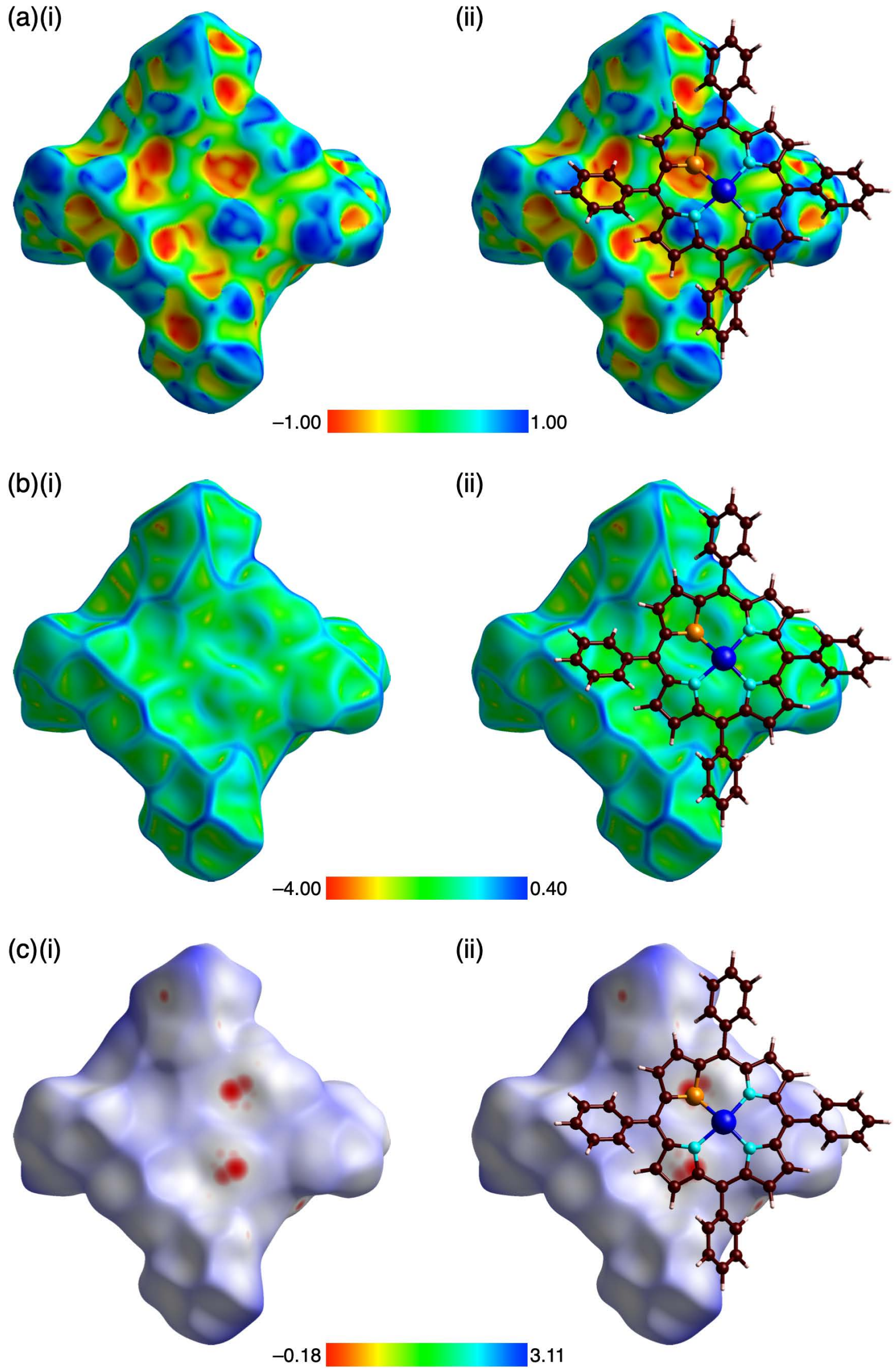

Fig. S26 Hirshfeld surface[S15,16] of **1cu**$^+$ in the crystal structure of **1cu**$^+$-BF_4^- mapped over (a) shape-index property, (b) curvedness property, and (c) d_{norm} property: (i) only surface and (ii) surface with a ball-and-stick model of the neighboring **1cu**$^+$. Shape index is a qualitative measure of shape and is sensitive to subtle changes in surface shape, particularly in a flat region by differing by sign represent complementary bumps (blue) and hollows (red), whereas curvedness is a function of the root-mean-square curvature of the surface, and maps of curvedness typically show large regions of green (relatively flat) separated by dark blue edges (large positive curvature). The flat region on the curvedness surface suggested the characteristic mapping pattern for stacking in dimeric **1cu**$^+$. Atom color code: brown, pink, cyan, orange, and blue refer to carbon, hydrogen, nitrogen, sulfur, and copper, respectively.

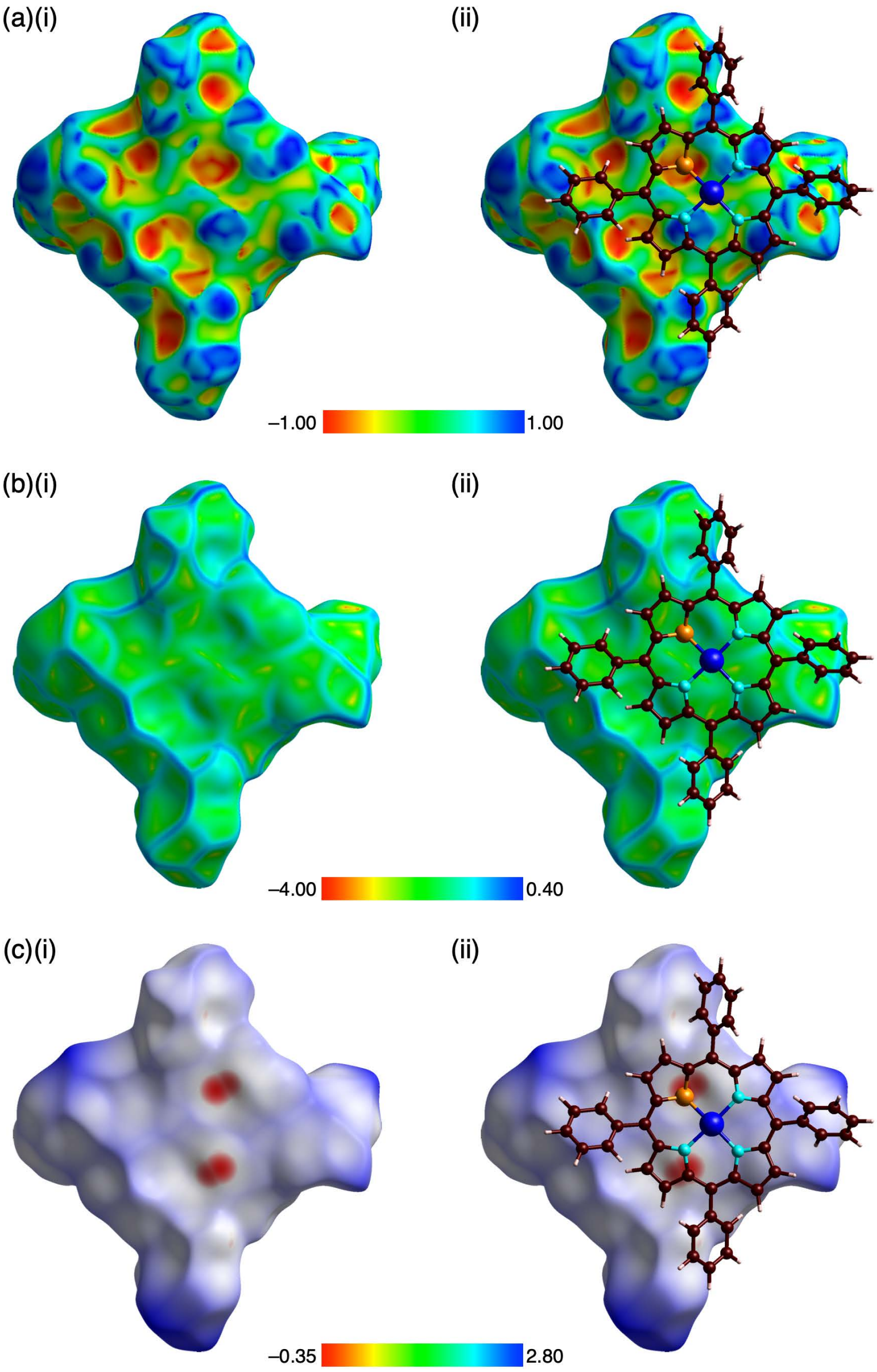

Fig. S27 Hirshfeld surface[S15,16] of **1cu**$^+$ in the crystal structure of **1cu**$^+$-PF_6^- mapped over (a) shape-index property, (b) curvedness property, and (c) d_{norm} property: (i) only surface and (ii) surface with a ball-and-stick model of the neighboring **1cu**$^+$. Shape index is a qualitative measure of shape and is sensitive to subtle changes in surface shape, particularly in a flat region by differing by sign represent complementary bumps (blue) and hollows (red), whereas curvedness is a function of the root-mean-square curvature of the surface, and maps of curvedness typically show large regions of green (relatively flat) separated by dark blue edges (large positive curvature). The flat region on the curvedness surface suggested the characteristic mapping pattern for stacking in dimeric **1cu**$^+$. Atom color code: brown, pink, cyan, orange, and blue refer to carbon, hydrogen, nitrogen, sulfur, and copper, respectively.

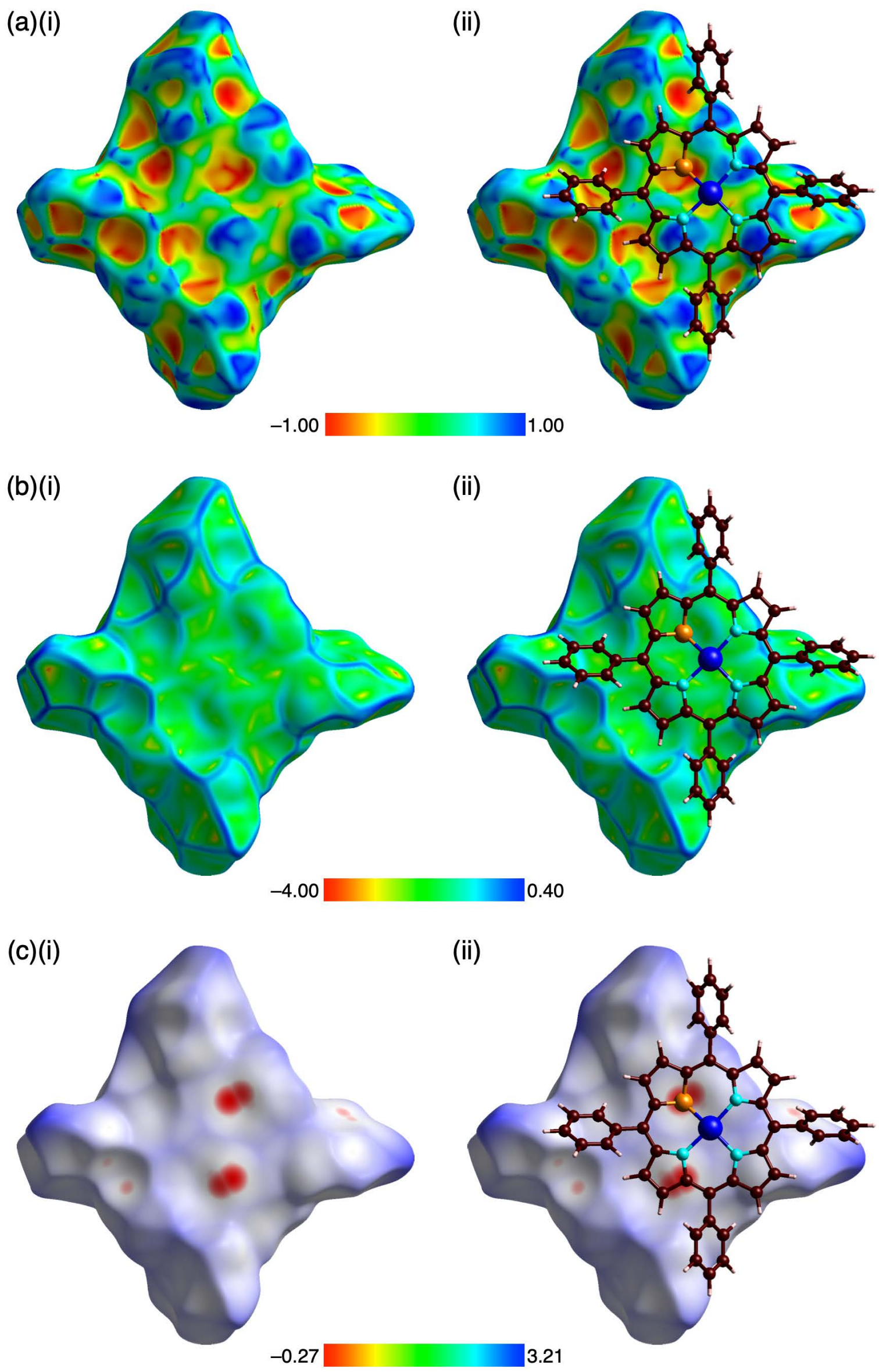

Fig. S28 Hirshfeld surface[S15,16] of **1cu**$^{+}$ in the crystal structure of **1cu**$^{+}$-$B(C_6F_5)_4^{-}$ mapped over (a) shape-index property, (b) curvedness property, and (c) d_{norm} property: (i) only surface and (ii) surface with a ball-and-stick model of the neighboring **1cu**$^{+}$. Shape index is a qualitative measure of shape and is sensitive to subtle changes in surface shape, particularly in a flat region by differing by sign represent complementary bumps (blue) and hollows (red), whereas curvedness is a function of the root-mean-square curvature of the surface, and maps of curvedness typically show large regions of green (relatively flat) separated by dark blue edges (large positive curvature). The flat region on the curvedness surface suggested the characteristic mapping pattern for stacking in dimeric **1cu**$^{+}$. Atom color code: brown, pink, cyan, orange, and blue refer to carbon, hydrogen, nitrogen, sulfur, and copper, respectively.

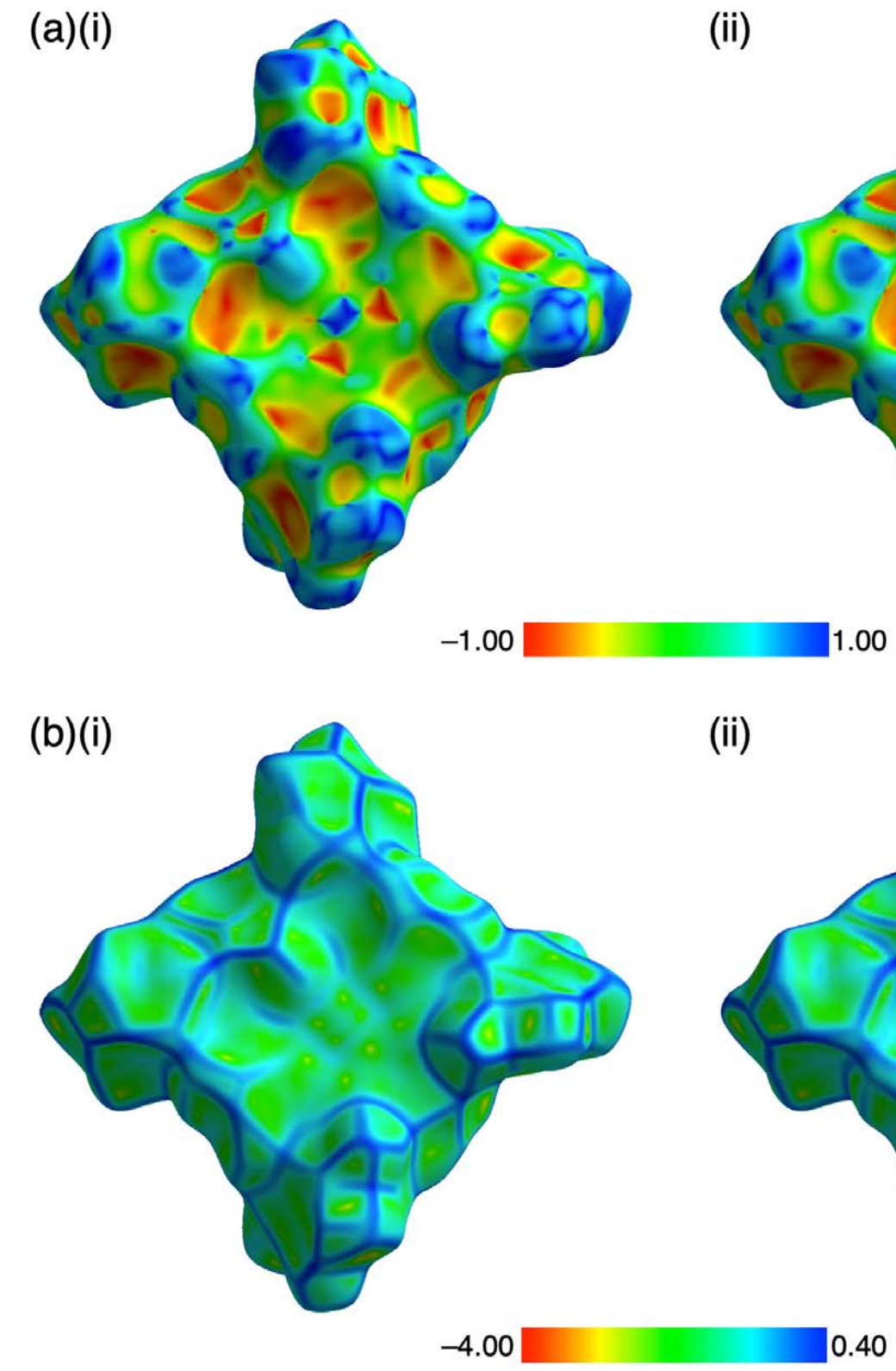

Fig. S29 Hirshfeld surface[S15,16] of **1cu**$^+$ whose sulfur atom is far from the PCCp$^-$ in the crystal structure of **1cu**$^+$-PCCp$^-$ (a major disordered structure) mapped over (a) shape-index property and (b) curvedness property: (i) only surface and (ii) surface with a ball-and-stick model of the neighboring PCCp$^-$. Shape index is a qualitative measure of shape and is sensitive to subtle changes in surface shape, particularly in a flat region by differing by sign represent complementary bumps (blue) and hollows (red), whereas curvedness is a function of the root-mean-square curvature of the surface, and maps of curvedness typically show large regions of green (relatively flat) separated by dark blue edges (large positive curvature). The surfaces of **1cu**$^+$ showed the red and blue triangles arranged in bow-tie shapes on the shape-index surface and flat region on the curvedness surface, indicating the characteristic mapping pattern for $^i\pi$–$^i\pi$ stacking.[S17] Atom color code: brown and cyan refer to carbon and nitrogen, respectively.

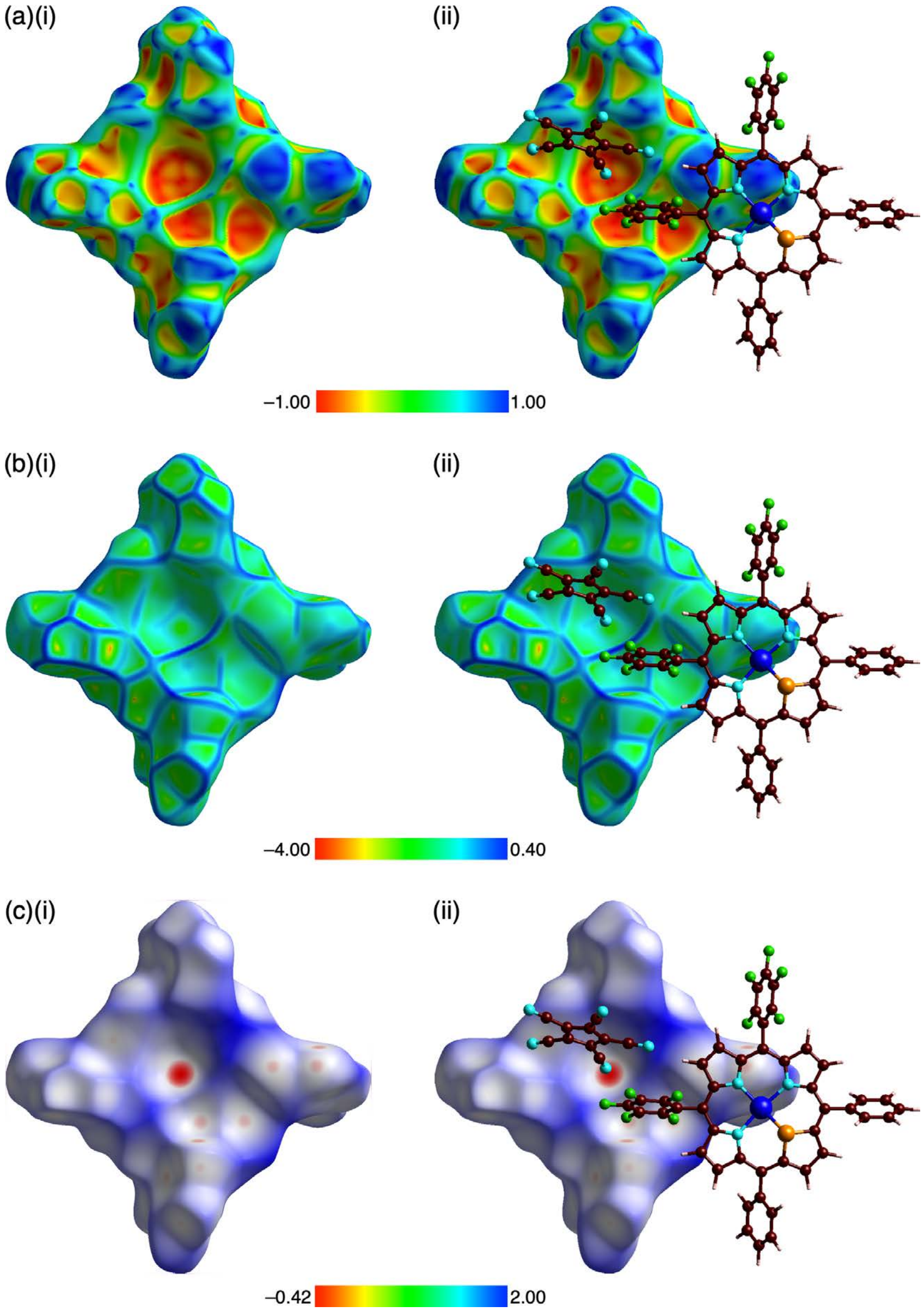

Fig. S30 Hirshfeld surface[S15,16] of **2cu**$^+$ whose sulfur atom is far from the PCCp$^–$ in the crystal structure of **2cu**$^+$-PCCp$^–$ mapped over (a) shape-index property, (b) curvedness property, and (c) d_{norm} property: (i) only surface and (ii) surface with a ball-and-stick model of the neighboring **2cu**$^+$ and PCCp$^–$. Shape index is a qualitative measure of shape and is sensitive to subtle changes in surface shape, particularly in a flat region by differing by sign represent complementary bumps (blue) and hollows (red), whereas curvedness is a function of the root-mean-square curvature of the surface, and maps of curvedness typically show large regions of green (relatively flat) separated by dark blue edges (large positive curvature). The surfaces of **2cu**$^+$ showed the red regions on the shape-index and d_{norm} surfaces, indicating axial coordination between Cu and PCCp$^–$ nitrogen and C–F···π interactions between **2cu**$^+$. Atom color code: brown, pink, cyan, yellow green, orange, and blue refer to carbon, hydrogen, nitrogen, fluorine, sulfur, and copper, respectively.

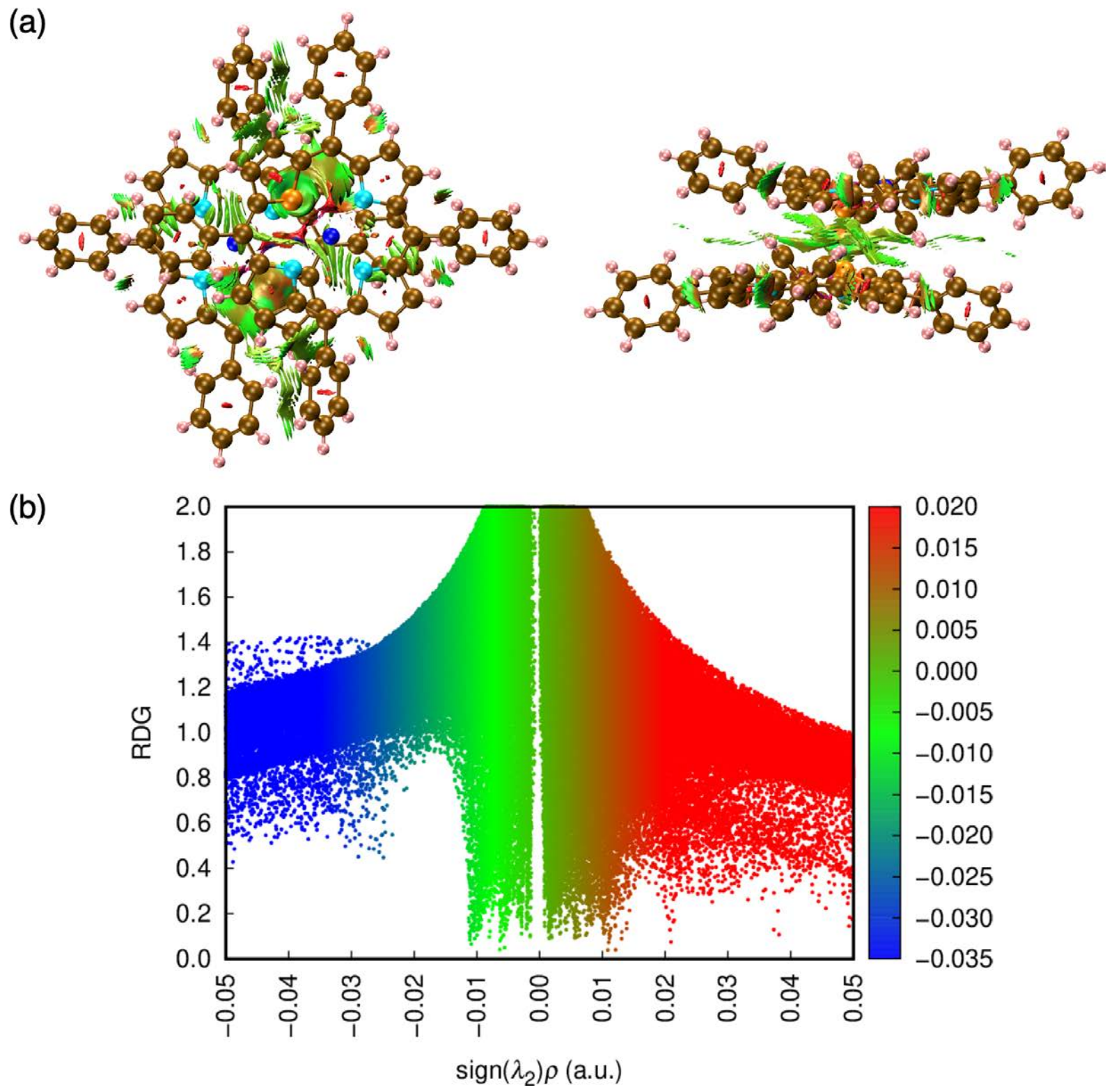

Fig. S31 NCI analysis[S18,19] of dimeric **1cu**$^{+}$ in the crystal structure of **1cu**$^{+}$-BF_4^{-}: (a) RDG isosurface and (b) RDG vs *sign*(λ_2)ρ plot. Weakly attractive regions corresponding to chalcogen bonding and $^{i}\pi$–$^{i}\pi$ interactions are observed. Atom color code: brown, pink, cyan, orange, and blue refer to carbon, hydrogen, nitrogen, sulfur, and copper, respectively.

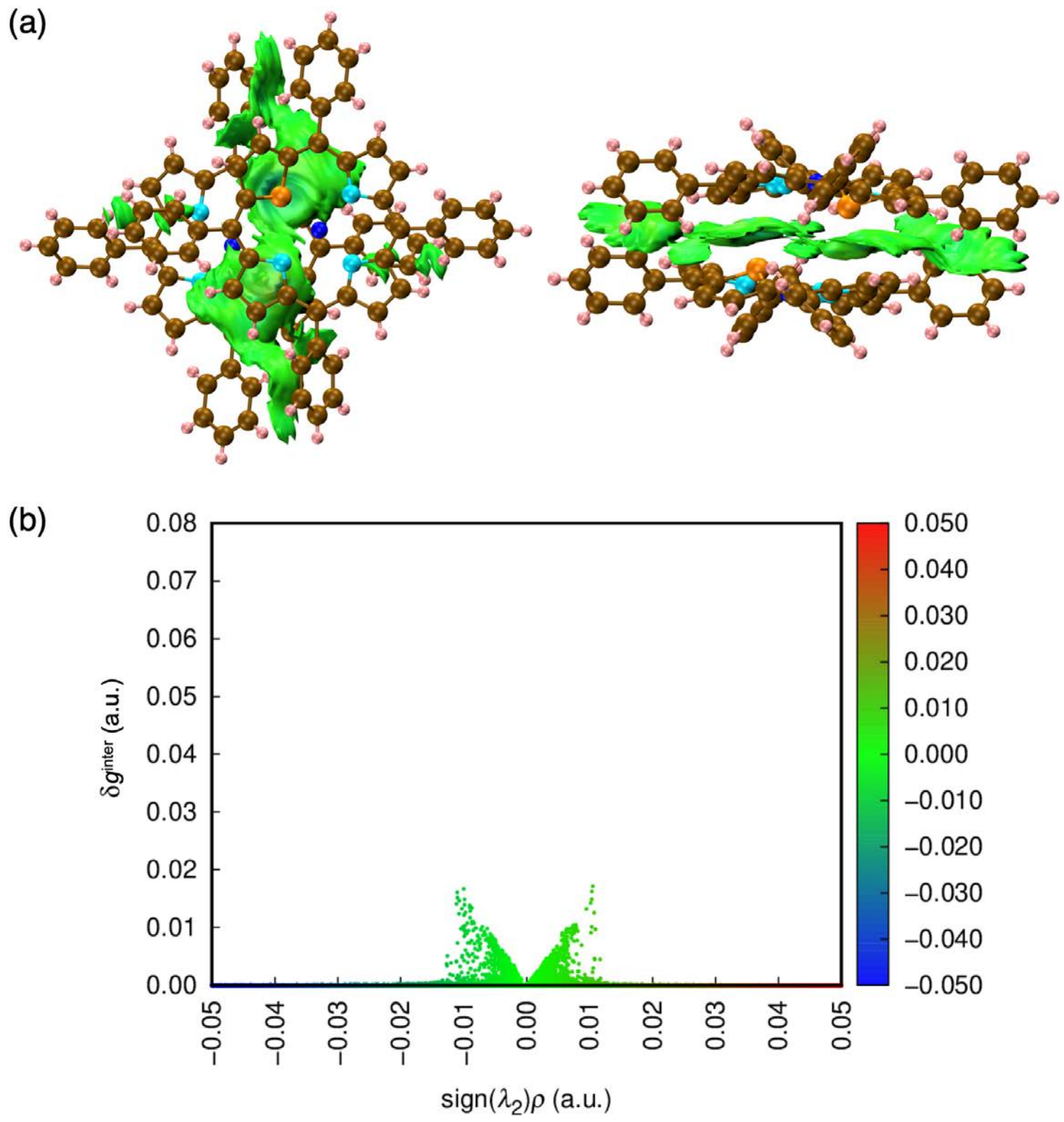

Fig. S32 NCI analysis[S18,19] of dimeric **1cu**$^{+}$ in the crystal structure of **1cu**$^{+}$-BF_4^{-}: (a) IGMH[S20] isosurface and (b) δg^{inter} vs *sign*(λ_2)ρ plot. Weakly attractive regions corresponding to chalcogen bonding and $^{i}\pi$–$^{i}\pi$ interactions are observed. Atom color code: brown, pink, cyan, orange, and blue refer to carbon, hydrogen, nitrogen, sulfur, and copper, respectively.

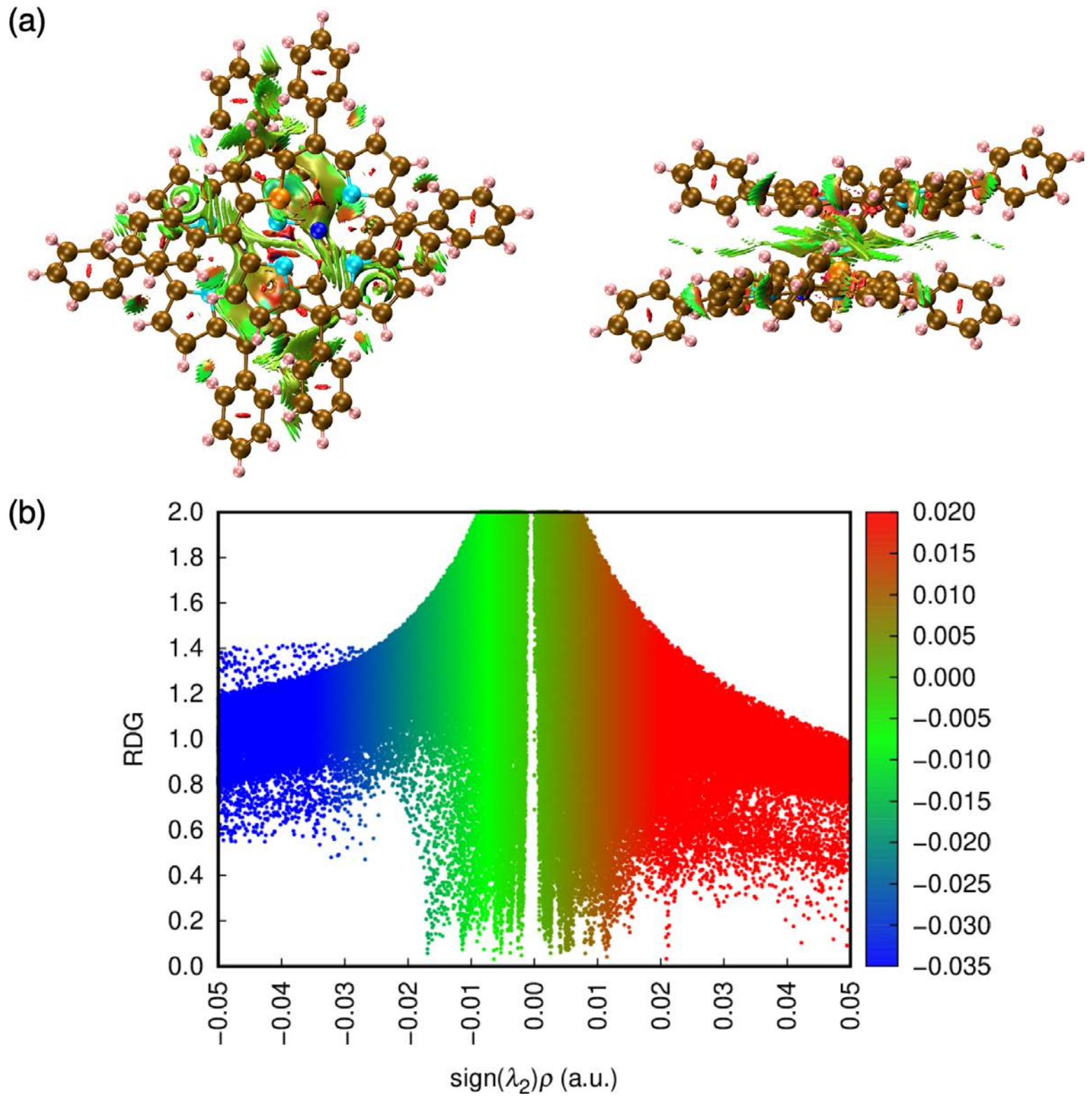

Fig. S33 NCI analysis[S18,19] of dimeric **1cu**$^+$ in the crystal structure of **1cu**$^+$-PF_6^-: (i) RDG isosurface and (ii) RDG vs *sign*(λ_2)ρ plot. Weakly attractive regions corresponding to chalcogen bonding and $^i\pi$–$^i\pi$ interactions were observed. Atom color code: brown, pink, cyan, orange, and blue refer to carbon, hydrogen, nitrogen, sulfur, and copper, respectively.

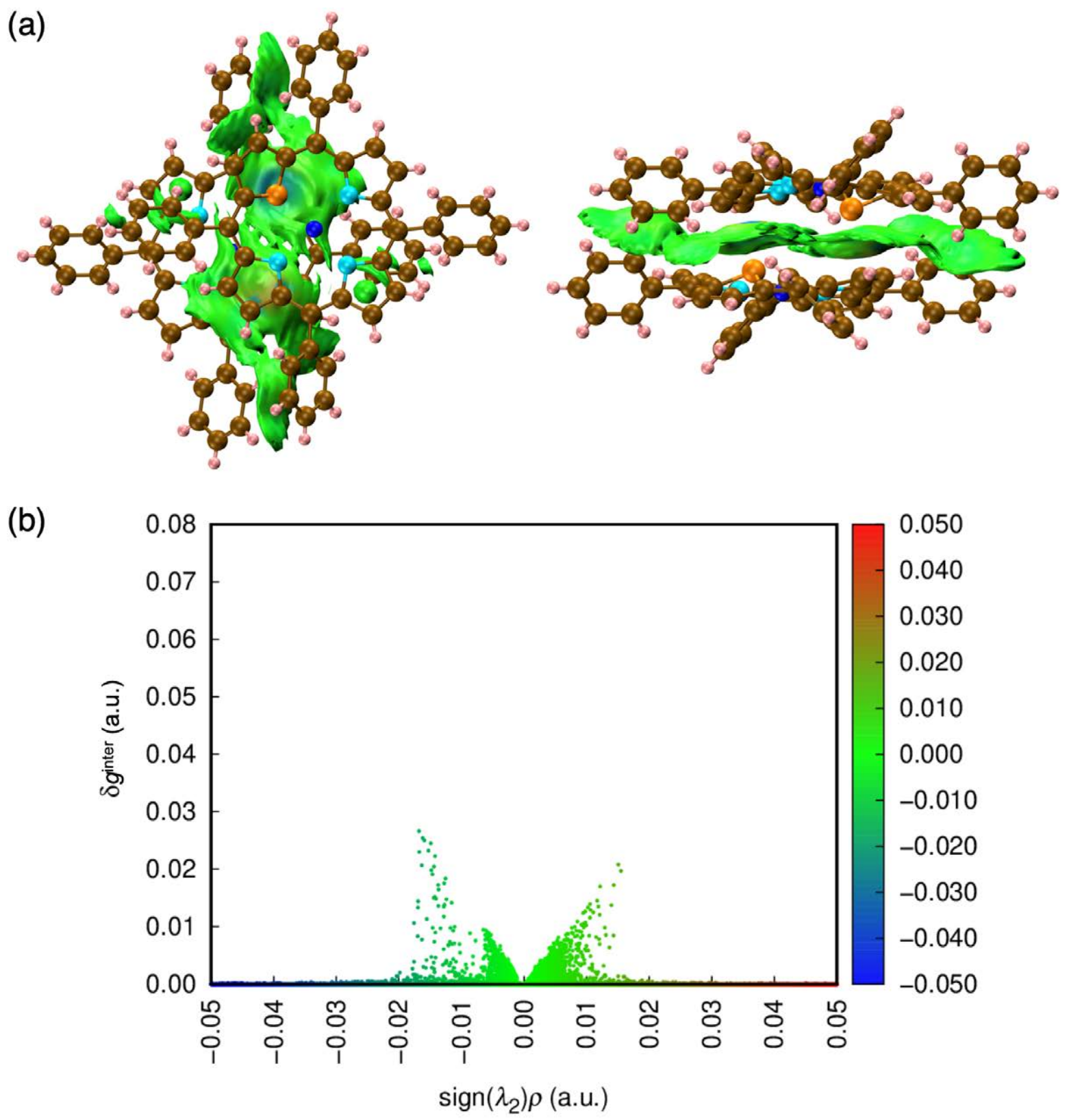

Fig. S34 NCI analysis[S18,19] of dimeric **1cu**$^+$ in the crystal structure of **1cu**$^+$-PF_6^-: (a) IGMH[S20] isosurface and (b) δg^{inter} vs *sign*(λ_2)ρ plot. Weakly attractive regions corresponding to chalcogen bonding and $^i\pi$–$^i\pi$ interactions are observed. Atom color code: brown, pink, cyan, orange, and blue refer to carbon, hydrogen, nitrogen, sulfur, and copper, respectively.

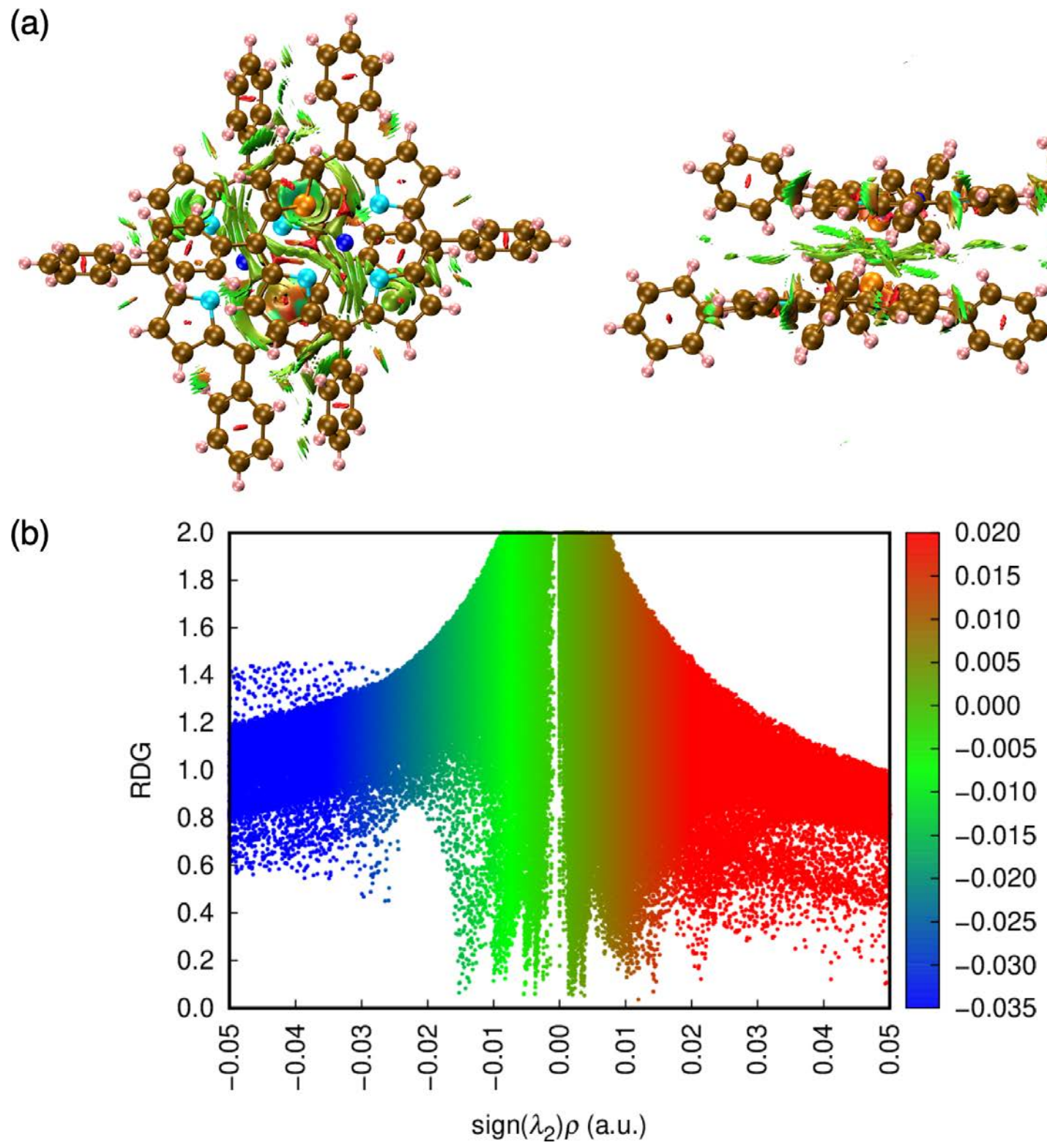

Fig. S35 NCI analysis[S18,19] of dimeric **1cu**$^+$ in the crystal structure of **1cu**$^+$-$B(C_6F_5)_4^-$: (a) RDG isosurface and (b) RDG vs *sign*(λ_2)ρ plot. Weakly attractive regions corresponding to chalcogen bonding and $^i\pi$–$^i\pi$ interactions were observed. Atom color code: brown, pink, cyan, orange, and blue refer to carbon, hydrogen, nitrogen, sulfur, and copper, respectively.

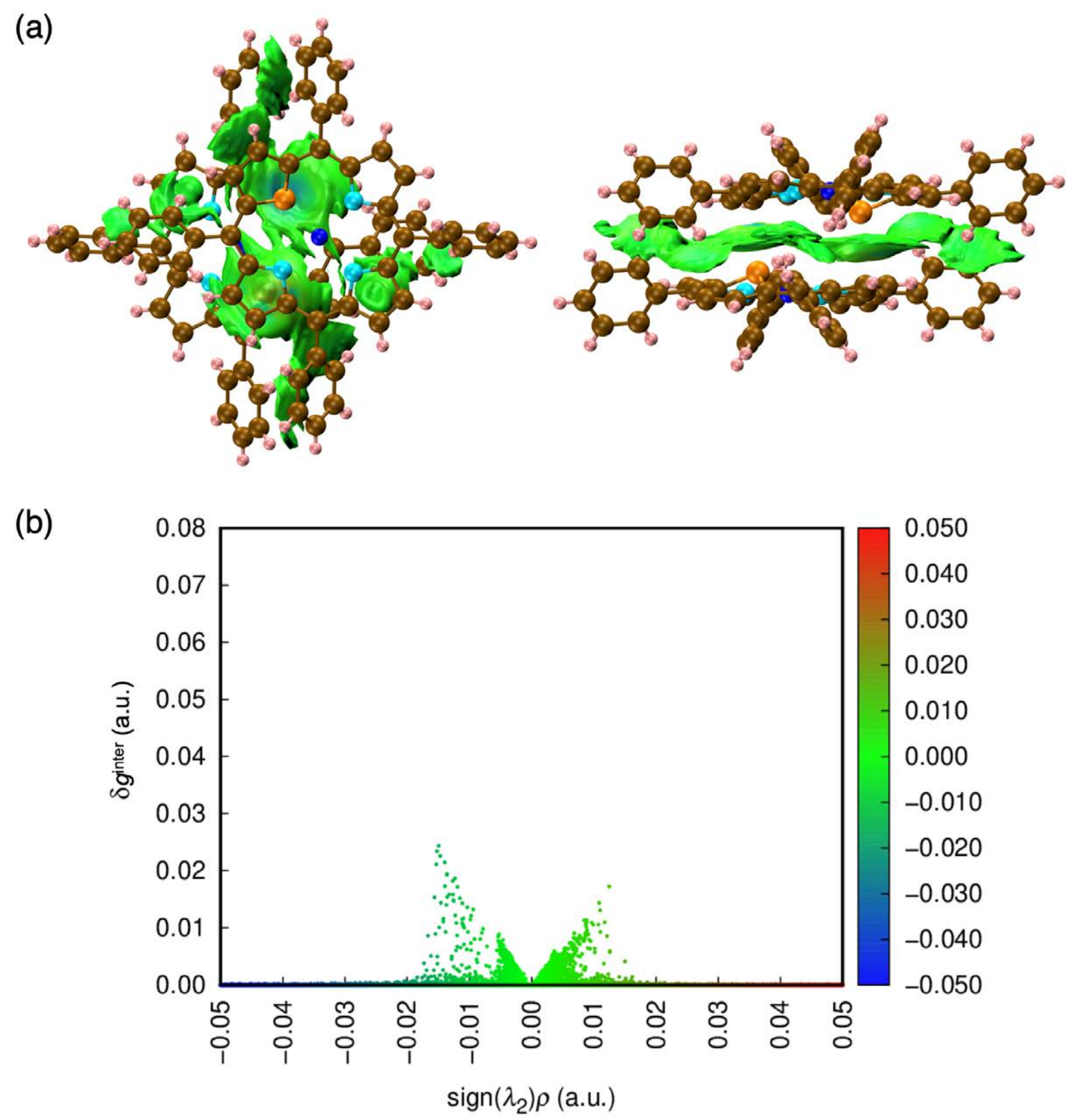

Fig. S36 NCI analysis[S18,19] of dimeric **1cu**$^+$ in the crystal structure of **1cu**$^+$-$B(C_6F_5)_4^-$: (a) IGMH[S20] isosurface and (b) δg^{inter} vs *sign*(λ_2)ρ plot. Weakly attractive regions corresponding to chalcogen bonding and $^i\pi$–$^i\pi$ interactions are observed. Atom color code: brown, pink, cyan, orange, and blue refer to carbon, hydrogen, nitrogen, sulfur, and copper, respectively.

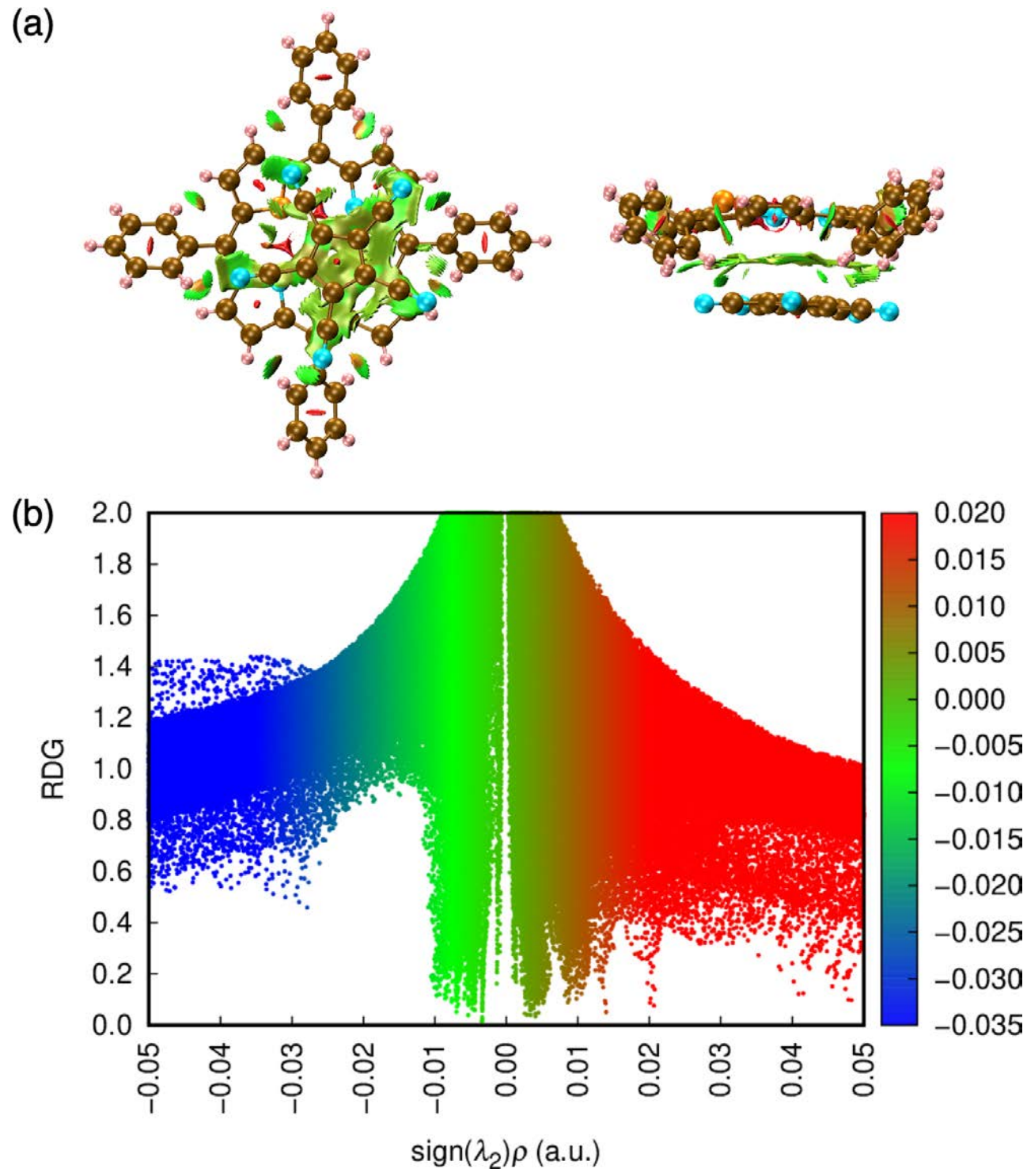

Fig. S37 NCI analysis[S18,19] of *π-sip* in the crystal structure of **1cu**$^+$-PCCp$^-$: (a) RDG isosurface and (b) RDG vs *sign*(λ_2)ρ plot. Weakly attractive region corresponding to $^i\pi$–$^i\pi$ interactions within the *π-sip* was observed. Atom color code: brown, pink, cyan, orange, and blue refer to carbon, hydrogen, nitrogen, sulfur, and copper, respectively.

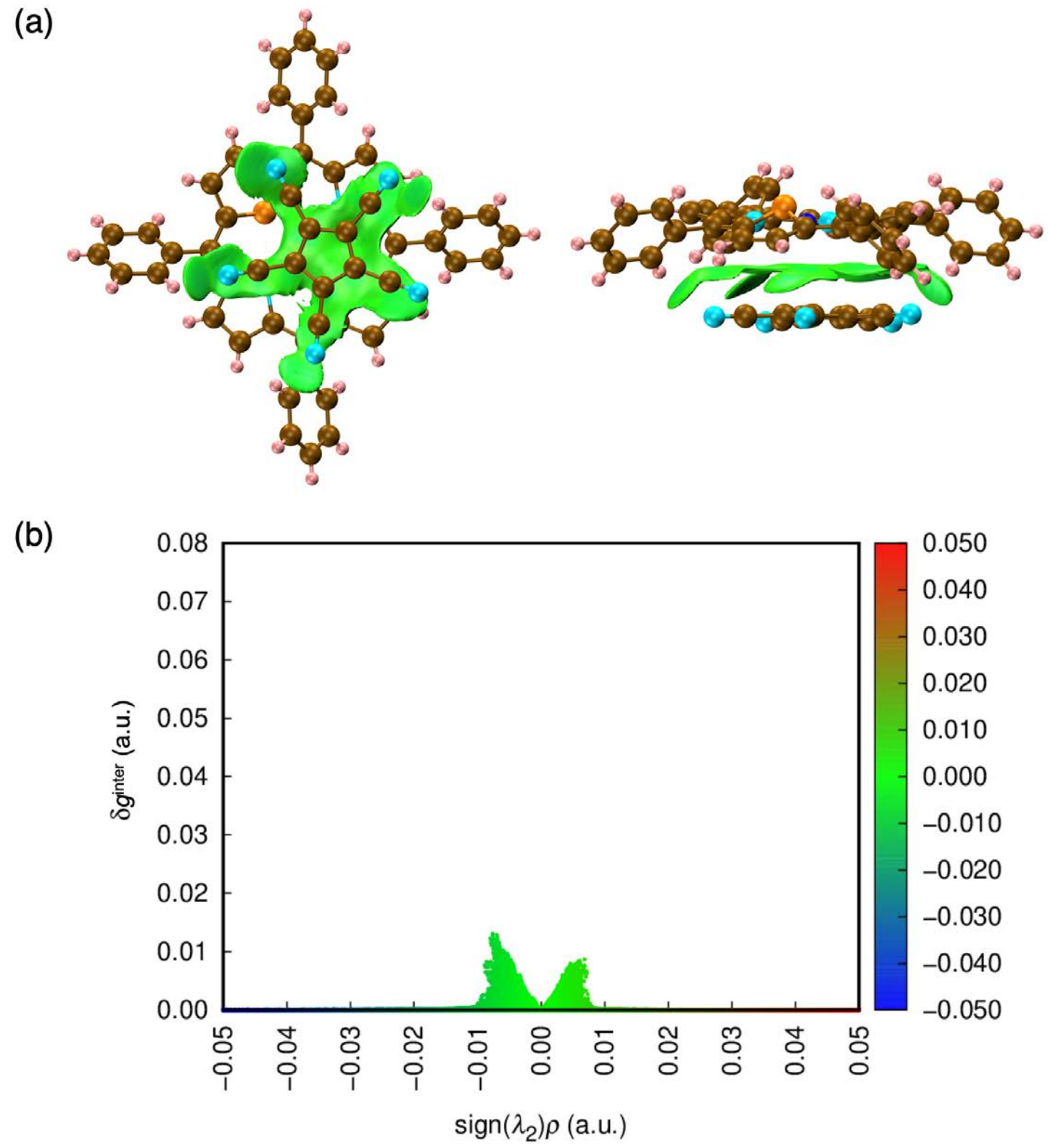

Fig. S38 NCI analysis[S18,19] of *π-sip* in the crystal structure of **1cu**$^+$-PCCp$^-$: (a) IGMH[S20] isosurface and (b) δg^{inter} vs *sign*(λ_2)ρ plot. Weakly attractive region corresponding to $^i\pi$–$^i\pi$ interactions within the *π-sip* was observed. Atom color code: brown, pink, cyan, orange, and blue refer to carbon, hydrogen, nitrogen, sulfur, and copper, respectively.

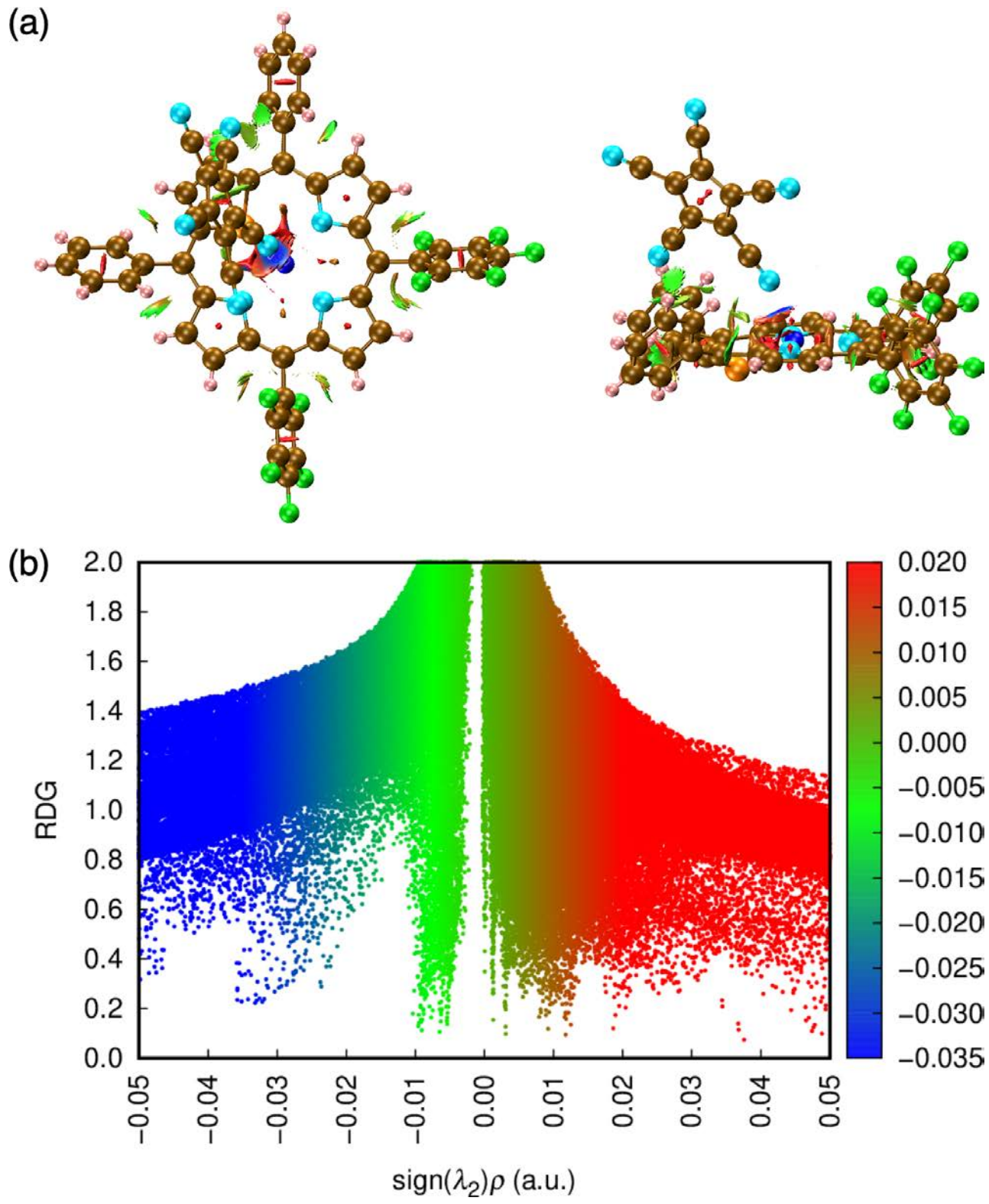

Fig. S39 NCI analysis[S18,19] of ion pair with axial coordination in the crystal structure of **2cu**$^+$-PCCp$^-$: (a) RDG isosurface and (b) RDG vs *sign*(λ_2)ρ plot. Weakly attractive region corresponding to axial coordination of the PCCp$^-$-N toward Cu was observed. Atom color code: brown, pink, cyan, yellow green, orange, and blue refer to carbon, hydrogen, nitrogen, fluorine, sulfur, and copper, respectively.

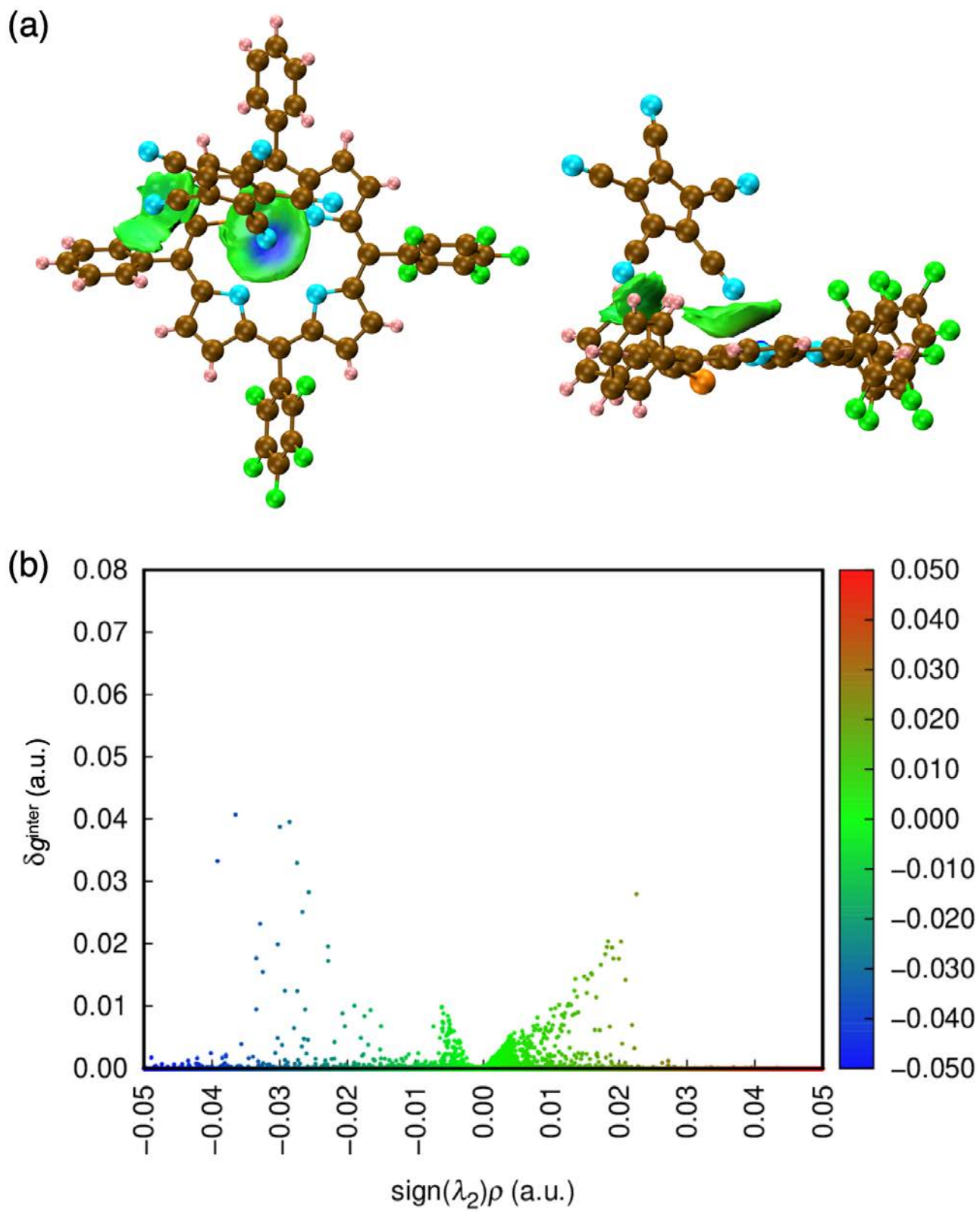

Fig. S40 NCI analysis[S18,19] of ion pair with axial coordination in the crystal structure of **2cu**$^+$-PCCp$^-$: (a) IGMH[S20] isosurface and (b) δg^{inter} vs *sign*(λ_2)ρ plot. Weakly attractive region corresponding to axial coordination of the PCCp$^-$-N toward Cu was observed. Atom color code: brown, pink, cyan, yellow green, orange, and blue refer to carbon, hydrogen, nitrogen, fluorine, sulfur, and copper, respectively.

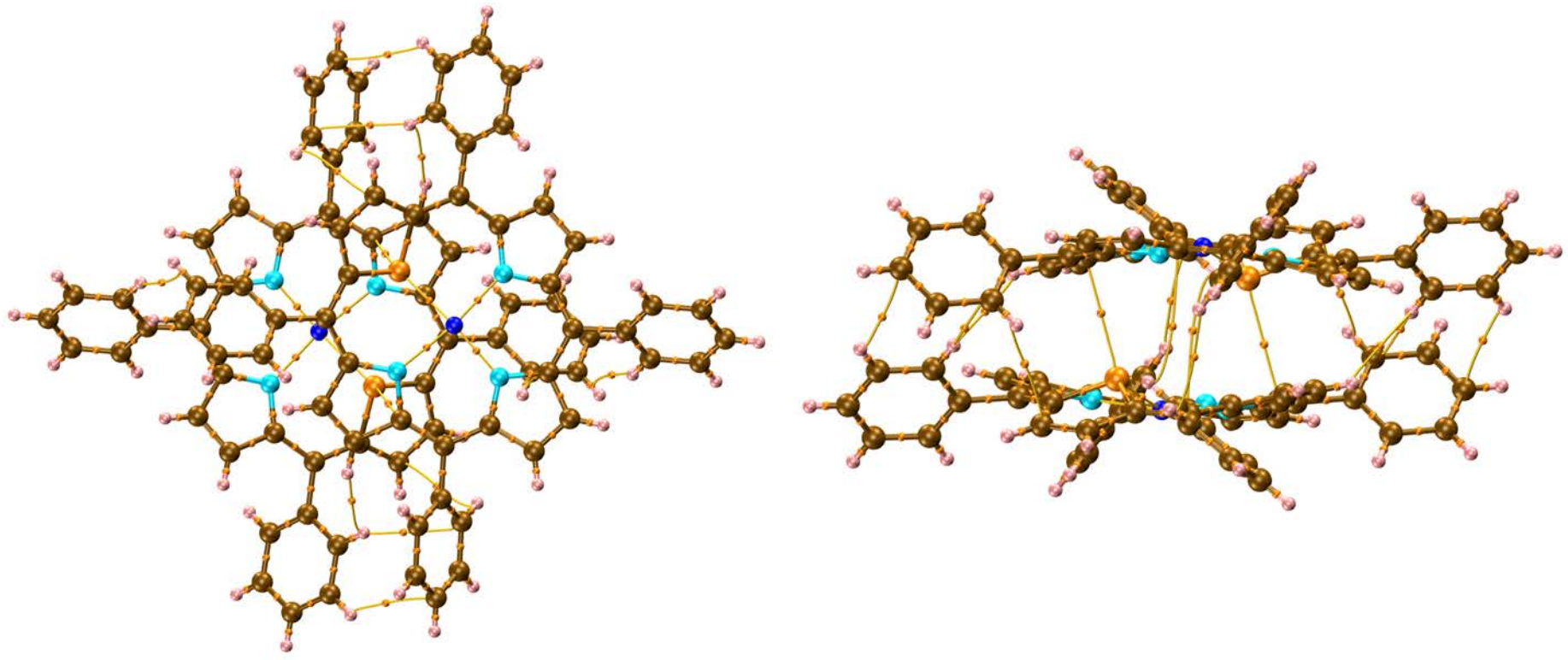

Fig. S41 Quantum theory of atoms in molecules (QTAIM) topology analysis[S21] of **1cu**$^+$-BF_4^- (top and side views). The bond critical points (BCPs) are shown as small orange spheres, and the S···π (pyrrole ring) interactions are observed. Atom color code: brown, pink, cyan, orange, and blue refer to carbon, hydrogen, nitrogen, sulfur, and copper, respectively.

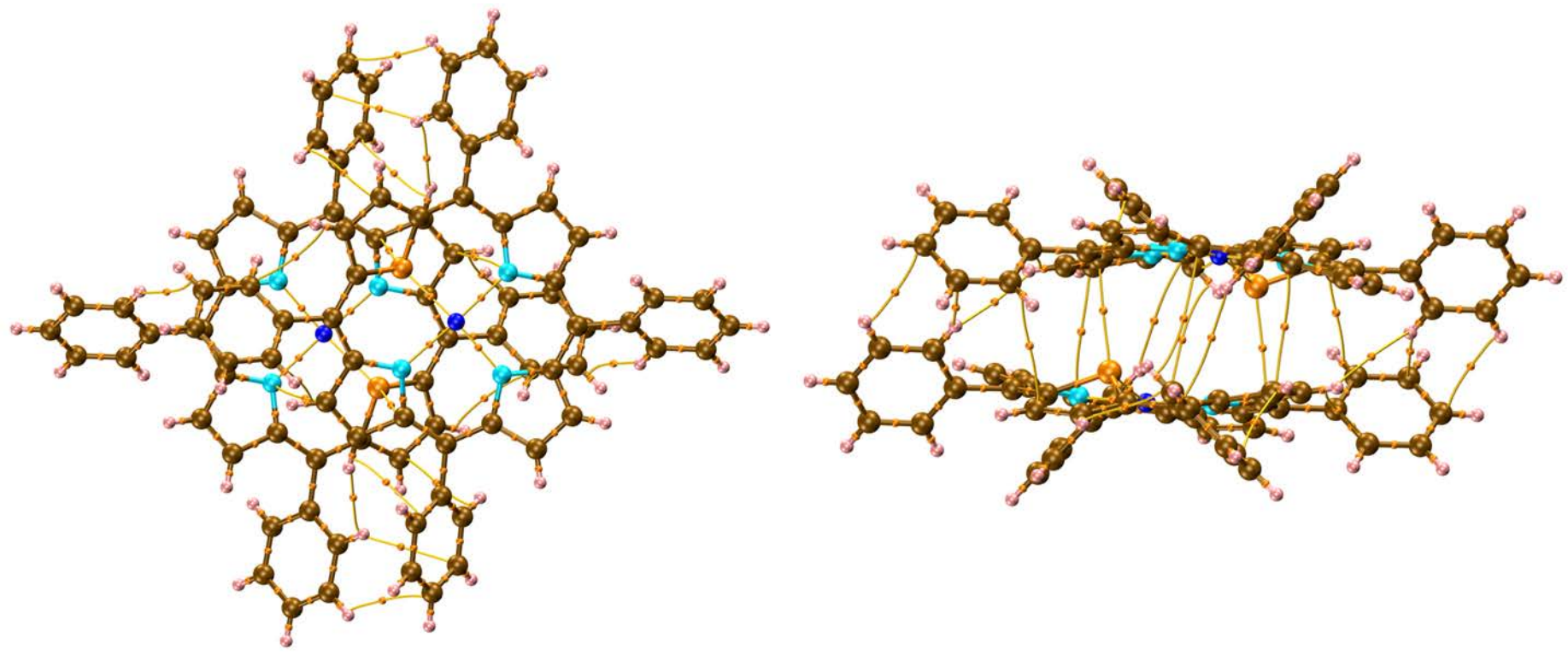

Fig. S42 Quantum theory of atoms in molecules (QTAIM) topology analysis[S21] of **1cu**$^+$-PF_6^- (top and side views). The bond critical points (BCPs) are shown as small orange spheres, and the S···π (pyrrole ring) interactions are observed. Atom color code: brown, pink, cyan, orange, and blue refer to carbon, hydrogen, nitrogen, sulfur, and copper, respectively.

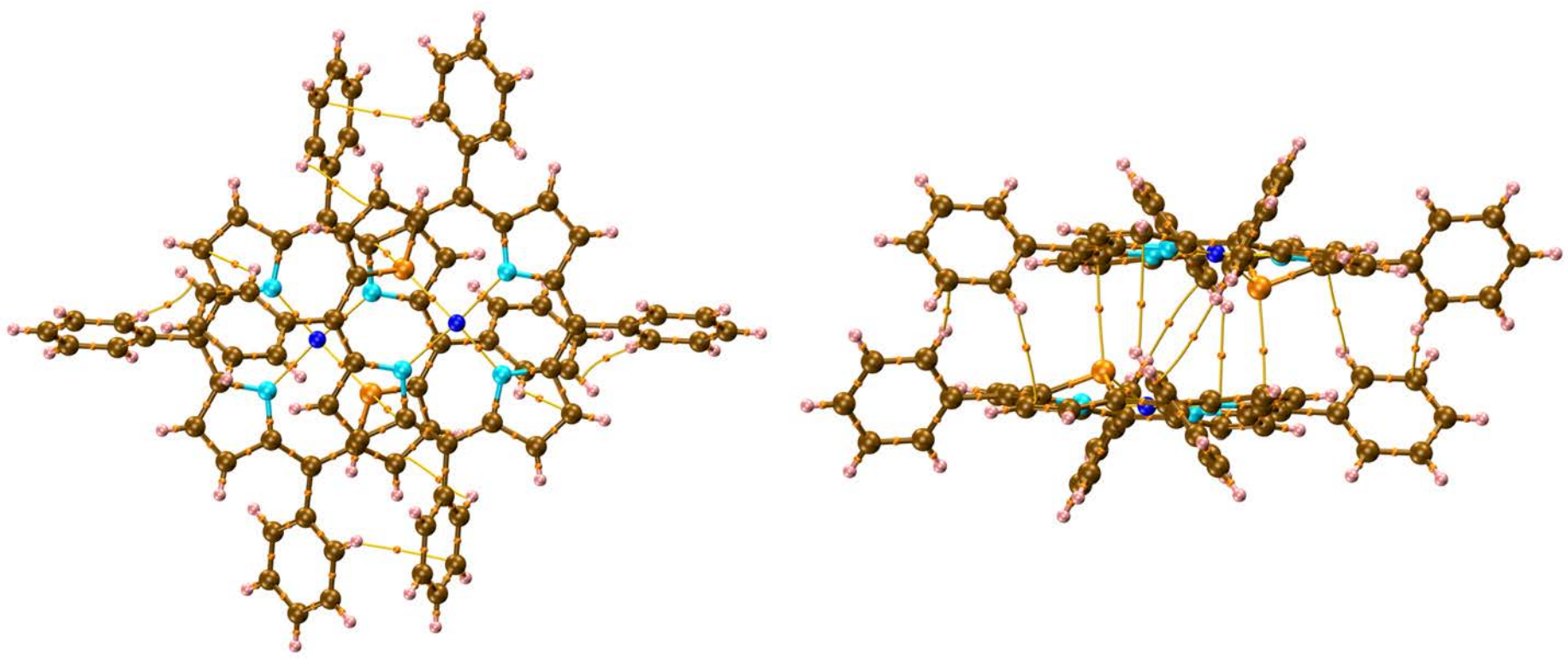

Fig. S43 Quantum theory of atoms in molecules (QTAIM) topology analysis[S21] of **1cu**$^+$-$B(C_6F_5)_4^-$ (top and side views). The bond critical points (BCPs) are shown as small orange spheres, and the S···π (pyrrole ring) interactions are observed. Atom color code: brown, pink, cyan, orange, and blue refer to carbon, hydrogen, nitrogen, sulfur, and copper, respectively.

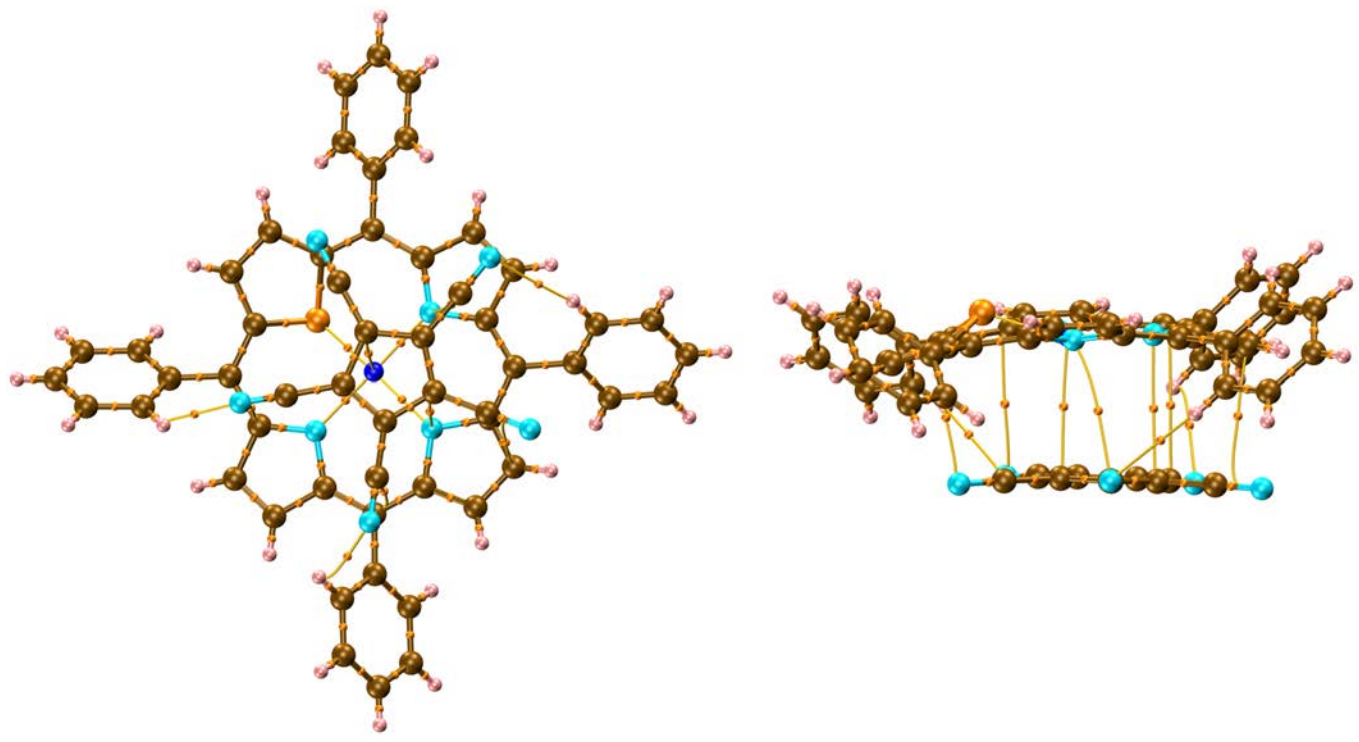

Fig. S44 Quantum theory of atoms in molecules (QTAIM) topology analysis[S21] of **1cu**$^{+}$-PCCp^{-} (top and side views). The bond critical points (BCPs) are shown as small orange spheres, and $^{i}\pi$–$^{i}\pi$ interactions are observed. Atom color code: brown, pink, cyan, orange, and blue refer to carbon, hydrogen, nitrogen, sulfur, and copper, respectively.

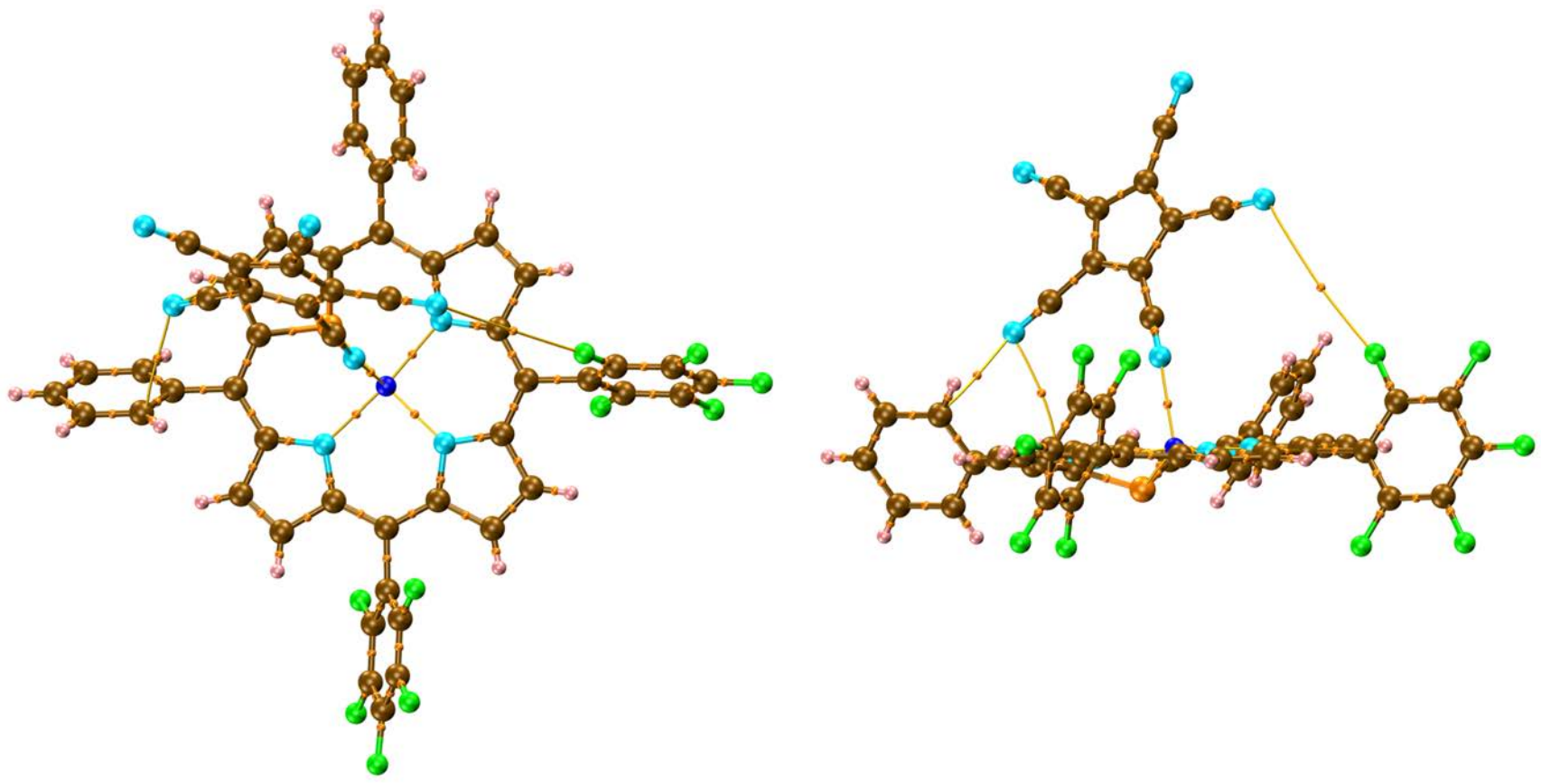

Fig. S45 Quantum theory of atoms in molecules (QTAIM) topology analysis[S21] of **2cu**$^{+}$-PCCp^{-} (top and side views). The bond critical points (BCPs) are shown as small orange spheres. Atom color code: brown, pink, cyan, green, orange, and blue refer to carbon, hydrogen, nitrogen, fluorine, sulfur, and copper, respectively.

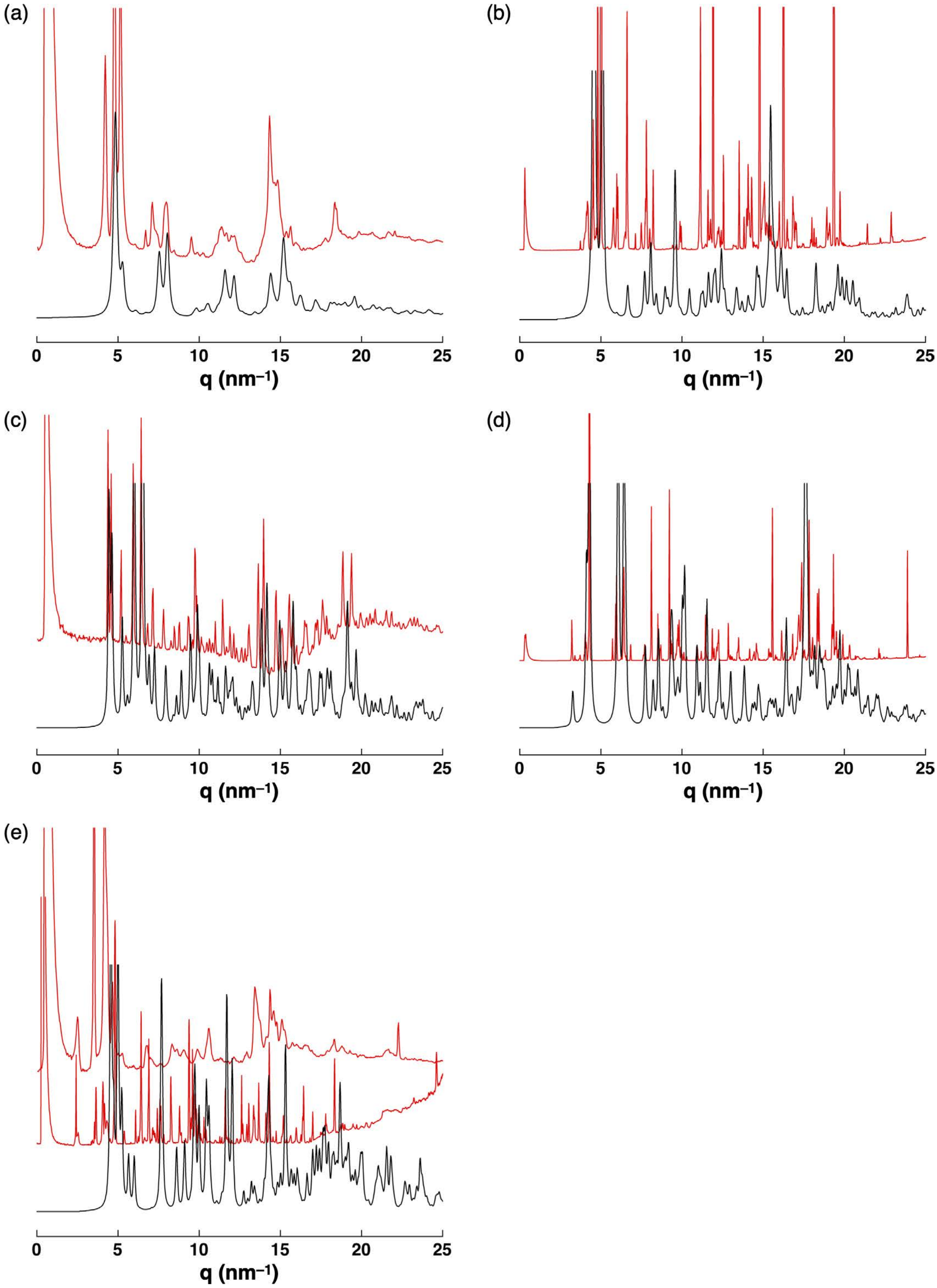

Fig. S46 XRD patterns of the solid materials for magnetic properties (25 °C) (red) and calculated patterns of single crystals (black) of (a) **1cu**$^+$-BF_4^-, (b) **1cu**$^+$-PF_6^-, (c) **1cu**$^+$-$B(C_6F_5)_4^-$, (d) **1cu**$^+$-PCCp$^-$, and (e) **2cu**$^+$-PCCp$^-$. For **2cu**$^+$-PCCp$^-$, solid-state ESR (Fig. S97) and magnetic susceptibility measurements (Fig. S98) were performed by combining two batches of solid materials. The detailed discussions are shown in the following Le Bail fitting profiles (Fig. S47).

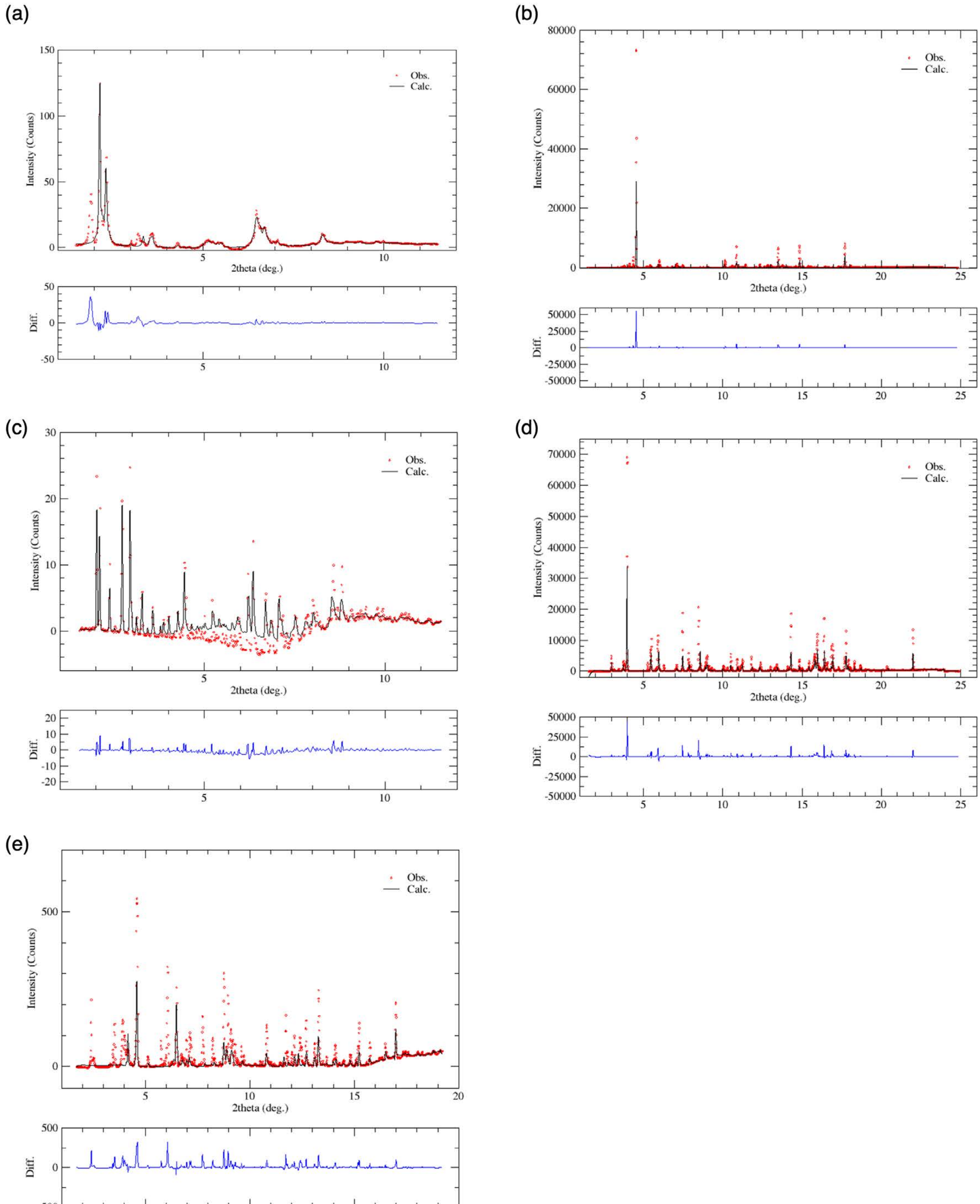

Fig. S47 Le Bail fitting profiles for (a) **1cu**$^{+}$-BF_4^{-}, (b) **1cu**$^{+}$-PF_6^{-}, (c) **1cu**$^{+}$-$B(C_6F_5)_4^{-}$, (d) **1cu**$^{+}$-PCCp^{-}, and (e) **2cu**$^{+}$-PCCp^{-} as observed (red) and calculated (black) powder XRD patterns (Fig. S46) (top) and the difference curves and Bragg reflection positions (bottom). The refinement converged with R_{wp} values of 32.572%, 75.376%, 35.779%, 69.639%, and 65.552%, respectively. While the solid samples of **1cu**$^{+}$-BF_4^{-}, **1cu**$^{+}$-PF_6^{-}, **1cu**$^{+}$-$B(C_6F_5)_4^{-}$, and **1cu**$^{+}$-PCCp^{-} show reasonable agreement with the lattice parameters of the corresponding single-crystal structures, minor discrepancies can be attributed to differences in measurement conditions and the presence of polymorphs.

[S5] (a) N. Yasuda, H. Murayama, Y. Fukuyama, J. E. Kim, S. Kimura, K. Toriumi, Y. Tanaka, Y. Moritomo, Y. Kuroiwa, K. Kato, H. Tanaka and M. Takata, *J. Synchrotron Rad.*, 2009, **16**, 352–357; (b) N. Yasuda, Y. Fukuyama, K. Toriumi, S. Kimura and M. Takata, *AIP Conf. Proc.*, 2010, **1234**, 147–150.

[S6] K. Hasegawa, N. Shimizu, H. Okumura, N. Mizuno, S. Baba, K. Hirata, T. Takeuchi, H. Yamazaki, Y. Senba, H. Ohashi, M. Yamamoto and T. Kumasaka, *J. Synchrotron Radiat.*, 2013, **20**, 910–913.

[S7] K. Sugimoto, H. Ohsumi, S. Aoyagi, E. Nishibori, C. Moriyoshi, Y. Kuroiwa, H. Sawa and M. Takata, *AIP Conf. Proc.*, 2010, **1234**, 887–890.

[S8] G. M. Sheldrick, *Acta Crystallogr. Sect. A*, 2008, **64**, 112–122.
[S9] (a) K. Wakita, *Yadokari-XG, Software for Crystal Structure Analyses*, 2001; (b) C. Kabuto, S. Akine, T. Nemoto and E. Kwon, *J. Cryst. Soc. Jpn.*, 2009, **51**, 218–224.
[S10] C. B. Hübschle, G. M. Sheldrick and B. Dittrich, *J. Appl. Cryst.*, 2011, **44**, 1281–1284.
[S11] A. L. Spek, *Acta Crystallogr. C*, 2015, **71**, 9–18.
[S12] L. Yang, D. R. Powell and R. P. Houser, *Dalton Trans.*, 2007, 955–964.
[S13] A. W. Addison, T. N. Rao, J. Reedijk, J. van Rijn and G. C. Verschoor, *J. Chem. Soc., Dalton Trans.*, 1984, 1349–1356.
[S14] (a) M. Pinsky and D. Avnir, *Inorg. Chem.*, 1998, **37**, 5575–5582; (b) S. Alvarez and M. Llunell, *J. Chem. Soc., Dalton Trans.*, 2000, 3288–3303; (c) J. Cirera, P. Alemany and S. Alvarez, *Chem. Eur. J.*, 2004, **10**, 190–207.
[S15] P. R. Spackman, M. J. Turner, J. J. McKinnon, S. K. Wolff, D. J. Grimwood, D. Jayatilaka and M. A. Spackman, *J. Appl. Cryst.*, 2021, **54**, 1006–1011.
[S16] (a) M. A. Spackman and D. Jayatilaka, *CrystEngComm*, 2009, **11**, 19–32; (b) J. J. McKinnon, M. A. Spackman and A. S. Mitchell, *Acta Crystallogr. Sect. B*, 2004, **60**, 627–668.
[S17] Y. Sasano, H. Tanaka, Y. Haketa, Y. Kobayashi, Y. Ishibashi, T. Morimoto, R. Sato, Y. Shigeta, N. Yasuda, T. Asahi and H. Maeda, *Chem. Sci.*, 2021, **12**, 9645–9657.
[S18] R. A. Boto, F. Peccati, R. Laplaza, C. Quan, A. Carbone, J.-P. Piquemal, Y. Maday and J. Contreras-Garcia, *NCIPLOT4*, 2020.
[S19] Reports for *NCIPLOT*: (a) E. R. Johnson, S. Keinan, P. Mori-Sanchez, J. Contreras-Garcia, A. J. Cohen and W. Yang, *J. Am. Chem. Soc.*, 2010, **132**, 6498–6506; (b) J. Contreras-Garcia, E. R. Johnson, S. Keinan, R. Chaudret, J.-P. Piquemal and D. N. Beratan, W. Yang, *J. Chem. Theory Comput.*, 2011, **7**, 625–632; (c) M. Ranjbar, A. Nowroozi and E. Nakhaei, *Comput. Theor. Chem.*, 2022, **1216**, 113867.
[S20] T. Lu and Q. Chen, *J. Comput. Chem.*, 2022, **43**, 539–555.
[S21] (a) *Atoms in Molecules: A Quantum Theory*, ed. R. F. W. Bader, Oxford University Press, Oxford, 1990; (b) R. F. Bader, *Chem. Rev.*, 1991, **91**, 893–928; (c) R. F. Bader, *J. Phys. Chem. A*, 1998, **102**, 7314–7323.

3. Theoretical studies

DFT calculations. DFT calculations were carried out using *Gaussian 16* program.[S22]

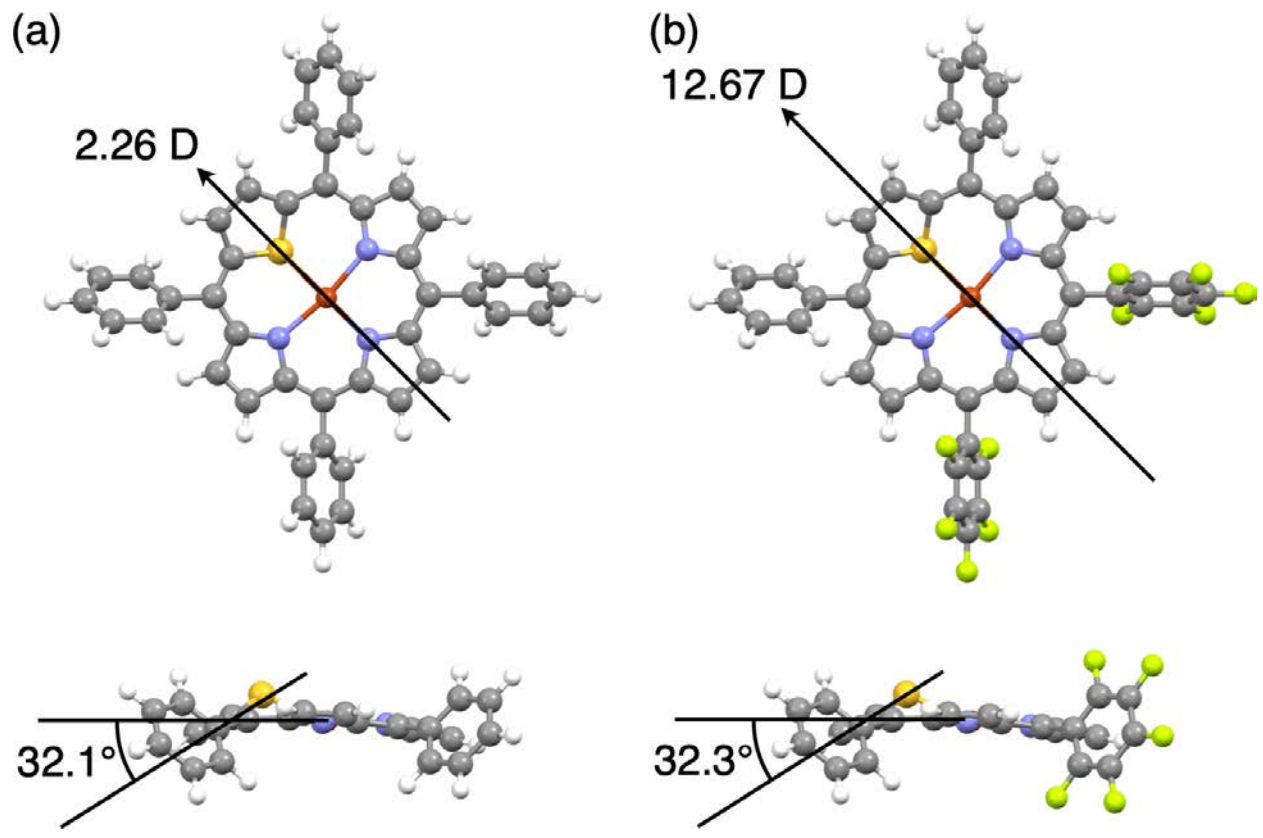

Fig. S48 Optimized structures of (a) **1cu**$^+$ and (b) **2cu**$^+$ at UB3LYP/6-31+G(d,p) with LanL2DZ for Cu as top and side views. Dipole moments were 2.26 and 12.67 D, respectively. The dihedral angles between the thiophene plane and the core porphyrin plane (25 atoms) are 32.1° and 32.3°, respectively. Mean-plane deviations of the core part (25 atoms) and τ_4 values are 0.27/0.26Å and 0.20/0.19, respectively.

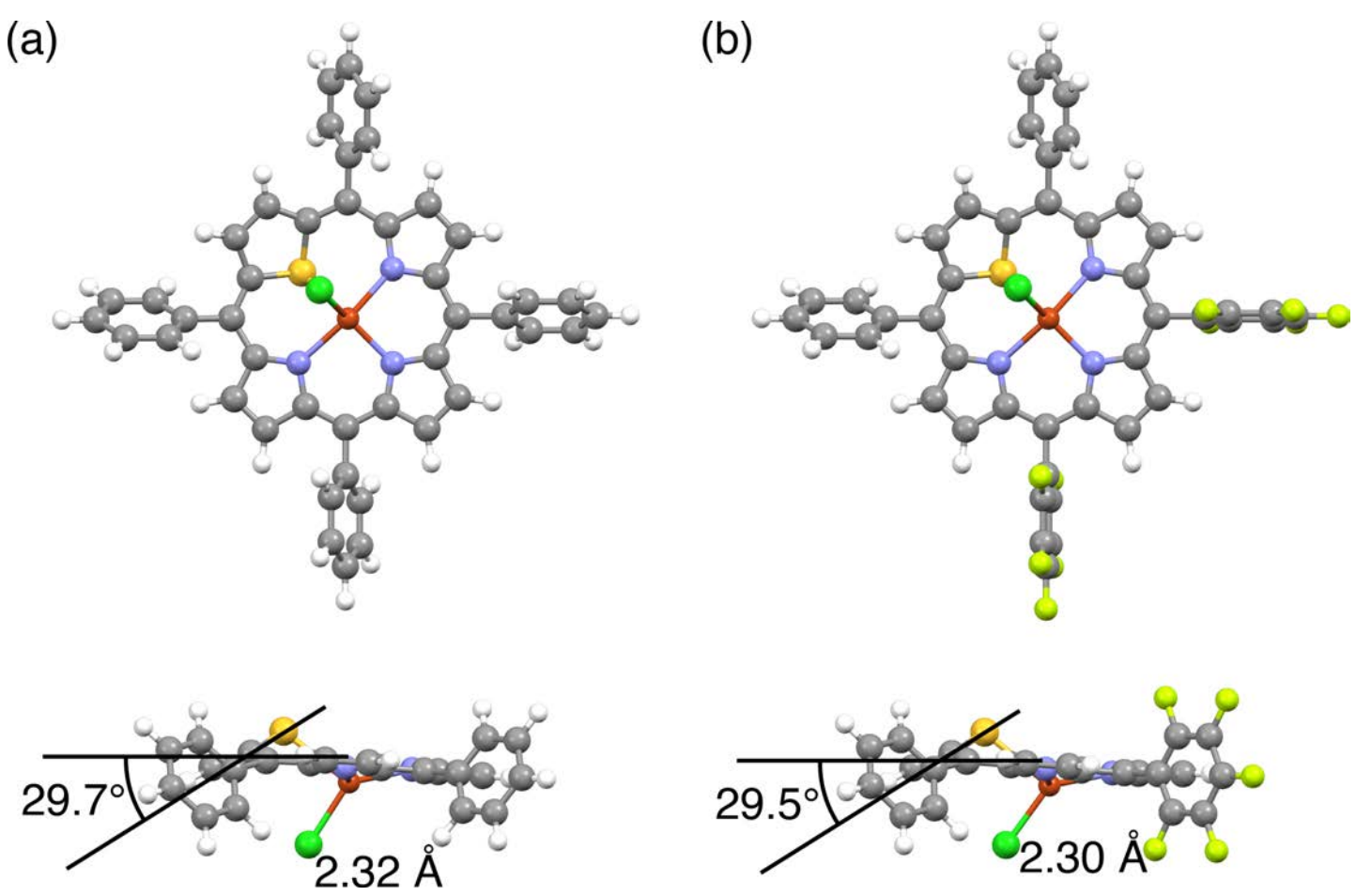

Fig. S49 Optimized structures of (a) **1cu**$^+$-Cl$^-$ and (b) **2cu**$^+$-Cl$^-$ at UB3LYP/6-31+G(d,p) with LanL2DZ for Cu as top and side views. The dihedral angles between the thiophene plane and the core porphyrin plane (25 atoms) and Cu···Cl distance are 29.7°/29.5° and 2.32/2.30 Å, respectively. Mean-plane deviations of the core part (25 atoms), τ_4, and τ_5 values are 0.25/0.25 Å, 0.53/0.56, and 0.33/0.24, respectively.

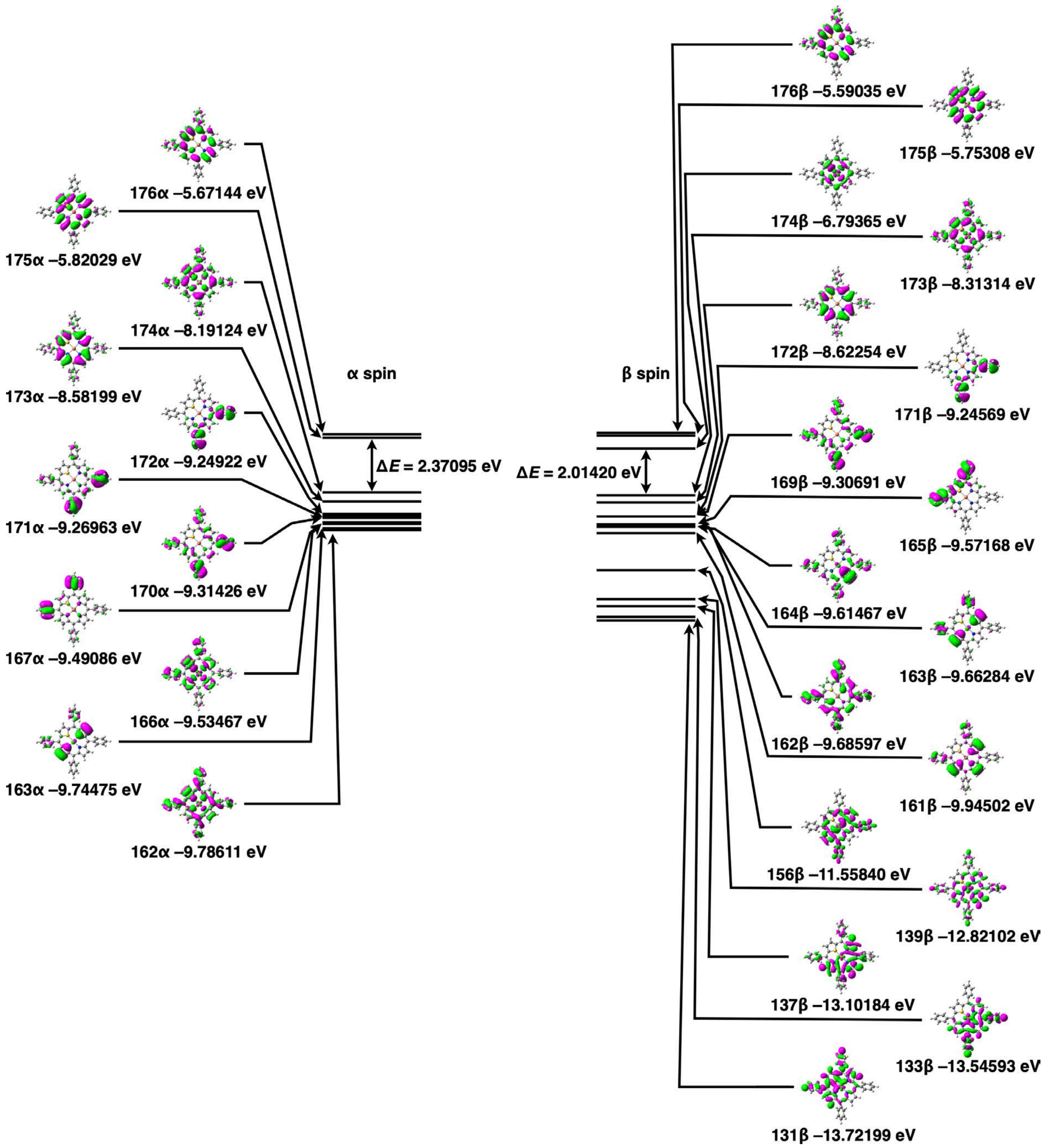

Fig. S50 Molecular orbitals (HOMO/LUMO) of doublet-state **1cu**$^{+}$ with α-spin (left) and β-spin (right) estimated at UB3LYP/6-31+G(d,p) with LanL2DZ for Cu.

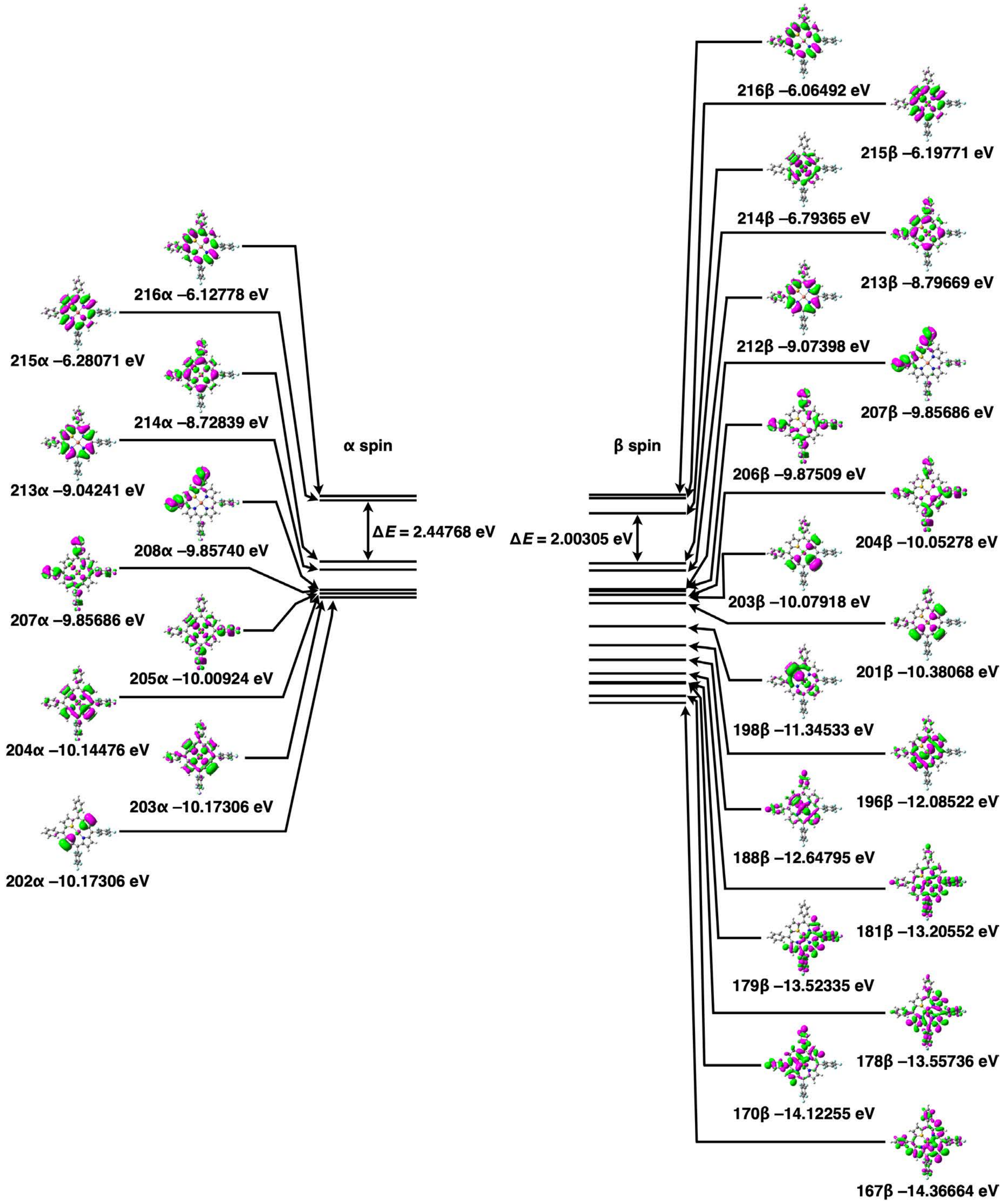

Fig. S51 Molecular orbitals (HOMO/LUMO) of doublet-state **2cu**$^{+}$ with α-spin (left) and β-spin (right) estimated at UB3LYP/6-31+G(d,p) with LanL2DZ for Cu.

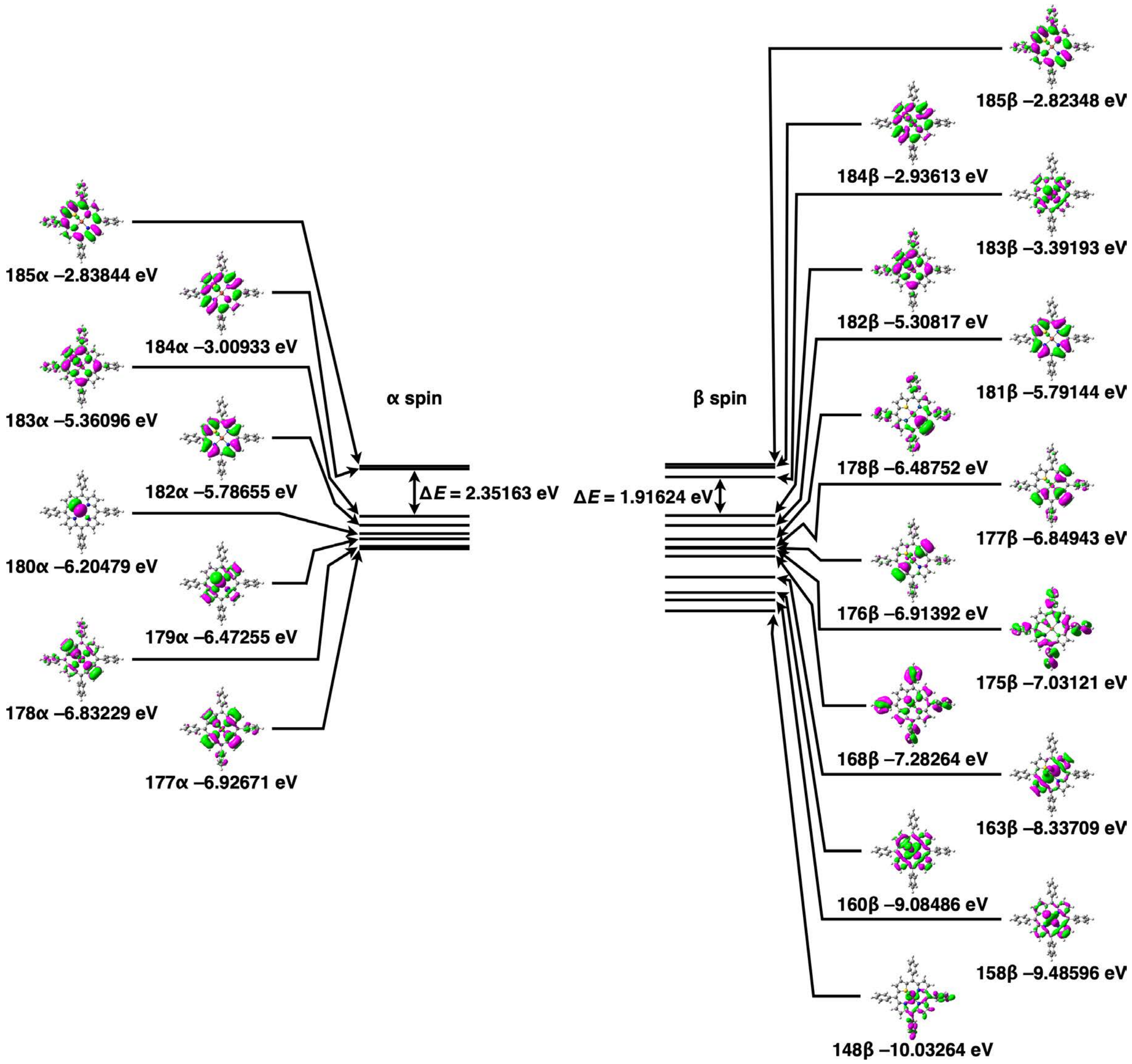

Fig. S52 Molecular orbitals (HOMO/LUMO) of doublet-state **1cu**$^{+}$-Cl^{-} with α-spin (left) and β-spin (right) estimated at UB3LYP/6-31+G(d,p) with LanL2DZ for Cu.

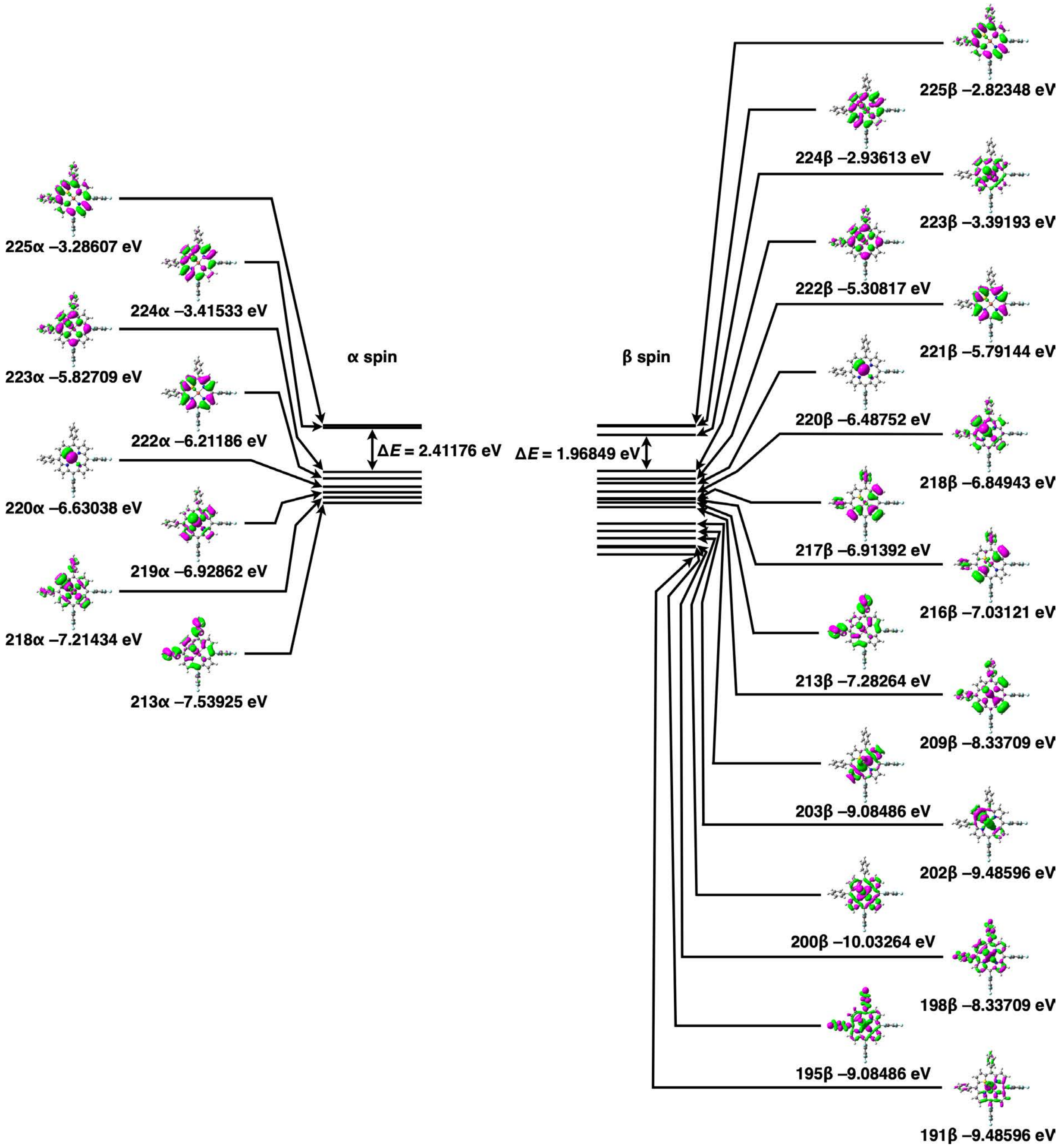

Fig. S53 Molecular orbitals (HOMO/LUMO) of doublet-state **2cu**$^+$-Cl$^-$ with α-spin (left) and β-spin (right) estimated at UB3LYP/6-31+G(d,p) with LanL2DZ for Cu.

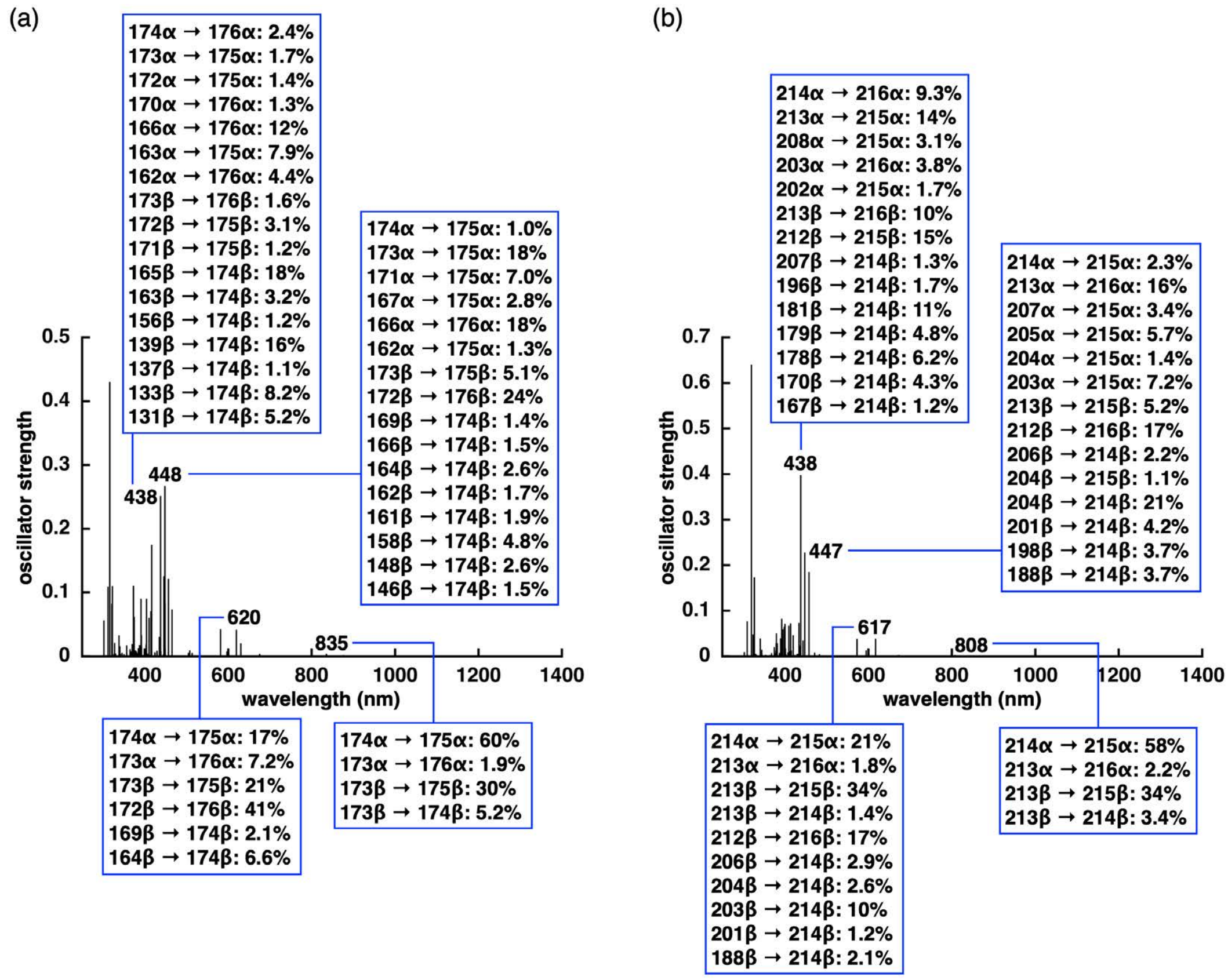

Fig. S54 TD-DFT-based UV/vis absorption stick spectra of (a) **1cu**$^+$ and (b) **2cu**$^+$ with the transitions correlated with molecular orbitals estimated at UB3LYP/6-31+G(d,p) with LanL2DZ for Cu.

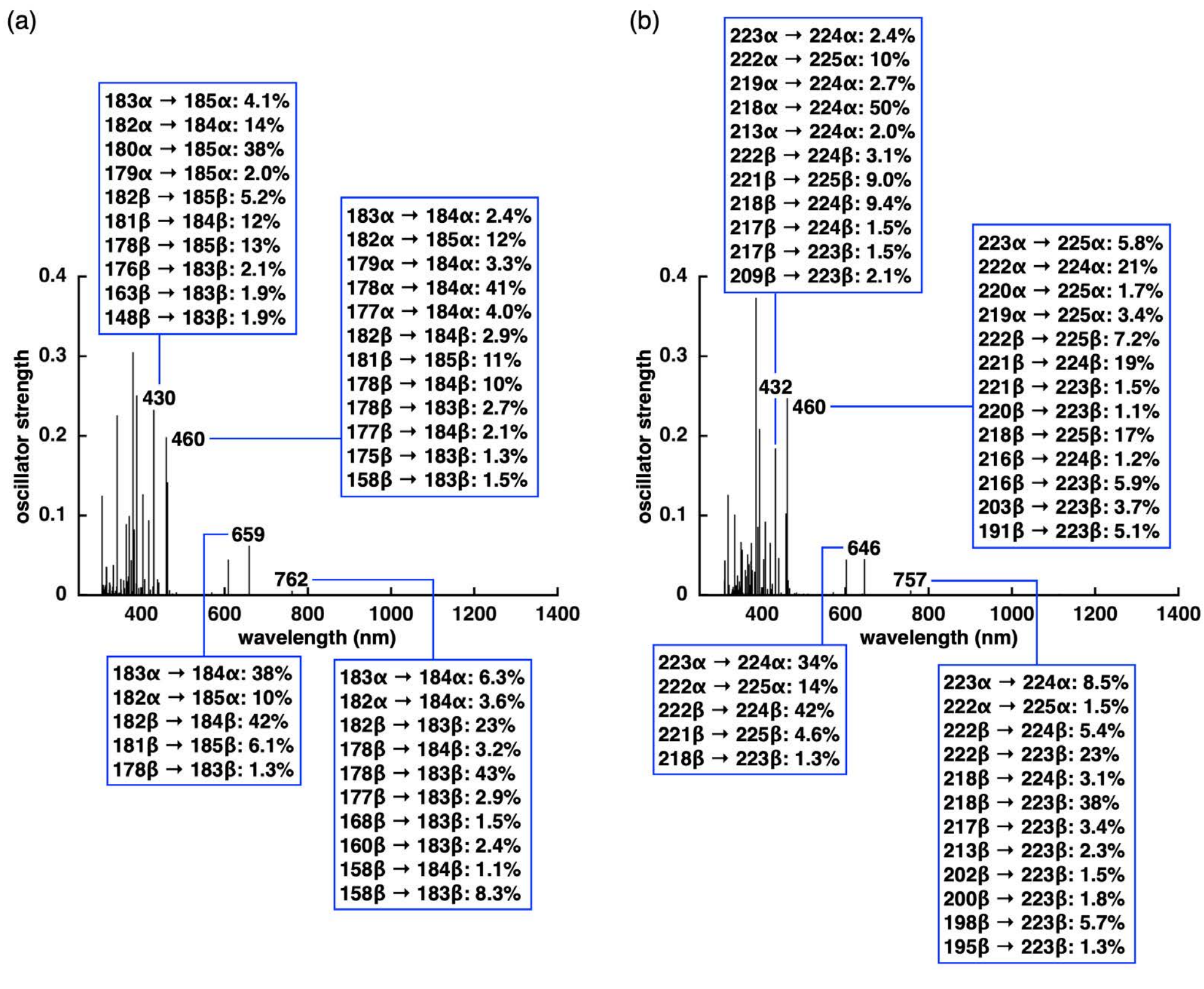

Fig. S55 TD-DFT-based UV/vis absorption stick spectra of (a) **1cu**$^+$-Cl$^–$ and (b) **2cu**$^+$-Cl$^–$ with the transitions correlated with molecular orbitals estimated at UB3LYP/6-31+G(d,p) with LanL2DZ for Cu.

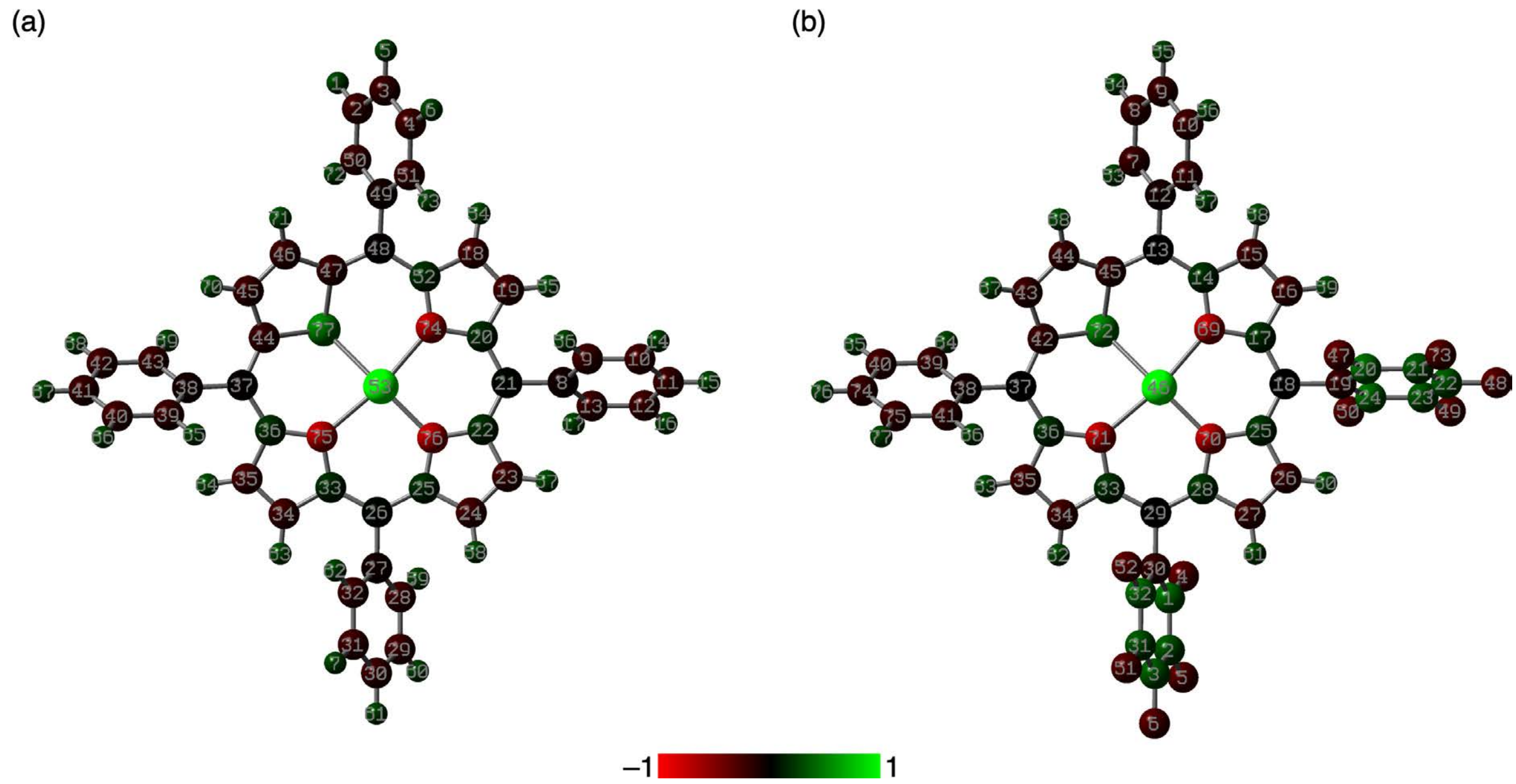

Fig. S56 Molecular atom labels of (a) **1cu**$^{+}$ and (b) **2cu**$^{+}$ visualized with color mapping based on natural population analysis (NPA) charges (Table S6,7) obtained from NBO calculations[S23,24] at UB3LYP/6-31+G(d,p) with LanL2DZ for Cu that are related with Lewis acidity.

Table S6 Natural Population Analysis (NPA) charges of **1cu**$^{+}$ obtained from NBO calculations[S23,24] at UB3LYP/6-31+G(d,p) with LanL2DZ for Cu. Atom labels were referred to Fig. S56.

atom labels		charge
H	1	0.26132
C	2	-0.23610
C	3	-0.21750
C	4	-0.23623
H	5	0.26133
H	6	0.26112
H	7	0.25958
C	8	-0.07390
C	9	-0.23037
C	10	-0.23564
C	11	-0.22628
C	12	-0.23491
C	13	-0.23022
H	14	0.25958
H	15	0.25988
H	16	0.25993
H	17	0.25725
C	18	-0.24314
C	19	-0.24731
C	20	0.22248
C	21	-0.00155
C	22	0.16535
C	23	-0.23117
C	24	-0.23117
C	25	0.16535
C	26	-0.00155
C	27	-0.07390
C	28	-0.23022
C	29	-0.23491
C	30	-0.22628
C	31	-0.23564
C	32	-0.23036
C	33	0.22248
C	34	-0.24731
C	35	-0.24315
C	36	0.17217
C	37	-0.01930
C	38	-0.07662
C	39	-0.22128
C	40	-0.23623
C	41	-0.21749
C	42	-0.23610
C	43	-0.22362
C	44	-0.18979
C	45	-0.21530
C	46	-0.21530
C	47	-0.18979
C	48	-0.01929
C	49	-0.07662
C	50	-0.22362
C	51	-0.22128
C	52	0.17217
Cu	53	1.16035
H	54	0.27354
H	55	0.27582
H	56	0.25636
H	57	0.27333
H	58	0.27333
H	59	0.25725
H	60	0.25993
H	61	0.25988
H	62	0.25636
H	63	0.27582
H	64	0.27354
H	65	0.25855
H	66	0.26112
H	67	0.26133
H	68	0.26132
H	69	0.25660
H	70	0.28338
H	71	0.28338
H	72	0.25660
H	73	0.25855
N	74	-0.65301
N	75	-0.65301
N	76	-0.63303
S	77	0.44317

Table S7 Natural Population Analysis (NPA) charges of **2cu**$^{+}$ obtained from NBO calculations[S23,24] at UB3LYP/6-31+G(d,p) with LanL2DZ for Cu. Atom labels were referred to Fig. S56.

atom labels		charge
C	1	0.38171
C	2	0.31301
C	3	0.33892
F	4	-0.31993
F	5	-0.30635
F	6	-0.30182
C	7	-0.22222
C	8	-0.23461
C	9	-0.21310
C	10	-0.23450
C	11	-0.22112
C	12	-0.08022
C	13	-0.00914
C	14	0.17807
C	15	-0.22815
C	16	-0.23850
C	17	0.17675
C	18	0.03335
C	19	-0.19097
C	20	0.38158
C	21	0.31244
C	22	0.33888
C	23	0.31219
C	24	0.38563
C	25	0.17119
C	26	-0.23522
C	27	-0.23266
C	28	0.25613
C	29	-0.03163
C	30	-0.18002
C	31	0.31240
C	32	0.38174
C	33	0.17938
C	34	-0.23562
C	35	-0.22800
C	36	0.17911
C	37	-0.00916
C	38	-0.08040
C	39	-0.22228
C	40	-0.23460
C	41	-0.22109
C	42	-0.19252
C	43	-0.20564
C	44	-0.21654
C	45	-0.19013
Cu	46	1.17663
F	47	-0.32138
F	48	-0.30182
F	49	-0.30649
F	50	-0.32001
F	51	-0.30703
F	52	-0.32101
H	53	0.25706
H	54	0.26324
H	55	0.26345
H	56	0.26369
H	57	0.26038
H	58	0.27700
H	59	0.26986
H	60	0.27070
H	61	0.26824
H	62	0.26964
H	63	0.27698
H	64	0.25708
H	65	0.26324
H	66	0.26038
H	67	0.28658
H	68	0.28805
N	69	-0.64308
N	70	-0.65302
N	71	-0.63935
S	72	0.45217
F	73	-0.30708
C	74	-0.21310
C	75	-0.23450
H	76	0.26345
H	77	0.26369

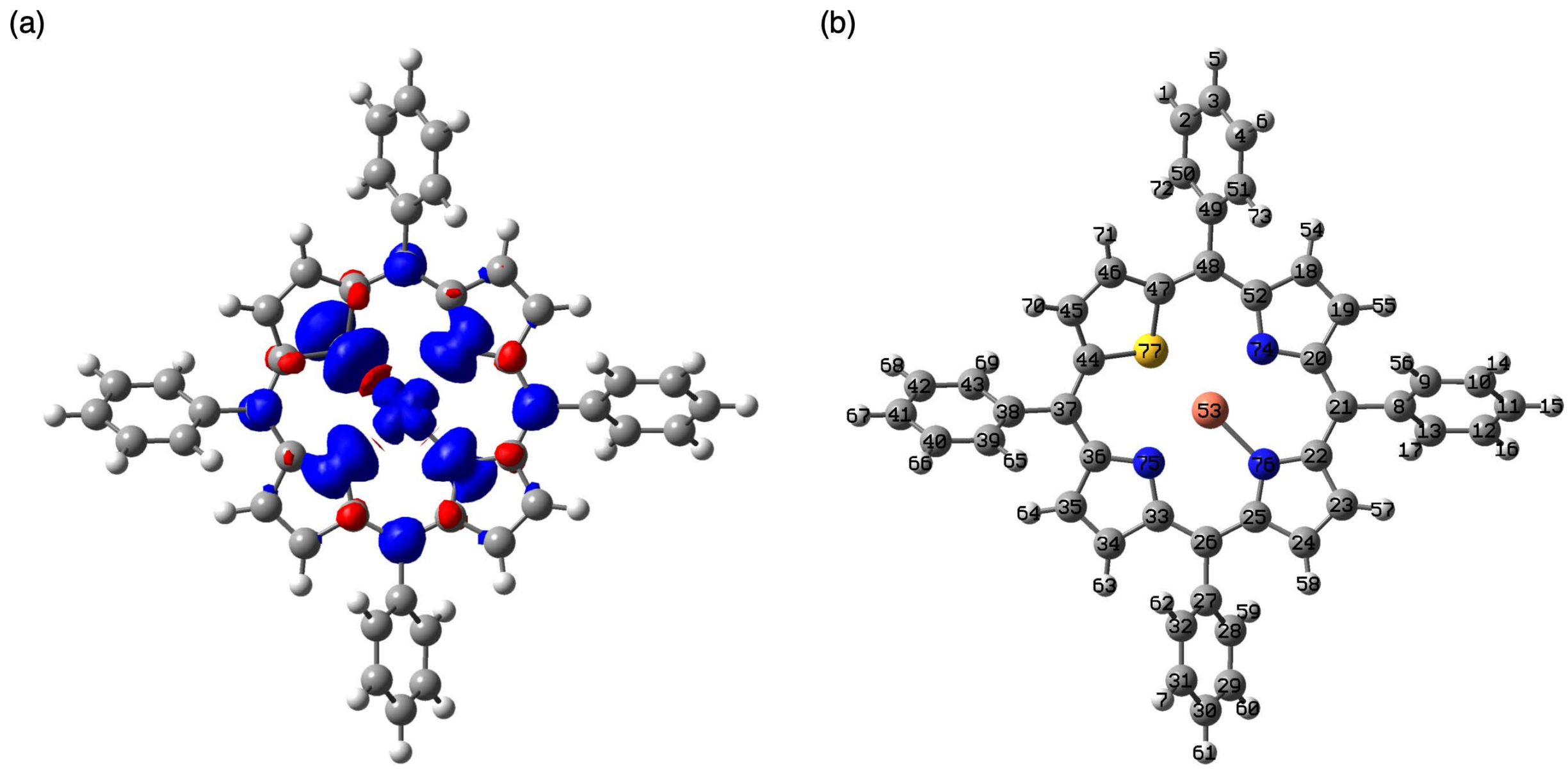

Fig. S57 Spin densities of dimeric **1cu**$^+$ as the optimized structure (Fig. S48) estimated at UB3LYP/Def2TZVP: (a) spin-density mapping (δ = 0.001) as (i) top and (ii) side views and (b) molecular structure with atom labels provided for referencing the spin-density values (Table S8) and for discussions related to the molar magnetic susceptibility (Fig. S98).

Table S8 Spin densities, excluding hydrogen atoms of **1cu**$^+$ estimated at UB3LYP/Def2TZVP. Atom labels were referred to Fig. S57.

atom labels		spin densities
2	C	-0.000937
3	C	0.002185
4	C	-0.000986
8	C	-0.003658
9	C	0.002682
10	C	-0.000902
11	C	0.001995
12	C	-0.000770
13	C	0.002611
18	C	-0.001216
19	C	0.003942
20	C	-0.017716
21	C	0.043534
22	C	-0.016366
23	C	0.001615
24	C	0.001620
25	C	-0.016361
26	C	0.043538
27	C	-0.003658
28	C	0.002610
29	C	-0.000769
30	C	0.001994
31	C	-0.000902
32	C	0.002681
33	C	-0.017714
34	C	0.003940
35	C	-0.001215
36	C	-0.007595
37	C	0.031564
38	C	-0.003627
39	C	0.002403
40	C	-0.000986
41	C	0.002185
42	C	-0.000937
43	C	0.002845
44	C	-0.015764
45	C	-0.000311
46	C	-0.000316
47	C	-0.015764
48	C	0.031558
49	C	-0.003626
50	C	0.002845
51	C	0.002403
52	C	-0.007591
53	Cu	0.472920
74	N	0.118675
75	N	0.118679
76	N	0.125743
77	S	0.112918

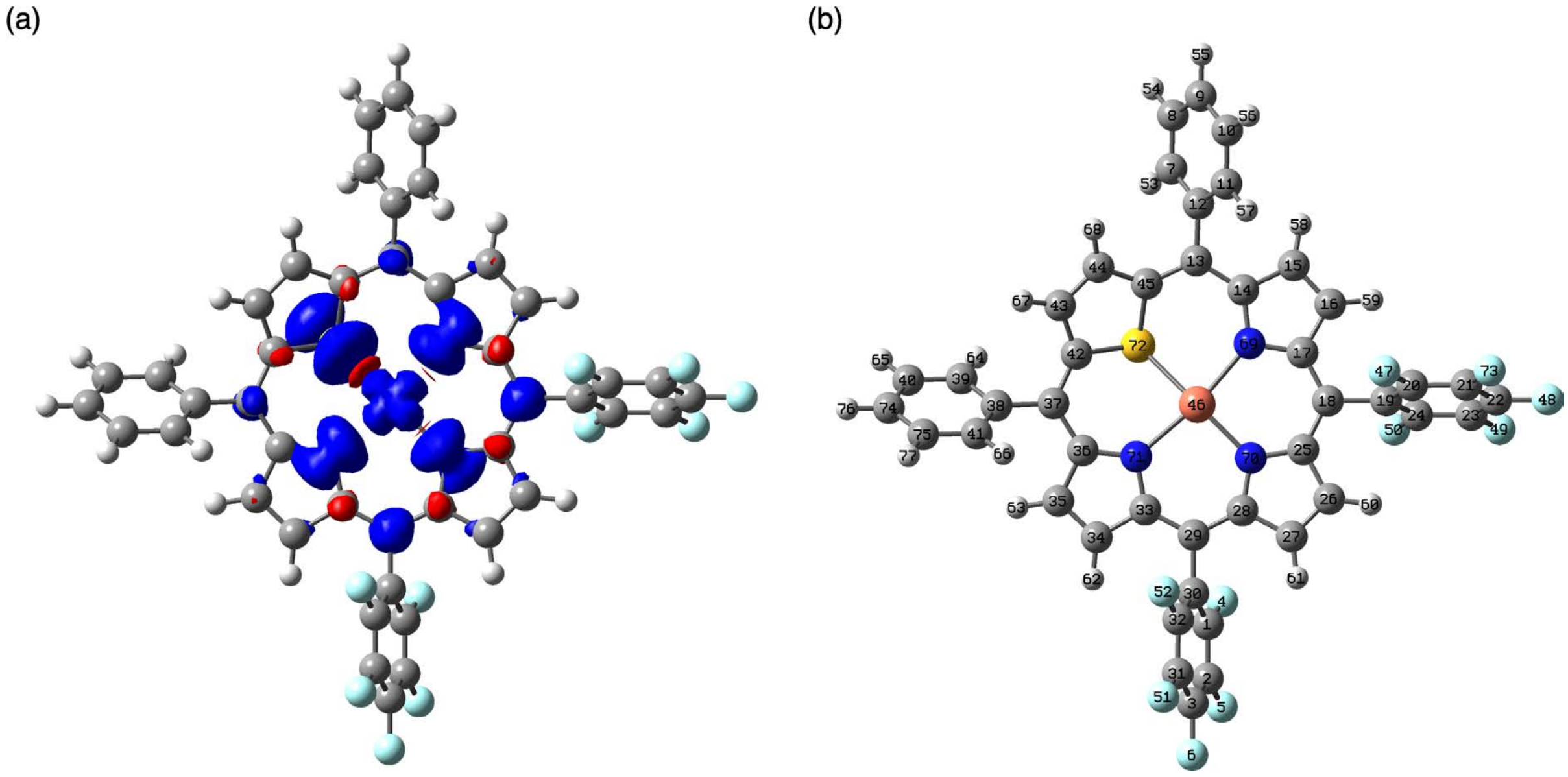

Fig. S58 Spin densities of dimeric **2cu**$^{+}$ as the optimized structure (Fig. S48) estimated at UB3LYP/Def2TZVP: (a) spin-density mapping (δ = 0.001) as (i) top and (ii) side views and (b) molecular structure with atom labels provided for referencing the spin-density values (Table S9) and for discussions related to the molar magnetic susceptibility (Fig. S98).

Table S9 Spin densities, excluding hydrogen atoms of **2cu**$^{+}$ estimated at UB3LYP/Def2TZVP. Atom labels were referred to Fig. S58.

atom labels		spin densities
1	C	0.001278
2	C	-0.000205
3	C	0.000461
4	F	0.000034
5	F	0.000016
6	F	0.000053
7	C	0.001830
8	C	-0.000520
9	C	0.001120
10	C	-0.000487
11	C	0.001286
12	C	-0.002566
13	C	0.019773
14	C	-0.002034
15	C	-0.002520
16	C	0.006323
17	C	-0.016212
18	C	0.040908
19	C	-0.002442
20	C	0.001378
21	C	-0.000243
22	C	0.000461
23	C	-0.000205
24	C	0.001278
25	C	-0.015847
26	C	0.001813
27	C	0.001808
28	C	-0.015845
29	C	0.040903
30	C	-0.002441
31	C	-0.000243
32	C	0.001378
33	C	-0.016209
34	C	0.006326
35	C	-0.002522
36	C	-0.002026
37	C	0.019761
38	C	-0.002564
39	C	0.001829
40	C	-0.000519
41	C	0.001285
42	C	-0.012306
43	C	0.002575
44	C	0.002586
45	C	-0.012315
46	Cu	0.486575
47	F	0.000013
48	F	0.000053
49	F	0.000016
50	F	0.000034
51	F	0.000009
52	F	0.000013
69	N	0.116029
70	N	0.128063
71	N	0.116029
72	S	0.106334
73	F	0.000009
74	C	0.001119
75	C	-0.000486

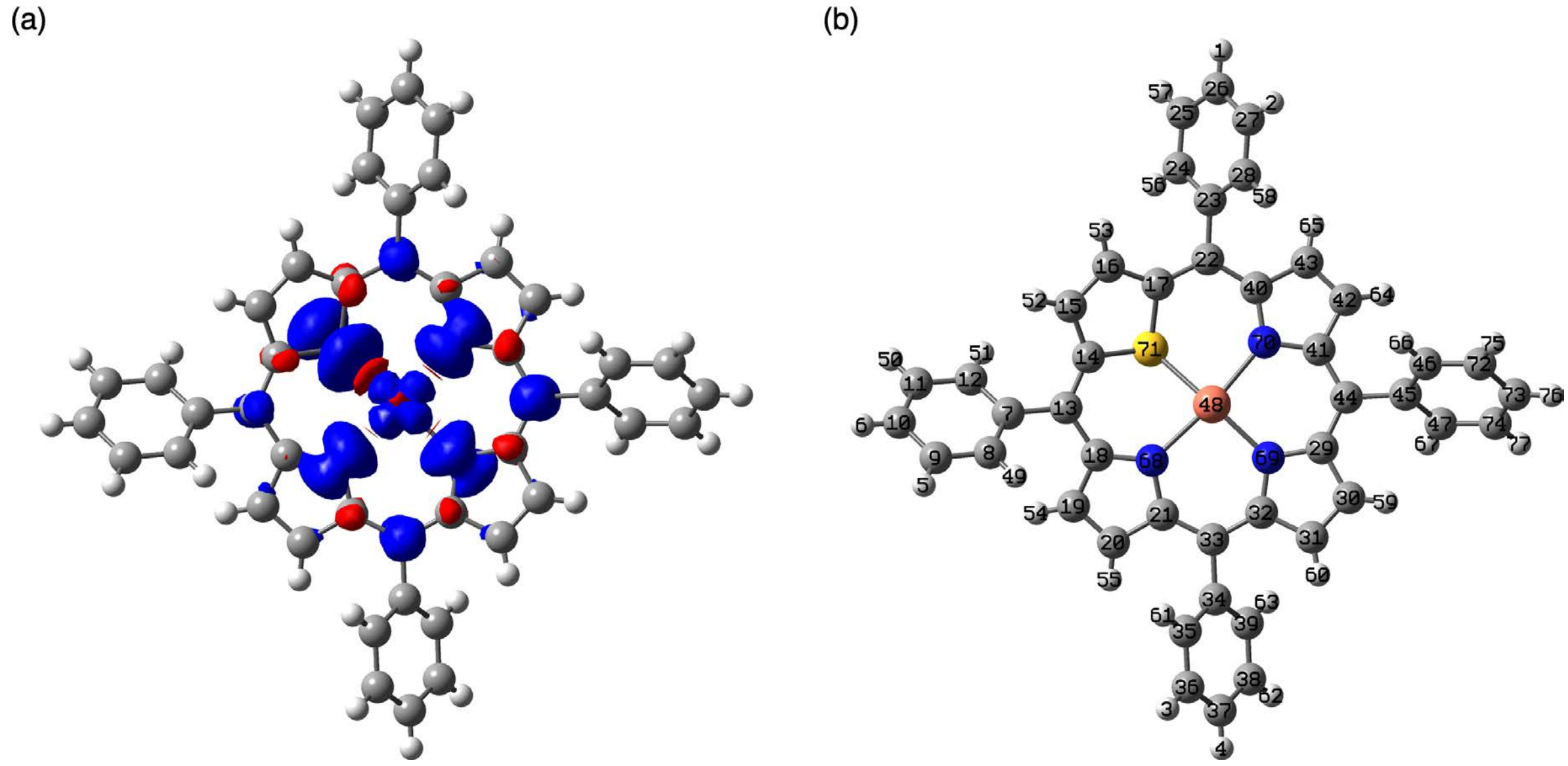

Fig. S59 Spin densities of **1cu**$^{+}$ from the crystal structure of **1cu**$^{+}$-BF_4^{-} estimated at UB3LYP/Def2TZVP: (a) spin-density mapping (δ = 0.001) and (b) molecular structures with atom labels provided for referencing the spin-density values (Table S10) and for discussions related to the molar magnetic susceptibility (Fig. S98).

Table S10 Spin densities, excluding hydrogen atoms of **1cu**$^{+}$ from the crystal structure of **1cu**$^{+}$-BF_4^{-} estimated at UB3LYP/Def2TZVP. Atom labels were referred to Fig. S59.

atom labels		spin densities
7	C	-0.003633
8	C	0.002449
9	C	-0.001085
10	C	0.002171
11	C	-0.001061
12	C	0.003105
13	C	0.025236
14	C	-0.013293
15	C	-0.000625
16	C	0.003038
17	C	-0.016535
18	C	-0.005343
19	C	-0.001495
20	C	0.004904
21	C	-0.016203
22	C	0.031816
23	C	-0.003545
24	C	0.002926
25	C	-0.001015
26	C	0.002211
27	C	-0.000973
28	C	0.002385
29	C	-0.015513
30	C	0.001492
31	C	0.000802
32	C	-0.014488
33	C	0.039536
34	C	-0.003513
35	C	0.003082
36	C	-0.001212
37	C	0.002484
38	C	-0.001002
39	C	0.002821
40	C	-0.006752
41	C	-0.017283
42	C	0.004793
43	C	-0.001184
44	C	0.043929
45	C	-0.003739
46	C	0.002980
47	C	0.003256
48	Cu	0.466089
68	N	0.120782
69	N	0.123982
70	N	0.122274
71	S	0.110555
72	C	-0.001228
73	C	0.002617
74	C	-0.000997

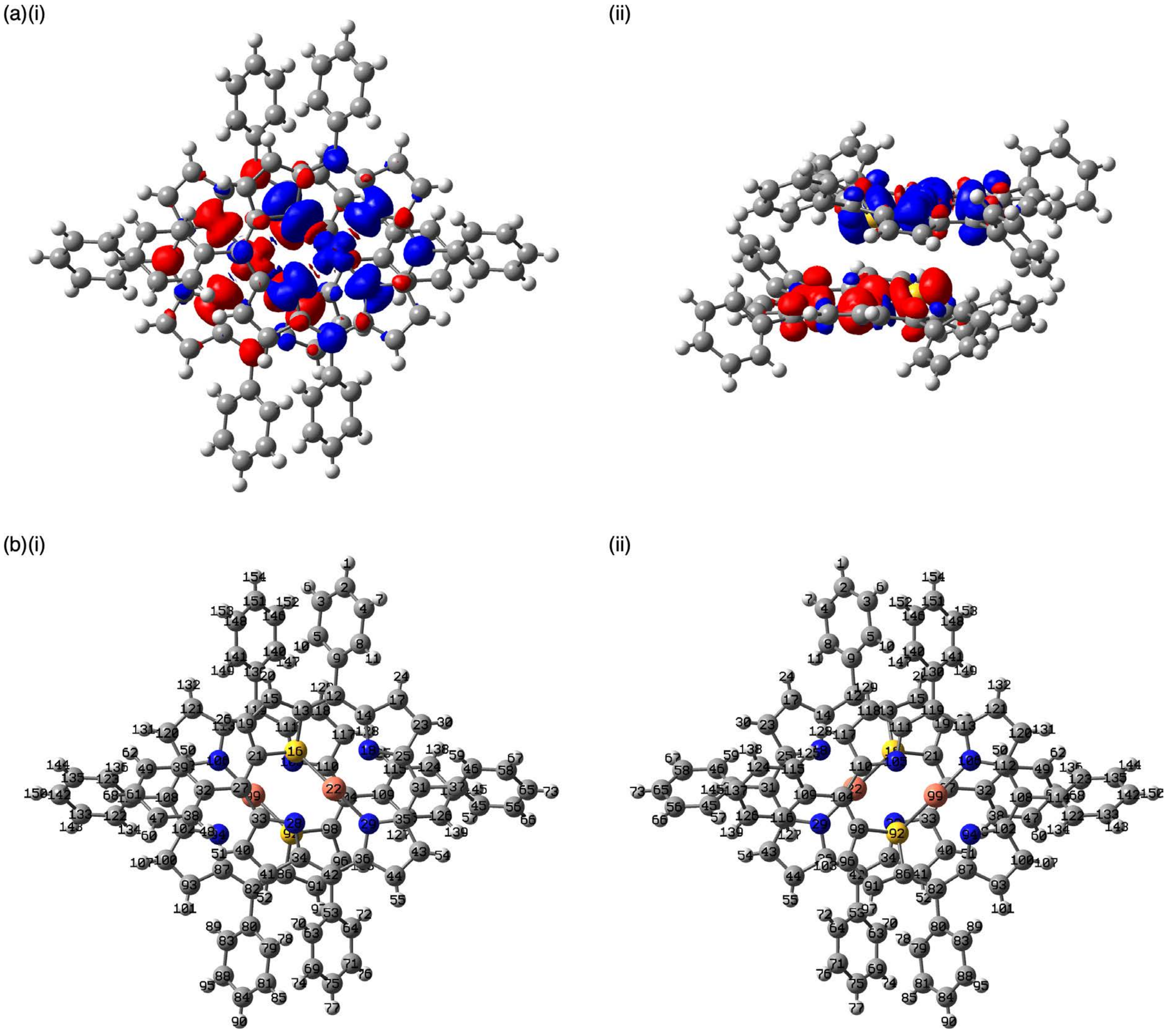

Fig. S60 Spin densities of dimeric **1cu**$^+$ from the crystal structure of **1cu**$^+$-BF_4^- estimated at GD3BJ-UB3LYP/Def2TZVP: (a) spin-density mapping (δ = 0.001) as (i) top and (ii) side views and (b) molecular structures with atom labels shown from (i) the same orientation as in (a)(i) and (ii) the opposite orientation, provided for referencing the spin-density values (Table S11) and for discussions related to the molar magnetic susceptibility (Fig. S98).

Table S11 Spin-density values, excluding hydrogen atoms of dimeric **1cu**$^{+}$ from the crystal structure of **1cu**$^{+}$-BF_4^{-} estimated at GD3BJ-UB3LYP/Def2TZVP. Atom labels were referred to Fig. S60.

atom labels		spin densities
2	C	0.002085
3	C	-0.000964
4	C	-0.000919
5	C	0.002832
8	C	0.002349
9	C	-0.003464
12	C	0.028117
13	C	-0.016961
14	C	-0.003703
15	C	0.002901
16	S	0.117658
17	C	-0.001185
18	N	0.119238
19	C	-0.000885
21	C	-0.013535
22	Cu	0.464043
23	C	0.005067
25	C	-0.015505
27	C	0.024368
28	N	0.116001
29	N	0.121841
31	C	0.040693
32	C	-0.003475
33	C	-0.004510
34	C	-0.012999
35	C	-0.013624
36	C	-0.012434
37	C	-0.003696
38	C	0.002408
39	C	0.003168
40	C	-0.001044
41	C	0.006550
42	C	0.038551
43	C	0.001246
44	C	0.000856
45	C	0.003167
46	C	0.003072
47	C	-0.001096
49	C	-0.001055
53	C	-0.003843
56	C	-0.000923
58	C	-0.001254
61	C	0.002150
63	C	0.003234
64	C	0.002635
65	C	0.002716
69	C	-0.001201
71	C	-0.000879
75	C	0.002385
79	C	-0.002839
80	C	0.003467
81	C	0.000967
82	C	-0.028161
83	C	-0.002357
84	C	-0.002097
86	C	0.017028
87	C	0.003773
88	C	0.000925
91	C	-0.002947
92	S	-0.117766
93	C	0.001137
94	N	-0.119231
96	C	0.000898
98	C	0.013535
99	Cu	-0.464087
100	C	-0.005041
102	C	0.015506
104	C	-0.024213
105	N	-0.116042
106	N	-0.121801
108	C	-0.040700
109	C	0.003463
110	C	0.004440
111	C	0.012961
112	C	0.013632
113	C	0.012382
114	C	0.003701
115	C	-0.002392
116	C	-0.003153
117	C	0.001047
118	C	-0.006562
119	C	-0.038426
120	C	-0.001277
121	C	-0.000841
122	C	-0.003079
123	C	-0.003184
124	C	0.001090
126	C	0.001047
130	C	0.003825
133	C	0.001254
135	C	0.000927
137	C	-0.002137
140	C	-0.003220
141	C	-0.002609
142	C	-0.002715
146	C	0.001193
148	C	0.000872
151	C	-0.002370

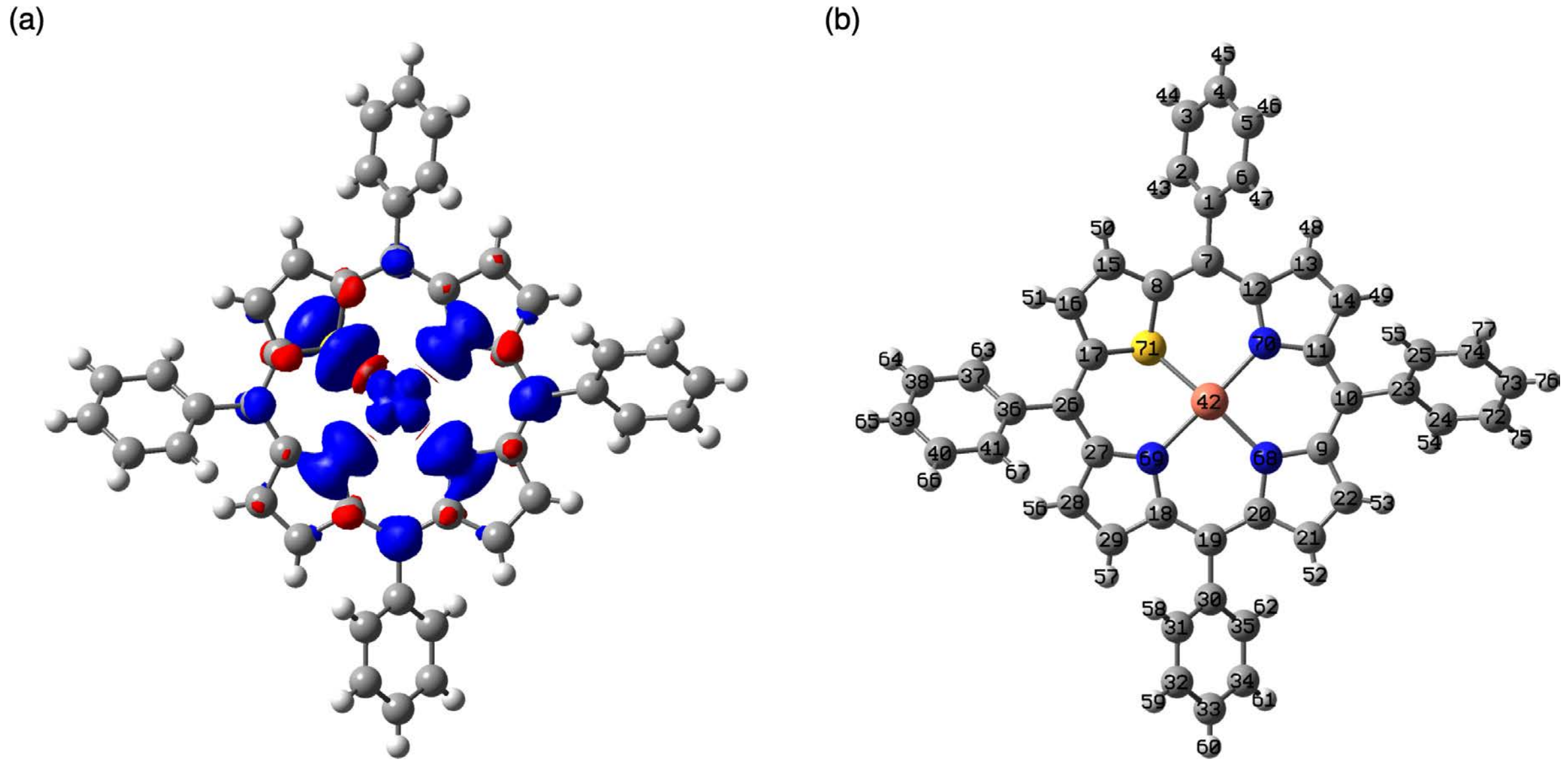

Fig. S61 Spin densities of **1cu**$^{+}$ with S1 thiophene from the crystal structure of **1cu**$^{+}$-PF_6^{-} estimated at UB3LYP/Def2TZVP: (a) spin-density mapping (δ = 0.001) and (b) molecular structures with atom labels provided for referencing the spin-density values (Table S12) and for discussions related to the molar magnetic susceptibility (Fig. S98).

Table S12 Spin densities, excluding hydrogen atoms of **1cu**$^{+}$ with S1 thiophene unit from the crystal structure of **1cu**$^{+}$-PF_6^{-} estimated at UB3LYP/Def2TZVP. Atom labels were referred to Fig. S61.

atom labels		spin densities
1	C	-0.002320
2	C	0.001783
3	C	-0.000478
4	C	0.000982
5	C	-0.000407
6	C	0.001012
7	C	0.018587
8	C	-0.012878
9	C	-0.013574
10	C	0.041094
11	C	-0.018622
12	C	-0.004783
13	C	-0.003501
14	C	0.006754
15	C	0.003054
16	C	0.006739
17	C	-0.014747
18	C	-0.016630
19	C	0.042120
20	C	-0.012294
21	C	0.000686
22	C	0.000811
23	C	-0.003569
24	C	0.003313
25	C	0.003220
26	C	0.023536
27	C	-0.005141
28	C	-0.003519
29	C	0.005571
30	C	-0.003759
31	C	0.003774
32	C	-0.001471
33	C	0.003537
34	C	-0.001431
35	C	0.003331
36	C	-0.003486
37	C	0.002959
38	C	-0.000974
39	C	0.001705
40	C	-0.000883
41	C	0.002111
42	Cu	0.459412
68	N	0.130757
69	N	0.131113
70	N	0.120838
71	S	0.105211
72	C	-0.001284
73	C	0.003073
74	C	-0.001336

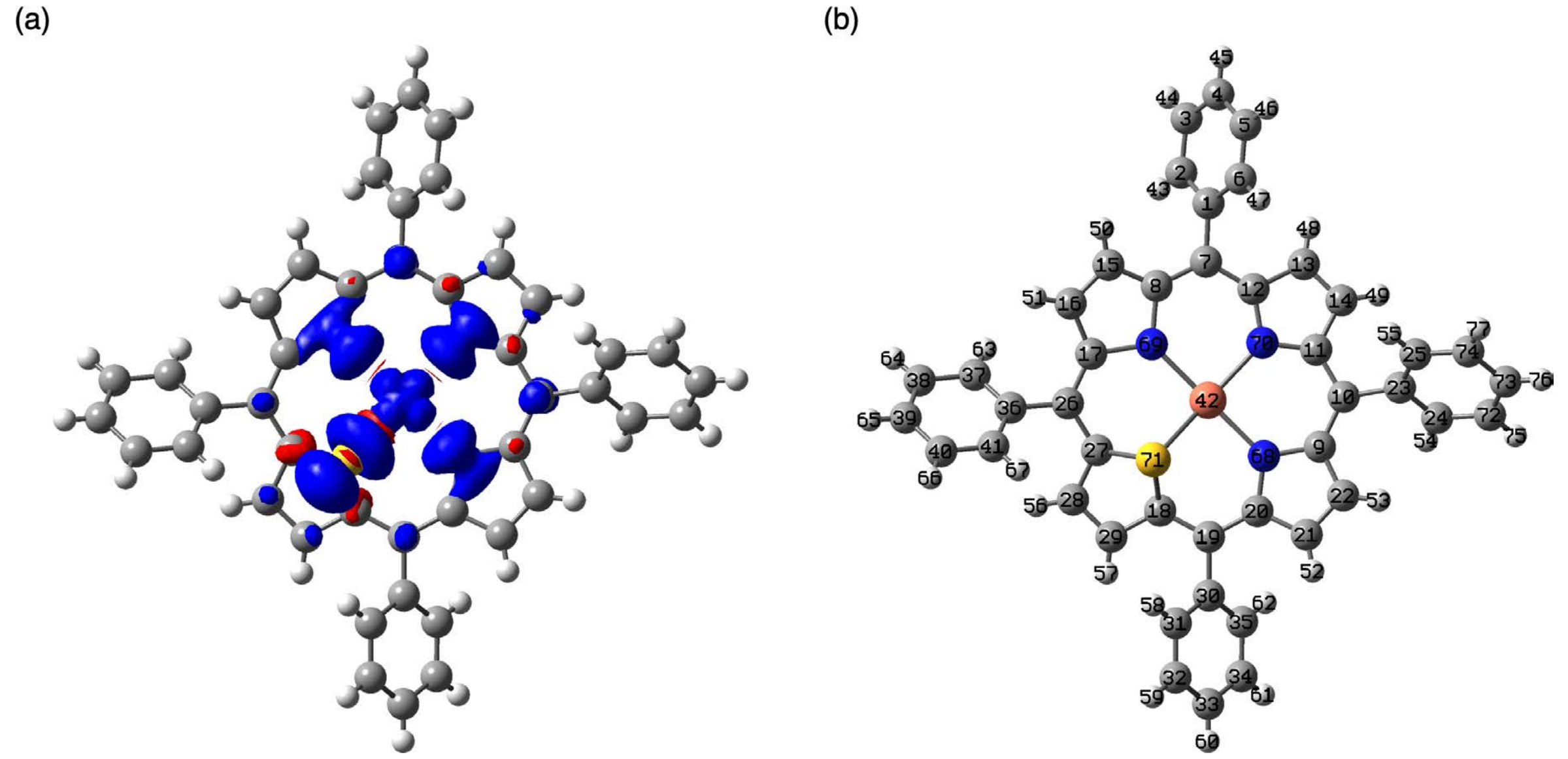

Fig. S62 Spin densities of **1cu**$^{+}$ with S2 thiophene from the crystal structure of **1cu**$^{+}$-PF_6^{-} estimated at UB3LYP/Def2TZVP: (a) spin-density mapping (δ = 0.001) and (b) molecular structures with atom labels provided for referencing the spin-density values (Table S13) and for discussions related to the molar magnetic susceptibility (Fig. S98).

Table S13 Spin densities, excluding hydrogen atoms of **1cu**$^{+}$ with S2 thiophene unit from the crystal structure of **1cu**$^{+}$-PF_6^{-} estimated at UB3LYP/Def2TZVP. Atom labels were referred to Fig. S62.

atom labels		spin densities
1	C	-0.002079
2	C	0.001673
3	C	-0.000626
4	C	0.001324
5	C	-0.000451
6	C	0.001423
7	C	0.021776
8	C	-0.005040
9	C	-0.010464
10	C	0.023813
11	C	-0.009887
12	C	-0.009824
13	C	0.002478
14	C	0.002430
15	C	0.002706
16	C	-0.001731
17	C	0.000665
18	C	-0.011952
19	C	0.010731
20	C	-0.001927
21	C	-0.001306
22	C	0.002892
23	C	-0.002081
24	C	0.001801
25	C	0.001519
26	C	0.010961
27	C	-0.014488
28	C	0.003537
29	C	0.004106
30	C	-0.001479
31	C	0.001283
32	C	-0.000282
33	C	0.000886
34	C	-0.000447
35	C	0.001127
36	C	-0.000677
37	C	0.001303
38	C	-0.000520
39	C	0.000798
40	C	-0.000253
41	C	0.001350
42	Cu	0.491833
68	N	0.115560
69	N	0.120208
70	N	0.122052
71	S	0.125111
72	C	-0.000724
73	C	0.001422
74	C	-0.000531

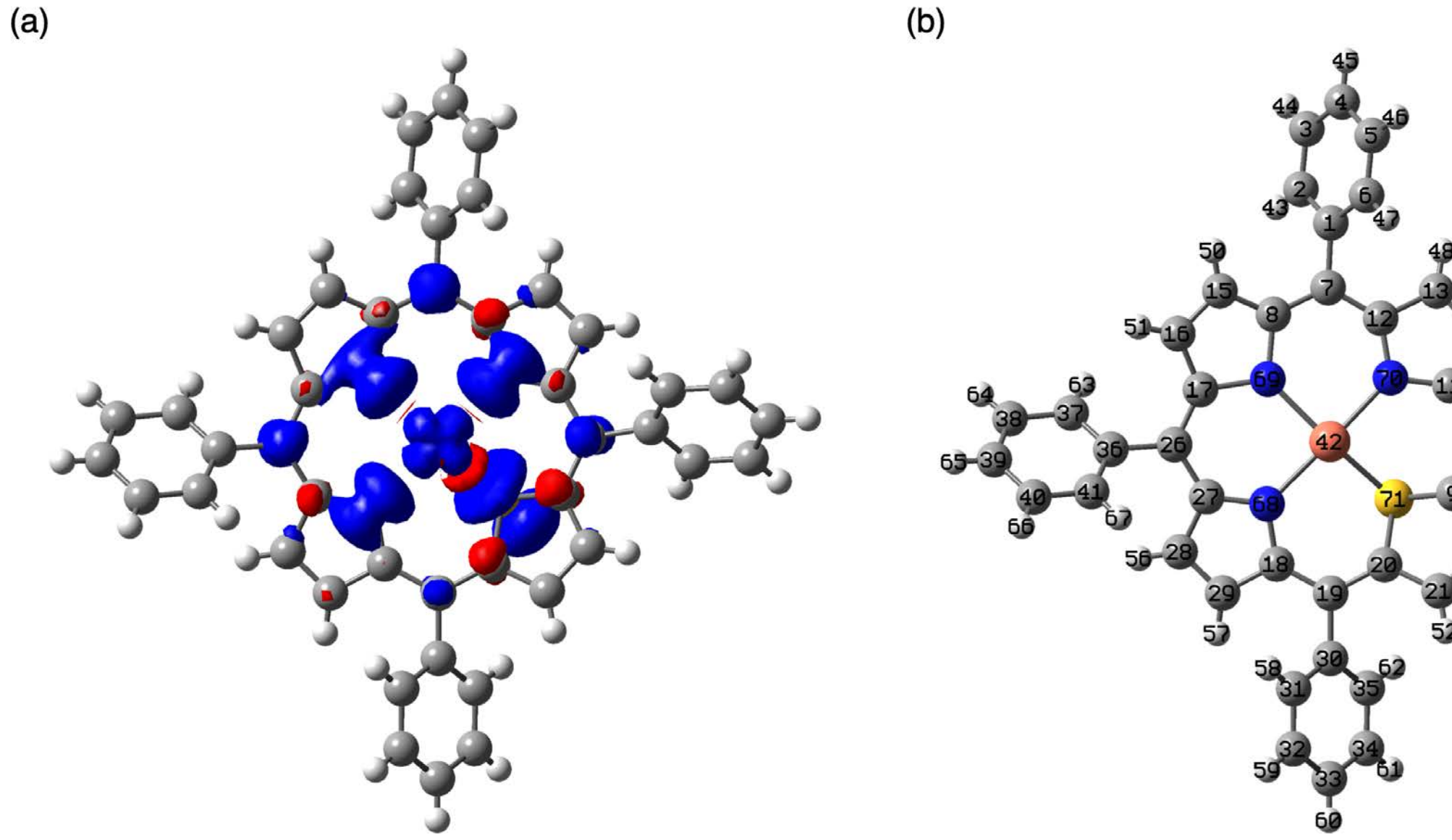

Fig. S63 Spin densities of **1cu**$^{+}$ with S3 thiophene from the crystal structure of **1cu**$^{+}$-PF_6^{-} estimated at UB3LYP/Def2TZVP: (a) spin-density mapping (δ = 0.001) and (b) molecular structures with atom labels provided for referencing the spin-density values (Table S14) and for discussions related to the molar magnetic susceptibility (Fig. S98).

Table S14 Spin densities, excluding hydrogen atoms of **1cu**$^{+}$ with S3 thiophene unit from the crystal structure of **1cu**$^{+}$-PF_6^{-} estimated at UB3LYP/Def2TZVP. Atom labels were referred to Fig. S63.

atom labels		spin densities
1	C	-0.003784
2	C	0.003262
3	C	-0.001177
4	C	0.002803
5	C	-0.001076
6	C	0.002981
7	C	0.041674
8	C	-0.008345
9	C	-0.018550
10	C	0.025175
11	C	-0.007039
12	C	-0.017917
13	C	0.005730
14	C	-0.001891
15	C	0.001229
16	C	-0.001114
17	C	-0.007953
18	C	-0.004414
19	C	0.014708
20	C	-0.016045
21	C	0.000911
22	C	0.005436
23	C	-0.003151
24	C	0.002355
25	C	0.001721
26	C	0.034287
27	C	-0.016387
28	C	0.006156
29	C	-0.004473
30	C	-0.002424
31	C	0.001493
32	C	-0.000596
33	C	0.001272
34	C	-0.000504
35	C	0.001605
36	C	-0.003167
37	C	0.003384
38	C	-0.001429
39	C	0.002848
40	C	-0.001263
41	C	0.003282
42	Cu	0.453375
68	N	0.118230
69	N	0.137736
70	N	0.138164
71	S	0.112796
72	C	-0.000761
73	C	0.001570
74	C	-0.000725

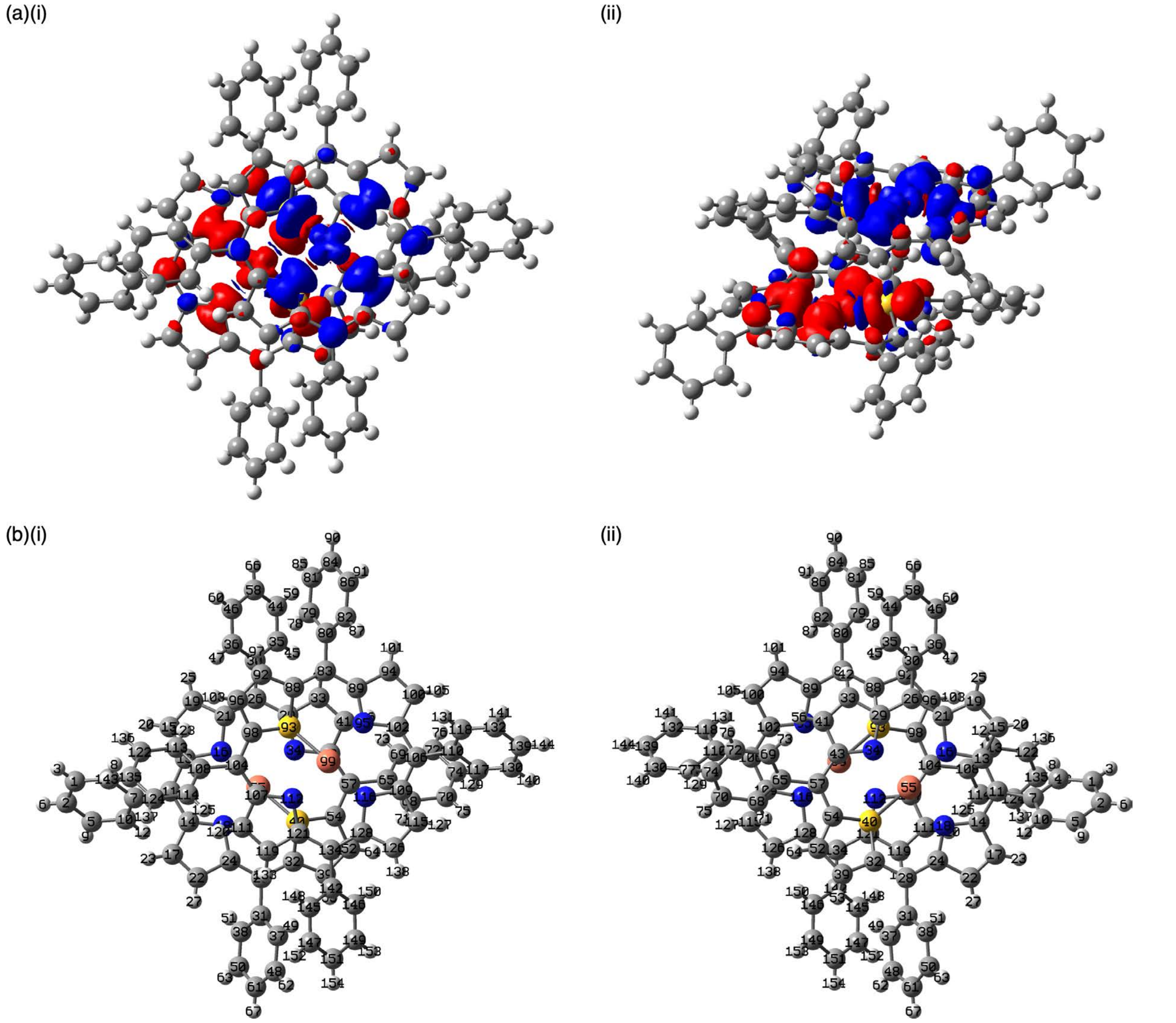

Fig. S64 Spin densities of dimeric **1cu**$^{+}$ from the crystal structure of **1cu**$^{+}$-PF_6^{-} as S1-S1 combination estimated at GD3BJ-UB3LYP/Def2TZVP: (a) spin-density mapping (δ = 0.001) as (i) top and (ii) side views and (b) molecular structures with atom labels shown from (i) the same orientation as in (a)(i) and (ii) the opposite orientation, provided for referencing the spin-density values (Table S15) and for discussions related to the molar magnetic susceptibility (Fig. S98).

Table S15 Spin-density values, excluding hydrogen atoms of dimeric **1cu**$^{+}$ from the crystal structure of **1cu**$^{+}$-PF_6^{-} as S1-S1 combination estimated at GD3BJ-UB3LYP/Def2TZVP. Atom labels were referred to Fig. S64.

atom labels		spin densities
1	C	0.001144
2	C	-0.003119
4	C	-0.003099
5	C	0.001335
7	C	0.003273
10	C	-0.003182
11	C	-0.036601
13	C	0.010062
14	C	0.016595
15	C	0.000409
16	N	-0.127450
17	C	-0.007234
18	N	-0.117785
19	C	-0.001826
21	C	0.011869
22	C	0.003821
24	C	0.001357
26	C	-0.041049
28	C	-0.014272
29	C	0.008184
30	C	0.004172
31	C	0.002229
32	C	0.012308
33	C	-0.006761
34	N	-0.123315
35	C	-0.004100
36	C	-0.003109
37	C	-0.001697
38	C	-0.000831
39	C	-0.002865
40	S	-0.112926
41	C	0.003472
43	C	0.003638
44	C	0.001476
46	C	0.001340
48	C	0.000421
50	C	0.000296
52	C	-0.007231
54	C	0.014381
55	Cu	-0.456952
57	C	-0.021468
58	C	-0.003514
61	C	-0.000697
65	C	0.003186
68	C	-0.002779
69	C	-0.001962
70	C	0.000760
72	C	0.000807
74	C	-0.001516
79	C	0.001699
80	C	-0.002237
81	C	-0.000423
82	C	0.000839
83	C	0.014347
84	C	0.000703
86	C	-0.000299
88	C	-0.012346
89	C	-0.001405
92	C	0.002915
93	S	0.112988
94	C	-0.003774
95	N	0.117800
96	C	0.007168
98	C	-0.014394
99	Cu	0.456855
100	C	0.007226
102	C	-0.016616
104	C	0.021562
106	C	0.036622
107	C	-0.003629
108	C	-0.003183
109	C	-0.010056
110	C	-0.003279
111	C	-0.003469
112	N	0.123413
113	C	0.002781
114	C	0.001972
115	C	-0.000420
116	N	0.127444
117	C	0.003107
118	C	0.003181
119	C	0.006765
121	C	-0.008176
122	C	-0.000764
124	C	-0.000813
126	C	0.001816
128	C	-0.011900
130	C	-0.001146
132	C	-0.001338
134	C	0.040911
135	C	0.001529
139	C	0.003123
142	C	-0.004143
145	C	0.004075
146	C	0.003081
147	C	-0.001466
149	C	-0.001330
151	C	0.003487

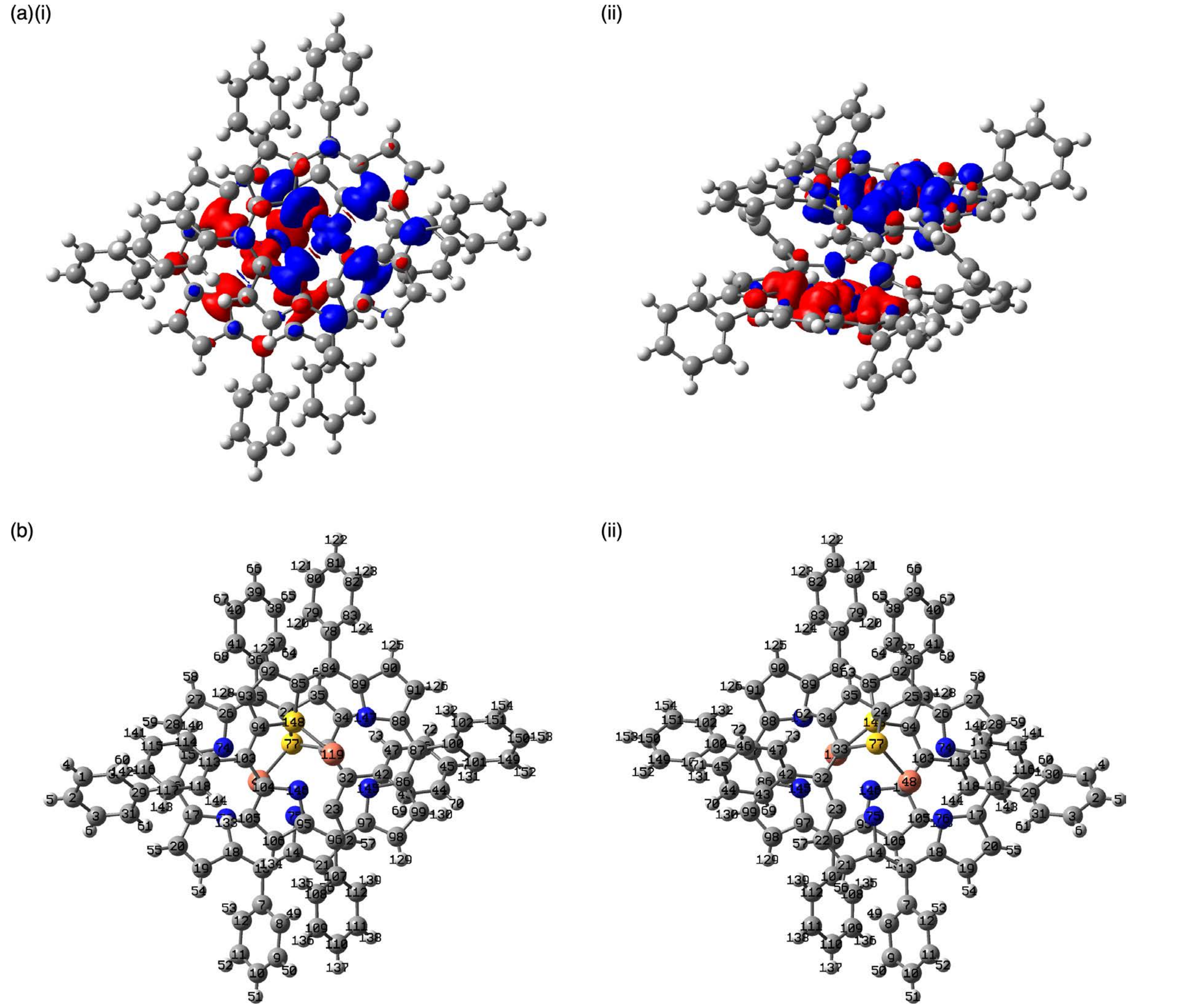

Fig. S65 Spin densities of dimeric **1cu**$^{+}$ from the crystal structure of **1cu**$^{+}$-PF_6^{-} as S1-S2 combination estimated at GD3BJ-UB3LYP/Def2TZVP: (a) spin-density mapping (δ = 0.001) as (i) top and (ii) side views and (b) molecular structures with atom labels shown from (i) the same orientation as in (a)(i) and (ii) the opposite orientation, provided for referencing the spin-density values (Table S16) and for discussions related to the molar magnetic susceptibility (Fig. S98).

Table S16 Spin-density values, excluding hydrogen atoms of dimeric **1cu**$^{+}$ from the crystal structure of **1cu**$^{+}$-PF_6^{-} as S1-S2 combination estimated at GD3BJ-UB3LYP/Def2TZVP. Atom labels were referred to Fig. S65.

atom labels		spin densities
1	C	0.000793
2	C	-0.001649
3	C	0.000585
7	C	0.002180
8	C	-0.001684
9	C	0.000700
10	C	-0.001491
11	C	0.000485
12	C	-0.001469
13	C	-0.021980
14	C	0.003396
15	C	0.009697
16	C	-0.024592
17	C	0.009982
18	C	0.007468
19	C	-0.002273
20	C	-0.002267
21	C	-0.002058
22	C	0.001402
23	C	-0.001365
24	C	0.009167
25	C	-0.011278
26	C	0.002517
27	C	0.001387
28	C	-0.002443
29	C	0.002202
30	C	-0.001955
31	C	-0.001652
32	C	-0.012259
33	C	0.016807
34	C	-0.002600
35	C	-0.005336
36	C	0.001223
37	C	-0.001720
38	C	0.000405
39	C	-0.001180
40	C	0.000502
41	C	-0.001000
42	C	0.000580
43	C	-0.001432
44	C	0.000527
45	C	-0.000909
46	C	0.000302
47	C	-0.001479
48	Cu	-0.488878
74	N	-0.114420
75	N	-0.120635
76	N	-0.119794
77	S	-0.123685
78	C	-0.002314
79	C	0.001957
80	C	-0.000503
81	C	0.001010
82	C	-0.000416
83	C	0.001109
84	C	0.017661
85	C	-0.013427
86	C	-0.011900
87	C	0.039884
88	C	-0.017154
89	C	-0.002992
90	C	-0.003435
91	C	0.006708
92	C	0.002374
93	C	0.006231
94	C	-0.014793
95	C	-0.013408
96	C	0.040974
97	C	-0.010293
98	C	0.000713
99	C	0.000430
100	C	-0.003503
101	C	0.003325
102	C	0.003328
103	C	0.022871
104	C	-0.004199
105	C	-0.003373
106	C	0.006013
107	C	-0.004046
108	C	0.003758
109	C	-0.001401
110	C	0.003339
111	C	-0.001317
112	C	0.003214
113	C	-0.003271
114	C	0.002891
115	C	-0.000819
116	C	0.001622
117	C	-0.000852
118	C	0.002108
119	Cu	0.457022
145	N	0.128925
146	N	0.127142
147	N	0.117664
148	S	0.111698
149	C	-0.001259
150	C	0.003265
151	C	-0.001377

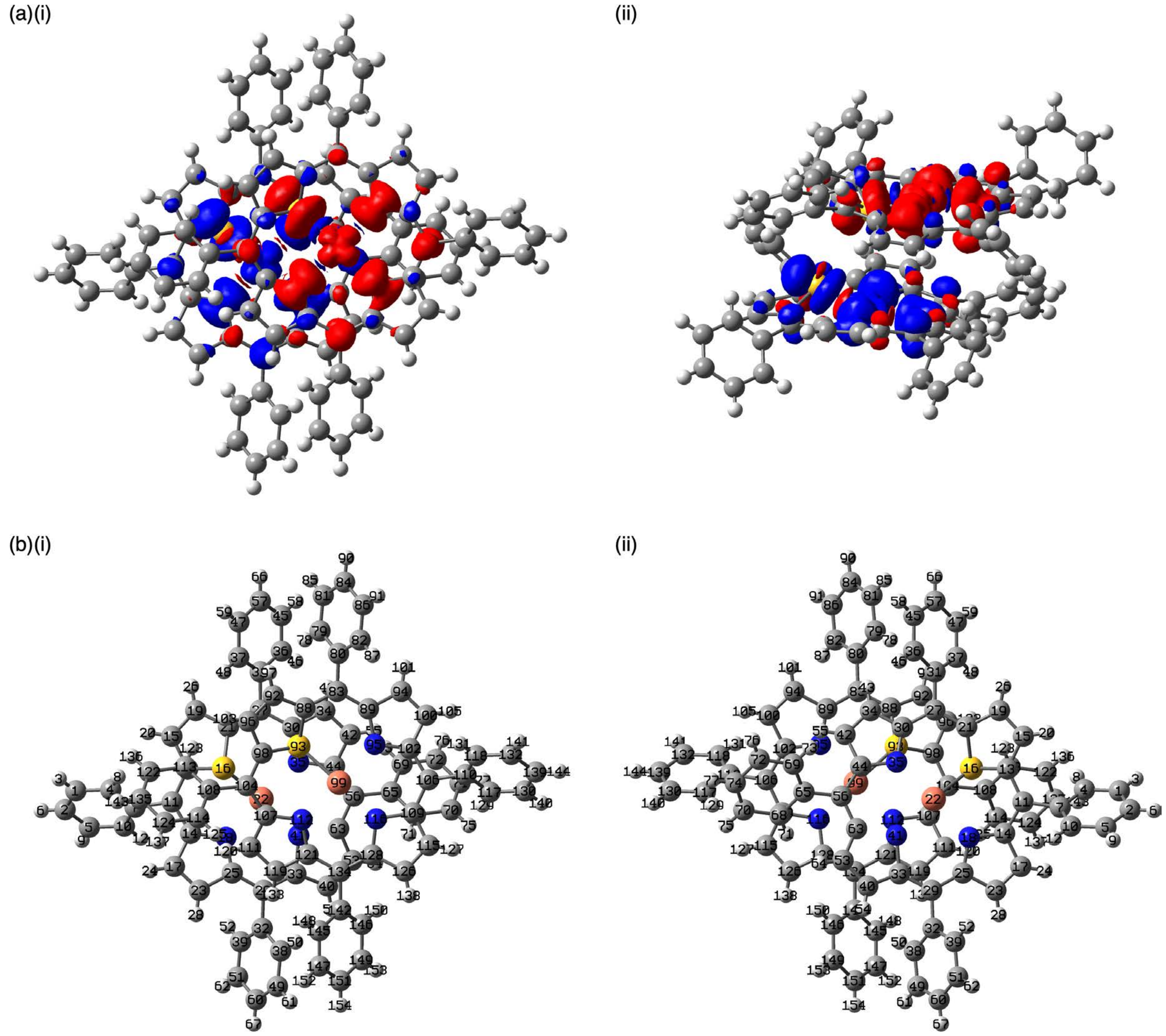

Fig. S66 Spin densities of dimeric **1cu**$^{+}$ from the crystal structure of **1cu**$^{+}$-PF_6^{-} as S1-S3 combination estimated at GD3BJ-UB3LYP/Def2TZVP: (a) spin-density mapping (δ = 0.001) as (i) top and (ii) side views and (b) molecular structures with atom labels shown from (i) the same orientation as in (a)(i) and (ii) the opposite orientation, provided for referencing the spin-density values (Table S17) and for discussions related to the molar magnetic susceptibility (Fig. S98).

Table S17 Spin-density values, excluding hydrogen atoms of dimeric **1cu**$^+$ from the crystal structure of **1cu**$^+$-PF_6^- as S1-S3 combination estimated at GD3BJ-UB3LYP/Def2TZVP. Atom labels were referred to Fig. S66.

atom labels		spin densities
1	C	-0.000665
2	C	0.001313
4	C	0.002216
5	C	-0.000596
7	C	-0.003168
10	C	0.001443
11	C	0.021965
13	C	-0.019513
14	C	-0.004985
15	C	0.005819
16	S	0.116922
17	C	-0.002357
18	N	0.135155
19	C	-0.000270
21	C	-0.014804
22	Cu	0.451213
23	C	0.006219
25	C	-0.014678
27	C	0.011654
29	C	0.039112
30	C	0.002495
31	C	-0.002597
32	C	-0.003650
33	C	-0.005329
34	C	-0.002955
35	N	0.112709
36	C	0.001710
37	C	0.001486
38	C	0.003007
39	C	0.002912
40	C	-0.000462
41	N	0.137681
42	C	0.005840
44	C	-0.015978
45	C	-0.000672
47	C	-0.000470
49	C	-0.001135
51	C	-0.001104
53	C	0.000067
56	C	0.035677
57	C	0.001405
60	C	0.002916
63	C	-0.007634
65	C	-0.003145
68	C	0.003442
69	C	0.003461
70	C	-0.001345
72	C	-0.001277
74	C	0.002967
79	C	-0.001854
80	C	0.002307
81	C	0.000481
82	C	-0.000984
83	C	-0.016564
84	C	-0.000872
86	C	0.000357
88	C	0.013221
89	C	0.002349
92	C	-0.003580
93	S	-0.111287
94	C	0.003550
95	N	-0.117692
96	C	-0.006039
98	C	0.012993
99	Cu	-0.458009
100	C	-0.006804
102	C	0.016882
104	C	-0.024963
106	C	-0.038346
107	C	0.006010
108	C	0.003545
109	C	0.011134
110	C	0.003424
111	C	0.004485
112	N	-0.127518
113	C	-0.002488
114	C	-0.002454
115	C	0.000212
116	N	-0.128923
117	C	-0.003227
118	C	-0.003241
119	C	-0.006458
121	C	0.013841
122	C	0.001278
124	C	0.001299
126	C	-0.001435
128	C	0.010571
130	C	0.001208
132	C	0.001345
134	C	-0.041139
135	C	-0.001856
139	C	-0.003172
142	C	0.004004
145	C	-0.003755
146	C	-0.003217
147	C	0.001390
149	C	0.001338
151	C	-0.003372

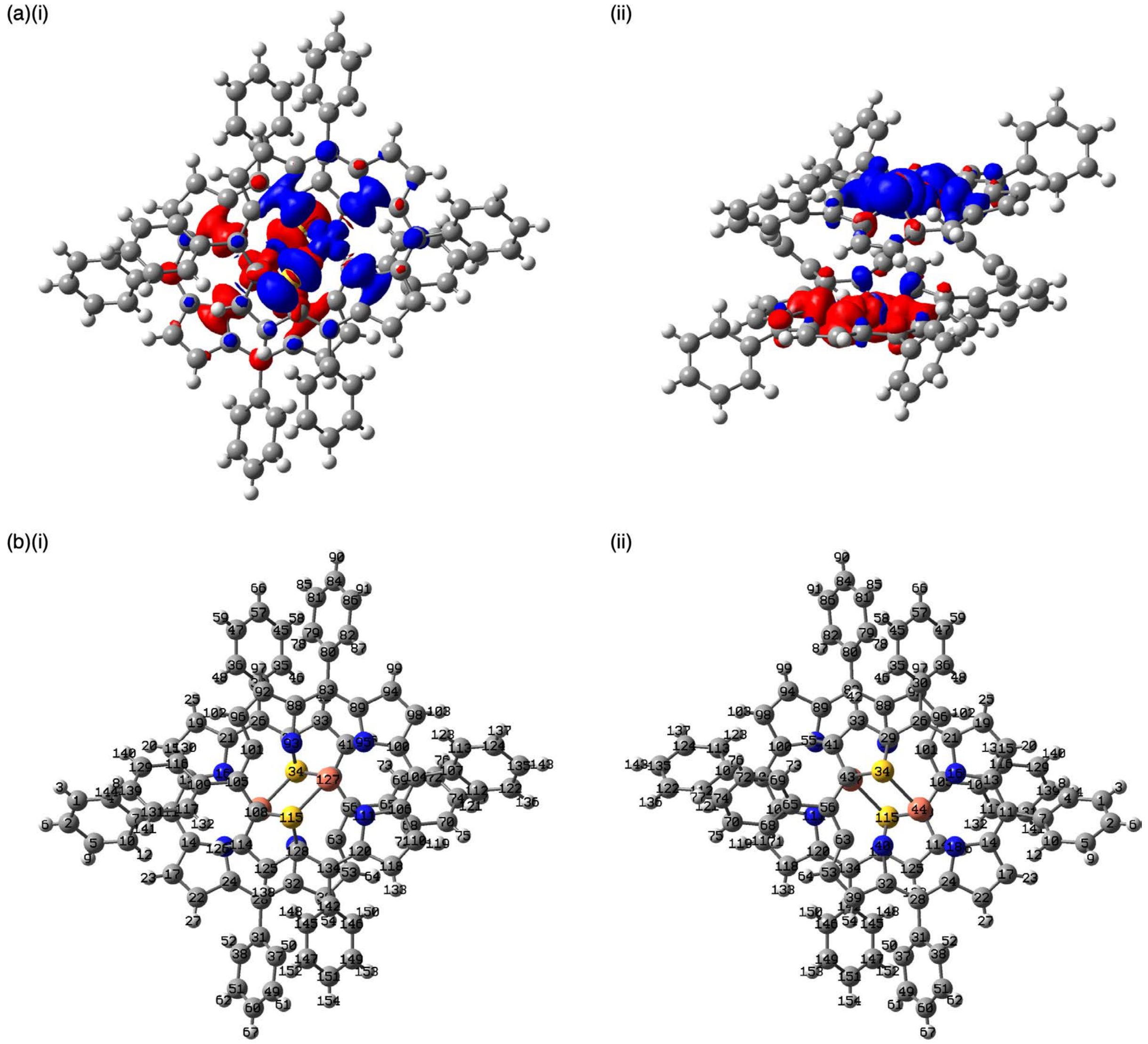

Fig. S67 Spin densities of dimeric **1cu**$^{+}$ from the crystal structure of **1cu**$^{+}$-PF_6^{-} as S2-S2 combination estimated at GD3BJ-UB3LYP/Def2TZVP: (a) spin-density mapping (δ = 0.001) as (i) top and (ii) side views and (b) molecular structures with atom labels shown from (i) the same orientation as in (a)(i) and (ii) the opposite orientation, provided for referencing the spin-density values (Table S18) and for discussions related to the molar magnetic susceptibility (Fig. S98).

Table S18 Spin-density values, excluding hydrogen atoms of dimeric **1cu**$^{+}$ from the crystal structure of **1cu**$^{+}$-PF_6^{-} as S2-S2 combination estimated at GD3BJ-UB3LYP/Def2TZVP. Atom labels were referred to Fig. S67.

atom labels		spin densities
1	C	0.000735
2	C	-0.001484
4	C	-0.001821
5	C	0.000526
7	C	0.002167
10	C	-0.001527
11	C	-0.023443
13	C	0.009246
14	C	0.009327
15	C	-0.002561
16	N	-0.114392
17	C	-0.002342
18	N	-0.117642
19	C	0.001362
21	C	0.000993
22	C	-0.002353
24	C	0.006800
26	C	-0.010626
28	C	-0.020041
29	C	0.012186
30	C	0.001174
31	C	0.002058
32	C	0.002670
33	C	-0.006687
34	S	-0.125599
35	C	-0.001300
36	C	-0.001068
37	C	-0.001553
38	C	-0.001387
39	C	-0.003050
40	N	-0.118385
41	C	-0.001604
43	C	0.012685
44	Cu	-0.491564
45	C	0.000302
47	C	0.000464
49	C	0.000638
51	C	0.000438
53	C	0.002047
56	C	-0.009877
57	C	-0.000931
60	C	-0.001353
63	C	-0.001815
65	C	0.000257
68	C	-0.001250
69	C	-0.001270
70	C	0.000462
72	C	0.000235
74	C	-0.000727
79	C	0.001545
80	C	-0.002055
81	C	-0.000636
82	C	0.001390
83	C	0.020037
84	C	0.001349
86	C	-0.000437
88	C	-0.002659
89	C	-0.006821
92	C	0.002987
93	N	0.118379
94	C	0.002390
95	N	0.117535
96	C	-0.001997
98	C	0.002344
100	C	-0.009313
101	C	0.001782
104	C	0.023401
105	C	0.009939
106	C	-0.009224
107	C	-0.002165
108	C	-0.012816
109	C	-0.000267
110	C	0.002571
111	N	0.114350
112	C	0.001819
113	C	0.001522
114	C	0.001686
115	C	0.125857
116	C	0.001251
117	C	0.001275
118	C	-0.001368
120	C	-0.001002
122	C	-0.000733
124	C	-0.000525
125	C	0.006539
127	Cu	0.491646
128	C	-0.012076
129	C	-0.000465
131	C	-0.000237
134	C	0.010518
135	C	0.001481
139	C	0.000735
142	C	-0.001154
145	C	0.001285
146	C	0.001053
147	C	-0.000296
149	C	-0.000458
151	C	0.000915

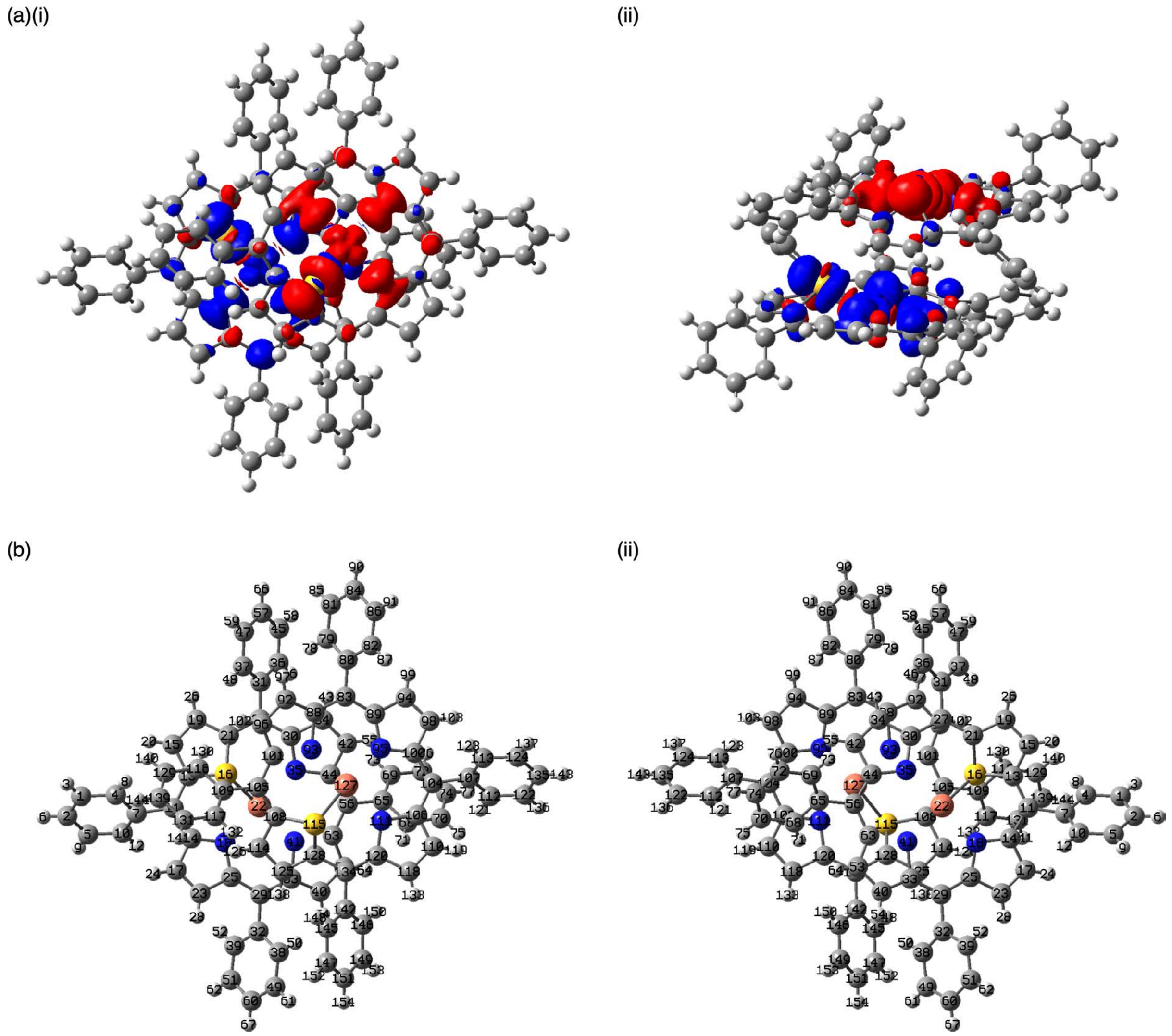

Fig. S68 Spin densities of dimeric **1cu**$^{+}$ from the crystal structure of **1cu**$^{+}$-PF_6^{-} as S2-S3 combination estimated at GD3BJ-UB3LYP/Def2TZVP: (a) spin-density mapping (δ = 0.001) as (i) top and (ii) side views and (b) molecular structures with atom labels shown from (i) the same orientation as in (a)(i) and (ii) the opposite orientation, provided for referencing the spin-density values (Table S19) and for discussions related to the molar magnetic susceptibility (Fig. S98).

Table S19 Spin-density values, excluding hydrogen atoms of dimeric **1cu**$^{+}$ from the crystal structure of **1cu**$^{+}$-PF_6^{-} as S2-S3 combination estimated at GD3BJ-UB3LYP/Def2TZVP. Atom labels were referred to Fig. S68.

atom labels		spin densities
1	C	-0.000661
2	C	0.001337
4	C	0.002230
5	C	-0.000606
7	C	-0.003175
10	C	0.001475
11	C	0.022110
13	C	-0.018619
14	C	-0.004245
15	C	0.005204
16	S	0.117219
17	C	-0.002219
18	N	0.133428
19	C	0.000838
21	C	-0.015548
22	Cu	0.451624
23	C	0.006302
25	C	-0.013933
27	C	0.013216
29	C	0.038845
30	C	-0.001936
31	C	-0.002688
32	C	-0.003673
33	C	-0.005177
34	C	-0.003604
35	N	0.115604
36	C	0.001565
37	C	0.001672
38	C	0.003006
39	C	0.002919
40	C	0.001166
41	N	0.135361
42	C	0.006012
44	C	-0.015583
45	C	-0.000632
47	C	-0.000512
49	C	-0.001118
51	C	-0.001068
53	C	-0.000896
56	C	0.034609
57	C	0.001324
60	C	0.002860
63	C	-0.006814
65	C	-0.003026
68	C	0.003293
69	C	0.003342
70	C	-0.001280
72	C	-0.001242
74	C	0.002841
79	C	-0.001580
80	C	0.002114
81	C	0.000651
82	C	-0.001381
83	C	-0.020623
84	C	-0.001365
86	C	0.000448
88	C	0.002773
89	C	0.007086
92	C	-0.002590
93	N	-0.118113
94	C	-0.002292
95	N	-0.118543
96	C	0.001605
98	C	-0.002383
100	C	0.009584
101	C	-0.003055
104	C	-0.023289
105	C	-0.011440
106	C	0.009453
107	C	0.002147
108	C	0.012739
109	C	0.000482
110	C	-0.002559
111	N	-0.114518
112	C	-0.001814
113	C	-0.001504
114	C	-0.000441
115	S	-0.124042
116	C	-0.000610
117	C	-0.001476
118	C	0.001441
120	C	0.000857
122	C	0.000734
124	C	0.000516
125	C	-0.007559
127	Cu	-0.492942
128	C	0.012488
129	C	0.000955
131	C	0.000649
134	C	-0.010056
135	C	-0.001471
139	C	-0.000906
142	C	0.001144
145	C	-0.001248
146	C	-0.001032
147	C	0.000276
149	C	0.000433
151	C	-0.000888

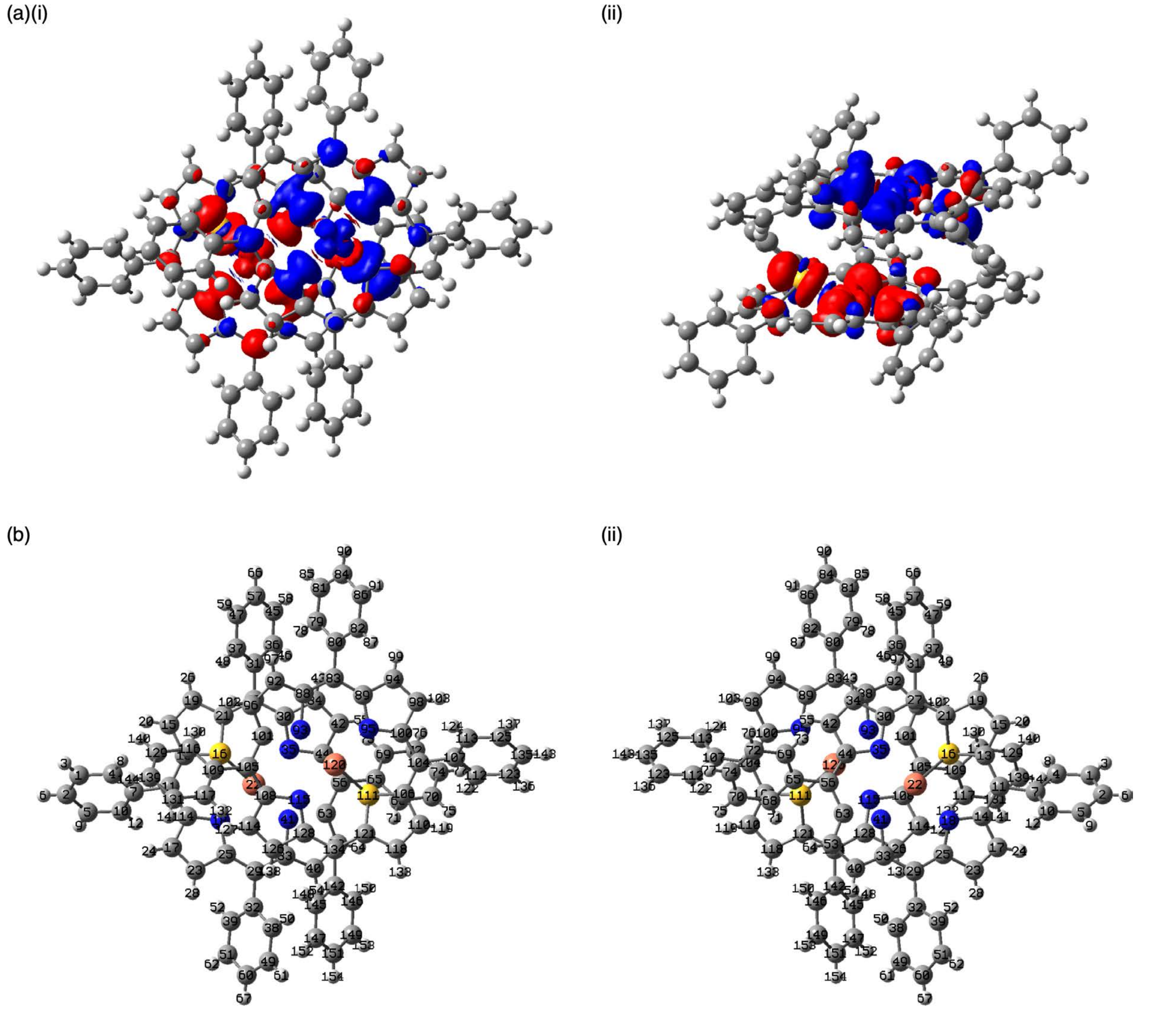

Fig. S69 Spin densities of dimeric **1cu**$^+$ from the crystal structure of **1cu**$^+$-PF_6^- as S3-S3 combination estimated at GD3BJ-UB3LYP/Def2TZVP: (a) spin-density mapping ($\delta = 0.001$) as (i) top and (ii) side views and (b) molecular structures with atom labels shown from (i) the same orientation as in (a)(i) and (ii) the opposite orientation, provided for referencing the spin-density values (Table S20) and for discussions related to the molar magnetic susceptibility (Fig. S98).

Table S20 Spin-density values, excluding hydrogen atoms of dimeric **1cu**$^{+}$ from the crystal structure of **1cu**$^{+}$-PF_6^{-} as S3-S3 combination estimated at GD3BJ-UB3LYP/Def2TZVP. Atom labels were referred to Fig. S69.

atom labels		spin densities
1	C	0.000643
2	C	-0.001246
4	C	-0.002166
5	C	0.000565
7	C	0.003141
10	C	-0.001384
11	C	-0.021227
13	C	0.018901
14	C	0.004317
15	C	-0.006024
16	S	-0.116357
17	C	0.002327
18	N	-0.133545
19	C	-0.000672
21	C	0.014553
22	Cu	-0.452995
23	C	-0.006263
25	C	0.014220
27	C	-0.011887
29	C	-0.038921
30	C	0.001433
31	C	0.002579
32	C	0.003699
33	C	0.004752
34	C	0.003073
35	N	-0.114787
36	C	-0.001419
37	C	-0.001583
38	C	-0.003009
39	C	-0.002916
40	C	-0.000239
41	N	-0.135756
42	C	-0.005062
44	C	0.015564
45	C	0.000567
47	C	0.000468
49	C	0.001109
51	C	0.001094
53	C	0.000161
56	C	-0.035512
57	C	-0.001219
60	C	-0.002868
63	C	0.005774
65	C	0.003315
68	C	-0.002698
69	C	-0.003447
70	C	0.001720
72	C	0.001587
74	C	-0.002927
79	C	0.002987
80	C	-0.003687
81	C	-0.001104
82	C	0.002909
83	C	0.038843
84	C	0.002849
86	C	-0.001085
88	C	-0.004780
89	C	-0.014251
92	C	0.000274
93	N	0.135679
94	C	0.006278
95	N	0.133471
96	C	-0.000175
98	C	-0.002311
100	C	-0.004365
101	C	-0.005723
104	C	0.021276
105	C	0.035364
106	C	-0.018925
107	C	-0.003141
108	C	-0.015512
109	C	-0.003306
110	C	0.006184
111	S	0.116287
112	C	0.002169
113	C	0.001386
114	C	0.005098
115	N	0.114792
116	C	0.002668
117	C	0.003430
118	C	0.000616
120	Cu	0.453222
121	C	-0.014360
123	C	-0.000644
125	C	-0.000567
126	C	-0.003048
128	C	-0.001336
129	C	-0.001709
131	C	-0.001583
134	C	0.011640
135	C	0.001249
139	C	0.002918
142	C	-0.002544
145	C	0.001394
146	C	0.001556
147	C	-0.000557
149	C	-0.000458
151	C	0.001197

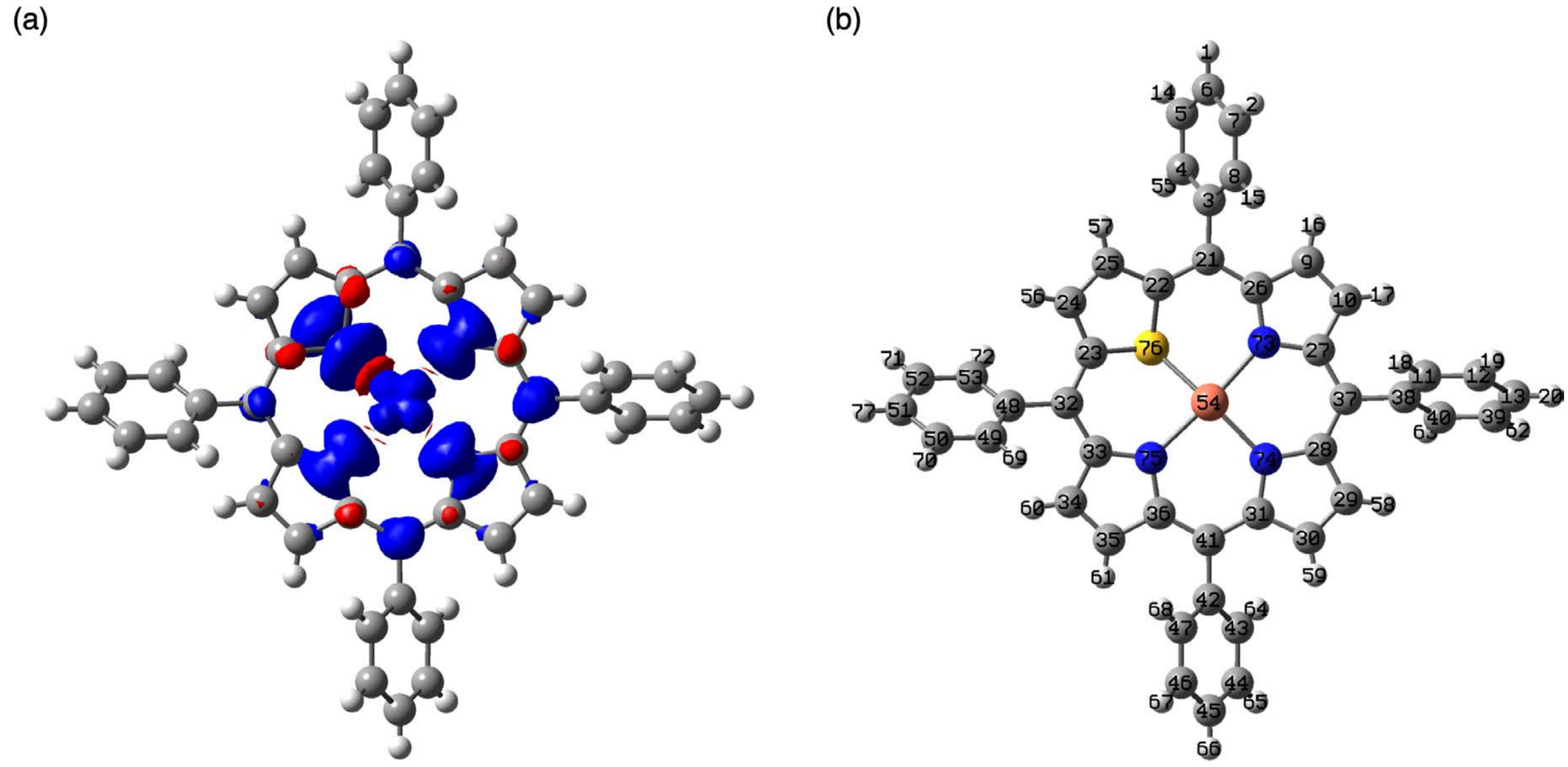

Fig. S70 Spin densities of **1cu**$^{+}$ with S1 thiophene from the crystal structure of **1cu**$^{+}$-$B(C_6F_5)_4^{-}$ estimated at UB3LYP/Def2TZVP: (a) spin-density mapping (δ = 0.001) and (b) molecular structures with atom labels provided for referencing the spin-density values (Table S21) and for discussions related to the molar magnetic susceptibility (Fig. S98).

Table S21 Spin densities, excluding hydrogen atoms of **1cu**$^{+}$ with S1 thiophene unit from the crystal structure of **1cu**$^{+}$-$B(C_6F_5)_4^{-}$ estimated at UB3LYP/Def2TZVP. Atom labels were referred to Fig. S70.

atom labels		spin densities
3	C	-0.002642
4	C	0.001813
5	C	-0.000478
6	C	0.001133
7	C	-0.000526
8	C	0.001339
9	C	-0.002273
10	C	0.005999
11	C	0.001847
12	C	-0.000550
13	C	0.001240
21	C	0.021358
22	C	-0.015354
23	C	-0.013065
24	C	0.003824
25	C	0.004717
26	C	-0.003723
27	C	-0.015734
28	C	-0.013363
29	C	0.001291
30	C	0.001035
31	C	-0.011655
32	C	0.019634
33	C	-0.002448
34	C	-0.002331
35	C	0.005825
36	C	-0.014314
37	C	0.037060
38	C	-0.002810
39	C	-0.000477
40	C	0.001934
41	C	0.036444
42	C	-0.003145
43	C	0.002854
44	C	-0.000974
45	C	0.002628
46	C	-0.001138
47	C	0.002728
48	C	-0.002812
49	C	0.001490
50	C	-0.000553
51	C	0.001230
52	C	-0.000553
53	C	0.001952
54	Cu	0.461758
73	N	0.122341
74	N	0.129614
75	N	0.129559
76	S	0.108270

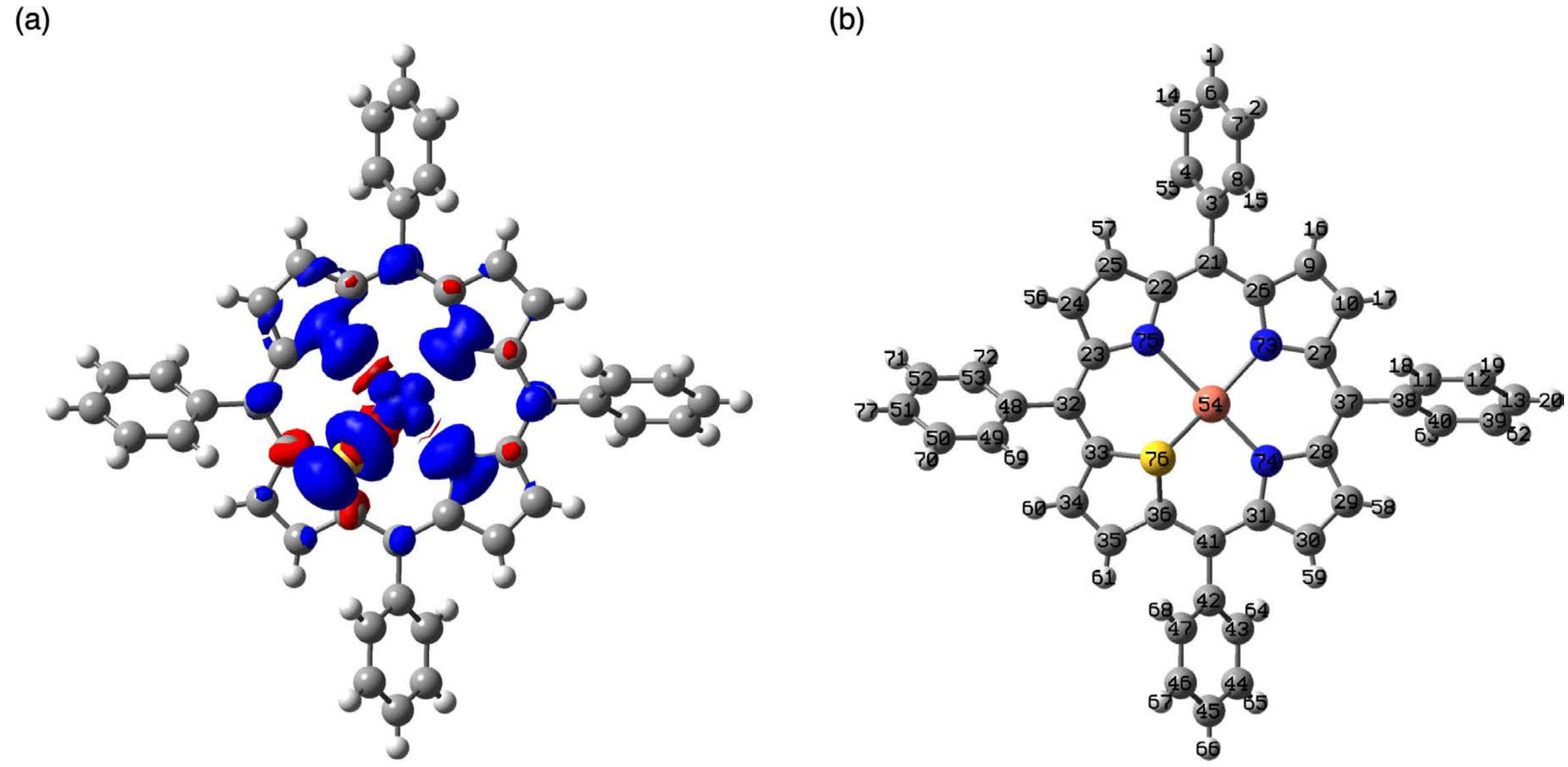

Fig. S71 Spin densities of **1cu**$^{+}$ with S2 thiophene from the crystal structure of **1cu**$^{+}$-$B(C_6F_5)_4^{-}$ estimated at UB3LYP/Def2TZVP: (a) spin-density mapping (δ = 0.001) and (b) molecular structures with atom labels provided for referencing the spin-density values (Table S22) and for discussions related to the molar magnetic susceptibility (Fig. S98).

Table S22 Spin densities, excluding hydrogen atoms of **1cu**$^{+}$ with S2 thiophene unit from the crystal structure of **1cu**$^{+}$-$B(C_6F_5)_4^{-}$ estimated at UB3LYP/Def2TZVP. Atom labels were referred to Fig. S71.

atom labels		spin densities
3	C	-0.002431
4	C	0.001950
5	C	-0.000762
6	C	0.001780
7	C	-0.000705
8	C	0.001996
9	C	0.002532
10	C	0.000765
11	C	0.001224
12	C	-0.000257
13	C	0.000752
21	C	0.026794
22	C	-0.011119
23	C	-0.003331
24	C	0.005328
25	C	0.011022
26	C	-0.010261
27	C	-0.008952
28	C	-0.011658
29	C	0.003514
30	C	-0.000427
31	C	-0.003661
32	C	0.014800
33	C	-0.018178
34	C	0.002636
35	C	0.003652
36	C	-0.015964
37	C	0.025721
38	C	-0.002139
39	C	-0.000358
40	C	0.001365
41	C	0.012323
42	C	-0.002170
43	C	0.001351
44	C	-0.000480
45	C	0.001032
46	C	-0.000318
47	C	0.001329
48	C	-0.002204
49	C	0.002003
50	C	-0.000577
51	C	0.001694
52	C	-0.000765
53	C	0.001661
54	Cu	0.435968
73	N	0.103641
74	N	0.130370
75	N	0.171966
76	S	0.127549

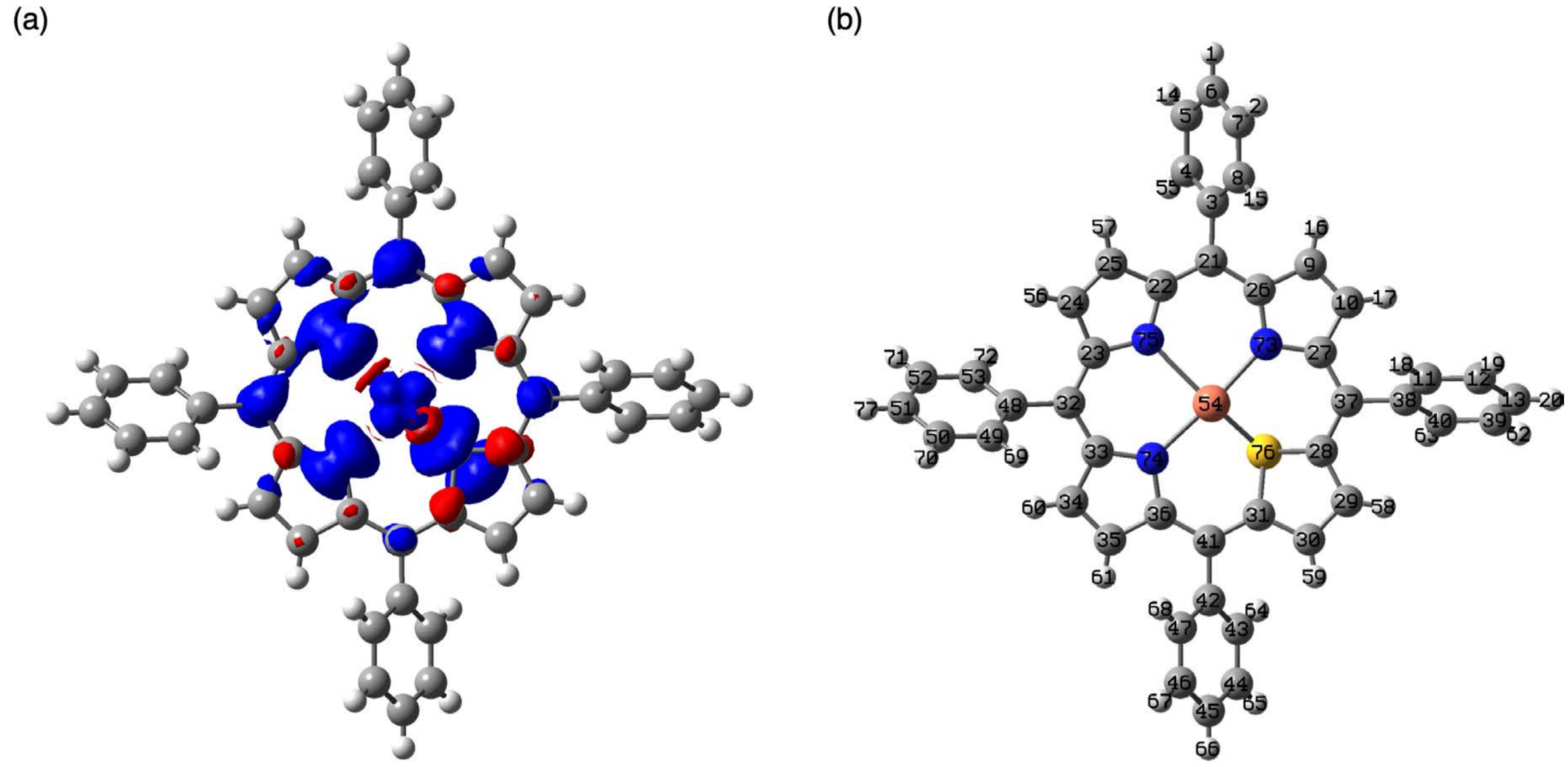

Fig. S72 Spin densities of **1cu**$^{+}$ with S3 thiophene from the crystal structure of **1cu**$^{+}$-$B(C_6F_5)_4^{-}$ estimated at UB3LYP/Def2TZVP: (a) spin-density mapping (δ = 0.001) and (b) molecular structures with atom labels provided for referencing the spin-density values (Table S23) and for discussions related to the molar magnetic susceptibility (Fig. S98).

Table S23 Spin densities, excluding hydrogen atoms of **1cu**$^{+}$ with S3 thiophene unit from the crystal structure of **1cu**$^{+}$-$B(C_6F_5)_4^{-}$ estimated at UB3LYP/Def2TZVP. Atom labels were referred to Fig. S72.

atom labels		spin densities
3	C	-0.003636
4	C	0.003107
5	C	-0.001174
6	C	0.002963
7	C	-0.00124
8	C	0.003282
9	C	0.006148
10	C	-0.002447
11	C	0.001354
12	C	-0.000385
13	C	0.000866
21	C	0.042309
22	C	-0.012546
23	C	-0.008878
24	C	0.005456
25	C	0.010384
26	C	-0.016969
27	C	-0.006707
28	C	-0.024051
29	C	0.006623
30	C	-0.000767
31	C	-0.018621
32	C	0.037737
33	C	-0.014972
34	C	0.007098
35	C	-0.004007
36	C	-0.002691
37	C	0.025855
38	C	-0.003275
39	C	-0.000293
40	C	0.001749
41	C	0.015666
42	C	-0.002797
43	C	0.001675
44	C	-0.000441
45	C	0.001226
46	C	-0.000559
47	C	0.001414
48	C	-0.003574
49	C	0.00386
50	C	-0.001511
51	C	0.003823
52	C	-0.001408
53	C	0.003323
54	Cu	0.390774
73	N	0.141052
74	N	0.12738
75	N	0.159538
76	S	0.128286

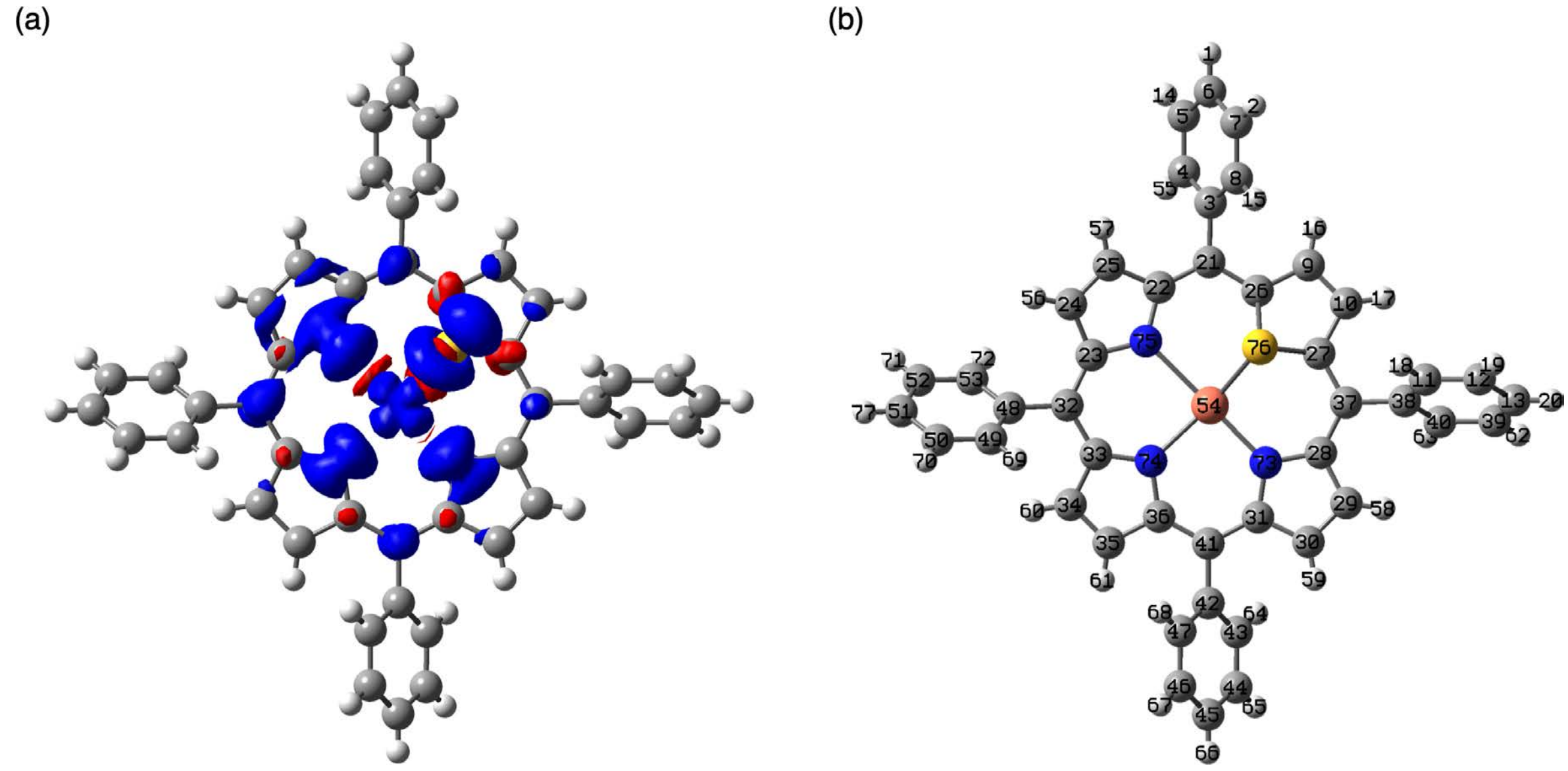

Fig. S73 Spin densities of **1cu**$^+$ with S4 thiophene from the crystal structure of **1cu**$^+$-$B(C_6F_5)_4^-$ estimated at UB3LYP/Def2TZVP: (a) spin-density mapping (δ = 0.001) and (b) molecular structures with atom labels provided for referencing the spin-density values (Table S24) and for discussions related to the molar magnetic susceptibility (Fig. S98).

Table S24 Spin densities, excluding hydrogen atoms of **1cu**$^+$ with S4 thiophene unit from the crystal structure of **1cu**$^+$-$B(C_6F_5)_4^-$ estimated at UB3LYP/Def2TZVP. Atom labels were referred to Fig. S73.

atom labels		spin densities
3	C	-0.002743
4	C	0.001599
5	C	-0.000606
6	C	0.001311
7	C	-0.000445
8	C	0.001780
9	C	0.004767
10	C	0.004142
11	C	0.000844
12	C	-0.000083
13	C	0.000485
21	C	0.017350
22	C	-0.003456
23	C	-0.011036
24	C	0.010288
25	C	0.007004
26	C	-0.017961
27	C	-0.018273
28	C	-0.005088
29	C	-0.001685
30	C	0.004336
31	C	-0.012130
32	C	0.031761
33	C	-0.011170
34	C	0.002698
35	C	-0.000203
36	C	-0.009359
37	C	0.013314
38	C	-0.001770
39	C	-0.000244
40	C	0.000874
41	C	0.026945
42	C	-0.002378
43	C	0.002140
44	C	-0.000796
45	C	0.001888
46	C	-0.000698
47	C	0.001949
48	C	-0.003135
49	C	0.002966
50	C	-0.001059
51	C	0.002873
52	C	-0.001189
53	C	0.002701
54	Cu	0.425506
73	N	0.126235
74	N	0.107070
75	N	0.184992
76	S	0.117686

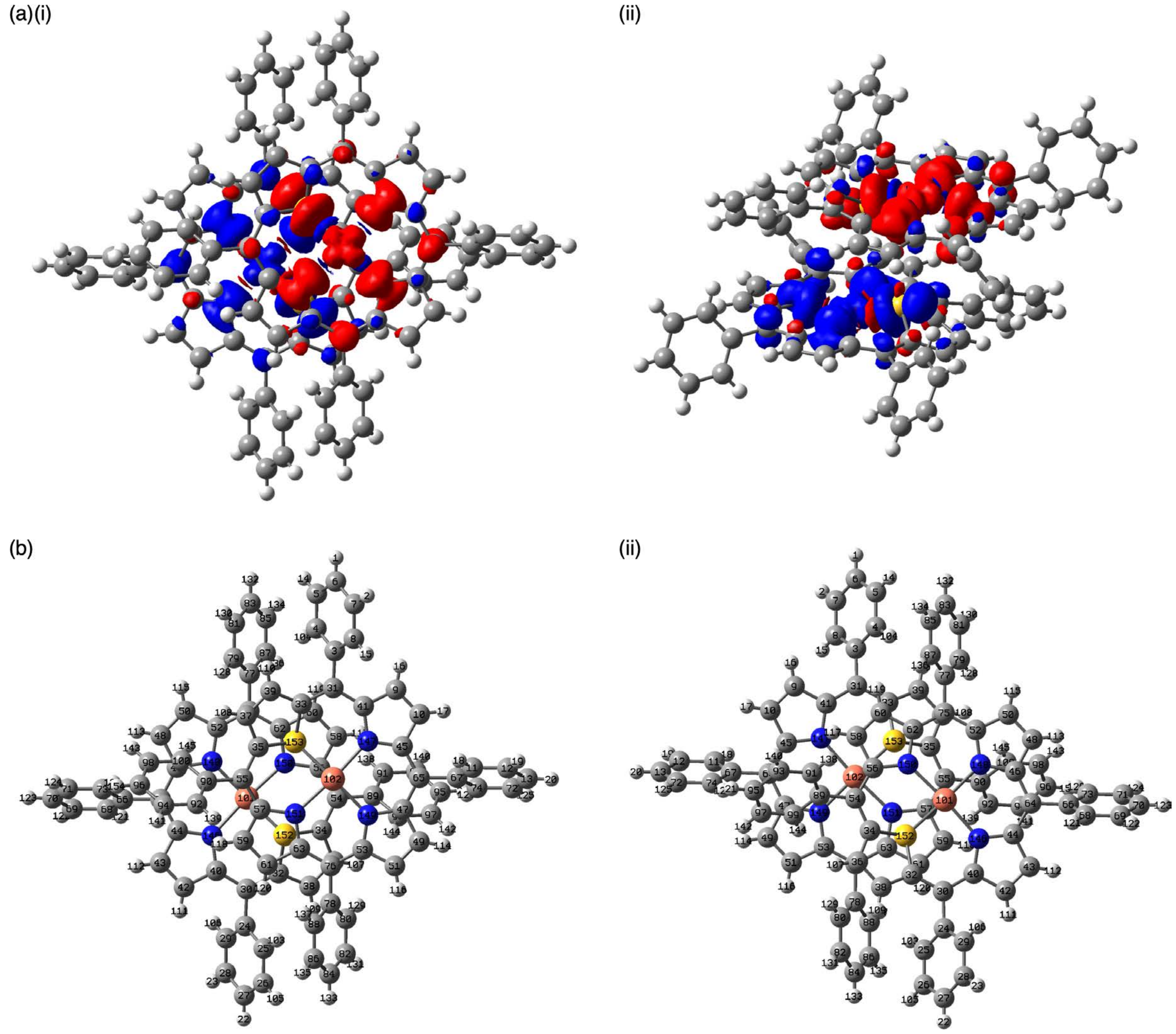

Fig. S74 Spin densities of dimeric **1cu**$^{+}$ from the crystal structure of **1cu**$^{+}$-$B(C_6F_5)_4^-$ as S1-S1 combination estimated at GD3BJ-UB3LYP/Def2TZVP: (a) spin-density mapping (δ = 0.001) as (i) top and (ii) side views and (b) molecular structures with atom labels shown from (i) the same orientation as in (a)(i) and (ii) the opposite orientation, provided for referencing the spin-density values (Table S25) and for discussions related to the molar magnetic susceptibility (Fig. S98).

Table S25 Spin-density values, excluding hydrogen atoms of dimeric **1cu**$^{+}$ from the crystal structure of **1cu**$^{+}$-$B(C_6F_5)_4^{-}$ as S1-S1 combination estimated at GD3BJ-UB3LYP/Def2TZVP. Atom labels were referred to Fig. S74.

atom labels		spin densities
3	C	0.002695
4	C	-0.001753
5	C	0.000432
6	C	-0.000960
7	C	0.000466
8	C	-0.001234
9	C	0.002665
10	C	-0.006593
11	C	-0.001868
12	C	0.000582
13	C	-0.001302
24	C	-0.002691
25	C	0.001749
26	C	-0.000431
27	C	0.000956
28	C	-0.000464
29	C	0.001229
30	C	0.017549
31	C	-0.017629
32	C	-0.014795
33	C	0.014846
34	C	-0.012145
35	C	0.012144
36	C	0.004028
37	C	-0.003986
38	C	0.004486
39	C	-0.004510
40	C	-0.000643
41	C	0.000698
42	C	-0.002687
43	C	0.006647
44	C	-0.014290
45	C	0.014276
46	C	-0.010979
47	C	0.010975
48	C	0.000634
49	C	-0.000624
50	C	0.001452
51	C	-0.001429
52	C	-0.010879
53	C	0.010820
54	C	0.016694
55	C	-0.016841
56	C	-0.000336
57	C	0.000368
58	C	-0.002310
59	C	0.002252
60	C	0.007776
61	C	-0.007733
62	C	-0.006583
63	C	0.006573
64	C	0.034370
65	C	-0.034277
66	C	-0.002730
67	C	0.002722
68	C	0.001875
69	C	-0.000587
70	C	0.001314
71	C	-0.000408
72	C	0.000407
73	C	0.001856
74	C	-0.001848
75	C	0.034670
76	C	-0.034579
77	C	-0.003749
78	C	0.003749
79	C	0.002643
80	C	-0.002634
81	C	-0.000893
82	C	0.000891
83	C	0.002631
84	C	-0.002628
85	C	-0.001155
86	C	0.001153
87	C	0.003092
88	C	-0.003090
89	C	-0.002691
90	C	0.002709
91	C	0.001337
92	C	-0.001357
93	C	-0.000466
94	C	0.000474
95	C	0.000993
96	C	-0.001017
97	C	-0.000373
98	C	0.000382
99	C	0.001887
100	C	-0.001906
101	Cu	0.458481
102	Cu	-0.458411
146	N	0.120196
147	N	-0.120353
148	N	0.126610
149	N	-0.126482
150	N	0.121754
151	N	-0.121755
152	S	0.116028
153	S	-0.116130

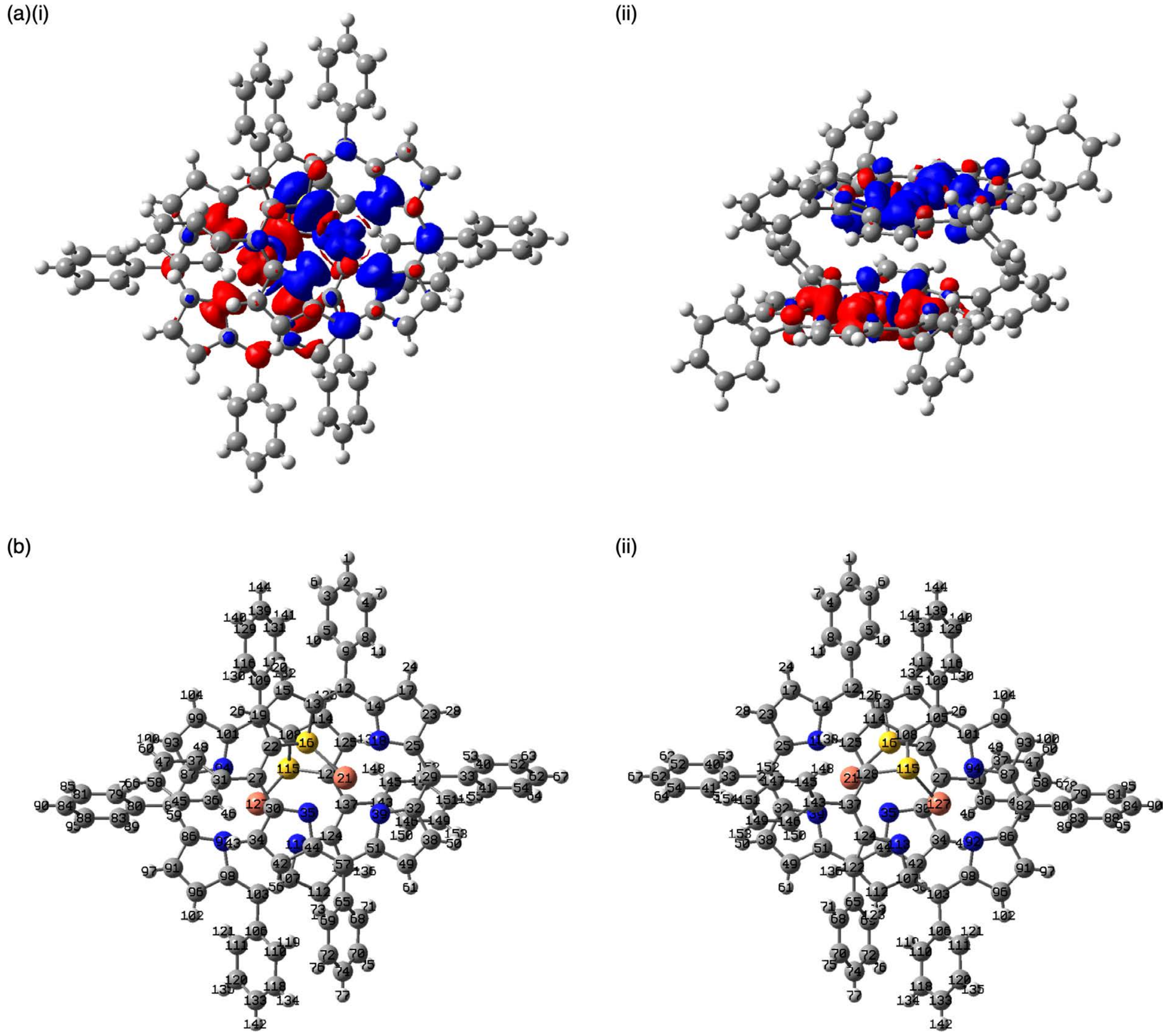

Fig. S75 Spin densities of dimeric **1cu**$^+$ from the crystal structure of **1cu**$^+$-$B(C_6F_5)_4^-$ as S1-S2 combination estimated at GD3BJ-UB3LYP/Def2TZVP: (a) spin-density mapping (δ = 0.001) as (i) top and (ii) side views and (b) molecular structures with atom labels shown from (i) the same orientation as in (a)(i) and (ii) the opposite orientation, provided for referencing the spin-density values (Table S26) and for discussions related to the molar magnetic susceptibility (Fig. S98).

Table S26 Spin-density values, excluding hydrogen atoms of dimeric **1cu**$^+$ from the crystal structure of **1cu**$^+$-$B(C_6F_5)_4^-$ as S1-S2 combination estimated at GD3BJ-UB3LYP/Def2TZVP. Atom labels were referred to Fig. S75.

atom labels		spin densities
2	C	0.001203
3	C	-0.000511
4	C	-0.000562
5	C	0.001967
8	C	0.001469
9	C	-0.002815
12	C	0.020746
13	C	-0.015927
14	C	-0.001890
15	C	0.004242
16	S	0.114831
17	C	-0.002342
18	N	0.120078
19	C	0.002894
21	Cu	0.457921
22	C	-0.012767
23	C	0.006161
25	C	-0.014700
27	C	0.018618
29	C	0.036958
30	C	-0.001564
31	C	-0.002788
32	C	-0.012227
33	C	-0.002860
34	C	-0.001857
35	N	0.125111
36	C	0.001506
37	C	0.001990
38	C	0.001084
39	N	0.127974
40	C	0.001945
41	C	0.001985
42	C	0.006255
44	C	-0.011113
45	C	-0.000520
47	C	-0.000425
49	C	0.000852
51	C	-0.009626
52	C	-0.000585
54	C	-0.000467
57	C	0.035876
58	C	0.001134
62	C	0.001359
65	C	-0.003524
68	C	0.002984
69	C	0.002891
70	C	-0.000987
72	C	-0.001137
74	C	0.002599
79	C	-0.001510
80	C	0.002134
81	C	0.000414
82	C	-0.027457
83	C	-0.001327
84	C	-0.000930
86	C	0.009846
87	C	0.011272
88	C	0.000309
91	C	-0.000445
92	N	-0.102667
93	C	-0.003299
94	N	-0.128721
96	C	-0.002436
98	C	0.008545
99	C	0.000786
101	C	0.003789
103	C	-0.027565
105	C	-0.012803
106	C	0.002578
107	C	0.008569
108	C	0.012885
109	C	0.002110
110	C	-0.002043
111	C	-0.002130
112	C	-0.009664
113	N	-0.169118
114	C	-0.003723
115	S	-0.125721
116	C	-0.001154
117	C	-0.001714
118	C	0.000835
120	C	0.000790
122	C	-0.006216
124	C	0.001258
125	C	-0.002944
127	Cu	-0.434212
128	C	0.020756
129	C	0.000508
131	C	0.000428
133	C	-0.002105
137	C	-0.015832
139	C	-0.001294
143	C	0.002289
145	C	-0.002184
146	C	-0.001832
147	C	0.000642
149	C	0.000781
151	C	-0.001919

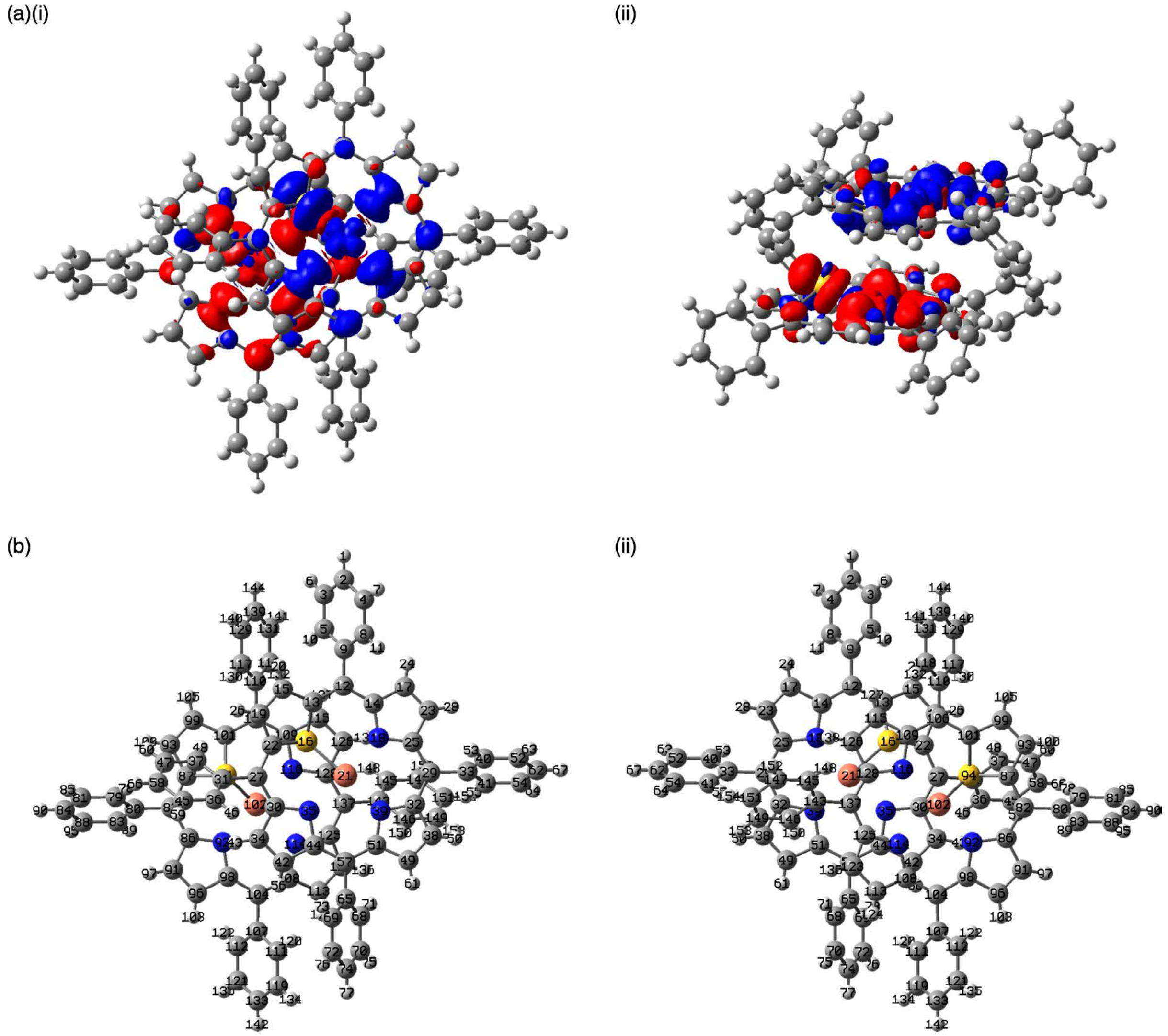

Fig. S76 Spin densities of dimeric **1cu**$^{+}$ from the crystal structure of **1cu**$^{+}$-$B(C_6F_5)_4^{-}$ as S1-S3 combination estimated at GD3BJ-UB3LYP/Def2TZVP: (a) spin-density mapping (δ = 0.001) as (i) top and (ii) side views and (b) molecular structures with atom labels shown from (i) the same orientation as in (a)(i) and (ii) the opposite orientation, provided for referencing the spin-density values (Table S27) and for discussions related to the molar magnetic susceptibility (Fig. S98).

Table S27 Spin-density values, excluding hydrogen atoms of dimeric **1cu**$^{+}$ from the crystal structure of **1cu**$^{+}$-$B(C_6F_5)_4^{-}$ as S1-S3 combination estimated at GD3BJ-UB3LYP/Def2TZVP. Atom labels were referred to Fig. S76.

atom labels		spin densities
2	C	0.001100
3	C	-0.000482
4	C	-0.000515
5	C	0.001877
8	C	0.001370
9	C	-0.002748
12	C	0.019909
13	C	-0.015742
14	C	-0.001663
15	C	0.005184
16	S	0.114324
17	C	-0.002445
18	N	0.120282
19	C	0.002728
21	Cu	0.458762
22	C	-0.010563
23	C	0.006155
25	C	-0.014578
27	C	0.020528
29	C	0.035857
30	C	-0.002981
31	C	-0.003503
32	C	-0.011829
33	C	-0.002816
34	C	-0.002532
35	N	0.125250
36	C	0.001856
37	C	0.000740
38	C	0.000681
39	N	0.127945
40	C	0.001915
41	C	0.001941
42	C	0.006497
44	C	-0.011307
45	C	-0.001143
47	C	-0.000888
49	C	0.001264
51	C	-0.009788
52	C	-0.000581
54	C	-0.000447
57	C	0.035909
58	C	0.001316
62	C	0.001335
65	C	-0.003500
68	C	0.003028
69	C	0.002905
70	C	-0.001005
72	C	-0.001129
74	C	0.002637
79	C	-0.001901
80	C	0.003183
81	C	0.000271
82	C	-0.024003
83	C	-0.001284
84	C	-0.000867
86	C	0.005628
87	C	0.025337
88	C	0.000381
91	C	0.003218
92	N	-0.138809
93	C	-0.007147
94	S	-0.131056
96	C	-0.006730
98	C	0.013654
99	C	0.003376
101	C	0.015534
102	Cu	-0.389700
104	C	-0.040200
106	C	-0.011523
107	C	0.003529
108	C	0.009147
109	C	-0.004707
110	C	0.003316
111	C	-0.003001
112	C	-0.003378
113	C	-0.008143
114	N	-0.156600
115	C	0.002644
116	N	-0.120601
117	C	-0.001574
118	C	-0.001610
119	C	0.001157
121	C	0.001344
123	C	-0.006751
125	C	0.008011
126	C	-0.007494
128	C	0.013408
129	C	0.000345
131	C	0.000593
133	C	-0.003292
137	C	-0.038860
139	C	-0.001285
143	C	0.003877
145	C	-0.003981
146	C	-0.003458
147	C	0.001513
149	C	0.001340
151	C	-0.003961

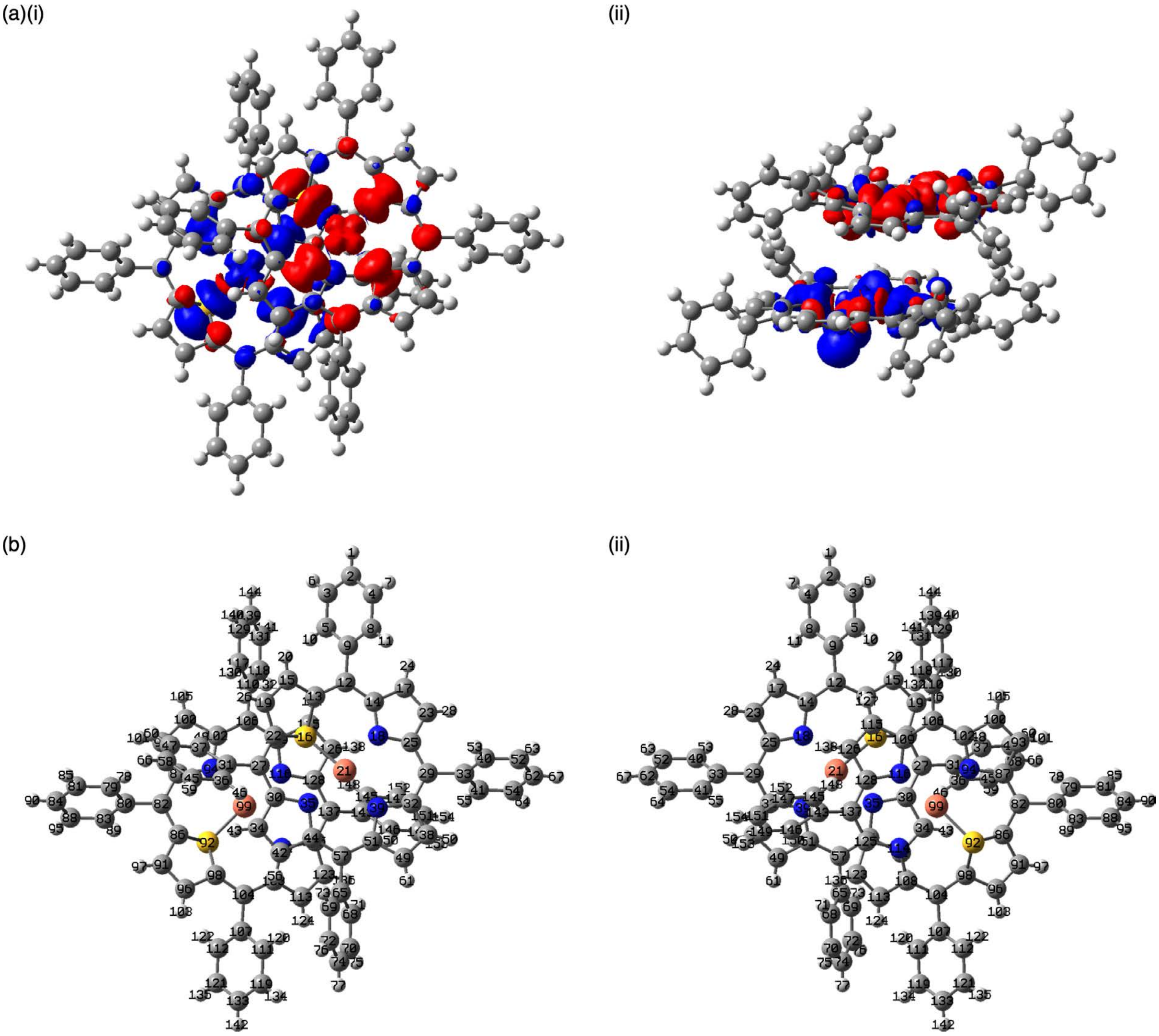

Fig. S77 Spin densities of dimeric **1cu**$^{+}$ from the crystal structure of **1cu**$^{+}$-$B(C_6F_5)_4^{-}$ as S1-S4 combination estimated at GD3BJ-UB3LYP/Def2TZVP: (a) spin-density mapping (δ = 0.001) as (i) top and (ii) side views and (b) molecular structures with atom labels shown from (i) the same orientation as in (a)(i) and (ii) the opposite orientation, provided for referencing the spin-density values (Table S28) and for discussions related to the molar magnetic susceptibility (Fig. S98).

Table S28 Spin-density values, excluding hydrogen atoms of dimeric **1cu**$^{+}$ from the crystal structure of **1cu**$^{+}$-$B(C_6F_5)_4^{-}$ as S1-S4 combination estimated at GD3BJ-UB3LYP/Def2TZVP. Atom labels were referred to Fig. S77.

atom labels		spin densities
2	C	-0.001072
3	C	0.000474
4	C	0.000504
5	C	-0.001865
8	C	-0.001341
9	C	0.002766
12	C	-0.019364
13	C	0.015451
14	C	0.001390
15	C	-0.004878
16	S	-0.115122
17	C	0.002638
18	N	-0.120351
19	C	-0.003275
21	Cu	-0.458256
22	C	0.011911
23	C	-0.006414
25	C	0.014783
27	C	-0.017349
29	C	-0.036365
30	C	0.000416
31	C	0.002673
32	C	0.011937
33	C	0.002854
34	C	0.003160
35	N	-0.124122
36	C	-0.001468
37	C	-0.001879
38	C	-0.000854
39	N	-0.127525
40	C	-0.001933
41	C	-0.001955
42	C	-0.007382
44	C	0.011494
45	C	0.000506
47	C	0.000412
49	C	-0.001097
51	C	0.009759
52	C	0.000589
54	C	0.000449
57	C	-0.036490
58	C	-0.001069
62	C	-0.001340
65	C	0.003630
68	C	-0.002977
69	C	-0.002893
70	C	0.000974
72	C	0.001123
74	C	-0.002577
79	C	0.000730
80	C	-0.001653
81	C	-0.000239
82	C	0.011055
83	C	0.000826
84	C	0.000485
86	C	-0.016185
87	C	-0.002484
88	C	-0.000085
91	C	0.002737
92	S	0.117988
93	C	-0.001876
94	N	0.122875
96	C	0.006506
98	C	-0.017861
99	Cu	0.427242
100	C	0.004644
102	C	-0.009499
104	C	0.015292
106	C	0.023296
107	C	-0.002685
108	C	0.000749
109	C	-0.002690
110	C	-0.002579
111	C	0.001455
112	C	0.001764
113	C	0.005999
114	N	0.180575
115	C	0.001289
116	N	0.100785
117	C	0.001836
118	C	0.002090
119	C	-0.000635
121	C	-0.000464
123	C	0.010941
125	C	-0.009577
126	C	0.002951
128	C	-0.010233
129	C	-0.000716
131	C	-0.000703
133	C	0.001396
137	C	0.031087
139	C	0.001871
143	C	-0.003205
145	C	0.002851
146	C	0.002649
147	C	-0.001002
149	C	-0.001075
151	C	0.002804

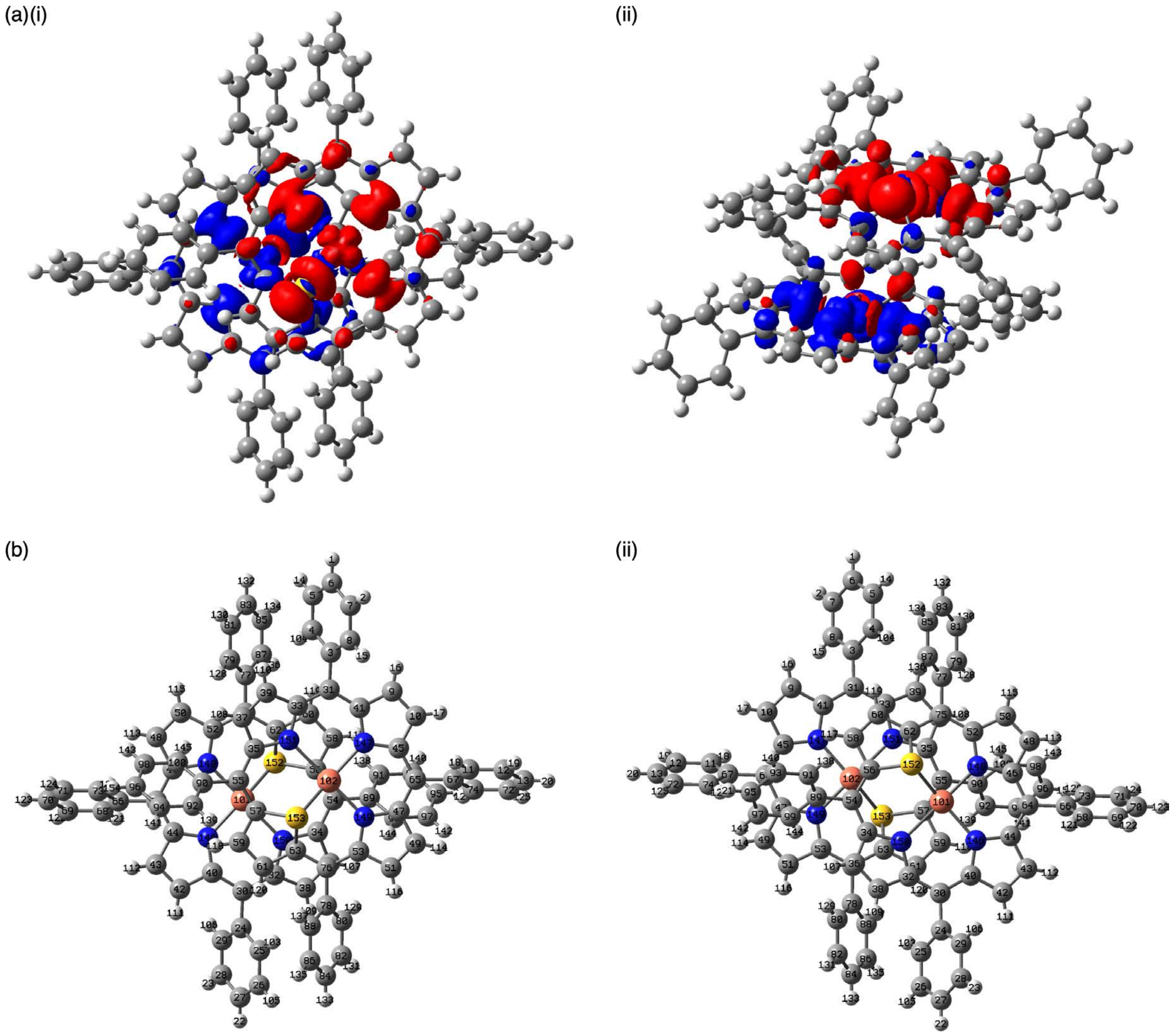

Fig. S78 Spin densities of dimeric **1cu**$^{+}$ from the crystal structure of **1cu**$^{+}$-$B(C_6F_5)_4^{-}$ as S2-S2 combination estimated at GD3BJ-UB3LYP/Def2TZVP: (a) spin-density mapping (δ = 0.001) as (i) top and (ii) side views and (b) molecular structures with atom labels shown from (i) the same orientation as in (a)(i) and (ii) the opposite orientation, provided for referencing the spin-density values (Table S29) and for discussions related to the molar magnetic susceptibility (Fig. S98).

Table S29 Spin-density values, excluding hydrogen atoms of dimeric **1cu**$^{+}$ from the crystal structure of **1cu**$^{+}$-$B(C_6F_5)_4^{-}$ as S2-S2 combination estimated at GD3BJ-UB3LYP/Def2TZVP. Atom labels were referred to Fig. S78.

atom labels		spin densities
3	C	0.002561
4	C	-0.001893
5	C	0.000779
6	C	-0.001912
7	C	0.000726
8	C	-0.001996
9	C	-0.002463
10	C	-0.000626
11	C	-0.001241
12	C	0.000275
13	C	-0.000838
24	C	-0.002563
25	C	0.001895
26	C	-0.000780
27	C	0.001911
28	C	-0.000725
29	C	0.001994
30	C	0.025678
31	C	-0.025651
32	C	-0.007579
33	C	0.007571
34	C	-0.001156
35	C	0.001228
36	C	0.005367
37	C	-0.005492
38	C	0.010324
39	C	-0.010329
40	C	-0.007799
41	C	0.007793
42	C	0.002443
43	C	0.000637
44	C	-0.009286
45	C	0.009242
46	C	-0.010899
47	C	0.010848
48	C	0.003312
49	C	-0.003306
50	C	-0.000616
51	C	0.000619
52	C	-0.002486
53	C	0.002423
54	C	0.013200
55	C	-0.013331
56	C	-0.016200
57	C	0.016363
58	C	0.001108
59	C	-0.001188
60	C	0.005484
61	C	-0.005365
62	C	-0.016282
63	C	0.016179
64	C	0.026250
65	C	-0.026123
66	C	-0.002104
67	C	0.002092
68	C	0.001250
69	C	-0.000280
70	C	0.000849
71	C	-0.000390
72	C	0.000387
73	C	0.001436
74	C	-0.001426
75	C	0.012940
76	C	-0.012805
77	C	-0.001969
78	C	0.001961
79	C	0.001402
80	C	-0.001391
81	C	-0.000564
82	C	0.000562
83	C	0.001112
84	C	-0.001106
85	C	-0.000358
86	C	0.000356
87	C	0.001393
88	C	-0.001386
89	C	-0.001964
90	C	0.001983
91	C	0.001954
92	C	-0.001977
93	C	-0.000569
94	C	0.000578
95	C	0.001665
96	C	-0.001691
97	C	-0.000714
98	C	0.000723
99	C	0.001660
100	C	-0.001676
101	Cu	0.437292
102	Cu	-0.437240
146	N	0.101095
147	N	-0.101129
148	N	0.129240
149	N	-0.129033
150	N	0.165946
151	N	-0.166237
152	S	0.127607
153	S	-0.127559

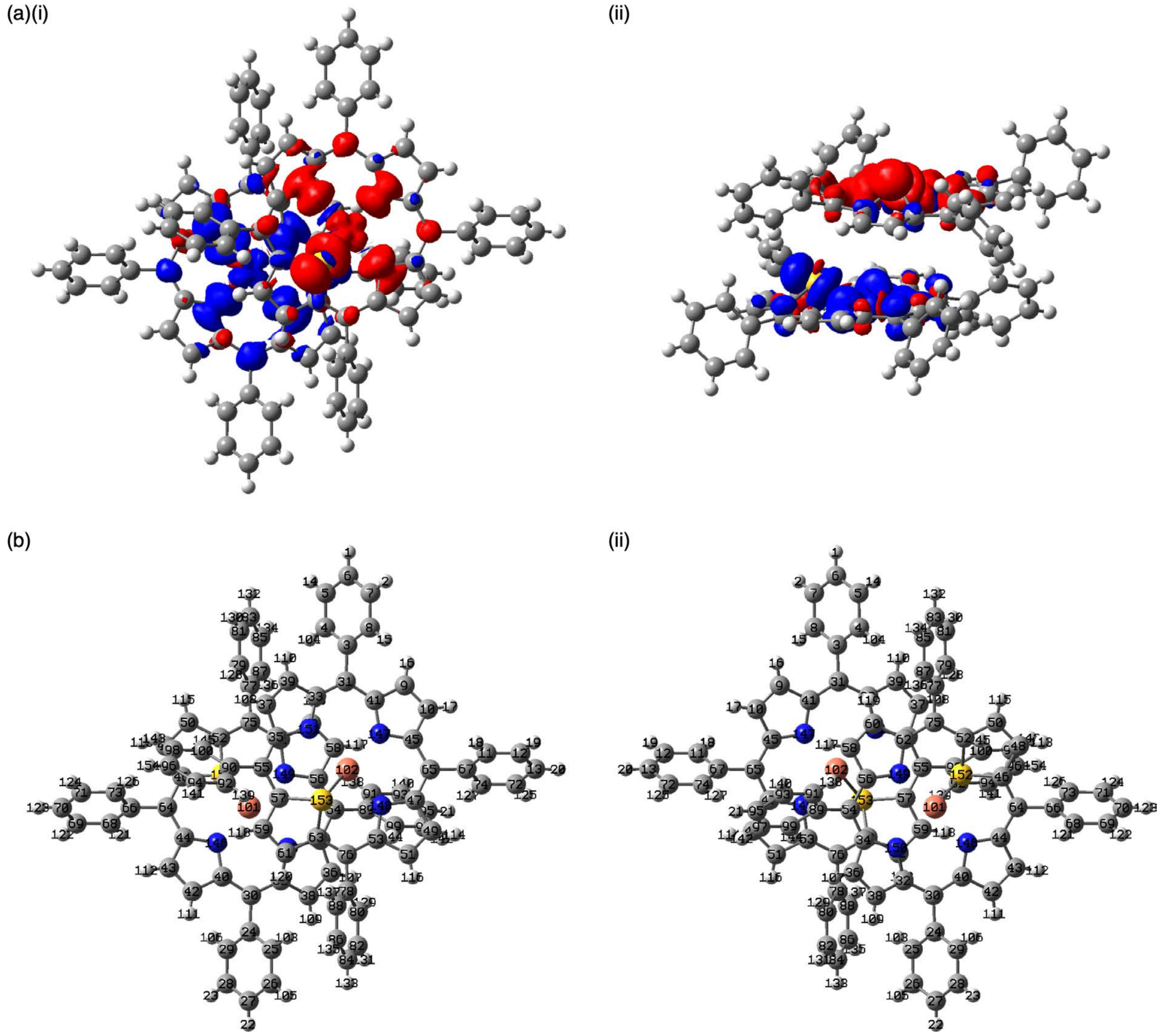

Fig. S79 Spin densities of dimeric **1cu**$^{+}$ from the crystal structure of **1cu**$^{+}$-$B(C_6F_5)_4^{-}$ as S2-S3 combination estimated at GD3BJ-UB3LYP/Def2TZVP: (a) spin-density mapping (δ = 0.001) as (i) top and (ii) side views and (b) molecular structures with atom labels shown from (i) the same orientation as in (a)(i) and (ii) the opposite orientation, provided for referencing the spin-density values (Table S30) and for discussions related to the molar magnetic susceptibility (Fig. S98).

Table S30 Spin-density values, excluding hydrogen atoms of dimeric **1cu**$^{+}$ from the crystal structure of **1cu**$^{+}$-$B(C_6F_5)_4^{-}$ as S2-S3 combination estimated at GD3BJ-UB3LYP/Def2TZVP. Atom labels were referred to Fig. S79.

atom labels		spin densities
3	C	0.002529
4	C	-0.001923
5	C	0.000787
6	C	-0.001960
7	C	0.000749
8	C	-0.002024
9	C	-0.002400
10	C	-0.000621
11	C	-0.001233
12	C	0.000274
13	C	-0.000845
24	C	-0.003624
25	C	0.002965
26	C	-0.001137
27	C	0.003160
28	C	-0.001287
29	C	0.003315
30	C	0.039874
31	C	-0.026336
32	C	-0.008818
33	C	0.007769
34	C	-0.007462
35	C	-0.000956
36	C	0.005320
37	C	-0.006022
38	C	0.009399
39	C	-0.009719
40	C	-0.012856
41	C	0.007958
42	C	0.006700
43	C	-0.002912
44	C	-0.005018
45	C	0.009470
46	C	-0.024417
47	C	0.011042
48	C	0.006132
49	C	-0.003273
50	C	-0.002215
51	C	0.000637
52	C	-0.016553
53	C	0.002186
54	C	0.038182
55	C	-0.014341
56	C	-0.013470
57	C	0.016661
58	C	0.007508
59	C	-0.000753
60	C	-0.003482
61	C	-0.006225
62	C	0.000510
63	C	0.016148
64	C	0.024171
65	C	-0.026122
66	C	-0.003178
67	C	0.002064
68	C	0.001290
69	C	-0.000377
70	C	0.000858
71	C	-0.000257
72	C	0.000395
73	C	0.001886
74	C	-0.001435
75	C	0.014086
76	C	-0.012046
77	C	-0.003267
78	C	0.001956
79	C	0.001941
80	C	-0.001390
81	C	-0.000488
82	C	0.000538
83	C	0.001304
84	C	-0.001078
85	C	-0.000598
86	C	0.000341
87	C	0.001553
88	C	-0.001370
89	C	-0.003809
90	C	0.002508
91	C	0.003884
92	C	-0.002161
93	C	-0.001482
94	C	0.001158
95	C	0.003797
96	C	-0.001750
97	C	-0.001276
98	C	0.001146
99	C	0.003303
100	C	-0.000040
101	Cu	0.390813
102	Cu	-0.438670
146	N	0.137194
147	N	-0.101939
148	N	-0.129311
149	N	0.123763
150	N	0.153339
151	N	-0.165102
152	S	0.132322
153	S	-0.125860

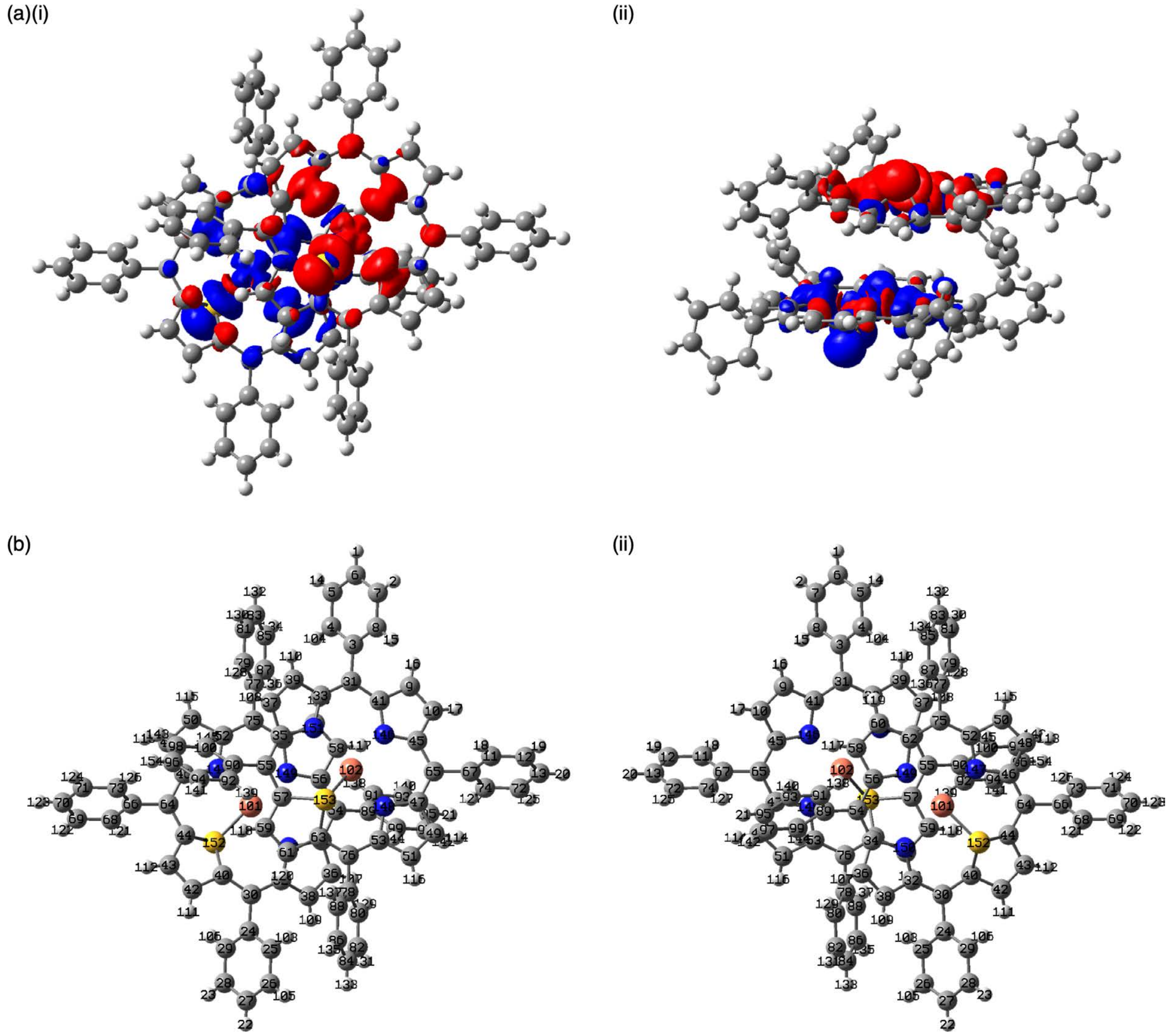

Fig. S80 Spin densities of dimeric **1cu**$^{+}$ from the crystal structure of **1cu**$^{+}$-B(C_6F_5)$_4^{-}$ as S2-S4 combination estimated at GD3BJ-UB3LYP/Def2TZVP: (a) spin-density mapping (δ = 0.001) as (i) top and (ii) side views and (b) molecular structures with atom labels shown from (i) the same orientation as in (a)(i) and (ii) the opposite orientation, provided for referencing the spin-density values (Table S31) and for discussions related to the molar magnetic susceptibility (Fig. S98).

Table S31 Spin-density values, excluding hydrogen atoms of dimeric **1cu**$^{+}$ from the crystal structure of **1cu**$^{+}$-$B(C_6F_5)_4^{-}$ as S2-S4 combination estimated at GD3BJ-UB3LYP/Def2TZVP. Atom labels were referred to Fig. S80.

atom labels		spin densities
3	C	0.002543
4	C	-0.001926
5	C	0.000791
6	C	-0.001946
7	C	0.000740
8	C	-0.002008
9	C	-0.002375
10	C	-0.000633
11	C	-0.001269
12	C	0.000289
13	C	-0.000863
24	C	-0.002756
25	C	0.001495
26	C	-0.000637
27	C	0.001392
28	C	-0.000460
29	C	0.001777
30	C	0.014883
31	C	-0.026306
32	C	0.001650
33	C	0.008155
34	C	-0.009710
35	C	0.000563
36	C	0.009641
37	C	-0.005927
38	C	0.007057
39	C	-0.010086
40	C	-0.016263
41	C	0.008015
42	C	0.005192
43	C	0.003530
44	C	-0.017798
45	C	0.009647
46	C	-0.003959
47	C	0.011042
48	C	-0.001745
49	C	-0.003395
50	C	0.004171
51	C	0.000748
52	C	-0.009737
53	C	0.002246
54	C	0.032043
55	C	-0.013180
56	C	-0.010863
57	C	0.016684
58	C	0.003131
59	C	-0.001434
60	C	0.000106
61	C	-0.004779
62	C	-0.007592
63	C	0.015952
64	C	0.013206
65	C	-0.026613
66	C	-0.001780
67	C	0.002103
68	C	0.000901
69	C	-0.000102
70	C	0.000535
71	C	-0.000263
72	C	0.000396
73	C	0.000818
74	C	-0.001448
75	C	0.026226
76	C	-0.012811
77	C	-0.002532
78	C	0.001977
79	C	0.002253
80	C	-0.001385
81	C	-0.000850
82	C	0.000537
83	C	0.001911
84	C	-0.001110
85	C	-0.000718
86	C	0.000351
87	C	0.002018
88	C	-0.001397
89	C	-0.003273
90	C	0.002053
91	C	0.002932
92	C	-0.002056
93	C	-0.001036
94	C	0.000615
95	C	0.002833
96	C	-0.001732
97	C	-0.001091
98	C	0.000744
99	C	0.002664
100	C	-0.001672
101	Cu	0.426796
102	Cu	-0.437563
146	N	-0.102187
147	N	0.124922
148	N	-0.128766
149	N	0.104433
150	N	0.178103
151	N	-0.167128
152	S	0.118056
153	S	-0.125705

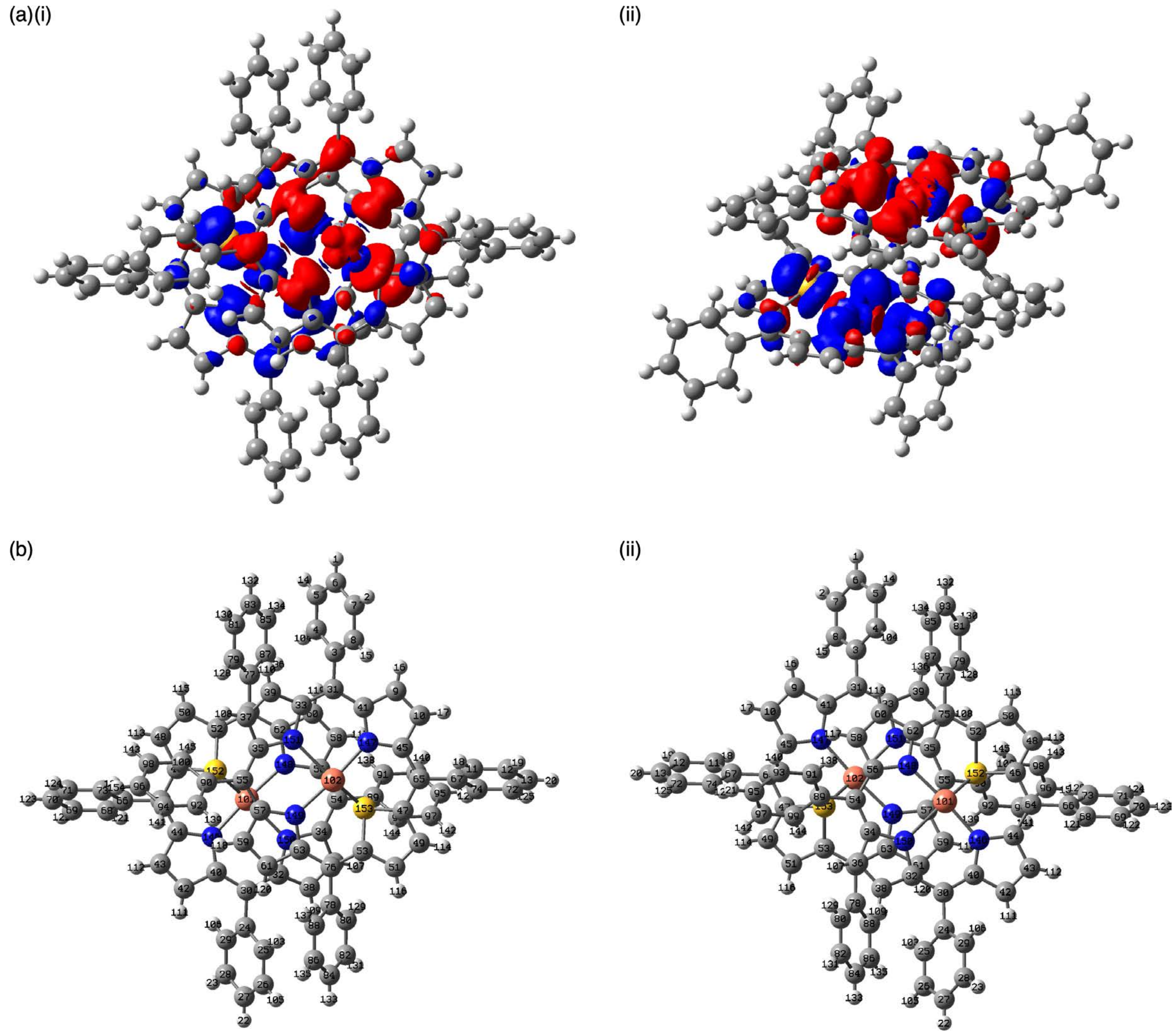

Fig. S81 Spin densities of dimeric **1cu**$^+$ from the crystal structure of **1cu**$^+$-$B(C_6F_5)_4^-$ as S3-S3 combination estimated at GD3BJ-UB3LYP/Def2TZVP: (a) spin-density mapping (δ = 0.001) as (i) top and (ii) side views and (b) molecular structures with atom labels shown from (i) the same orientation as in (a)(i) and (ii) the opposite orientation, provided for referencing the spin-density values (Table S32) and for discussions related to the molar magnetic susceptibility (Fig. S98).

Table S32 Spin-density values, excluding hydrogen atoms of dimeric **1cu**$^+$ from the crystal structure of **1cu**$^+$-$B(C_6F_5)_4^-$ as S3-S3 combination estimated at GD3BJ-UB3LYP/Def2TZVP. Atom labels were referred to Fig. S81.

atom labels		spin densities
3	C	0.003560
4	C	-0.002975
5	C	0.001125
6	C	-0.003226
7	C	0.001335
8	C	-0.003370
9	C	-0.006863
10	C	0.003183
11	C	-0.001240
12	C	0.000363
13	C	-0.000827
24	C	-0.003566
25	C	0.002980
26	C	-0.001127
27	C	0.003226
28	C	-0.001333
29	C	0.003369
30	C	0.040124
31	C	-0.040082
32	C	-0.008377
33	C	0.008348
34	C	-0.006178
35	C	0.006246
36	C	0.005902
37	C	-0.006050
38	C	0.008318
39	C	-0.008370
40	C	-0.013149
41	C	0.013139
42	C	0.006822
43	C	-0.003172
44	C	-0.004809
45	C	0.004689
46	C	-0.024606
47	C	0.024429
48	C	0.007160
49	C	-0.007053
50	C	-0.002179
51	C	0.002041
52	C	-0.015337
53	C	0.015394
54	C	0.039123
55	C	-0.039301
56	C	-0.013427
57	C	0.013530
58	C	0.007105
59	C	-0.007116
60	C	-0.003200
61	C	0.003210
62	C	0.001261
63	C	-0.001219
64	C	0.023328
65	C	-0.023129
66	C	-0.003174
67	C	0.003160
68	C	0.001251
69	C	-0.000367
70	C	0.000839
71	C	-0.000267
72	C	0.000263
73	C	0.001881
74	C	-0.001870
75	C	0.012368
76	C	-0.012350
77	C	-0.003138
78	C	0.003137
79	C	0.001851
80	C	-0.001847
81	C	-0.000436
82	C	0.000435
83	C	0.001193
84	C	-0.001191
85	C	-0.000532
86	C	0.000531
87	C	0.001415
88	C	-0.001414
89	C	-0.004459
90	C	0.004482
91	C	0.003980
92	C	-0.004015
93	C	-0.001995
94	C	0.002009
95	C	0.003709
96	C	-0.003755
97	C	-0.001637
98	C	0.001653
99	C	0.001677
100	C	-0.001699
101	Cu	0.392381
102	Cu	-0.391827
146	N	0.137549
147	N	-0.137636
148	N	0.122575
149	N	-0.122645
150	N	0.152718
151	N	-0.153079
152	S	0.131740
153	S	-0.131495

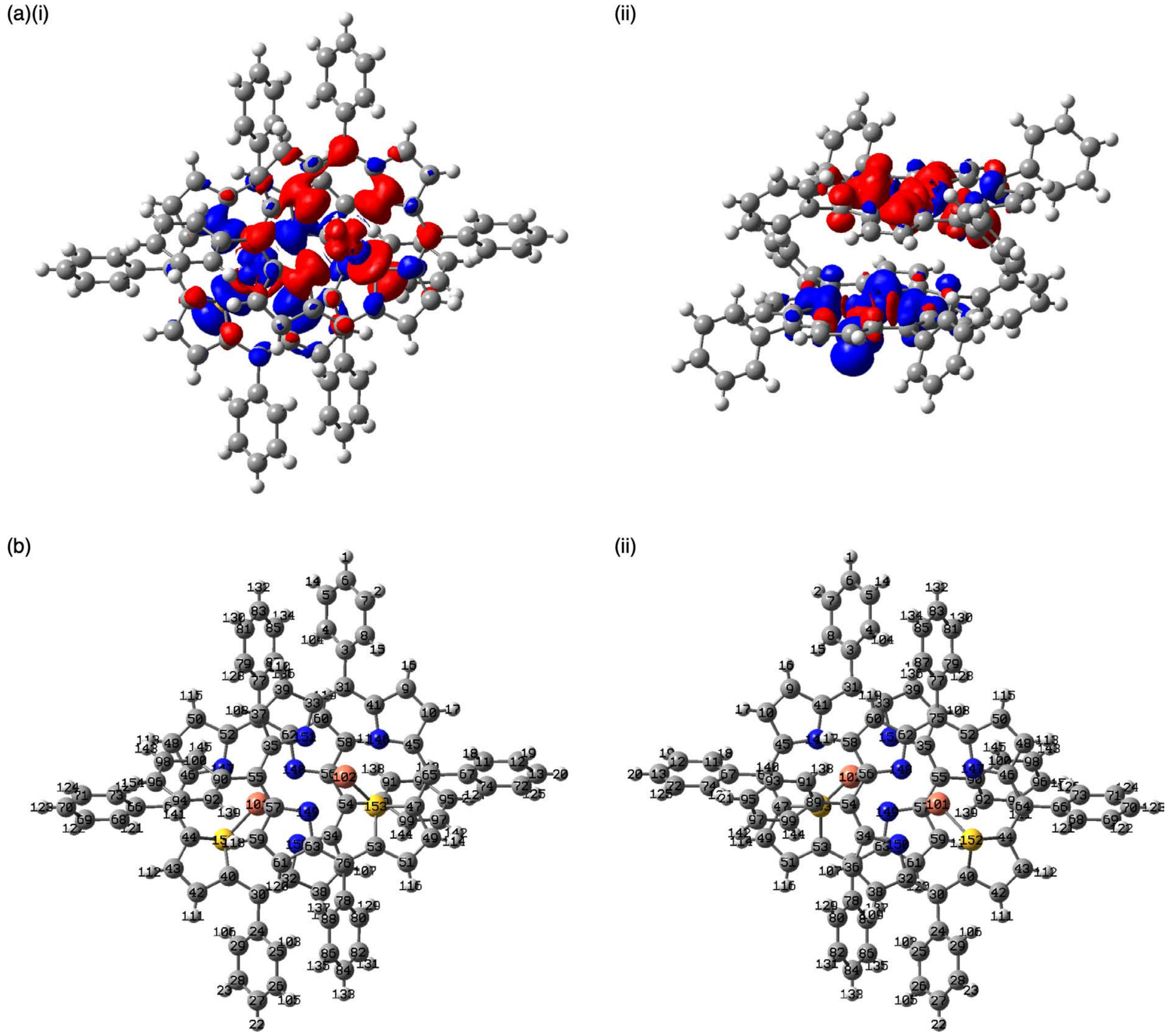

Fig. S82 Spin densities of dimeric **1cu**$^{+}$ from the crystal structure of **1cu**$^{+}$-$B(C_6F_5)_4^{-}$ as S3-S4 combination estimated at GD3BJ-UB3LYP/Def2TZVP: (a) spin-density mapping (δ = 0.001) as (i) top and (ii) side views and (b) molecular structures with atom labels shown from (i) the same orientation as in (a)(i) and (ii) the opposite orientation, provided for referencing the spin-density values (Table S33) and for discussions related to the molar magnetic susceptibility (Fig. S98).

Table S33 Spin-density values, excluding hydrogen atoms of dimeric **1cu**$^{+}$ from the crystal structure of **1cu**$^{+}$-$B(C_6F_5)_4^{-}$ as S3-S4 combination estimated at GD3BJ-UB3LYP/Def2TZVP. Atom labels were referred to Fig. S82.

atom labels		spin densities
3	C	0.003594
4	C	-0.002975
5	C	0.001138
6	C	-0.003182
7	C	0.001307
8	C	-0.003325
9	C	-0.006562
10	C	0.002952
11	C	-0.001277
12	C	0.000373
13	C	-0.000843
24	C	-0.002619
25	C	0.001440
26	C	-0.000622
27	C	0.001366
28	C	-0.000460
29	C	0.001740
30	C	0.014809
31	C	-0.040107
32	C	0.001875
33	C	0.008909
34	C	-0.007378
35	C	0.007248
36	C	0.010123
37	C	-0.006044
38	C	0.006462
39	C	-0.008789
40	C	-0.015693
41	C	0.013184
42	C	0.005385
43	C	0.004356
44	C	-0.017276
45	C	0.005366
46	C	-0.003353
47	C	0.024829
48	C	-0.001848
49	C	-0.006650
50	C	0.004439
51	C	0.002606
52	C	-0.009296
53	C	0.015696
54	C	0.032284
55	C	-0.038058
56	C	-0.011240
57	C	0.012054
58	C	0.002583
59	C	-0.006344
60	C	0.000545
61	C	0.002704
62	C	-0.007019
63	C	-0.000042
64	C	0.011938
65	C	-0.023985
66	C	-0.001672
67	C	0.003184
68	C	0.000841
69	C	-0.000087
70	C	0.000490
71	C	-0.000246
72	C	0.000260
73	C	0.000759
74	C	-0.001877
75	C	0.024782
76	C	-0.013637
77	C	-0.002399
78	C	0.003277
79	C	0.002186
80	C	-0.001883
81	C	-0.000816
82	C	0.000445
83	C	0.001822
84	C	-0.001252
85	C	-0.000650
86	C	0.000560
87	C	0.001891
88	C	-0.001511
89	C	-0.003836
90	C	0.003929
91	C	0.003063
92	C	-0.003897
93	C	-0.001571
94	C	0.001489
95	C	0.002746
96	C	-0.003791
97	C	-0.001454
98	C	0.001332
99	C	0.001013
100	C	-0.003335
101	Cu	0.429824
102	Cu	-0.390671
146	N	-0.138060
147	N	0.124586
148	N	0.103621
149	N	-0.122981
150	N	0.176285
151	N	-0.154683
152	S	0.116979
153	S	-0.131373

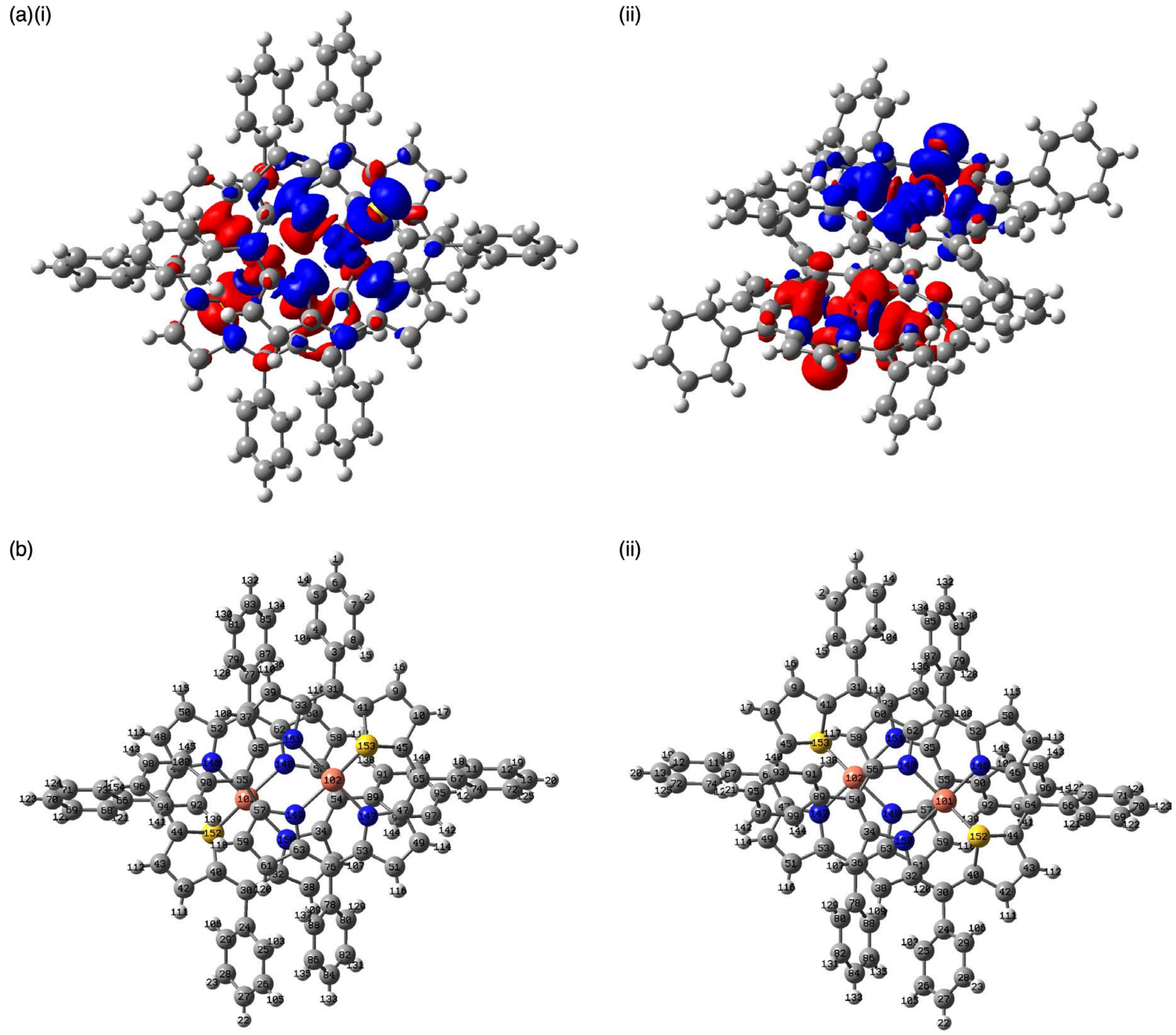

Fig. S83 Spin densities of dimeric **1cu**$^+$ from the crystal structure of **1cu**$^+$-$B(C_6F_5)_4^-$ as S4-S4 combination estimated at GD3BJ-UB3LYP/Def2TZVP: (a) spin-density mapping (δ = 0.001) as (i) top and (ii) side views and (b) molecular structures with atom labels shown from (i) the same orientation as in (a)(i) and (ii) the opposite orientation, provided for referencing the spin-density values (Table S34) and for discussions related to the molar magnetic susceptibility (Fig. S98).

Table S34 Spin-density values, excluding hydrogen atoms of dimeric **1cu**$^{+}$ from the crystal structure of **1cu**$^{+}$-$B(C_6F_5)_4^{-}$ as S4-S4 combination estimated at GD3BJ-UB3LYP/Def2TZVP. Atom labels were referred to Fig. S83.

atom labels		spin densities
3	C	-0.002650
4	C	0.001447
5	C	-0.000621
6	C	0.001362
7	C	-0.000455
8	C	0.001742
9	C	0.005536
10	C	0.003588
11	C	0.000859
12	C	-0.000094
13	C	0.000503
24	C	0.002650
25	C	-0.001442
26	C	0.000618
27	C	-0.001353
28	C	0.000451
29	C	-0.001733
30	C	-0.014949
31	C	0.015013
32	C	-0.001327
33	C	0.001311
34	C	0.008954
35	C	-0.008994
36	C	-0.010256
37	C	0.010344
38	C	-0.006614
39	C	0.006692
40	C	0.016521
41	C	-0.016548
42	C	-0.005571
43	C	-0.003619
44	C	0.017238
45	C	-0.017228
46	C	0.003427
47	C	-0.003412
48	C	0.001785
49	C	-0.001805
50	C	-0.004331
51	C	0.004324
52	C	0.009405
53	C	-0.009383
54	C	-0.030920
55	C	0.031084
56	C	0.009205
57	C	-0.009262
58	C	-0.001844
59	C	0.001886
60	C	-0.001112
61	C	0.001085
62	C	0.007926
63	C	-0.007924
64	C	-0.012408
65	C	0.012279
66	C	0.001712
67	C	-0.001701
68	C	-0.000868
69	C	0.000096
70	C	-0.000510
71	C	0.000253
72	C	-0.000250
73	C	-0.000783
74	C	0.000774
75	C	-0.025896
76	C	0.025802
77	C	0.002564
78	C	-0.002554
79	C	-0.002191
80	C	0.002179
81	C	0.000810
82	C	-0.000808
83	C	-0.001844
84	C	0.001840
85	C	0.000680
86	C	-0.000678
87	C	-0.001965
88	C	0.001962
89	C	0.003275
90	C	-0.003298
91	C	-0.002868
92	C	0.002901
93	C	0.001015
94	C	-0.001029
95	C	-0.002739
96	C	0.002781
97	C	0.001090
98	C	-0.001105
99	C	-0.002576
100	C	0.002603
101	Cu	-0.428413
102	Cu	0.428175
146	N	-0.124187
147	N	0.124003
148	N	-0.103446
149	N	0.103399
150	N	-0.178416
151	N	0.178783
152	S	-0.117359
153	S	0.117409

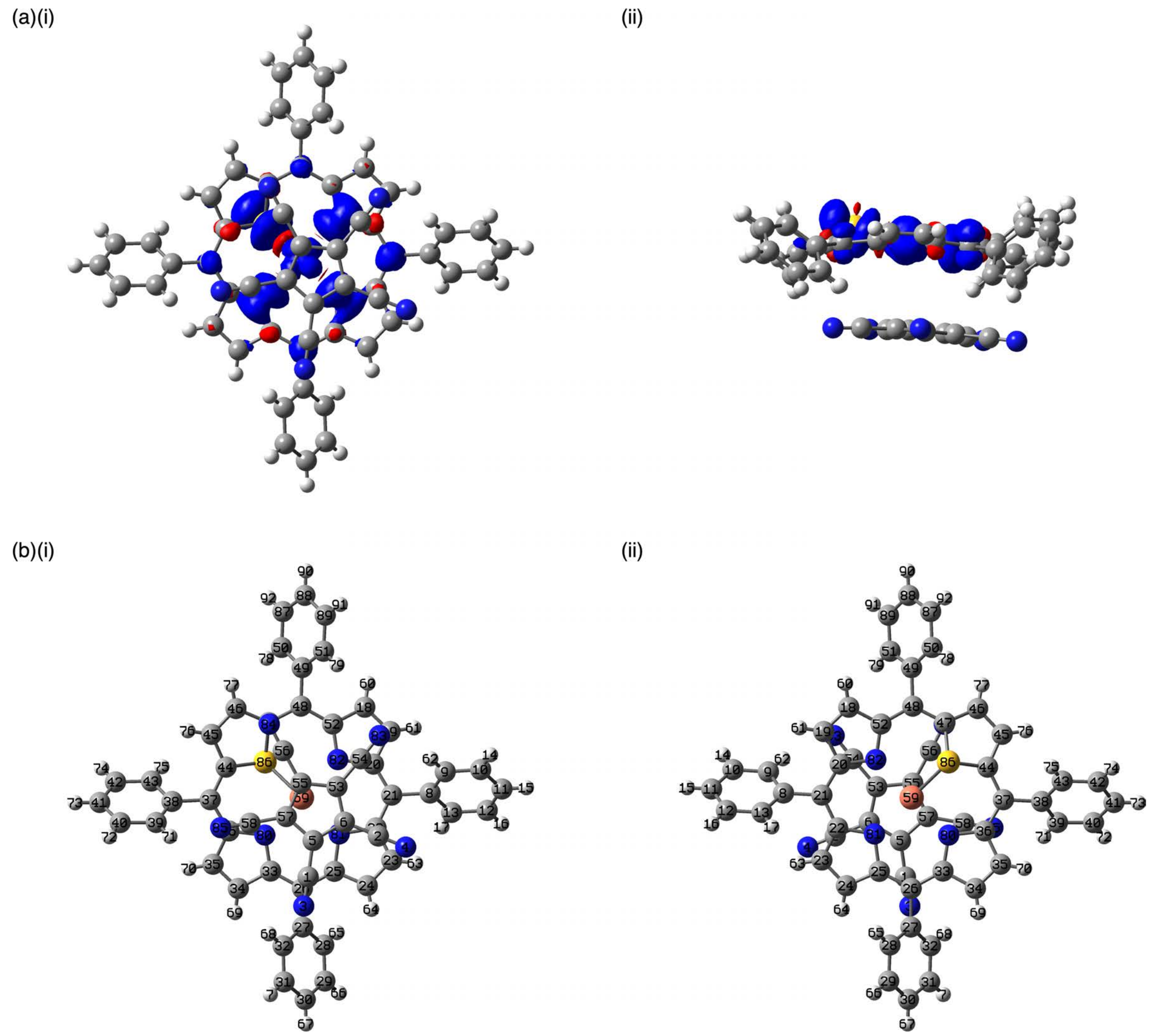

Fig. S84 Spin densities of dimeric **1cu**$^+$ from the crystal structure of **1cu**$^+$-PCCp$^-$ as the major structure estimated at GD3BJ-UB3LYP/Def2TZVP: (a) spin-density mapping (δ = 0.001) as (i) top and (ii) side views and (b) molecular structures with atom labels shown from (i) the same orientation as in (a)(i) and (ii) the opposite orientation, provided for referencing the spin-density values (Table S35) and for discussions related to the molar magnetic susceptibility (Fig. S98).

Table S35 Spin-density values, excluding hydrogen atoms of dimeric **1cu**$^+$ from the crystal structure of **1cu**$^+$-PCCp$^-$ estimated at GD3BJ-UB3LYP/Def2TZVP. Atom labels were referred to Fig. S84.

atom labels		spin densities
1	C	-0.262182
2	C	-0.265485
3	N	0.015777
4	N	0.026944
5	C	0.058126
6	C	0.046045
8	C	0.158394
9	C	-0.060751
10	C	0.038983
11	C	0.001472
12	C	0.044439
13	C	-0.177516
18	C	-0.093374
19	C	-0.008662
20	C	0.226605
21	C	0.094249
22	C	0.131234
23	C	-0.066365
24	C	0.006615
25	C	0.121724
26	C	0.090433
27	C	0.136915
28	C	-0.163277
29	C	0.033677
30	C	0.007229
31	C	0.040644
32	C	-0.100714
33	C	0.262998
34	C	-0.043976
35	C	-0.066255
36	C	0.3009
37	C	0.005355
38	C	0.117968
39	C	-0.077351
40	C	0.054367
41	C	-0.001897
42	C	0.044704
43	C	-0.175683
44	C	0.131304
45	C	0.044192
46	C	-0.021363
47	C	0.113829
48	C	0.071494
49	C	0.141808
50	C	-0.162202
51	C	-0.094528
52	C	0.314451
53	C	0.085329
54	C	-0.194901
55	C	0.039002
56	C	-0.245457
57	C	0.059821
58	C	-0.221645
59	Cu	-0.24765
80	N	-0.121864
81	N	-0.168622
82	N	-0.107989
83	N	-0.043518
84	N	-0.014821
85	N	-0.005133
86	S	0.055337
87	C	0.037878
88	C	0.009178
89	C	0.043760

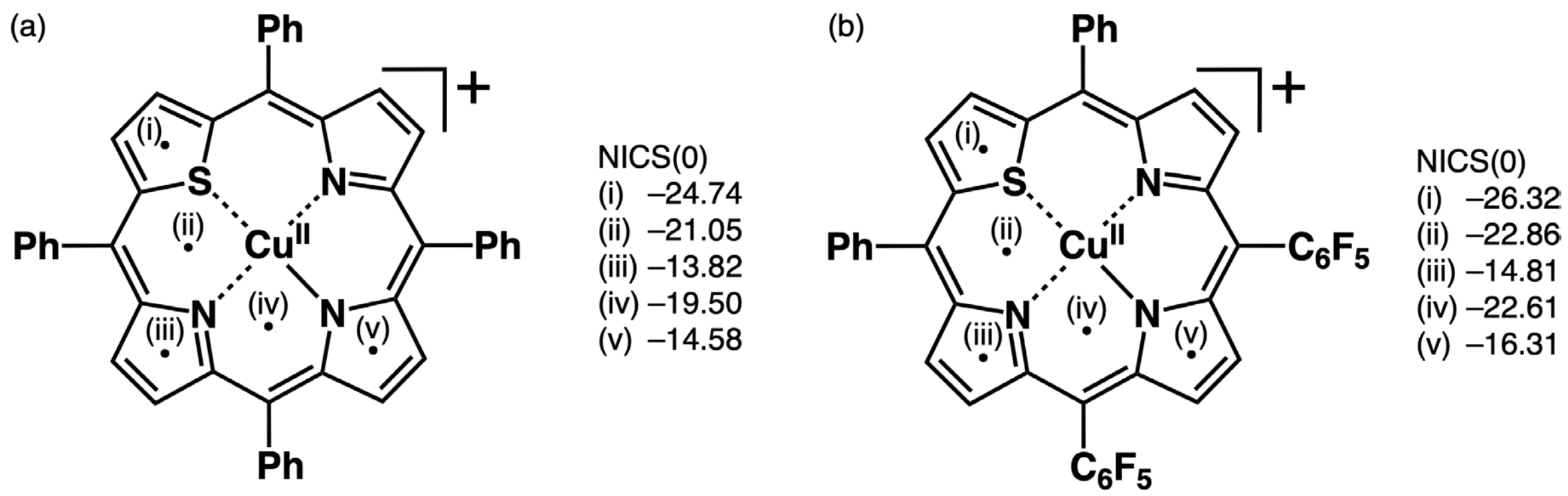

Fig. S85 NICS values (ppm)[S25] of (a) **1cu**$^+$ and (b) **2cu**$^+$ based on the optimized structures (Fig. S48) at PCM-UB3LYP/6-31+G(d,p) with LanL2DZ for Cu (CH_2Cl_2).

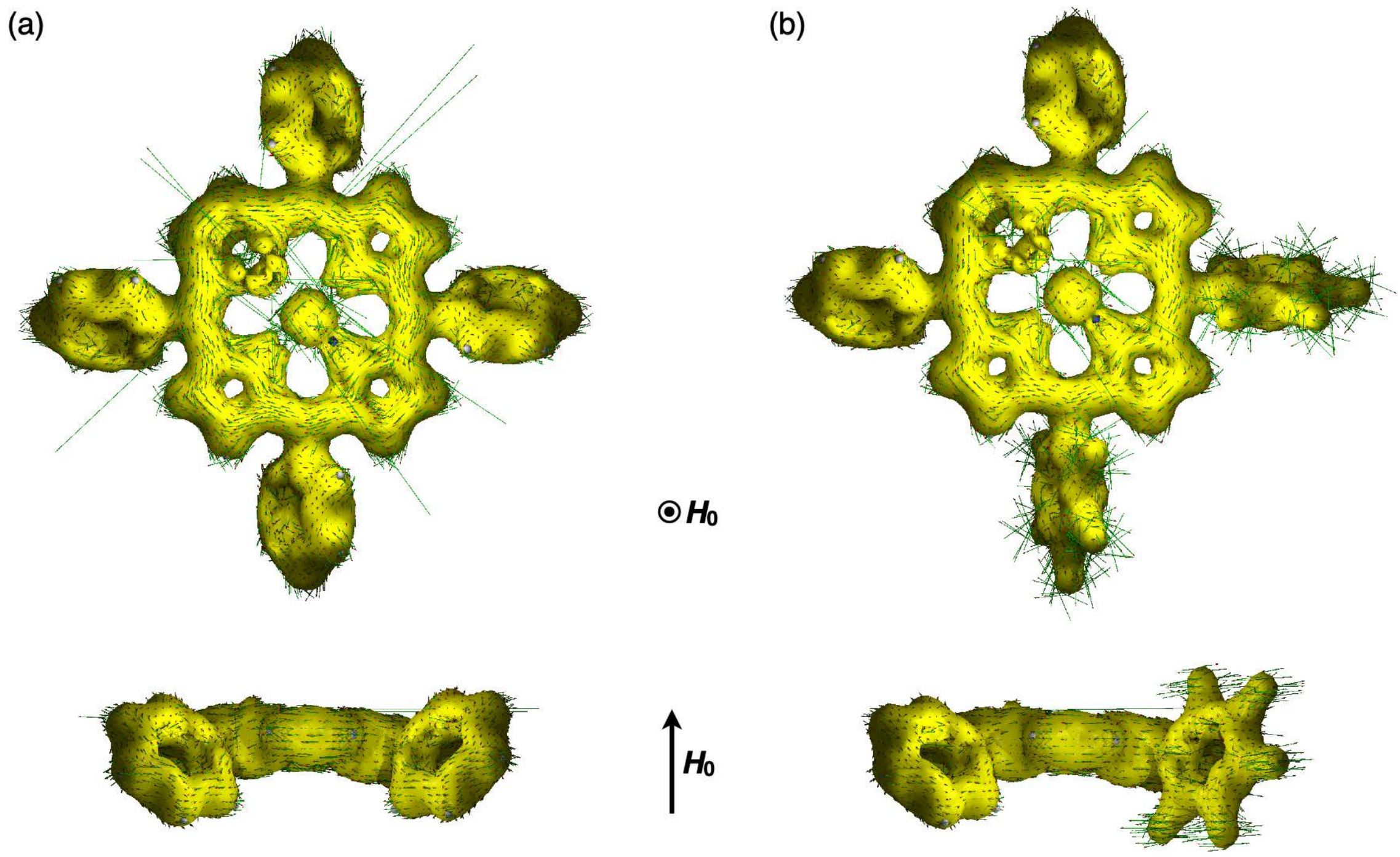

Fig. S86 Anisotropy of the induced current density (ACID)[S26] of (a) **1cu**$^+$ and (b) **2cu**$^+$ (top and side views) at isosurface value of δ = 0.015 based on the optimized structures (Fig. S48) at PCM-UB3LYP/6-31+G(d,p) with LanL2DZ for Cu (CH_2Cl_2). Current density vectors are plotted on to the ACID isosurface based on the vector of the magnetic field (H_0) which is orthogonal with respect to the molecule. The theoretical results were consistent with the NICS values (Fig. S85).

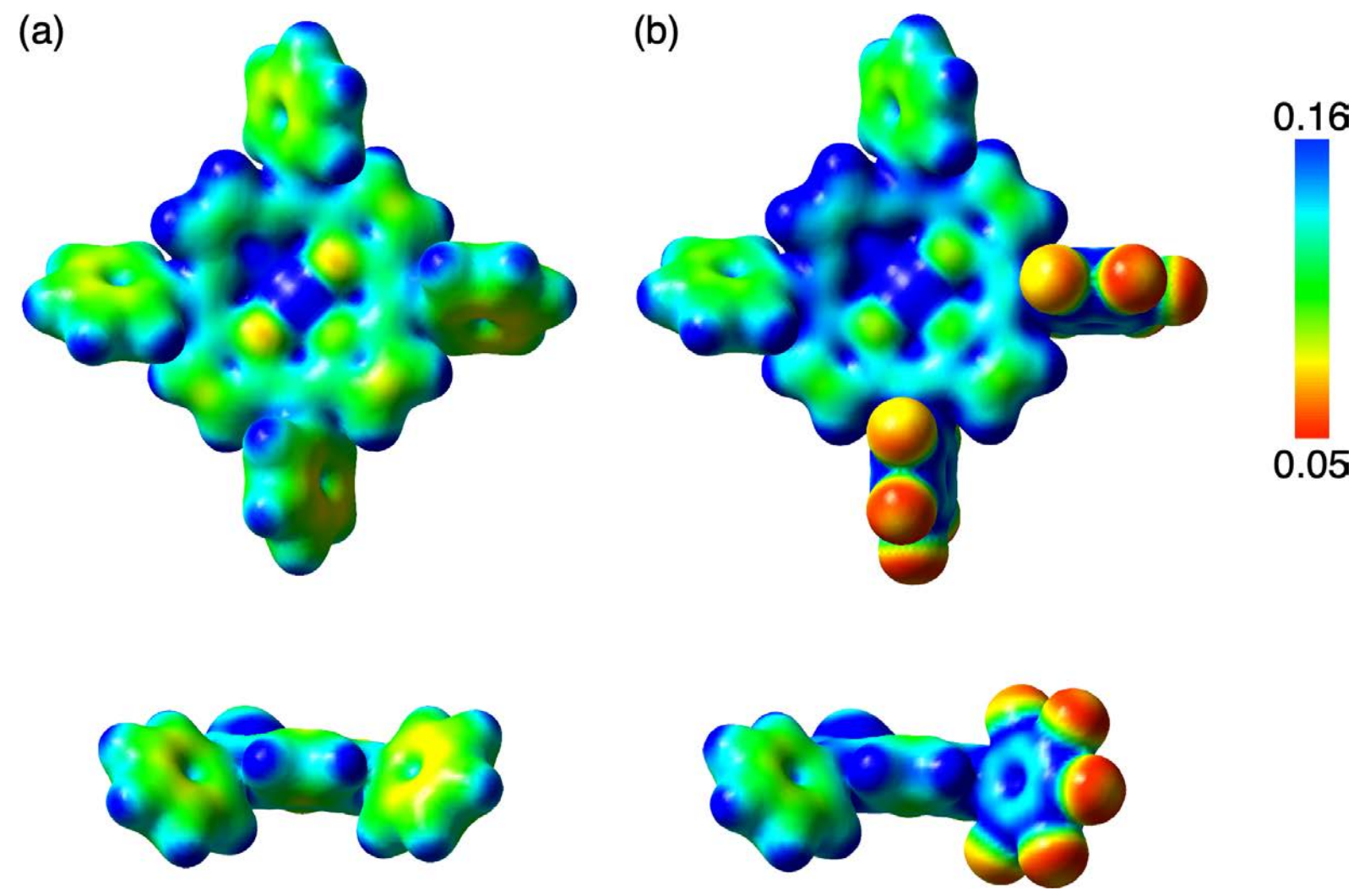

Fig. S87 Electrostatic potential (ESP) mapping (top and side views, δ = 0.01) of (a) **1cu**$^+$ and (b) **2cu**$^+$ at calculated at UB3LYP/6-31+G(d,p) with LanL2DZ for Cu based on the optimized structures (Fig. S48).

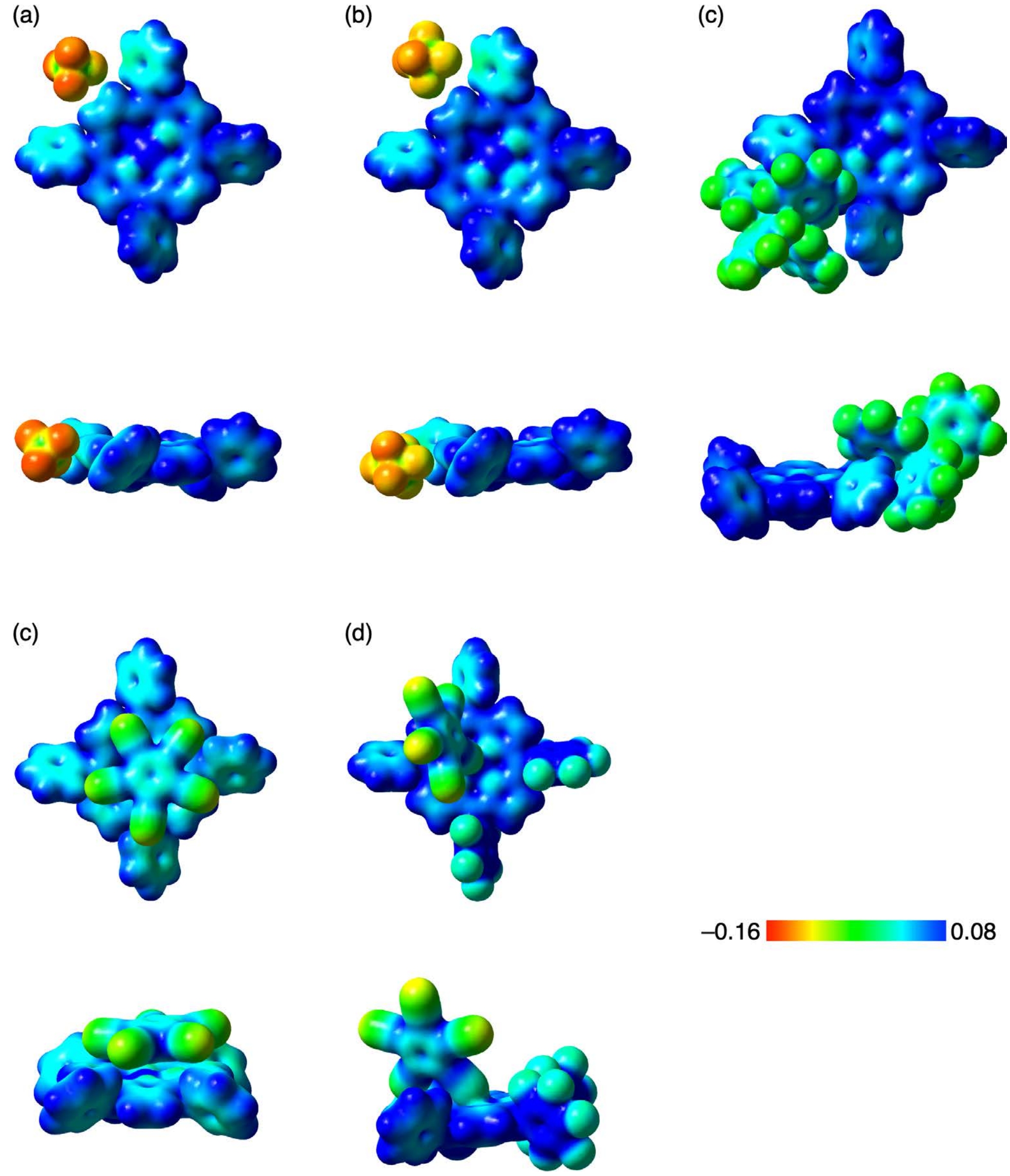

Fig. S88 Electrostatic potential (ESP) mapping (top and side views, δ = 0.01) of (a) **1cu**$^{+}$-BF_4^{-}, (b) **1cu**$^{+}$-PF_6^{-}, (c) **1cu**$^{+}$-$B(C_6F_5)_4^{-}$, (d) **1cu**$^{+}$-$PCCp^{-}$, and (e) **2cu**$^{+}$-$PCCp^{-}$ calculated at UB3LYP/6-31+G(d,p) with LanL2DZ for Cu. Single-crystal X-ray structures (Fig. S14,16,18,20,22) were used for the calculations of corresponding ion pairs.

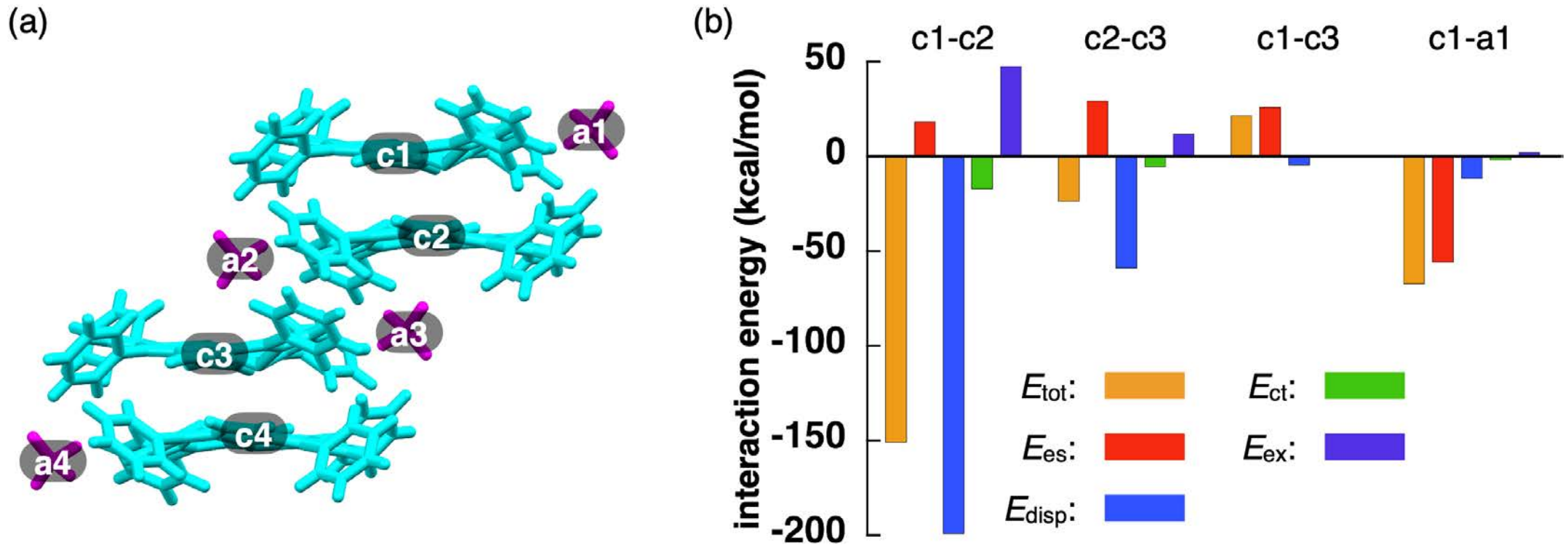

Fig. S89 PIEDA[S27] of **1cu**$^+$-BF_4^-: (a) single-crystal X-ray structure and (b) intermolecular interaction energies (kcal/mol) between selected ions estimated at an FMO2-MP2 using mixed basis sets including NOSeC-V-DZP with MCP with TZP for Cu.[S28–30] The labels (c1–4 and a1–4) correspond to the fragments shown in Table S36.

Table S36 Energies between selected fragments in **1cu**$^+$-BF_4^- (Fig. S89) estimated by EDA calculations[S27] based on an FMO2-MP2 using mixed basis sets including NOSeC-V-DZP with MCP with TZP for Cu.[S28–30]

fragments	total interaction energy (E_{tot}) (kcal/mol)	electrostatic interaction energy (E_{es}) (kcal/mol)	dispersion interaction energy (E_{disp}) (kcal/mol)	charge-transfer interaction energy (E_{ct+mix}) (kcal/mol)	exchange repulsion interaction energy (E_{ex}) (kcal/mol)
c1-a1	–67.230	–55.903	–11.622	–2.008	2.303
c2-a1	–53.772	–44.580	–8.808	–1.676	1.292
c2-c1	–150.896	18.241	–199.336	–17.295	47.494
a3-a1	26.929	26.929	0.000	0.000	0.000
a3-c1	–30.035	–30.035	0.000	0.000	0.000
a3-c2	–77.885	–58.296	–19.186	–3.225	2.821
a2-a1	21.928	21.928	0.000	0.000	0.000
a2-c1	–53.122	–44.061	–8.747	-1.611	1.297
a2-c2	–61.581	–50.718	–11.394	–1.848	2.379
a2-a3	39.752	39.752	0.000	0.000	0.000
c3-a1	–18.540	–18.540	0.000	0.000	0.000
c3-c1	21.418	25.979	–4.527	–0.033	0.000
c3-c2	–23.566	29.191	–59.096	–5.455	11.793
c3-a3	–61.429	–50.586	–11.374	–1.847	2.378
c3-a2	–77.510	–57.905	–19.212	–3.212	2.820
c4-a1	–16.401	–16.401	0.000	0.000	0.000
c4-c1	19.437	19.437	0.000	0.000	0.000
c4-c2	21.523	26.081	–4.525	–0.033	0.000
c4-a3	–53.151	–44.090	–8.749	–1.609	1.297
c4-a2	–30.001	–30.001	0.000	0.000	0.000
c4-c3	–150.487	18.728	–199.504	–17.279	47.569
a4-a1	12.622	12.622	0.000	0.000	0.000
a4-c1	–16.388	–16.388	0.000	0.000	0.000
a4-c2	–18.608	–18.608	0.000	0.000	0.000
a4-a3	21.925	21.925	0.000	0.000	0.000
a4-a2	26.926	26.926	0.000	0.000	0.000
a4-c3	–55.096	–45.815	–8.883	–1.683	1.286
a4-c4	–66.968	–55.647	–11.618	–2.005	2.302

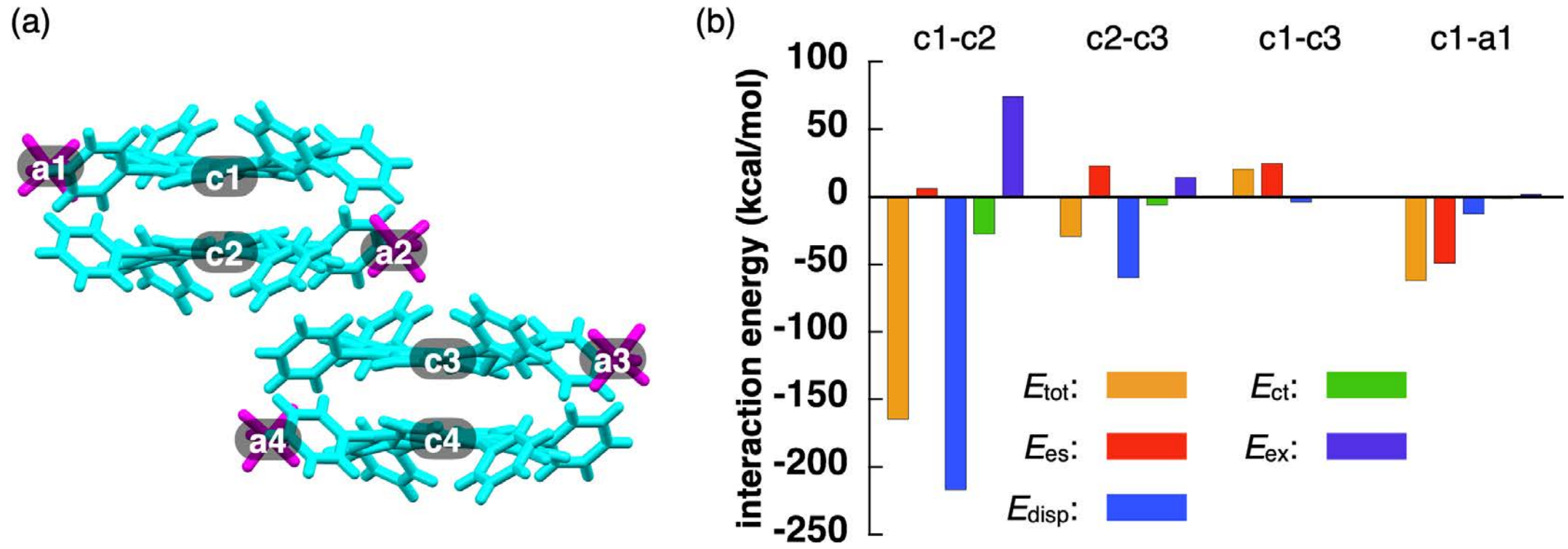

Fig. S90 PIEDA[S27] of **1cu**$^+$-PF_6^-: (a) single-crystal X-ray structure and (b) intermolecular interaction energies (kcal/mol) between selected ions estimated at an FMO2-MP2 using mixed basis sets including NOSeC-V-DZP with MCP with TZP for Cu.[S28–30] The labels (c1–4 and a1–4) correspond to the fragments shown in Table S37.

Table S37 Energies between selected fragments in **1cu**$^+$-PF_6^- (Fig. S90) estimated by EDA calculations[S27] based on an FMO2-MP2 using mixed basis sets including NOSeC-V-DZP with MCP with TZP for Cu.[S28–30]

fragments	total interaction energy (E_{tot}) (kcal/mol)	electrostatic interaction energy (E_{es}) (kcal/mol)	dispersion interaction energy (E_{disp}) (kcal/mol)	charge-transfer interaction energy (E_{ct+mix}) (kcal/mol)	exchange repulsion interaction energy (E_{ex}) (kcal/mol)
a1-c1	–62.187	–49.214	–12.949	–1.634	1.611
c4-c1	20.042	20.042	0.000	0.000	0.000
c4-a1	–23.454	–23.454	0.000	0.000	0.000
c2-c1	–164.753	5.856	–216.895	–27.794	74.079
c2-a1	–50.643	–39.108	–11.541	–1.465	1.472
c2-c4	19.641	23.933	–4.250	–0.043	0.000
a2-c1	–57.103	–45.558	–11.446	–1.528	1.428
a2-a1	20.805	20.805	0.000	0.000	0.000
a2-c4	–18.196	–18.196	0.000	0.000	0.000
a2-c2	–65.918	–52.956	–12.938	–1.637	1.613
a4-c1	–16.041	–16.041	0.000	0.000	0.000
a4-a1	22.148	22.148	0.000	0.000	0.000
a4-c4	–61.363	–48.317	–13.004	–1.654	1.612
a4-c2	–16.965	–16.965	0.000	0.000	0.000
a4-a2	12.750	12.750	0.000	0.000	0.000
c3-c1	20.295	24.304	–3.936	–0.076	0.002
c3-a1	–34.310	–33.217	–1.087	–0.007	0.000
c3-c4	–171.454	5.800	–224.032	–27.936	74.714
c3-c2	–29.471	22.591	–59.999	–6.041	13.978
c3-a2	–18.865	–18.865	0.000	0.000	0.000
c3-a4	–58.216	–46.694	–11.437	–1.513	1.428
a3-c1	–32.369	–31.529	–0.793	–0.046	0.000
a3-a1	30.804	30.804	0.000	0.000	0.000
a3-c4	–56.351	–44.808	–11.532	–1.475	1.464
a3-c2	–65.011	–49.742	–14.985	–1.848	1.564
a3-a2	31.776	31.776	0.000	0.000	0.000
a3-a4	20.796	20.796	0.000	0.000	0.000
a3-c3	–58.903	–46.066	–12.915	–1.578	1.657

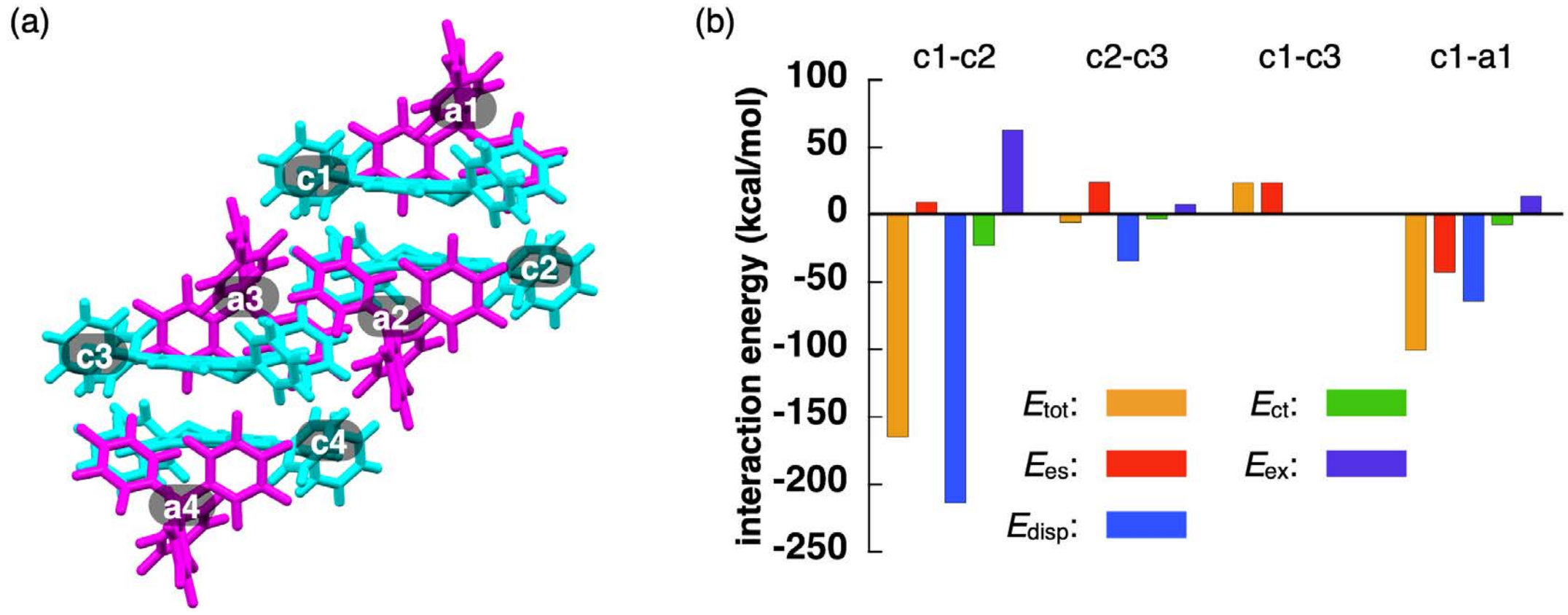

Fig. S91 PIEDA[S27] of **1cu**$^+$-B$(C_6F_5)_4^-$: (a) single-crystal X-ray structure and (b) intermolecular interaction energies (kcal/mol) between selected ions estimated at an FMO2-MP2 using mixed basis sets including NOSeC-V-DZP with MCP with TZP for Cu.[S28–30] The labels (c1–4 and a1–4) correspond to the fragments shown in Table S38.

Table S38 Energies between selected fragments in **1cu**$^+$-B$(C_6F_5)_4^-$ (Fig. S91) estimated by EDA calculations[S27] based on an FMO2-MP2 using mixed basis sets including NOSeC-V-DZP with MCP with TZP for Cu.[S28–30]

fragments	total interaction energy (E_{tot}) (kcal/mol)	electrostatic interaction energy (E_{es}) (kcal/mol)	dispersion interaction energy (E_{disp}) (kcal/mol)	charge-transfer interaction energy (E_{ct+mix}) (kcal/mol)	exchange repulsion interaction energy (E_{ex}) (kcal/mol)
c1-a1	–100.358	–42.514	–64.384	–7.332	13.871
a4-a1	12.132	12.132	0.000	0.000	0.000
a4-c1	–16.083	–16.083	0.000	0.000	0.000
c3-a1	–16.903	–16.903	0.000	0.000	0.000
c3-c1	23.816	23.816	0.000	0.000	0.000
c3-a4	–68.916	–35.855	–35.430	–2.457	4.827
a2-a1	19.758	19.758	0.000	0.000	0.000
a2-c1	–67.719	–34.311	–35.814	–2.523	4.929
a2-a4	23.825	24.948	–1.122	–0.002	0.000
a2-c3	–76.306	–36.223	–41.862	–3.699	5.479
c2-a1	–69.229	–35.898	–35.781	–2.463	4.914
c2-c1	–165.095	9.048	–213.921	–22.991	62.769
c2-a4	–16.877	–16.877	0.000	0.000	0.000
c2-c3	–5.621	24.211	–34.448	–3.136	7.753
c2-a2	–97.926	–41.062	–63.423	–7.424	13.984
c4-a1	–16.081	–16.081	0.000	0.000	0.000
c4-c1	19.889	19.889	0.000	0.000	0.000
c4-a4	–100.369	–42.520	–64.392	–7.334	13.876
c4-c3	–164.260	8.954	–211.603	–23.331	61.720
c4-a2	–55.525	–32.082	–23.476	–1.887	1.919
c4-c2	23.780	23.780	0.000	0.000	0.000
a3-a1	23.849	24.973	–1.122	–0.002	0.000
a3-c1	–55.562	–32.103	–23.489	–1.888	1.919
a3-a4	19.754	19.754	0.000	0.000	0.000
a3-c3	–98.687	–41.087	–64.280	–7.417	14.097
a3-a2	26.659	26.659	0.000	0.000	0.000
a3-c2	–76.196	–36.365	–41.581	–3.708	5.458
a3-c4	–67.668	–34.286	–35.798	–2.517	4.933

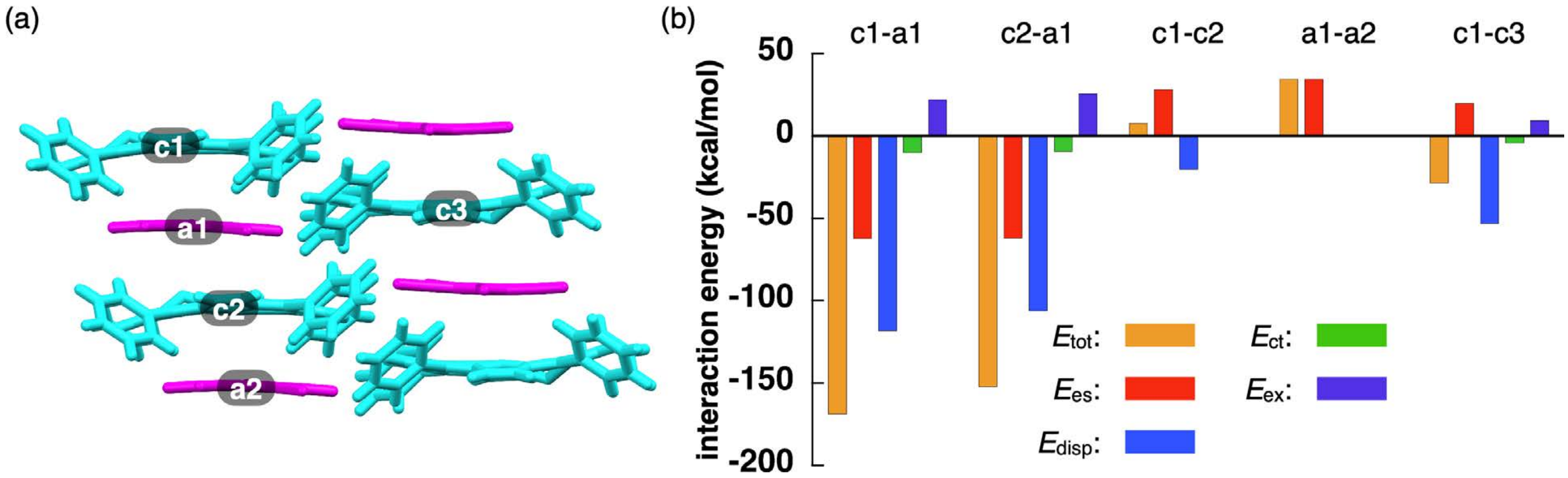

Fig. S92 PIEDA[S27] of **1cu**$^+$-PCCp$^-$: (a) single-crystal X-ray structure and (b) intermolecular interaction energies (kcal/mol) between selected ions estimated at an FMO2-MP2 using mixed basis sets including NOSeC-V-DZP with MCP with TZP for Cu.[S28–30] The labels (c1–4 and a1–4) correspond to the fragments shown in Table S39. For this system, two-fragment models (c1-a1, c2-a1, c1-c2, a1-a2, and c1-c3) were employed.

Table S39 Energies between selected fragments in **1cu**$^+$-PCCp$^-$ (Fig. S92) estimated by EDA calculations[S27] based on an FMO2-MP2 using mixed basis sets including NOSeC-V-DZP with MCP with TZP for Cu.[S28–30]

fragments	total interaction energy (E_{tot}) (kcal/mol)	electrostatic interaction energy (E_{es}) (kcal/mol)	dispersion interaction energy (E_{disp}) (kcal/mol)	charge-transfer interaction energy (E_{ct+mix}) (kcal/mol)	exchange repulsion interaction energy (E_{ex}) (kcal/mol)
c1-a1	–168.704	–62.284	–118.377	–10.140	22.096
c2-a1	–152.297	–62.183	–106.160	–9.582	25.628
c1-c2	7.655	28.225	-20.426	–0.150	0.006
a1-a2	34.268	34.268	0.000	0.000	0.000
c1-c3	–28.641	19.884	–53.307	–4.357	9.139

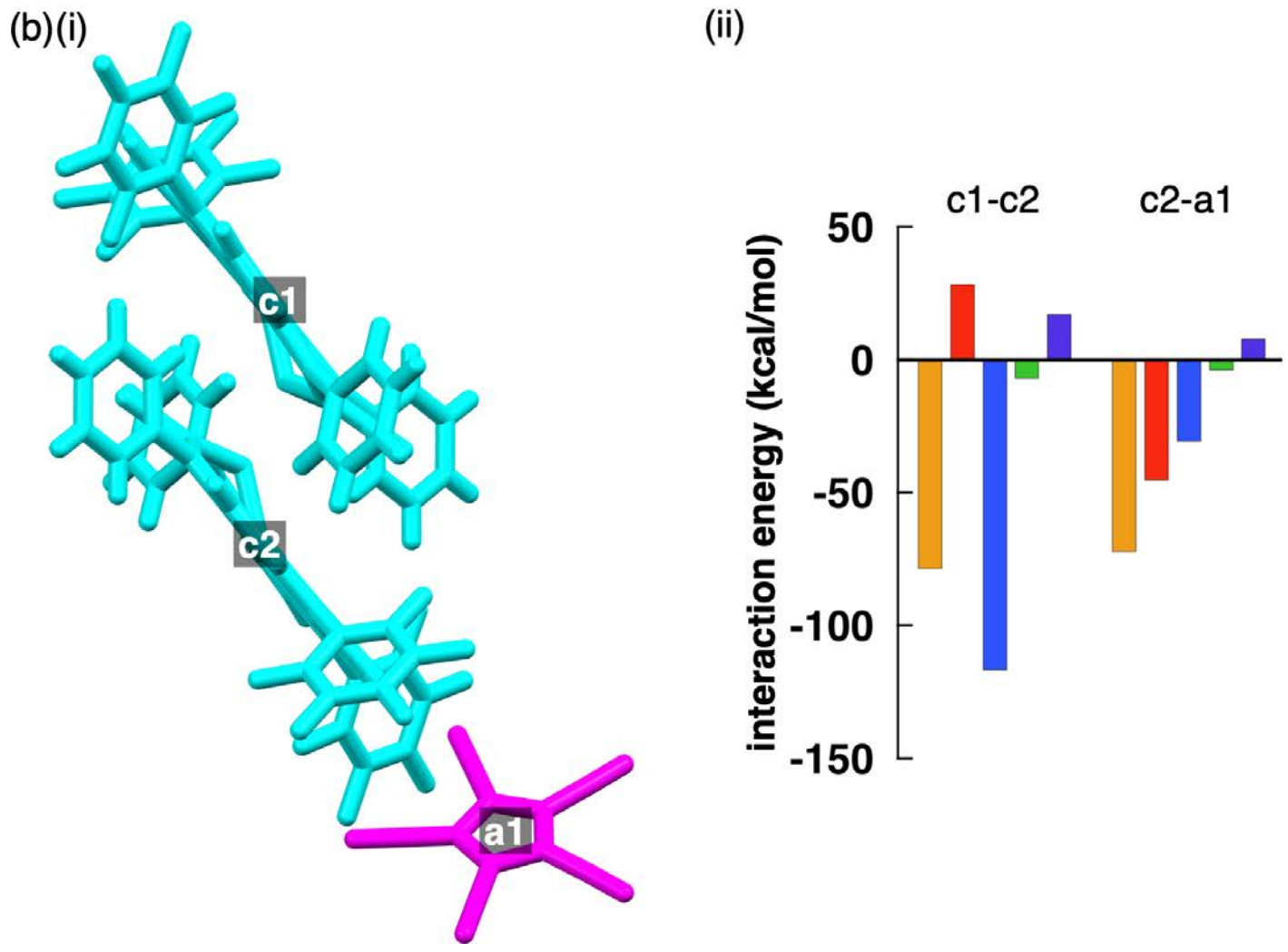

Fig. S93 PIEDA[S27] of **2cu**$^+$-PCCp$^-$: (a) single-crystal X-ray structure and (b) intermolecular interaction energies (kcal/mol) between selected ions estimated at an FMO2-MP2 using mixed basis sets including NOSeC-V-DZP with MCP with TZP for Cu.[S28–30] The labels (c1–4 and a1–4) correspond to the fragments shown in Table S40. For this system, two-fragment models (c1-a1, c2-a1, c1-c2, a1-a2, and c1-c3) were employed.

Table S40 Energies between selected fragments in **2cu**$^+$-PCCp$^-$ (Fig. S93) estimated by EDA calculations[S27] based on an FMO2-MP2 using mixed basis sets including NOSeC-V-DZP with MCP with TZP for Cu.[S28–30]

fragments	total interaction energy (E_{tot}) (kcal/mol)	electrostatic interaction energy (E_{es}) (kcal/mol)	dispersion interaction energy (E_{disp}) (kcal/mol)	charge-transfer interaction energy (E_{ct+mix}) (kcal/mol)	exchange repulsion interaction energy (E_{ex}) (kcal/mol)
c1-c2	–78.654	28.156	–116.71	–7.0740	16.976
c2-a1	–72.214	–45.273	–30.673	–3.8940	7.6270

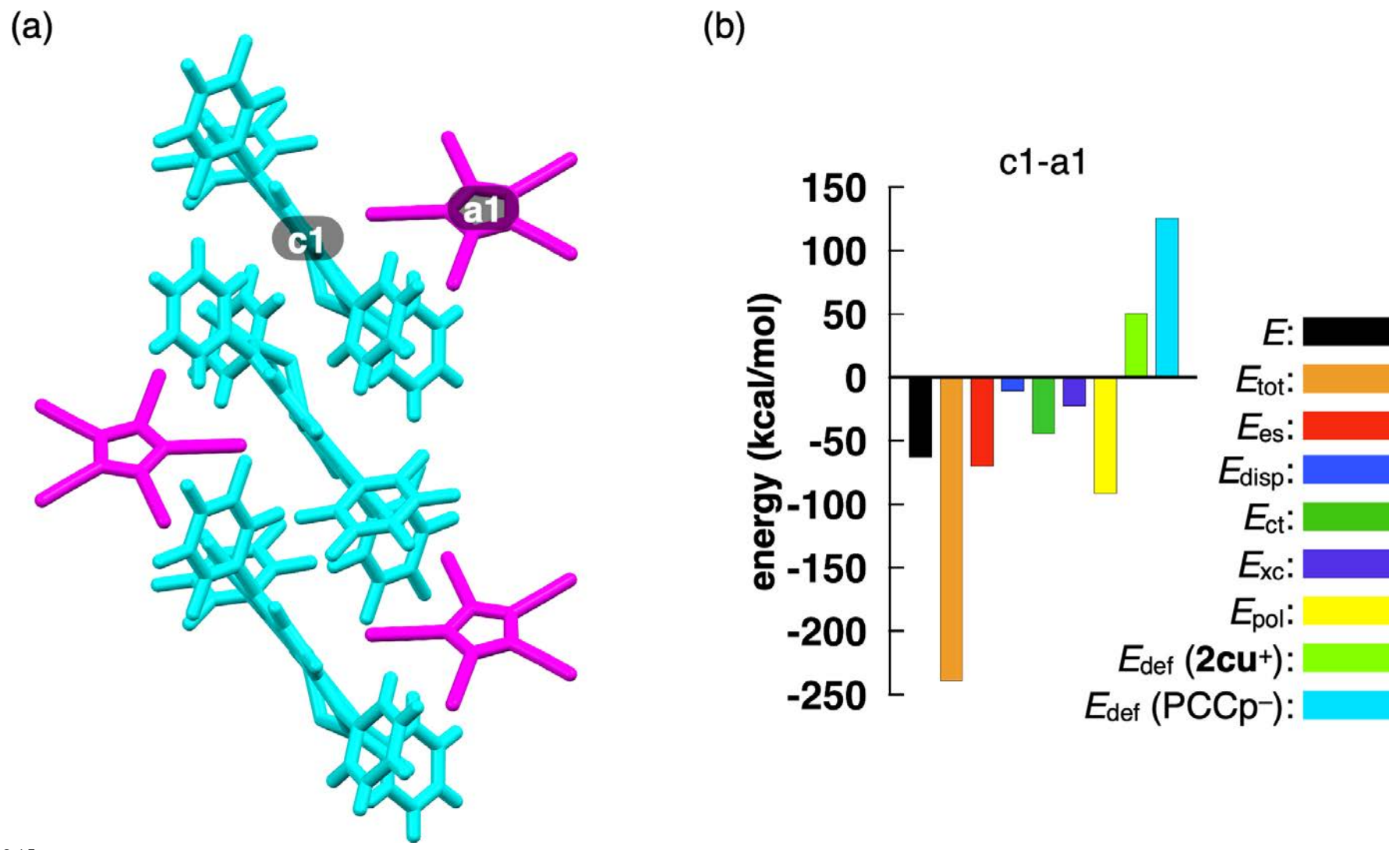

Fig. S94 NEDA[S31] of **2cu**$^+$-PCCp$^-$: (a) single-crystal X-ray structure and (b) deformation energy (kcal/mol) of each fragment and intermolecular interaction energies (kcal/mol) between the ions estimated at B3LYP-D3/6-311+G(d,p). The labels (c1 and a1) correspond to the fragments shown in Table S41. For this system, a two-fragment model (c1-a1) based on the dimeric packing structure was employed. NEDA decomposes a total interaction energy (E_{tot}) into electrical (E_{el}), core (E_{core}), and charge transfer (E_{ct}) components:

$$E_{\text{tot}} = E_{\text{el}} + E_{\text{core}} + E_{\text{ct}}$$

The E_{el} term consists of an electrostatic energy (E_{es}) and an induced polarization energy (E_{pol}), along with an associated self-energy correction term (E_{se}) that accounts for the energetic penalty at each polarizing center:

$$E_{\text{el}} = E_{\text{es}} + E_{\text{pol}} + E_{\text{se}}$$

The E_{core} term consists of exchange-correlation (E_{xc}), dispersion (E_{disp}), and deformation (E_{def}) energies with E_{se} subtracted to represent the net core repulsion in NEDA:

$$E_{\text{core}} = E_{\text{xc}} + E_{\text{disp}} + E_{\text{def}} - E_{\text{se}}$$

In this ion pair, E_{el}, E_{core}, and E_{ct} contribute to the interaction associated with the coordination of PCCp$^-$ to the Cu^{II} center in **2cu**$^+$. These energy components are summarized in Table S41.

Table S41 Energies between selected fragments in **2cu**$^+$-PCCp$^-$ (Fig. S94) estimated by NEDA calculations[S31] based on B3LYP-D3/6-311+G(d,p).

total interaction energy (E_{tot}) (kcal/mol)	electrostatic interaction energy (E_{es}) (kcal/mol)	polarization interaction energy (E_{pol}) (kcal/mol)	dispersion interaction energy (E_{disp}) (kcal/mol)	charge-transfer interaction energy (E_{ct}) (kcal/mol)	exchange repulsion/attraction and correation interaction energy (E_{xc}) (kcal/mol)	deformation energy (E_{def}) (self-energy (E_{se})) of **2cu**$^+$ (kcal/mol)	deformation energy (E_{def}) (self-energy (E_{se})) of PCCp$^-$ (kcal/mol)
–238.9	–70.1	–91.6	–10.6	–44.2	–22.5	50.5 (13.6)	125.4 (33.9)

Cartesian coordination of optimized structures

Cartesian Coordination of 1cu$^+$

–2452.0411841 hartree
H,12.4394342621,14.7525360388,20.8889867223
C,11.5623450937,14.5295661286,20.2889978276
C,10.526814831,13.7539625982,20.8197969998
C,9.4052515566,13.4614787088,20.0372744689
H,10.593184603,13.3801900925,21.8370451738
H,8.5938656447,12.8683141088,20.4481549545
H,5.512398091,20.2337567668,7.7420881606
C,8.3429968322,11.8821463073,12.1327850673
C,7.3704764835,11.1277675768,12.8113867584
C,7.0750300311,9.8242945698,12.4060739845
C,7.7565782218,9.252502906,11.3276197821
C,8.7300661211,9.9929441422,10.650370286
C,9.0165868879,11.3019531716,11.0442482118
H,6.3125305147,9.2574041807,12.9318599594
H,7.5302789763,8.237090436,11.0167114681
H,9.2675747849,9.5528333716,9.8157535224
H,9.7770709521,11.8717691464,10.51799124
C,10.0751927789,12.9313882385,15.8515031391
C,9.6359729598,12.3846196177,14.6839506756
C,9.2273602721,13.4750493993,13.8300660347
C,8.6506170839,13.2839943437,12.5541336281
C,8.3344711042,14.3140134976,11.6437933372
C,7.6332394491,14.1404429722,10.3908639211
C,7.5435727699,15.3710429026,9.8090149576
C,8.1890370209,16.3094396439,10.7003379569
C,8.3291967131,17.6938162743,10.4691846616
C,7.8816251957,18.212829585,9.1396541974
C,8.5417219679,17.8192973478,7.9629400179
C,8.1245014513,18.3036564328,6.7211409826
C,7.0335210658,19.1738561349,6.6367951252
C,6.3651067733,19.5639585476,7.8010768131
C,6.7898757857,19.0929308597,9.0453922101

C,8.851997843,18.6241073802,11.3956598907
C,9.0797452754,20.0139570642,11.0769538538
C,9.514318692,20.6213763937,12.2159045022
C,9.5803508035,19.6115338846,13.2420955566
C,9.9272072255,19.9153842164,14.59128938
C,9.8739176387,21.3277561124,15.0575795207
C,8.7238024862,22.1149737574,14.8595989823
C,8.6775696346,23.4298567929,15.3244657368
C,9.7801369188,23.981696941,15.9844708927
C,10.9286520573,23.2095938681,16.1854732471
C,10.9731585978,21.8900819637,15.7338012002
C,10.2419156055,18.9345812704,15.5387130173
C,10.1208573295,18.9477107222,16.9500062355
C,10.2121864309,17.6964466458,17.5415480068
C,10.4098675929,16.6331551273,16.6267230341
C,10.2675563447,15.2512596308,16.796321935
C,10.3563963746,14.7173956416,18.1827348251
C,11.4749991985,15.0153750281,18.9838318097
C,9.3209090453,13.9344394376,18.7271795767
C,9.9632419643,14.362580245,15.7236589881
Cu,9.3169545361,16.4608785113,13.4404659585
H,10.4749896631,12.4170648877,16.7126628875
H,9.6040520102,11.3392182589,14.4168949093
H,6.8338017894,11.5703747167,13.645676314
H,7.2430378713,13.2051332889,10.0185154397
H,7.0667223914,15.6251683077,8.8742749376
H,9.3928085835,17.147064403,8.0239957891
H,8.6520419948,18.0014448263,5.8213803405
H,6.7061707734,19.5449648355,5.6703292991
H,6.2634784277,19.393422552,9.9468201909
H,8.9391206391,20.4607043997,10.1044081202
H,9.8011979398,21.6542488754,12.3456567248
H,7.860043913,21.6873225825,14.3597114322
H,7.77966558,24.0218325305,15.1749803889
H,9.7443374926,25.0068710982,16.3403394033
H,11.7908728454,23.6355568091,16.6895745341
H,11.871970474,21.2980540563,15.8803281089
H,9.8666523904,19.8507907025,17.4910932435
H,10.03705046,17.5158431638,18.5949527177
H,12.2874441319,15.6064014962,18.5709759665
H,8.4418625085,13.7155853835,18.1286568645
N,9.4585065234,14.6784130727,14.4685679912
N,9.1880435323,18.3876343991,12.7148904555
N,8.6493427804,15.6381541394,11.8026956542
S,10.7485474102,17.3237445315,15.0441498913

Cartesian Coordination of 2cu^{+}

–3444.3444489 hartree
C,-3.7921721852,-3.4342069544,-1.0819898741
C,-4.7786186661,-4.4173080878,-1.0300818021
C,-5.5009675068,-4.6025482419,0.149321601
F,-3.1152775839,-3.2718321438,-2.2308638471
F,-5.0357341947,-5.1773943369,-2.0978392196
F,-6.4457671935,-5.5393430321,0.2124881887
C,3.8050423544,5.4915634797,-0.7791558559
C,4.8018612946,6.4326867848,-0.5204635586
C,5.6561703461,6.26699772,0.5744883301
C,5.5133619429,5.1527006912,1.4074167473
C,4.5273995991,4.2013211365,1.1434957801
C,3.6615142437,4.3583299971,0.0442775304
C,2.5884216066,3.3668107078,-0.2343147034
C,2.9111234026,1.985026354,-0.3053788927
C,4.2696437242,1.5134554174,-0.4336024707
C,4.2333419534,0.1550111059,-0.3818457189
C,2.8510578083,-0.2272589817,-0.2091252435
C,2.4334206622,-1.5657572679,-0.0525376474
C,3.5015948399,-2.6138430806,0.0159717527
C,4.2448852336,-2.8243882227,1.1828288723
C,5.2355242068,-3.8008727822,1.2633395775
C,5.5045622173,-4.5981478781,0.1501151473
C,4.7822415831,-4.4134754245,-1.0293947068
C,3.7950181394,-3.4311621689,-1.0814425612
C,1.1050065257,-2.0062566158,0.0483934699
C,0.6817199971,-3.3746739319,0.2679161864
C,-0.6791098006,-3.3752224768,0.2678146956
C,-1.1034644306,-2.0071481859,0.0482222173
C,-2.4322151654,-1.5677115066,-0.0528989443
C,-3.4995646066,-2.6166467078,0.0154628097
C,-5.232731069,-3.8050516468,1.2625809546
C,-4.2428591639,-2.8277777833,1.1822105402
C,-2.8508862081,-0.2295453987,-0.2095412235
C,-4.2334507442,0.151643153,-0.3824100453
C,-4.2708137469,1.5100601807,-0.4341196325
C,-2.9126700637,1.982688439,-0.3058055122
C,-2.5910724286,3.364726339,-0.2346870556
C,-3.6650034894,4.3553702737,0.0437818245
C,-3.8092793953,5.4885576804,-0.7795835261
C,-4.8069032372,6.4288588267,-0.5210033385
C,-4.5309866725,4.1975602948,1.1428072135
C,-1.2757489411,3.8486721098,-0.3255990161
C,-0.6974820938,5.0206456785,0.2091415043
C,0.6934355123,5.0212070601,0.2092391873
C,1.272721744,3.8497000575,-0.3254199033
Cu,-0.0003244428,0.7350000084,-0.217738472
F,4.0016775983,-2.0731314425,2.2702758027
F,6.4500996164,-5.5341884167,0.2134154472
F,5.0401241479,-5.1733477545,-2.0971193201
F,3.1181676473,-3.2693174428,-2.2304175982
F,-5.9214006438,-3.982029627,2.3936062853
F,-4.0004112759,-2.076319845,2.2696883585
H,3.1522413195,5.6172742609,-1.6381756923
H,4.9142584529,7.2917418383,-1.1748241644
H,6.4280620588,7.002838577,0.7782085192
H,6.1666192441,5.0260761517,2.2653285733
H,4.4108126182,3.3463825698,1.8026203598
H,5.1326955884,2.146237884,-0.5761142215
H,5.0667277214,-0.5270251309,-0.4630204973
H,1.3385851505,-4.22057144,0.4084869206
H,-1.335313855,-4.221649752,0.4082844894
H,-5.0662965523,-0.5310429682,-0.463664245
H,-5.1343501341,2.1421732878,-0.5766733744
H,-3.1564080577,5.6148687217,-1.6384620705
H,-4.9198595839,7.287882555,-1.1753087584
H,-4.4138542006,3.342646172,1.8018672829
H,-1.2982648755,5.7909533512,0.6767849919
H,1.2935303945,5.7919991004,0.6769674312
N,2.0529991325,0.8955896273,-0.1864791812
N,0.0004575332,-1.2077539616,-0.0749936765
N,-2.0537058667,0.8939268467,-0.1868046498
S,-0.0010731731,2.8902040897,-1.0675666471

F,5.9241669926,-3.9773097774,2.3944661896
C,-5.6612958077,6.2623858702,0.5737646837
C,-5.5177638142,5.148125376,1.4066179833
H,-6.4338189402,6.9975881866,0.7773966643
H,-6.1710953435,5.0208954528,2.2643837546

Cartesian Coordination of 1cu^{+}-Cl^{-}

–2912.4481691 hartree
H,6.3185296702,-6.1171068817,0.6583564537
C,5.5620137093,-5.367896417,0.4429230538
C,5.3223001253,-4.3302006905,1.3493639142
H,5.8850189476,-4.2759726375,2.2770047645
C,5.523921535,5.5416839138,0.1278398536
H,6.2960573937,6.303851644,0.1818666026
H,5.8896842984,4.7939094873,2.1196913761
H,5.0031787264,-6.2387579601,-1.4521264076
C,-3.6286571752,-3.3952498144,-0.1156672134
C,3.5218659378,3.5661821667,-0.0121353412
C,-4.2803673289,-0.5454423649,-0.417491385
C,-2.9096806454,-1.0051209584,-0.3332743947
C,-3.4943001921,3.5932481336,-0.0121231581
C,4.2586874868,0.7797585285,-0.3365070155
C,-2.5678613711,-2.3880637144,-0.4035203886
C,2.4547489273,2.5148812934,-0.0816545888
C,2.8730532005,1.172957887,-0.1812786287
C,-1.0917461716,2.9621524512,-0.0553803564
C,3.7559808549,4.4260392746,-1.0974631722
C,-4.2525991155,0.8125906312,-0.3364778472
C,-2.8639712156,1.1950885702,-0.1812612344
C,-4.8623451282,-5.401039399,-0.7405113964
C,-2.435325966,2.5337453459,-0.0816444758
C,-3.8794747516,-4.4478151445,-1.0145267535
C,-0.664999459,4.3408486921,0.0656877942
C,2.5493070257,-2.4078035633,-0.4035453479
C,-3.7217827523,4.4548789319,-1.0974546349
C,-5.6033114393,-5.3248210761,0.4429771498
C,2.9017850198,-1.027538674,-0.3332997349
C,-0.7064861056,-4.0878173579,-0.1906280605
C,1.1145142256,2.9536413281,-0.0553835311
C,-1.2721558129,-2.8630007728,-0.6320404222
C,-4.3787696504,-3.3306408476,1.0729697992
C,1.2499739755,-2.8727305764,-0.6320543086
C,4.2759763851,-0.5784476414,-0.4175306345
C,3.6023030372,-3.4231442899,-0.1157027098
C,0.6748761128,-4.0931464469,-0.190635752
C,0.6984178747,4.3355893281,0.0656824466
C,4.30562336,3.709980224,1.1442536403
C,3.8449893029,-4.4776081965,-1.0145693016
C,4.7514920479,5.4043942619,-1.0293761838
C,-5.3555962805,-4.2889998453,1.3494106522
C,4.3528953453,-3.3643322742,1.0729324695
C,5.2969865666,4.6924095533,1.2148779876
C,4.8204772572,-5.4383885947,-0.7405633214
Cl,-0.004150152,-1.0771395815,2.2183706198
H,-5.1393997299,-1.1818181519,-0.571460735
H,5.0974822478,1.4568491219,-0.400410965
H,3.1602386408,4.3190567326,-1.9996287522
H,-5.0861456646,1.496132097,-0.4003782471
H,-5.0512185784,-6.1999797761,-1.4520688879
H,-3.3110161381,-4.5040065858,-1.9385392114
H,-3.1268906237,4.34329777,-1.9996241157
H,-6.3655843754,-6.0681713349,0.6584177282
H,-1.3103503946,-4.8721133231,0.2482257069
H,-4.1736494945,-2.5392128585,1.7871910171
H,5.1300719955,-1.2214310067,-0.5715121454
H,1.2726758958,-4.8820782534,0.2482114167
H,1.3610549088,5.1836328736,0.1543309231
H,4.1282436157,3.0537992435,1.9915861263
H,3.2761109625,-4.5294065554,-1.9385801625
H,4.9237218183,6.0569166083,-1.8808286438
H,-5.9178771436,-4.2304260928,2.2770528463
H,4.1538886391,-2.5713506484,1.7871599672
N,0.008271724,2.1511345722,-0.1269658965
N,-2.0678469431,0.0739705959,-0.1887243799
N,2.0683031702,0.0580152818,-0.188740444
S,-0.0071858112,-1.8546807935,-1.3180147822
C,-5.481054026,5.5841377592,0.1278550508
C,-4.709715756,5.440885688,-1.0293660845
C,-4.2769158733,3.7430957001,1.1442709812
C,-5.2606695876,4.7331446309,1.2148967875
H,-6.2472863615,6.3522401715,0.1818830094
H,-1.3210735238,5.1939792388,0.1543406551
H,-4.8769127987,6.0947120765,-1.8808213063
H,-4.1045972697,3.0855710581,1.9916062317
H,-5.8525596342,4.8392199286,2.1197140692
Cu,0.000680473,0.1819873061,0.2701573661

Cartesian Coordination of 2cu^{+}-Cl^{-}

–3904.7644403 hartree
H,-6.3221157981,7.0094375175,0.5273648529
C,-5.5676477281,6.2519417781,0.3355094836
C,-5.3298296959,5.2438885188,1.2751092343
H,-5.8914213724,5.221267474,2.2045878885
C,-5.4940733149,-4.7032759946,0.0285552637
H,-5.0082180003,7.0614078359,-1.5864299991
C,3.6151768815,4.2861693689,-0.1622772883
C,-3.4961669516,-2.7057392694,-0.0306960857
C,4.2881989072,1.4323817565,-0.3455403065
C,2.9120969608,1.8890202839,-0.3092803467
C,3.495778694,-2.7062196408,-0.0310185532
C,-4.2612394851,0.0770661813,-0.2542011928
C,2.5648607315,3.2606255012,-0.4194794792
C,-2.4346544384,-1.6468187238,-0.0622308421
C,-2.8678981582,-0.3076060984,-0.1451258139
C,1.1014991802,-2.0866568354,-0.0421281816
C,-3.9662172194,-3.3081344418,-1.202528008
C,4.261238715,0.0764730419,-0.254461983
C,2.8678479961,-0.307992848,-0.1452936038
C,4.8276121721,6.2829197771,-0.8504528146
C,2.4344137271,-1.6471479847,-0.0624305837
C,3.853683891,5.3128194341,-1.0940352809
C,0.6800988297,-3.4751001743,-0.0257722711
C,-2.5643991795,3.260959624,-0.4194027624
C,3.965669115,-3.3086195917,-1.2029119823
C,5.5685421823,6.251226098,0.3352326706
C,-2.9118230498,1.8894038271,-0.3091914992
C,0.6926821474,4.9432088916,-0.2103319694
C,-1.1017989757,-2.0865085179,-0.0420225767
C,1.2578566872,3.7243778263,-0.6552649634
C,4.3641293015,4.2663008509,1.0287378363

C,-1.2573423641,3.724540726,-0.6552325421
C,-4.2879985721,1.4329756182,-0.3453185513
C,-3.6145632364,4.2866409319,-0.1621310277
C,-0.6919978989,4.9432982637,-0.210314263
C,-0.6805852574,-3.4750085378,-0.0256981697
C,-4.0631316025,-3.1397931006,1.1723890284
C,-3.8529487641,5.3133652462,-1.0938381509
C,-4.9505903259,-4.2946262903,-1.1880289607
C,5.3306023522,5.2432509543,1.2748853037
C,-4.3634965196,4.2668165435,1.0288962695
C,-5.0494256599,-4.1241690689,1.215639287
C,-4.8267380648,6.2835886523,-0.8501900801
Cl,0.0003563382,2.0366971039,2.1911665226
H,5.1506086457,2.0687528031,-0.4774713769
H,-5.1029976445,-0.599953197,-0.2780649882
H,5.1028904067,-0.6006750135,-0.2784296866
H,5.0091840884,7.0606795335,-1.5867328294
H,3.2844413918,5.3350363573,-2.0189691243
H,6.3231182308,7.0086270705,0.5270374662
H,1.2943776539,5.7282661494,0.229862731
H,4.1655006365,3.4972480971,1.7688232852
H,-5.150322736,2.0694777253,-0.4771799257
H,-1.2935808585,5.7284332816,0.2298956918
H,-1.3372748489,-4.3330982418,-0.0209988845
H,-3.2837222039,5.3355463449,-2.0187826766
H,5.8922074082,5.2205980685,2.2043551043
H,-4.1649629042,3.4976971035,1.7689381886
N,-0.0000955261,-1.2778980251,-0.0511104794
N,2.0685307444,0.8032229543,-0.1774954168
N,-2.0684094533,0.8034804563,-0.1774696246
S,0.0001823339,2.7037547019,-1.3257682596
C,5.4934075851,-4.7040402143,0.0280008683
C,4.9499043803,-4.2952506307,-1.1885273332
C,4.062758608,-3.1404162932,1.1720077721
C,5.0489170381,-4.1249331463,1.2151436681
H,1.3366731084,-4.3332785383,-0.0211391978
Cu,0.0000666095,0.6862298188,0.3337234107
F,-3.4660889765,-2.9365135483,-2.393139431
F,-5.3776546382,-4.8504232549,-2.3308590152
F,-6.4399135703,-5.6489884875,0.0564957529
F,-5.5711570514,-4.5169766709,2.3859538813
F,-3.6578182397,-2.6042676472,2.3342765462
F,3.4655163503,-2.9368667156,-2.3934718565
F,5.3768173697,-4.8510481228,-2.3314136169
F,6.4391168369,-5.6498870202,0.0558315079
F,5.5706680501,-4.5178752643,2.3854043364
F,3.6575949978,-2.6048939427,2.3339490042

[S22] M. J. Frisch, G. W. Trucks, H. B. Schlegel, G. E. Scuseria, M. A. Robb, J. R. Cheeseman, G. Scalmani, V. Barone, G. A. Petersson, H. Nakatsuji, X. Li, M. Caricato, A. V. Marenich, J. Bloino, B. G. Janesko, R. Gomperts, B. Mennucci, H. P. Hratchian, J. V. Ortiz, A. F. Izmaylov, J. L. Sonnenberg, D. Williams-Young, F. Ding, F. Lipparini, F. Egidi, J. Goings, B. Peng, A. Petrone, T. Henderson, D. Ranasinghe, V. G. Zakrzewski, J. Gao, N. Rega, G. Zheng, W. Liang, M. Hada, M. Ehara, K. Toyota, R. Fukuda, J. Hasegawa, M. Ishida, T. Nakajima, Y. Honda, O. Kitao, H. Nakai, T. Vreven, K. Throssell, J. A. Montgomery, Jr., J. E. Peralta, F. Ogliaro, M. J. Bearpark, J. J. Heyd, E. N. Brothers, K. N. Kudin, V. N. Staroverov, T. A. Keith, R. Kobayashi, J. Normand, K. Raghavachari, A. P. Rendell, J. C. Burant, S. S. Iyengar, J. Tomasi, M. Cossi, J. M. Millam, M. Klene, C. Adamo, R. Cammi, J. W. Ochterski, R. L. Martin, K. Morokuma, O. Farkas and J. B. Foresman, D. J. Fox, *Gaussian 16, Revision C.01*, Gaussian, Inc., Wallingford CT, 2016.

[S23] E. D. Glendening, J. K. Badenhoop, A. E. Reed, J. E. Carpenter, J. A. Bohmann, C. M. Morales, P. Karafiloglou, C. R. Landis and F. Weinhold, *NBO 7.0*, 2018.

[S24] Report for *NBO 7.0*: E. D. Glendening, C. R. Landis and F. Weinhold, *J. Comput. Chem.*, 2019, **40**, 2234–2241.

[S25] Z. Chen, C. S. Wannere, C. Corminboeuf, R. Puchta and P. v R. Schleyer, *Chem. Rev.*, 2005, **105**, 3842–3888.

[S26] R. Herges and D. Geuenich, *J. Phys. Chem. A*, 2001, **105**, 3214–3220.

[S27] M. J. S. Phipps, T. Fox, C. S. Tautermann and C.-K. Skylaris, *Chem. Soc. Rev.*, 2015, **44**, 3177–3211.

[S28] Articles for *GAMESS*: (a) M. W. Schmidt, K. K. Baldridge, J. A. Boatz, S. T. Elbert, M. S. Gordon, J. H. Jensen, S. Koseki, N. Matsunaga, K. A. Nguyen, S. Su, T. L. Windus, M. Dupuis and J. A. Montgomery, *J. Comput. Chem.*, 1993, **14**, 1347–1363; (b) M. S. Gordon and M. W. Schmidt, *In Theory and Applications of Computational Chemistry: The First Forty Years*, eds. C. E. Dykstra, G. Frenking, K. S. Kim, G. E. Scuseria, Elsevier, 2005, pp.1167–1189.

[S29] Report for FMO: K. Kitaura, E. Ikeo, T. Asada, T. Nakano and M. Uebayasi, *Chem. Phys. Lett.*, 1999, **313**, 701–706.

[S30] Report for pair interaction energy decomposition analysis (PIEDA): D. G. Fedorov and K. Kitaura, *J. Comput. Chem.*, 2007, **28**, 222–237.

[S31] Report for natural energy decomposition analysis (NEDA): (a) E. D. Glendening and A. Streitwieser, *J. Chem. Phys.*, 1994, **100**, 2900–2909; (b) E. D. Glendening, *J. Am. Chem. Soc.*, 1996, **118**, 2473–2482; (c) G. K. Schenter and E. D. Glendening, *J. Phys. Chem.*, 1996, **100**, 17152–17156.

4. Solution-state and solid-state properties

Electrochemical analysis. Cyclic voltammograms (CVs) were measured under Ar atmosphere in CH_2Cl_2 solutions containing the sample and $TBAPF_6$ (0.1 M) as a supporting electrolyte using an ALS/CH Instruments 619E electrochemical analyzer with a glassy-carbon disk working electrode (3-mm diameter), an $Ag/AgNO_3$ (0.010 M) reference electrode, and a Pt counter electrode.

ESR measurements. ESR measurements were performed by a JEOL JES-FA200 spectrometer equipped with an OXFROD ESR900 cryostat. The measurements temperature was controlled by OXFORD mercuryITC temperature controller in the temperature range from 5 K to 294 K.

Magnetic measurements and fittings. Magnetic measurements were performed by a Quantum Design MPMS-3 SQUID magnetometer under dc field of 1 T in the 2–300 K. The obtained data were analyzed using the Curie-Weiss law to determine Curie-Weiss temperatures (θ).

Method for steady-state absorption measurement of a single crystal. Steady-state absorption spectrum of a single crystal was measured by an inverted optical microscope (IX71, Olympus). Light from a 50 W halogen lamp positioned above the sample stage was used as the incident light and focused onto a single microcrystal using a ×40 objective lens (NA 0.40). The transmitted light was collected with a ×60 objective lens (NA 0.70) and passed through a confocal pinhole (1 μm in diameter) to spatially select the monitoring area. The transmitted-light intensity was measured with a CCD camera (DU970P, Andor) coupled to a polychrometer (250is, Chromex). The absorption spectrum was calculated using light transmitted through a coverslip without a crystal as the reference.

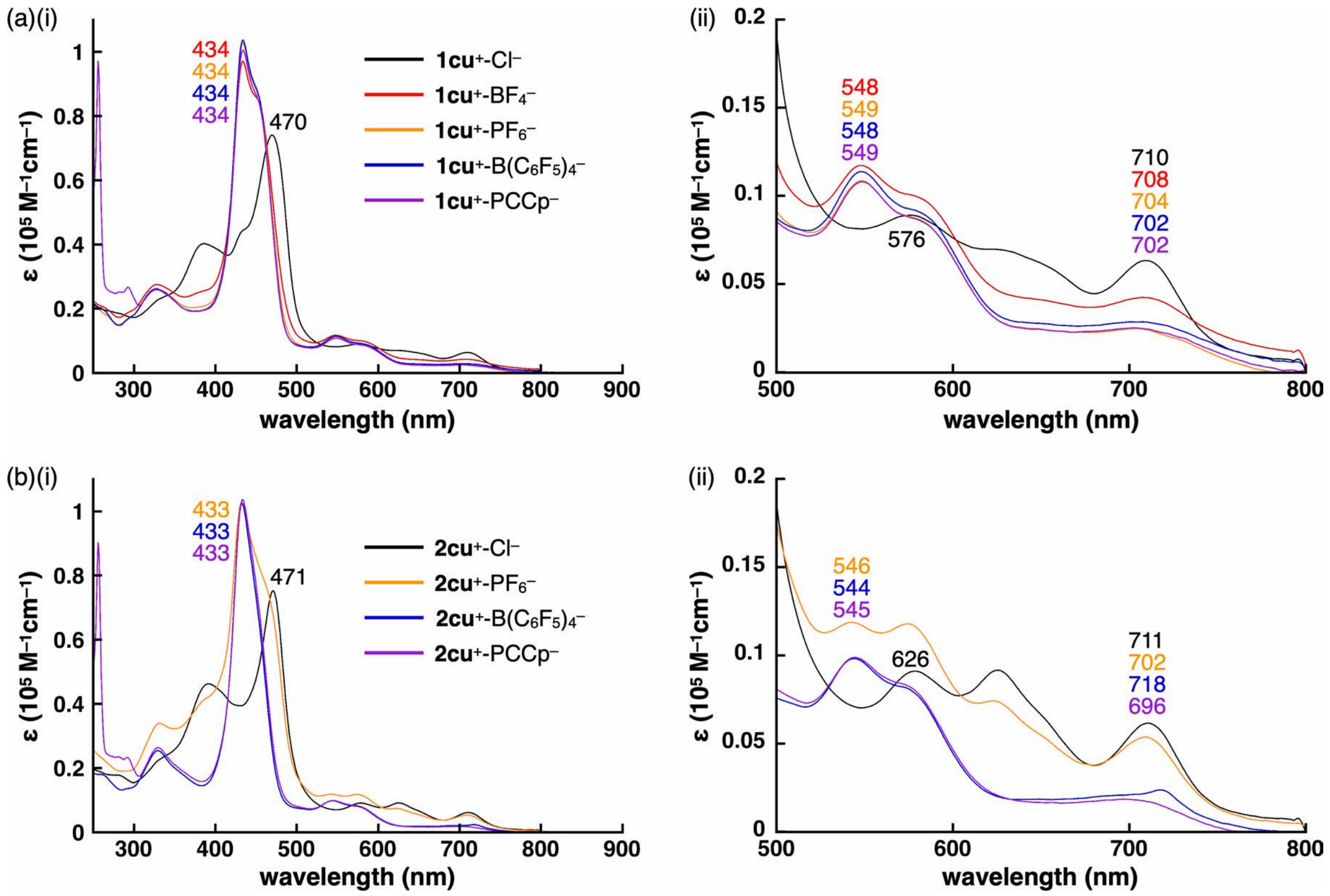

Fig. S95 UV/vis absorption spectra of (a) **1cu**$^+$-X^- (X^- = Cl^-, BF_4^-, PF_6^-, $B(C_6F_5)_4^-$, $PCCp^-$) and (b) **2cu**$^+$-X^- (X^- = Cl^-, PF_6^-, $B(C_6F_5)_4^-$, $PCCp^-$) in CH_2Cl_2 (1×10^{-5} M) as (i) wide-range and (ii) enlarged versions. In **2cu**$^+$-PF_6^-, Soret and Q bands were different from those of **2cu**$^+$-$B(C_6F_5)_4^-$ and **2cu**$^+$-$PCCp^-$, suggesting the axially coordinated counteranions to **2cu**$^+$ due to the enhanced Lewis acidity.

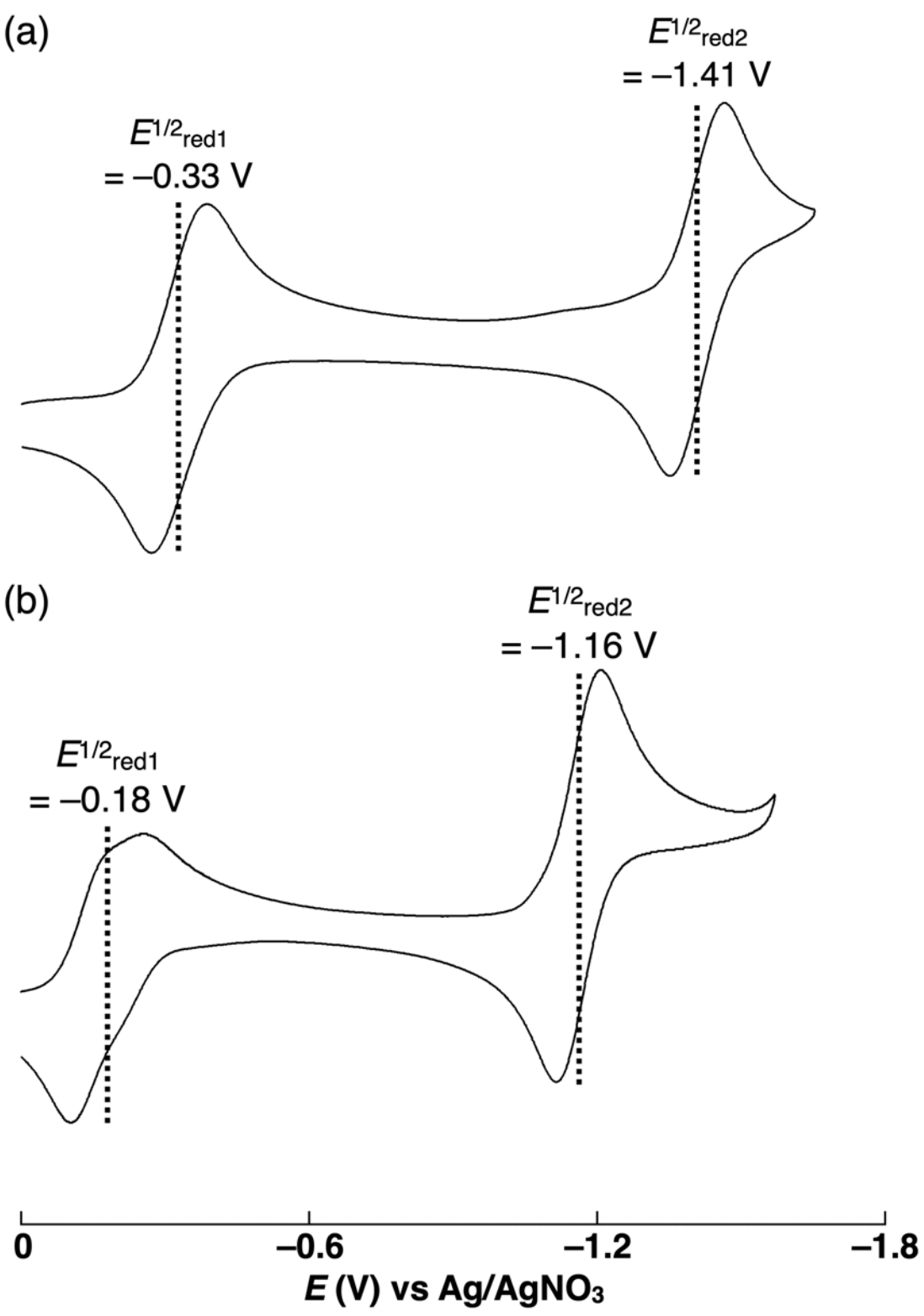

Fig. S96 Cyclic voltammograms (CVs) of (a) **1cu**$^{+}$-PF_6^{-} and (b) **2cu**$^{+}$-PF_6^{-} in CH_2Cl_2 (1.0×10^{-3} M) containing $TBAPF_6$ (0.1 M) as an electrolyte under Ar atmosphere at a scan rate of 100 mV/s.

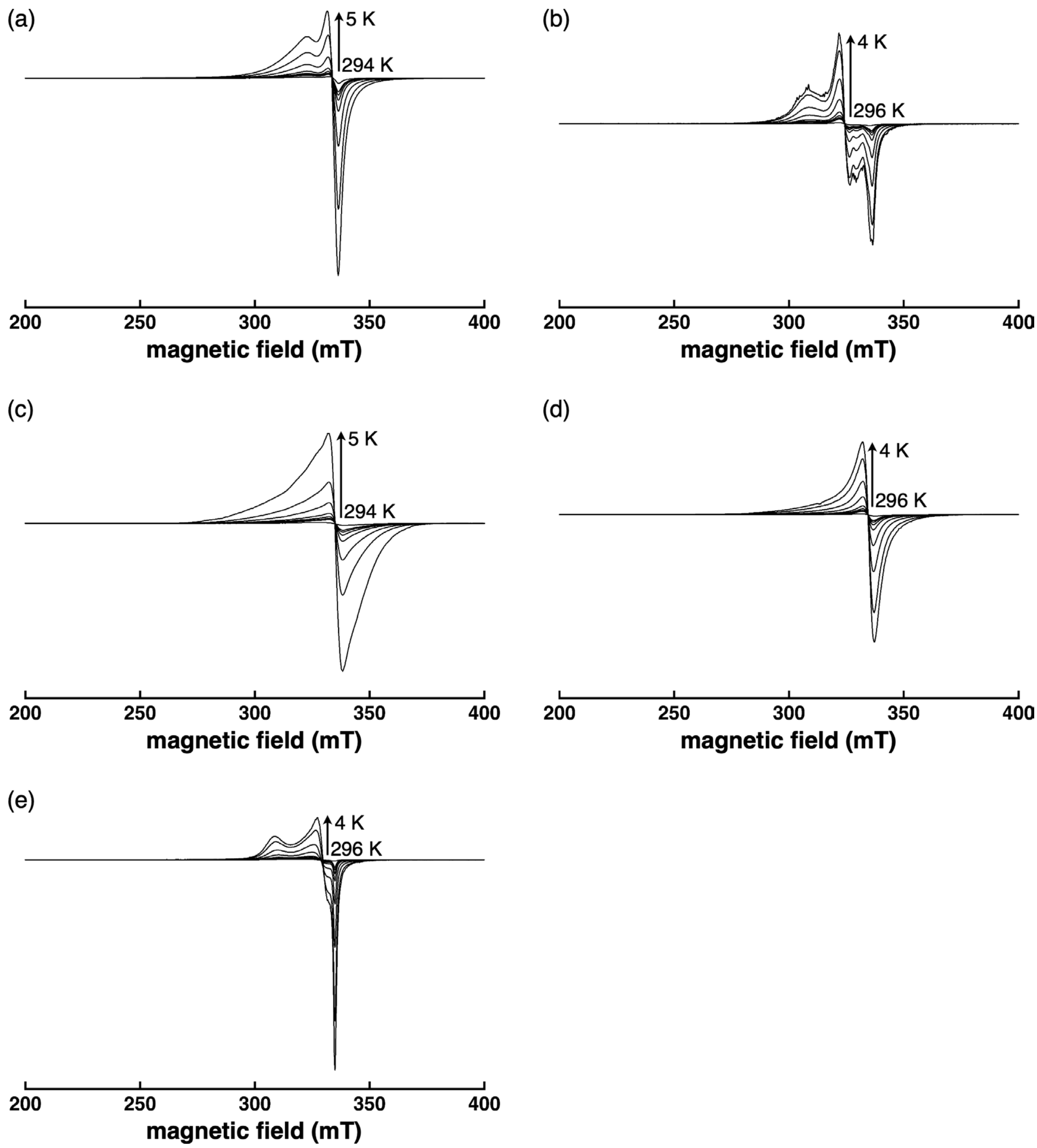

Fig. S97 Solid-state VT ESR spectra of (a) **1cu**$^+$-BF_4^- (g = 2.043, 2.043, 2.199, $\tilde{g}$ = 2.095), (b) **1cu**$^+$-PF_6^- (g = 2.035, 2.110, 2.227, $\tilde{g}$ = 2.124), (c) **1cu**$^+$-$B(C_6F_5)_4^-$ (g = 2.040, 2.040, 2.129, $\tilde{g}$ = 2.070), (d) **1cu**$^+$-PCCp$^-$ (g = 2.035, 2.045, 2.167, $\tilde{g}$ = 2.082), and (e) **2cu**$^+$-PCCp$^-$ (g = 2.042, 2.076, 2.217, $\tilde{g}$ = 2.112) measured as solid materials.

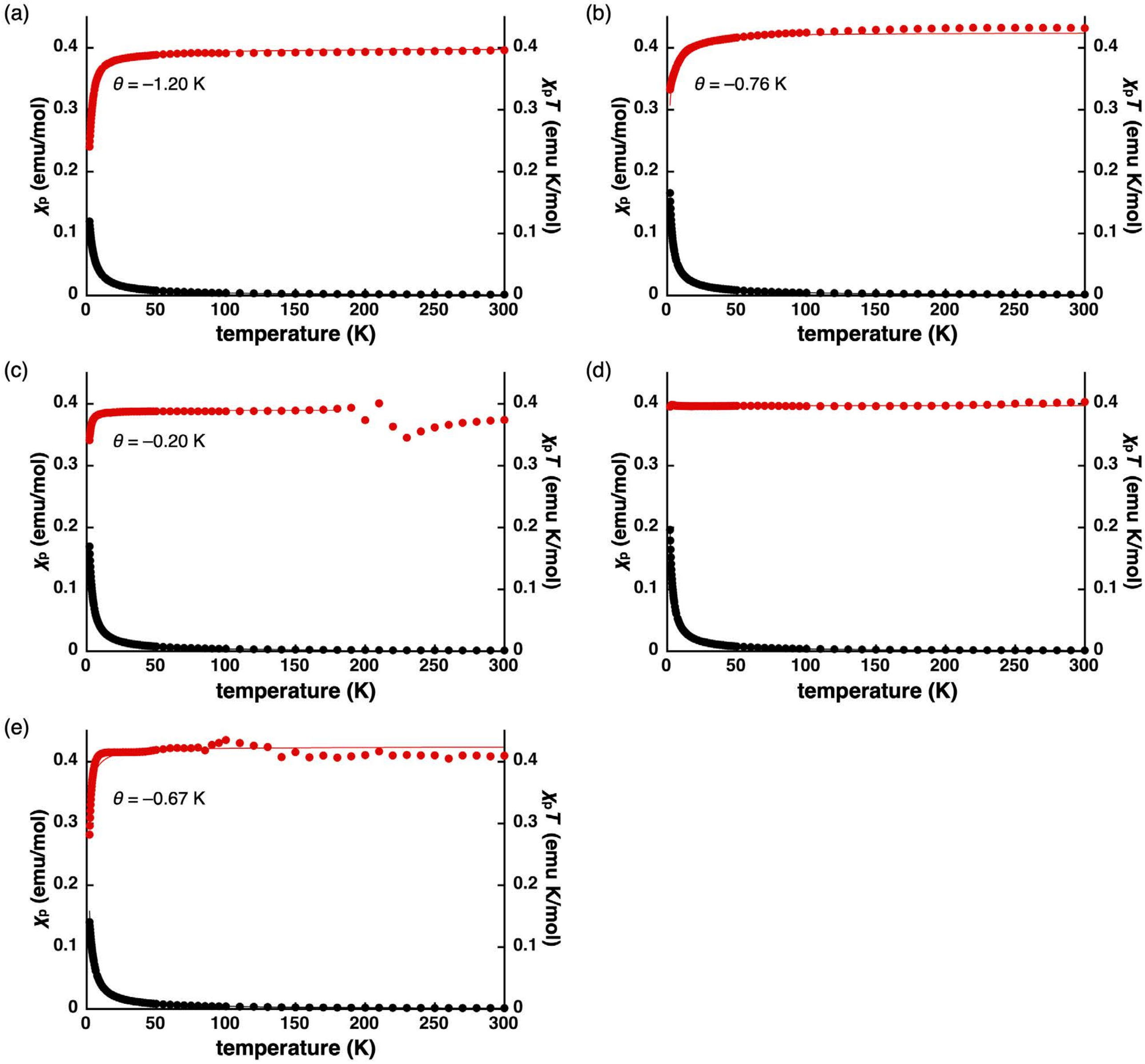

Fig. S98 Temperature dependence of the molar magnetic susceptibility (χ_{P}: black, $\chi_{\mathrm{P}}T$: red) and solid lines represent Curie-Weiss fits with Curie-Weiss temperatures θ (K) of (a) **1cu**$^{+}$-BF_4^{-} ($\theta = -1.20$), (b) **1cu**$^{+}$-PF_6^{-} ($\theta = -0.76$), (c) **1cu**$^{+}$-FABA^{-} ($\theta = -0.20$), (d) **1cu**$^{+}$-PCCp^{-}, and (e) **2cu**$^{+}$-PCCp^{-} ($\theta = -0.67$) measured as powder samples. No detectable Curie-Weiss temperature was obtained for **1cu**$^{+}$-PCCp^{-}. In (c), θ was obtained by fitting the χ_{P} data below 180 K because χ_{P} was not constant above 180 K. The variation in θ correlates with differences in the ion-pairing assembly modes, including intra/interdimer distances, structural disorder of thiaporphyrin cores, and peripheral substituents, indicating the importance of ion-pairing assembly control in modulating magnetic interactions. To investigate the overlap of the magnetic orbitals of the stacked cation dimers, the calculations were performed with the ORCA program at GD3BJ-UB3LYP/Def2TZVP. The broken-symmetry (BS) calculations of the stacked cation dimer in the single-crystal X-ray structure of **1cu**$^{+}$-BF_4^{-} revealed that the α and β spins are localized on the two Cu centres, respectively. Although the overlap between the magnetic orbitals is very small, the magnetic coupling constant (J) was calculated to be -5.22 cm^{-1} using the Yamaguchi scheme, suggesting antiferromagnetic interactions within the stacked cation dimers.

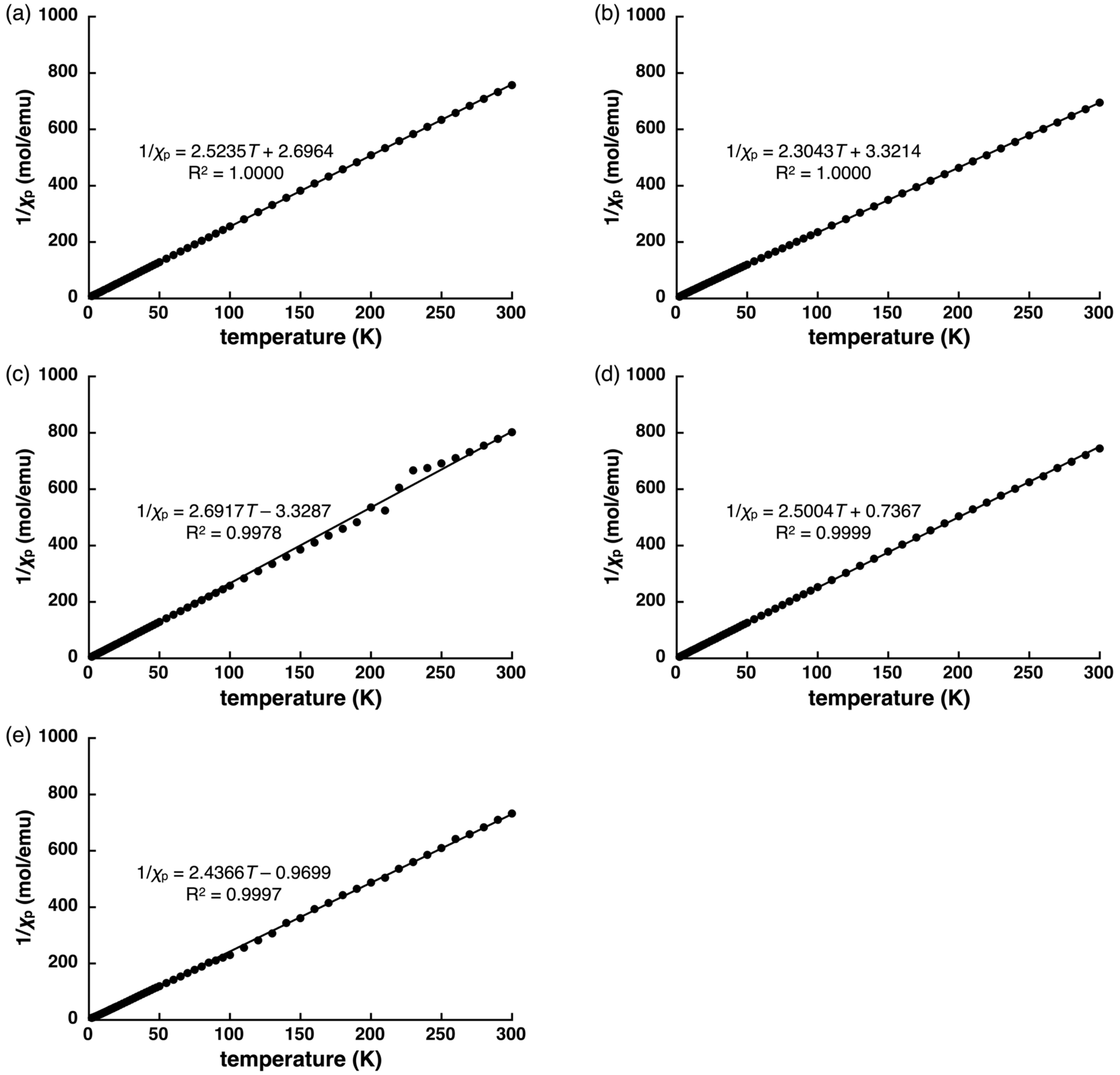

Fig. S99 Temperature dependence of the inverse molar magnetic susceptibility ($1/\chi_p$). Solid lines represent liner fits for (a) **1cu**$^+$-BF_4^-, (b) **1cu**$^+$-PF_6^-, (c) **1cu**$^+$-FABA$^-$, (d) **1cu**$^+$-PCCp$^-$, and (e) **2cu**$^+$-PCCp$^-$ measured as powder samples.

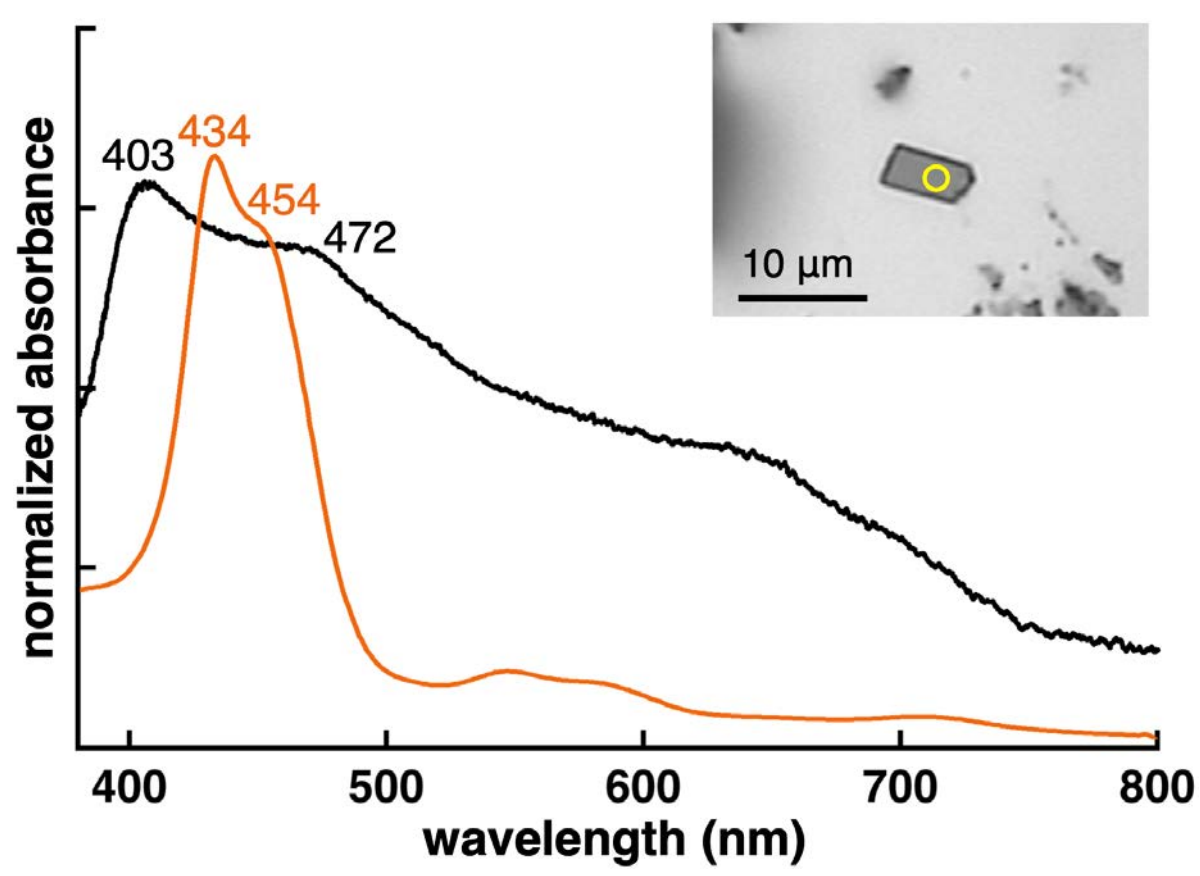

Fig. S100 UV/vis absorption spectra of the single crystal (black) and the solution state (orange, 1×10^{-5} M in CH_2Cl_2) of **1cu**$^+$-BF_4^- (inset: optical microscopy image of a single crystal of **1cu**$^+$-BF_4^- with the yellow circle indicating the position where UV/vis absorption measurement was conducted).
